# New reductive rearrangement of *N*-arylindoles triggered by the Grubbs–Stoltz reagent Et_3_SiH/KO^*t*^Bu†

**DOI:** 10.1039/d0sc00361a

**Published:** 2020-03-11

**Authors:** Andrew J. Smith, Daniela Dimitrova, Jude N. Arokianathar, Krystian Kolodziejczak, Allan Young, Mark Allison, Darren L. Poole, Stuart G. Leach, John A. Parkinson, Tell Tuttle, John A. Murphy

**Affiliations:** Department of Pure and Applied Chemistry, University of Strathclyde 295 Cathedral Street Glasgow G1 1XL UK john.murphy@strath.ac.uk tell.tuttle@strath.ac.uk; GlaxoSmithKline Medicines Research Centre Gunnels Wood Road, Stevenage SG1 2NY UK

## Abstract

*N*-Arylindoles are transformed into dihydroacridines in a new type of rearrangement, through heating with triethylsilane and potassium *tert-*butoxide. Studies indicate that the pathway involves (i) the formation of indole radical anions followed by fragmentation of the indole C2–N bond, and (ii) a ring-closing reaction that follows a potassium-ion dependent hydrogen atom transfer step. Unexpected behaviors of ‘radical-trap’ substrates prove very helpful in framing the proposed mechanism.

## Abstract 2

*N*-Arylindoles are transformed into dihydroacridines in a new type of rearrangement, through heating with triethylsilane and potassium *tert-*butoxide.

## Graphical abstract

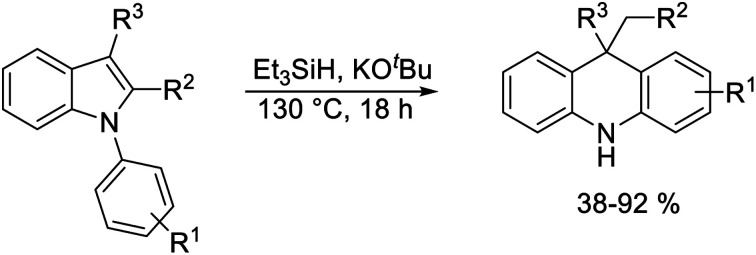

## Introduction

Indoles are an important class of compounds that feature widely in medicinal chemistry. Accordingly, the synthesis and reactivity of indoles have both been well explored. The indole nucleus is electron-rich, and indoles routinely act as aromatic nucleophiles, principally undergoing substitution by electrophiles or addition of electrophiles at the 2, 3-double-bond.

**Scheme 1:**
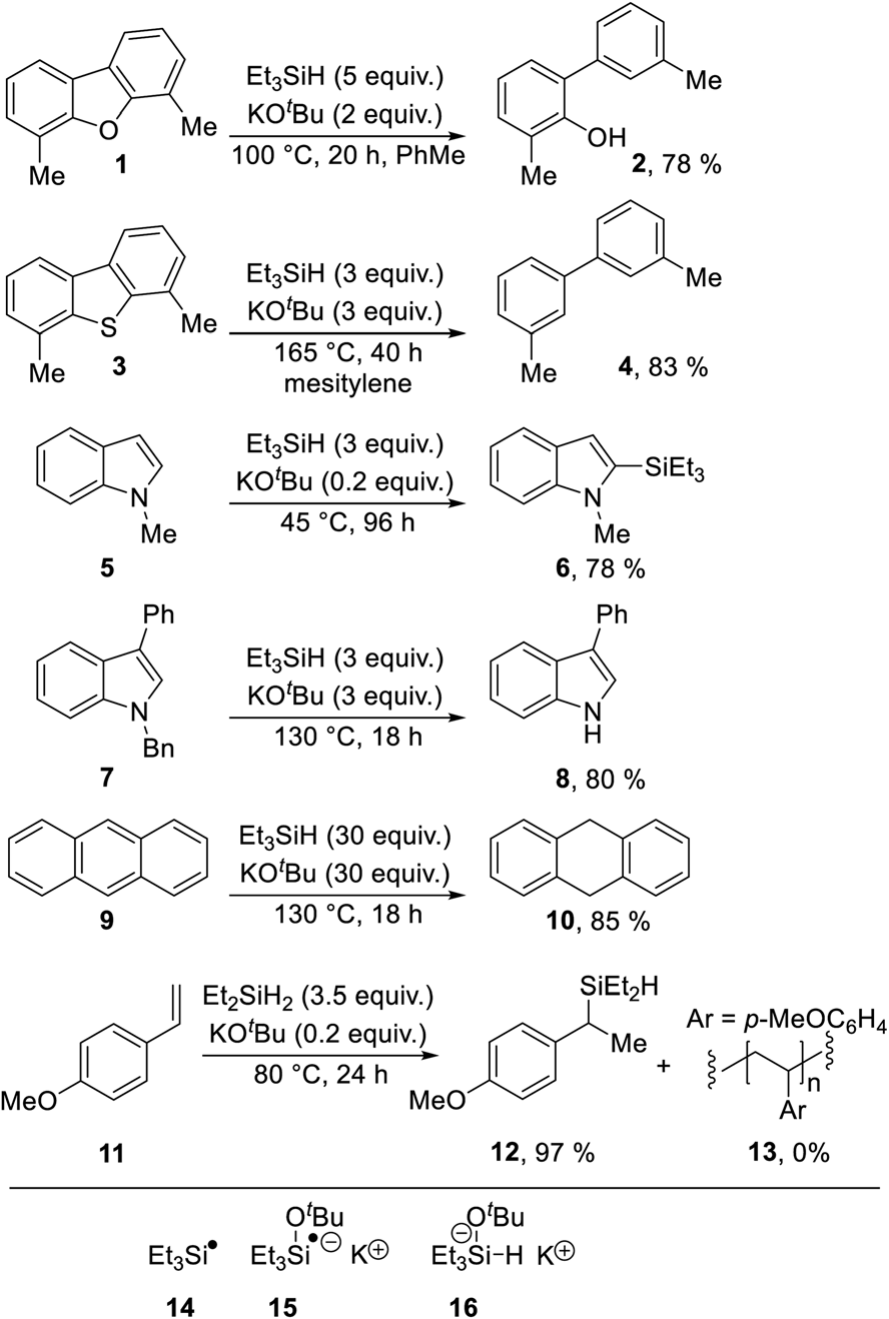
Selected transformations with both (i) triethylsilane or diethylsilane and (ii) potassium *tert-*butoxide.

In view of their position as electron-rich substrates, reductive transformations of indoles are very rare,^1^ but we now report a new radical-based rearrangement of *N*-arylindoles that arises from reductive activation. The reaction is brought about by the Grubbs–Stoltz reagent,^2–9^ resulting from heating triethylsilane with potassium *tert*-butoxide. Since its discovery in 2013, this reagent has been shown to drive a range of useful transformations, including cleavage of Ar–O (Scheme 1, **1** → **2**)^2,5^ and Ar–S bonds (**3** → **4**),^5^ regioselective C-silylation of indoles (**5** → **6**),^3,4,6,7^ reductive debenzylation of *N*-benzylindoles (**7** → **8**)^8^ and reduction of unsaturated hydrocarbons (**9** → **10**).^8^ A wide range of mechanisms has been proposed for these transformations featuring diverse intermediates, including (i) silyl radicals **14**,^2,5,8,9^ (ii) silyl radical anions, *e.g.***15** (ref. 6, 8 and 9) and (iii) silanate anions, *e.g.***16**;^2,6,7,9^ leading to extensive discussion in the literature. This is illustrated by both open-shell and closed-shell mechanisms being proposed by the same authors in back-to-back papers, as possible pathways for converting **5** → **6**.^6,7^

Most recently, in 2019, a unique role for the closely related KO^*t*^Bu + Et_2_SiH_2_ (as well as KO^*t*^Bu + Et_3_SiH) in hydrosilylation of styrenes (**11** → **12**) was proposed by Jeon *et al.*, and supported with good evidence in a reaction that critically depends on K^+^–arene interactions. (In the absence of potassium ions, polymer **13** is formed instead of **12**).^10^

Thus, this apparently simple mixture of base and silane provides great diversity in its reactions. Its applications already include methods for the desulfurisation of fuels^5^ and for the silylation of amines,^11^ together with proposed applications to the controlled cleavage of lignins.^2,5^ To harness its chemistry, further understanding is clearly needed of the mechanisms of its reactions. In this paper, we report a new rearrangement that transforms *N*-arylindoles to dihydroacridines, and we provide evidence supporting (i) an initial fragmentation of the C2–N bond of indole radical anions, followed by (ii) K^+^ ion-assisted hydrogen atom transfer chemistry for the conversion of the ring-opened intermediates to dihydroacridines.

**Scheme 2:**
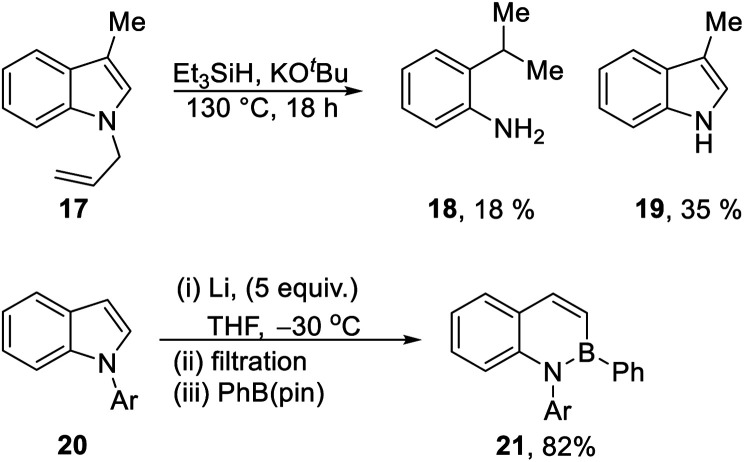
(a) Reductive ring-opening of *N*-allylindole **17** (ref. 8) and *N*-arylindole **20**.^1^

Our recent report^8^ used Et_3_SiH/KO^*t*^Bu to transform *N*-benzylindoles *e.g.***7** to indoles (**8**), and we proposed that electron transfer from radical anion **15** to the substrate could induce the fragmentation of the *N*-benzyl bond. In the same paper, *N*-allyl-3-methylindole **17** (Scheme 2) was converted to 3-methylindole **19** (35%); unexpectedly, the reaction also afforded 2-iso-propylaniline **18** (18%). Reductive ring-opening reactions of indoles, as seen in the formation of **18**, were unknown at the time and inspired the investigation reported in this paper; however, very recently, elegant studies by Yorimitsu *et al.*^1^ have reductively opened indoles **20** with excess Li powder, trapping the intermediate vinyl and amidyl dianions with boron electrophiles to afford benzazaborin products **21** in high yield.^1,12^

**Scheme 3:**
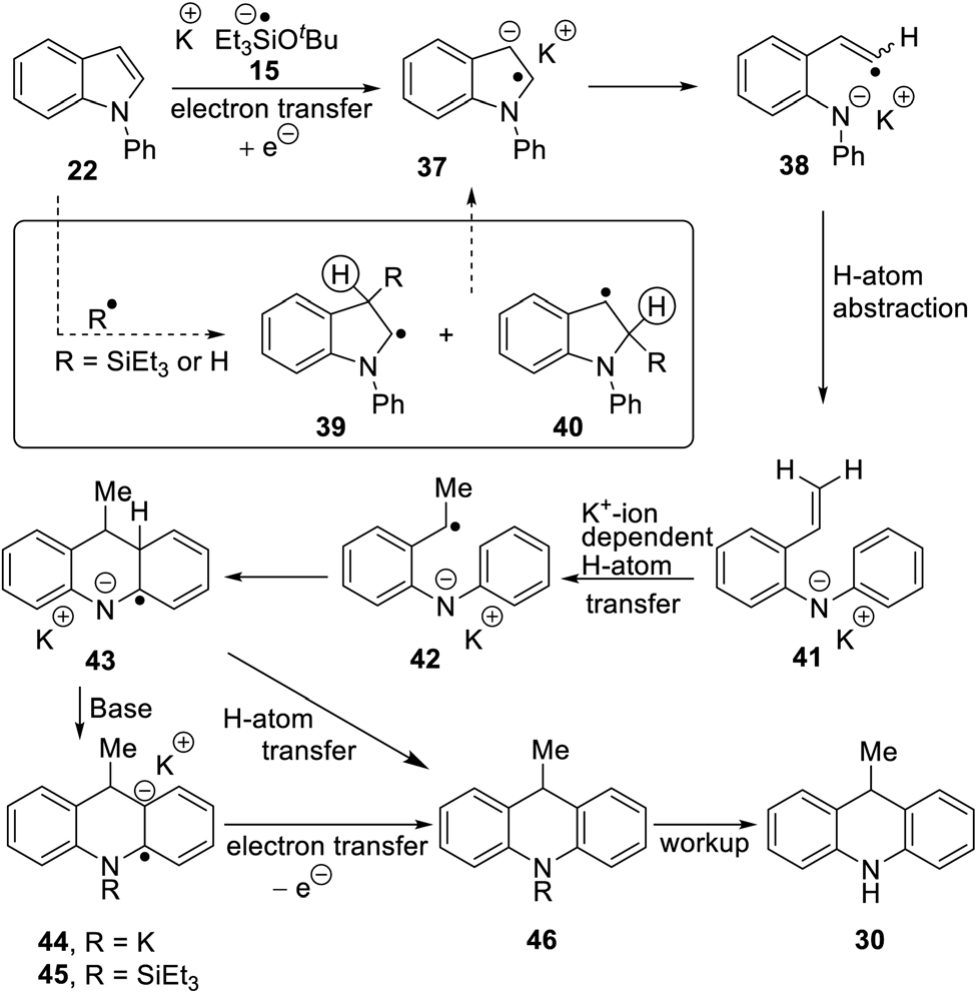


A selection of *N*-arylindoles **22–29** was prepared (see ESI†) and tested under the conditions shown in Table 1. Indole heterocyclic rings were cleaved, as the substrates underwent unexpected and facile conversion to 9,10-dihydroacridines **30–36** in moderate to excellent yields. Substituents were tolerated in the 2- and 3-positions of the indole (entries 1–7), as well as in the *N*-aryl group (entry 8). To rationalise the rearrangements, we adopt a working hypothesis for the mechanism, shown in Scheme 3. A key intermediate is radical anion **37**. This could form by electron transfer from donor **15** to the indole or, alternatively, by H-atom addition (H-atom addition is discussed later in this paper) to the indole ring system, to form **39** or **40** (R = H) or an isomer, followed by deprotonation under the basic conditions of the reaction. This radical anion then fragments to afford the distal radical anion **38**. Abstraction of a hydrogen atom by the vinyl radical (likely donors would include triethylsilane or silanate **16** or intermediates **39** or **40**) affords the styrene/amide anion **41**. This styrene now undergoes regioselective hydrogen atom addition as proposed by Jeon *et al.*^10^ to give benzyl radical anion **42**, and instead of forming a C–Si bond as in Jeon's example, a C–C bond is formed as cyclisation gives radical anion **43**. This could function directly as an H-atom donor to form **46** or, alternatively, deprotonation of the cyclohexadienyl radical then affords the electron-rich species **44** (or, following silylation, **45** (ref. 11)) which behaves as an electron donor.

**Table 1:** Conversion of indoles to dihydroacridines^a^

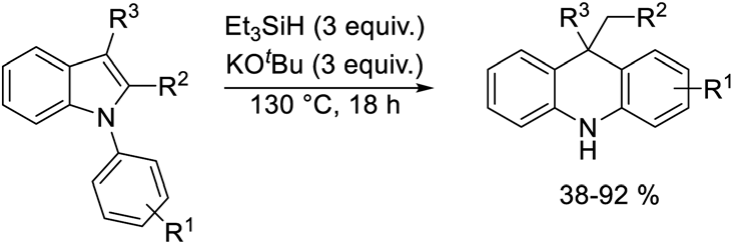			
Entry	Substrate	Product	Yield%
1	**22**, R^1^ = R^2^ = R^3^ = H	**30**, R^1^ = R^2^ = R^3^ = H	58
2	**23**, R^1^ = R^2^ = H, R^3^ = Me	**31**, R^1^ = R^2^ = H, R^3^ = Me	77
3	**24**, R^1^ = R^2^ = H, R^3^ = Et	**32**, R^1^ = H, R^2^ = R^3^ = Me	66
4	**25**, R^1^ = H, R^2^ = R^3^ = Me	**32**, R^1^ = H, R^2^ = R^3^ = Me	53
5	**26**, R^1^ = R^2^ = H, R^3^ = Ph	**33**, R^1^ = R^2^ = H, R^3^ = Ph	92
6	**27**, R^1^ = R^2^ = H, R^3^ = C_8_H_17_	**34**, R^1^ = R^2^ = H, R^3^ = C_8_H_17_	55
7	**28**, R^1^ = R^2^ = H, R^3^ = C_4_H_9_	**35**, R^1^ = R^2^ = H, R^3^ = C_4_H_9_	71
8	**29**, R^1^ = 4-Me, R^2^ = R^3^ = H	**36**, R^1^ = 2-Me, R^2^ = R^3^ = H	38

a Reactions were carried out neat (*i.e.* without added solvent).

**Fig. 1:**
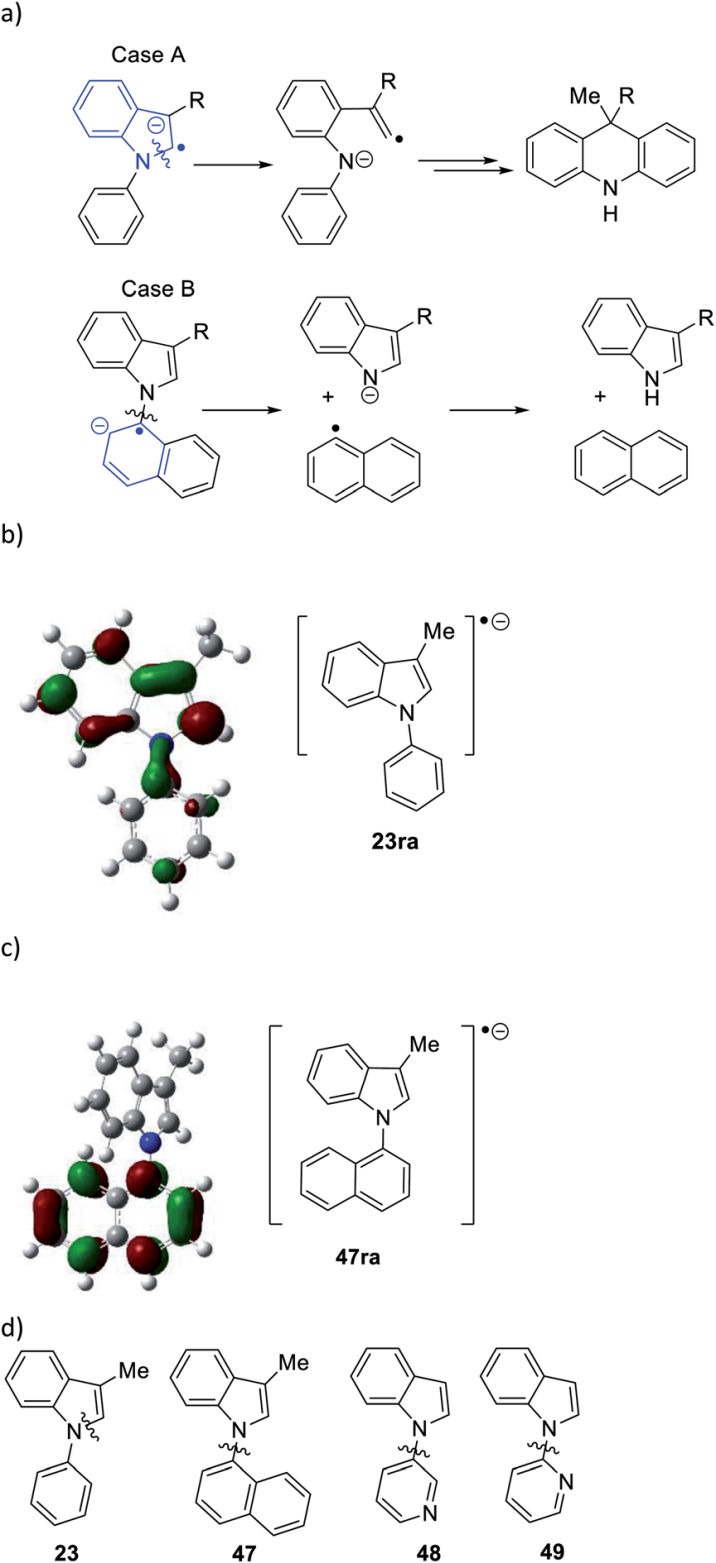
(a) Case A, when the unpaired electron in radical anion of *N*-arylindoles localises on the indole group in the radical anion of **23** (*i.e.***23ra**), then fragmentation of the indole nucleus results, while with the excess electron is housed in the *N*-aryl group in **47ra**, then cleavage of the indole *N*-aryl bond results (Case B); (b) radical anion **23ra** showing localisation of spin in the indole nucleus and (c) radical anion **47ra**, showing localisation of spin in the naphthyl group; (d) substrates **47** → **49** undergo indole *N*-aryl bond cleavage (for computational methods, see ESI†).

Considering the behavior of radical anion **37**, fragmentation of the 5-membered ring could depend on the excess electron of the radical anion being localised extensively in the indole ring system, rather than in the *N*-phenyl group (Fig. 1a, Case A). Computational studies performed on the 3-methyl analogue **23** are consistent with this (Fig. 1b). We rationalise that the greater delocalisation associated with the bicyclic π-system of the indole results in this preferential localisation of the excess electron density of the radical anion.

We investigated further substrates by computation and experiment, starting with the *N*-naphthyl substrate **47**. In this case, the naphthyl π-system is the preferred site for the radical anion (Fig. 1c). In the laboratory, that should result in cleavage of the *N*-naphthyl bond to form an indolyl anion and a naphthyl radical, rather than in cleavage of the indole nucleus (Fig. 1a, Case B). Accordingly, the α-naphthyl substrate **47** (Fig. 1d) was prepared. Under the standard reaction conditions, the predicted cleavage occurred to form 3-methylindole **19** (55%), and none of the corresponding dihydroacridine was detected.

Two additional substrates, **48** and **49**, were prepared, featuring pyridine rings. Computation (see ESI†) suggested that, due to the electron-deficient nature of these rings, the radical anions of these substrates would house the added electron in the pyridine ring, leading again to [indole N]–pyridine bond cleavage, rather than cleavage of the indole nucleus. This was indeed the case, with indole being isolated in 48% and 39% from **48** and **49** respectively following workup, and no evidence of cleavage of the indole nucleus. Thus, the cleavages of substrates **22–29**, and **47–49** follow the regioselectivity that is predicted from a study of the respective radical anions, providing strong evidence that radical anions are intermediates in these reactions.

To investigate the proposed mechanism involving SET, several other reaction conditions were investigated (Table 2) with substrate **22**. The standard conditions for Et_3_SiH + KO^*t*^Bu afforded **30** in 58% yield (entry 1). With a view to forming the radical anion of Me_3_SiO^*t*^Bu, analogous to **15**, without using triethylsilane or any other compound that contains a Si–H group, hexamethyldisilane was treated with the radical anion, potassium di-*tert-*butylbiphenylide (KDTBB) as reducing agent and KO^*t*^Bu as base. This system afforded product **30** (33%, entry 2). This successful outcome would result if the disilane were reduced to a silyl radical and a silyl anion by KDTBB. Addition of a silyl radical to *N*-phenylindole^7^ could give **39**, **40** (R = SiEt_3_, Scheme 3) or an isomeric radical. Both **39** and **40** have a labile hydrogen atom (circled) that can be removed either as a proton, *e.g.* by trimethylsilyl anion, to form Me_3_SiH, or as a hydrogen atom, *e.g.* by trimethylsilyl radical to again form Me_3_SiH, putting in place all the components for Scheme 3.

**Table 2:** Mechanistic probes

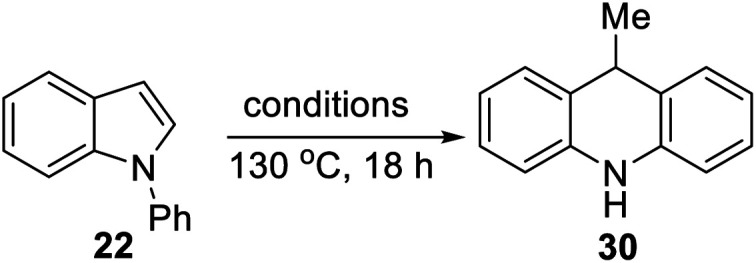		
Entry	Reagents (3 equiv. of each)	Yield 30 (%)
1	Et_3_SiH, KO^*t*^Bu	58
2	KDTBB, Me_6_Si_2_, KO^*t*^Bu	33
3	KDTBB, KO^*t*^Bu	0
4	Me_6_Si_2_, KO^*t*^Bu	0
5	KO^*t*^Bu	0

In the presence of KTDBB, but in the absence of any silane or disilane, no product **30** was formed (entry 3). When KTDBB was omitted (entry 4), no cleavage of the Si–Si bond could occur and so the reaction gave no rearranged product. Entry 5 shows that KO^*t*^Bu alone could not bring about the rearrangement. From these results, it was clear that (i) an appropriate silyl compound, (ii) KO^*t*^Bu and (iii) reducing power were all needed to bring about the rearrangement efficiently.

The requirement for all three components above was also shown when potassium metal was tested as a reducing agent in the presence of KO^*t*^Bu (see ESI†). This led to consumption of the indole substrates. Acridine products, rather than dihydroacridines, were produced, reinforcing that reductive activation^1^ of the indole nucleus leads to fragmentation of the indole 5-membered ring, but the absence of H-atom sources meant that the reaction of Scheme 3 was not supported; the acridine products were formed in very low yield (5%, see ESI file†). However, from this result and from the results of Yorimitsu,^1^ it is clear that reductive activation of the indole nucleus of *N*-arylindoles does lead to fragmentation of the 5-membered ring.^13^

**Scheme 4:**
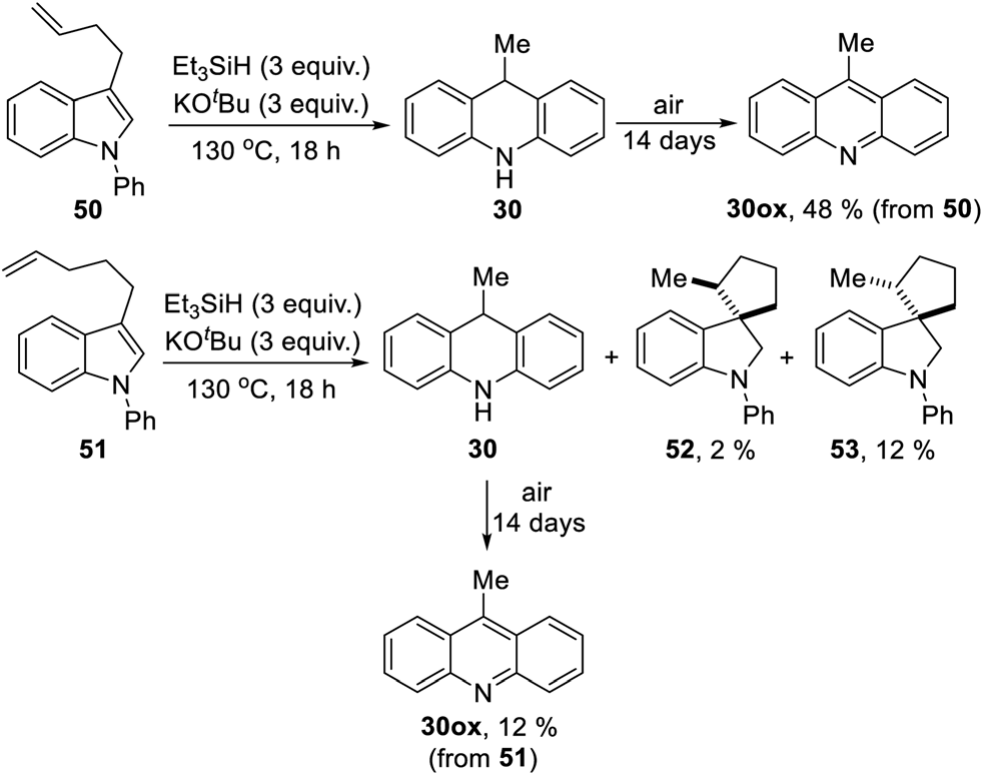


We now looked for evidence of reactive intermediates on the reaction pathway shown in Scheme 3, using substrates that incorporated radical clocks. These substrates provided most surprising outcomes and important mechanistic information. Substrates **50**, **51**, (Scheme 4), **61** (Scheme 6) and **73** (Scheme 7) were prepared and reacted under the standard conditions; the products were then analyzed to provide evidence of reaction intermediates.

Substrate **50** gave an unexpected result when it afforded dihydroacridine **30** in good yield, confirmed by NMR. For ease of isolation, the product was exposed to oxidation in air, affording **30ox** in 48% yield from **50**. For this to happen, the butenyl side-chain must be severed during the reaction. Substrate **51** also afforded dihydroacridine **30** as well as the diastereomeric spiroindolines **52** and **53**. The loss of the side-chains, seen in the formation of **30**, is clearly associated with the alkene group in the side-chain, since Table 1 shows that substrates with saturated side-chains, **27** and **28**, suffered no loss of their saturated alkyl side-chains.

**Scheme 5:**
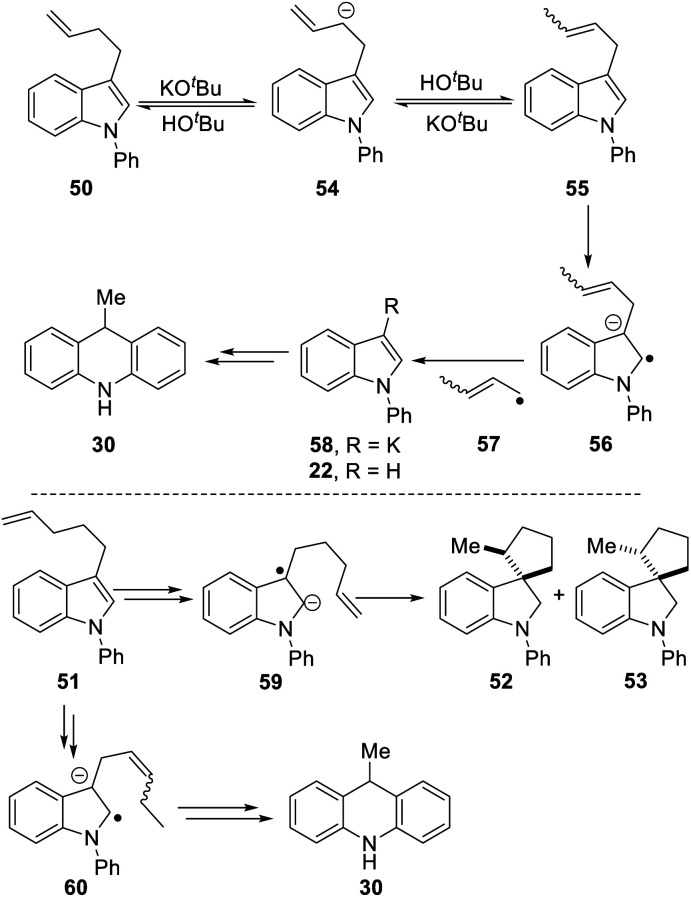


Scheme 5 proposes an explanation for both substrates **50** and **51**. Substrate **50** can suffer reversible deprotonation by *tert-*butoxide in the allylic position to afford anion **54**. Reprotonation of the anion can afford **55** which is converted to radical anion **56**. (We draw the formation of the radical anion occurring following the regioisomerisation of the alkene, but the opposite sequence may well occur). Radical anion **56** then fragments to the 2-butenyl radical **57**, leaving indolylpotassium **58**; following proton abstraction, this gives *N*-phenylindole **22**, which is then converted, as in Scheme 3, into 9-methyldihydroacridine **30**. (For NMR evidence in favor of the alkene migration in the side-chain of **50**, see ESI file†).

The outcome of reaction with substrate **51** can be explained in like manner. In this case, conversion to its radical anion **59** leads to cyclisation to diastereomeric radicals that abstract hydrogen atoms to afford **52** and **53**. On the other hand, if KO^*t*^Bu-induced regioisomerisation migrates the alkene double-bond sequentially along the side-chain, the substrate then is converted to radical anion **60**. This species can expel a delocalised pent-2-enyl radical, again forming indolylpotassium **58**, which, following proton abstraction, is ultimately transformed to 9-methyldihydroacridine **30**.

**Scheme 6:**
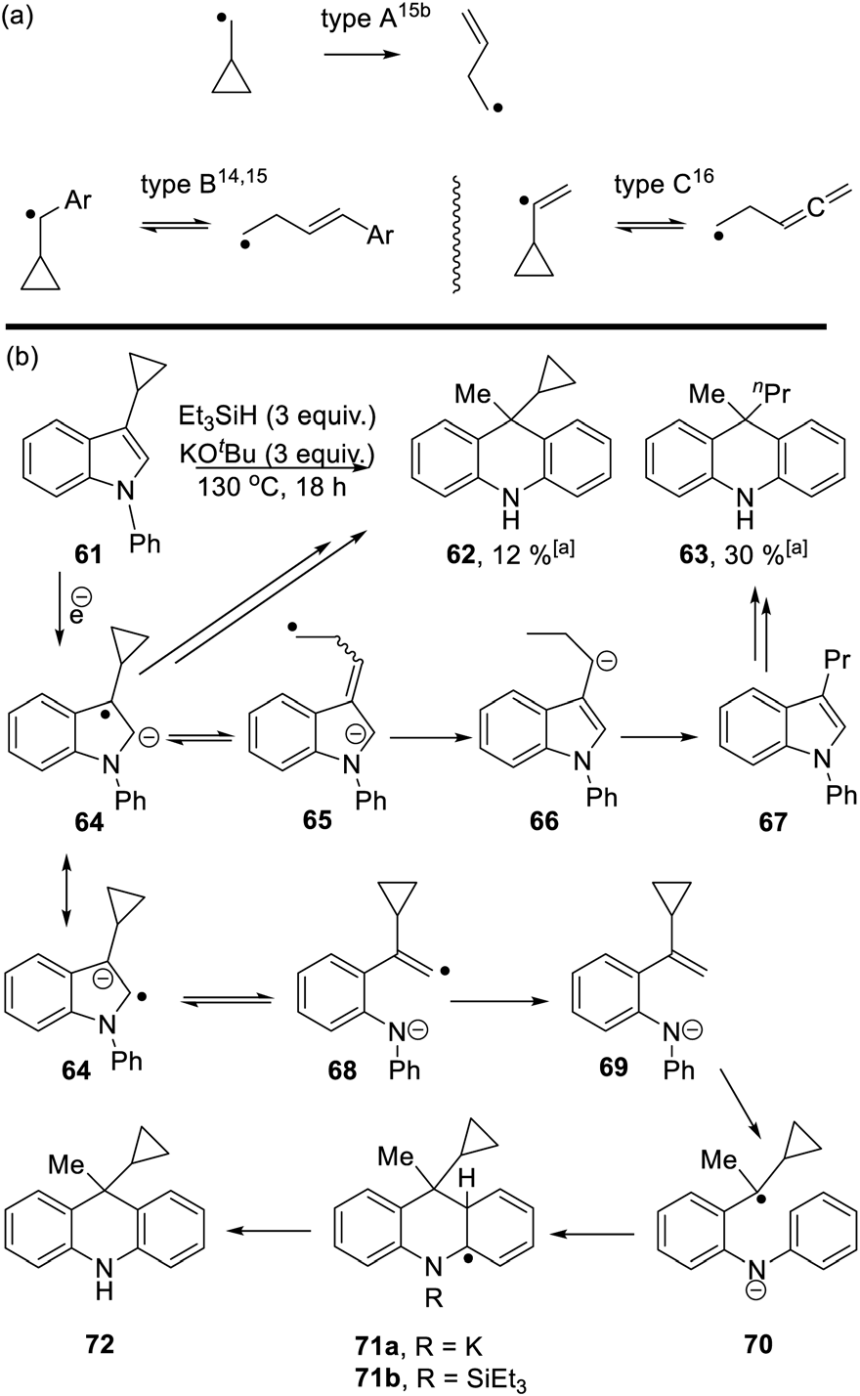


Studies were next carried out with substrate **61** and **73** bearing a cyclopropyl substituent as a radical probe^6,10^ (Scheme 6).

Cyclopropyl groups are routinely used as indicators of radical intermediates. Simple cyclopropylcarbinyl radicals (type A, Scheme 6a) ring-open very rapidly to form butenyl radicals.^14,15^ The rate of the reverse reaction is comparatively very slow, so that these reactions routinely afford ring-opened products as the sole products from reactions. However, when the initial radical carbon is benzylic (type B)^14,15^ or is a vinyl radical (type C),^16^ then the cyclopropyl ring-opening is readily reversible and cyclic and/or open-chain products can result. As seen below, these subtleties of cyclopropanes as probes of radicals can be important in understanding mechanisms.

Starting with substrate **61**, reaction with KO^*t*^Bu and Et_3_SiH afforded a mixture of cyclopropyl product **62** (12%) and *n-*propyl analogue **63** (30%). Following formation of radical anion **64**,^8^ fragmentation of the cyclopropyl ring would afford radical anion **65**. As radical anion **64** incorporates a benzylic cyclopropylcarbinyl radical, this type B reaction will likely be reversible and thus both **64** and **65** can be considered as likely intermediates. For **65**, abstraction of (i) a hydrogen *e.g.* from Et_3_SiH and (ii) a proton leads to 3-propyl-*N*-phenylindole **67**. This compound can then be processed, according to Scheme 3, to afford dihydroacridine **63** following workup.

On the other hand, fragmentation of the 5-membered ring in **64** affords radical anion **68**. Hydrogen atom abstraction leads to **69**. Regioselective hydrogen atom transfer^10^ to styrene **69** gives benzylic radical **70**. This is a cyclopropylbenzyl radical (type B) and so can open reversibly. Cyclisation affords radical anion **71a**. Aromaticity is restored as **72** is formed; this could proceed with the amide anion still in place, as in **71a**, or following silylation of the nitrogen as in **71b**, as mentioned in Scheme 3.^11^

**Scheme 7:**
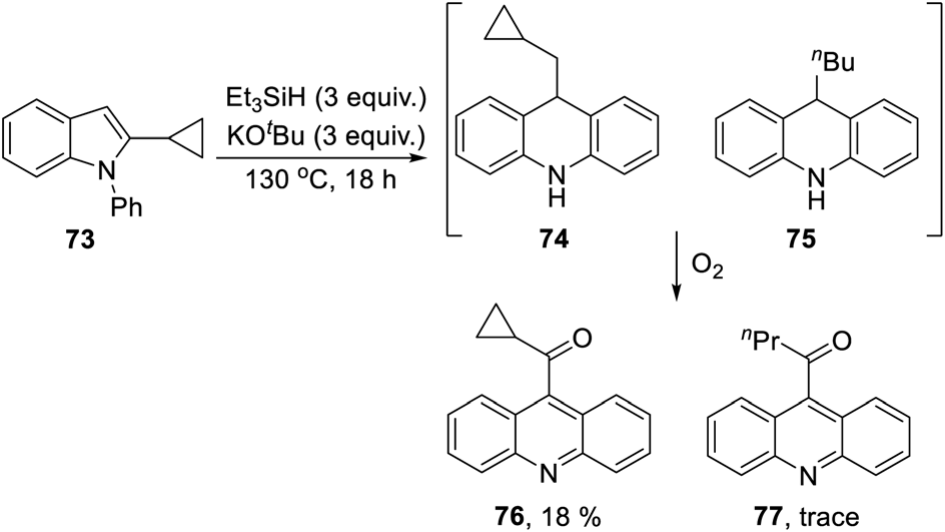


The 2-cyclopropyl substrate **73** (Scheme 7) was transformed into products from which **77** (trace amount) was separated and characterised. Exposure of the residue to air allowed isolation of acridine **76** (18%).

**Scheme 8:**
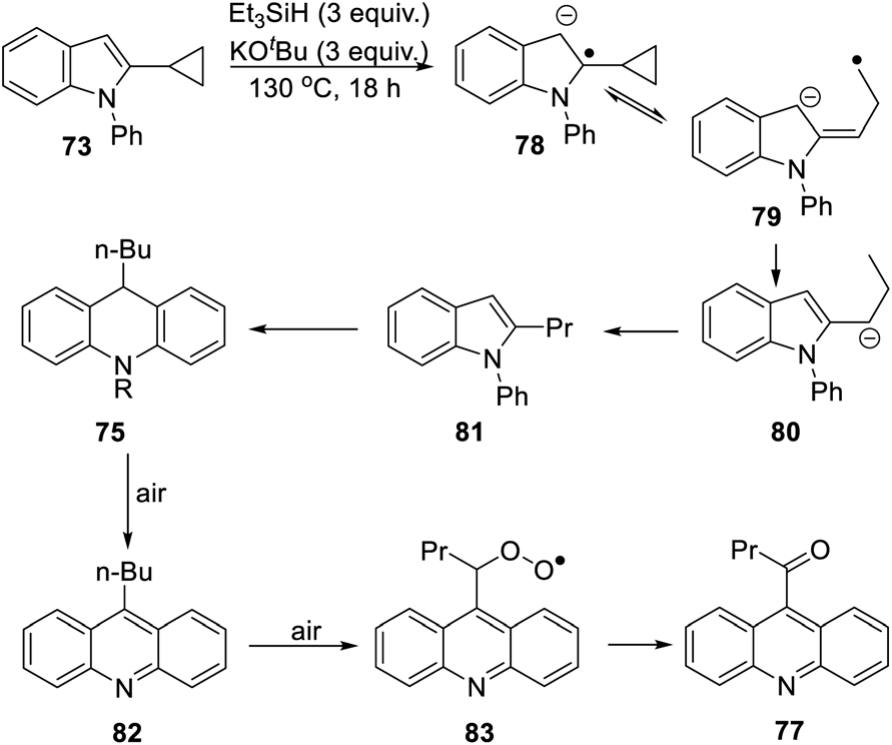


**Scheme 9:**
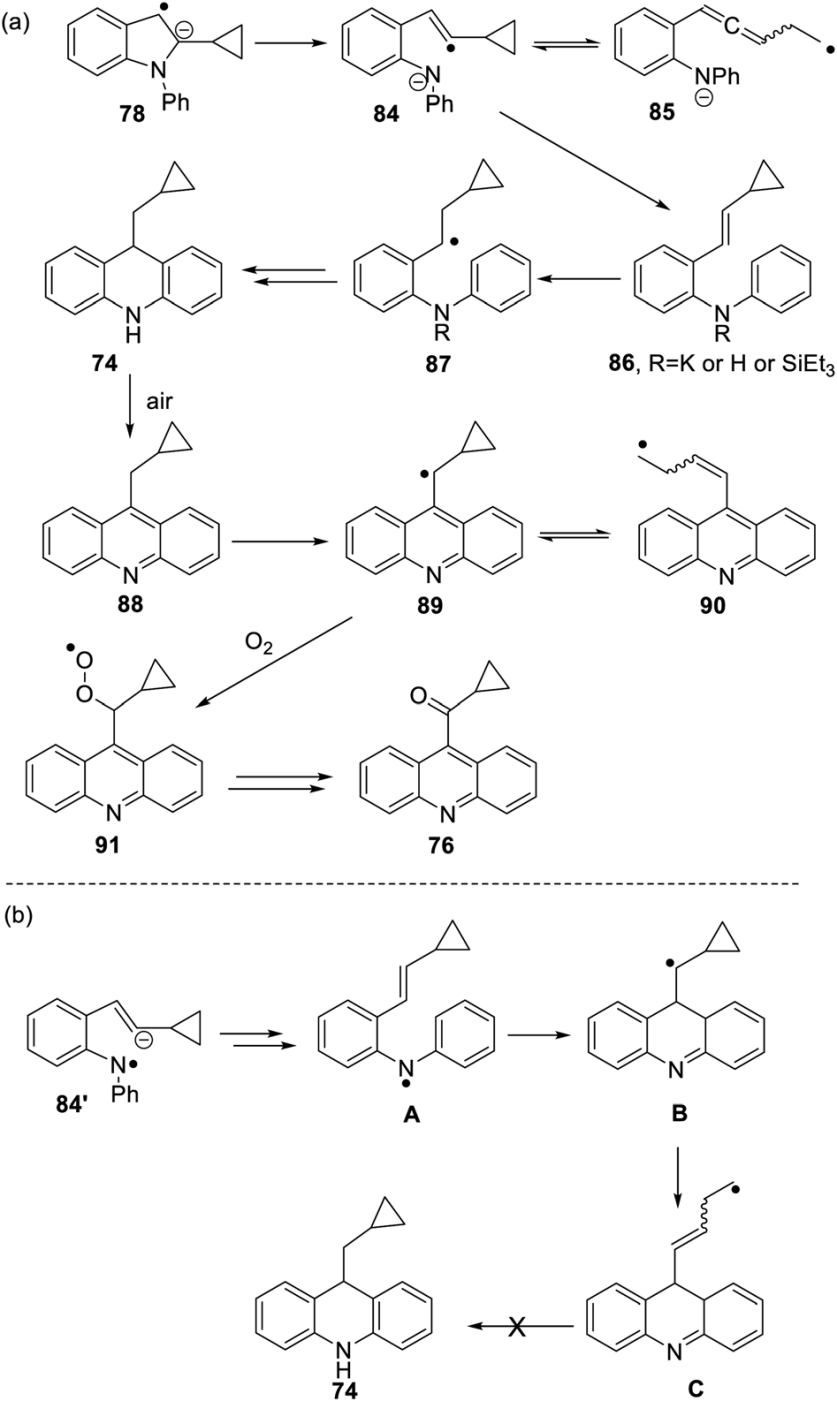


The formation of **77** follows pathways that are familiar from other examples in this paper, starting with the fragmentation of the 3-membered ring in radical anion **78**; (Scheme 8); the oxidation of **82** by air likely involves abstraction of a benzylic hydrogen atom, with the peroxyl radical **83** as key intermediate on the pathway to ketone **77**. The formation of compound **76** (Scheme 7) was also informative. Fragmentation of the 5-membered ring in **78** (see Scheme 9) leads to radical anion **84** which is a type C cyclopropylvinyl radical, likely to be in equilibrium with its ring-opened form **85**. No product derived from **85** was detected, but **84** did lead to observed product.

Hydrogen abstraction by radical **84** leads to vinyl cyclopropane **86**. Following Jeon,^10^ regioselective hydrogen atom addition gives radical **87**, which then proceeds to the cyclopropylmethyl-substituted dihydroacridine **74**. On deliberate exposure to air, aromatisation slowly occurs to yield **88**, followed by activation of the benzylic C–H, ultimately leading to ketone **76**.

Substrate **73** played an important role in our understanding of mechanism. An alternative route to the dihydroacridine products was initially considered [Scheme 9(b)] where fragmentation of the indole radical anion **78** would afford distal radical anion **84′**. Proton abstraction, giving **A** and radical cyclisation would yield radical **B**. However, this cyclopropylcarbinyl radical of type A would open to give radical **C**, and no cyclopropyl product would be detected from this intermediate. Accordingly, H-atom addition to the styrene **86** is the favored route.

**Scheme 10:**
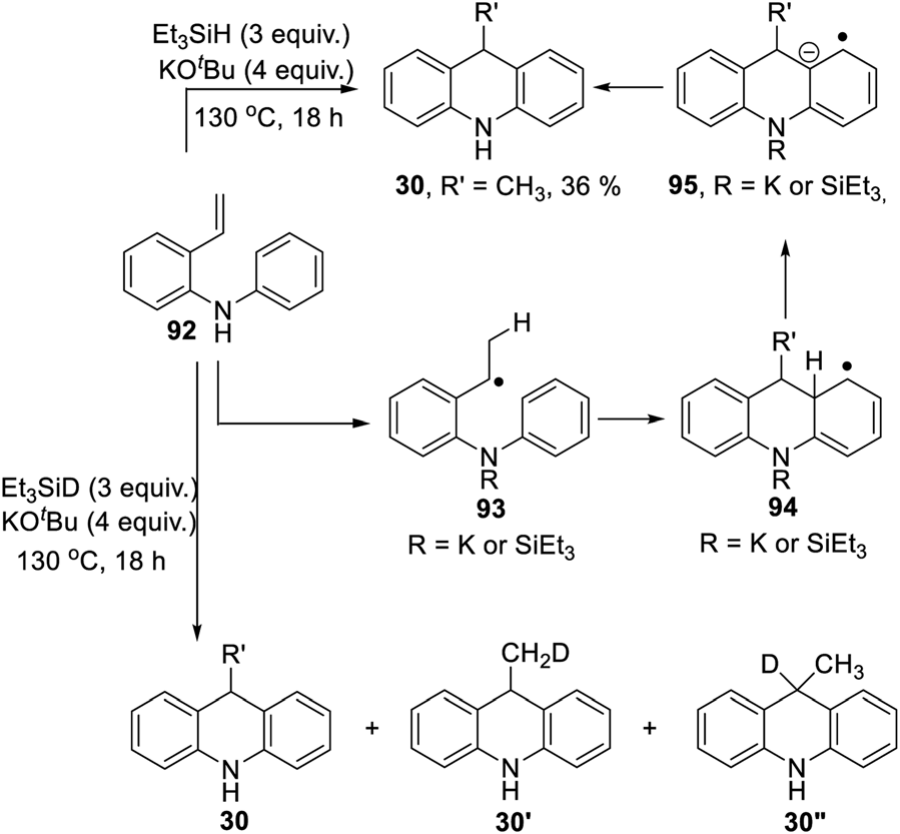


Scheme 3 proposed styrene **41** as a possible intermediate in the rearrangement reaction. To check its viability, substrate **92** was prepared (Scheme 10). On treatment with triethylsilane and KO^*t*^Bu, this afforded 9-methyldihydroacridine **30** (36%). The upper part of Scheme 10 shows the proposed pathway. Hydrogen atom addition would form radical **93**; notably, just a single H atom in **30** is proposed to derive from Et_3_SiH in this mechanism, and to be located in the methyl group of the product. To check this, the reaction was repeated using Et_3_SiD instead of Et_3_SiH. The ^2^H-NMR spectrum clearly showed labelling in the methyl group. The ^13^C {^1^H}-spectrum showed two resonances for the methyl carbon, one a 1:1:1 triplet, due to a CH_2_D group in **30′**, and the other a singlet, due to an undeuterated methyl group in **30**.

Additionally, we detected **30′′**. No signals were seen corresponding to more than one deuterium atom in the methyl group. Deuteration was also seen in the aryl groups. This process will have exchanged original Ar–H hydrogen atoms for D atoms and correspondingly produced Si–H bonds in place of Si–D bonds, and these H atoms will have led to some unlabeled methyl groups seen in the product.

To check that this conversion of **92** to **30** was dependent on potassium ions,^10^ the reaction was repeated with Et_3_SiH + NaO^*t*^Bu; this gave no reaction.

**Scheme 11:**
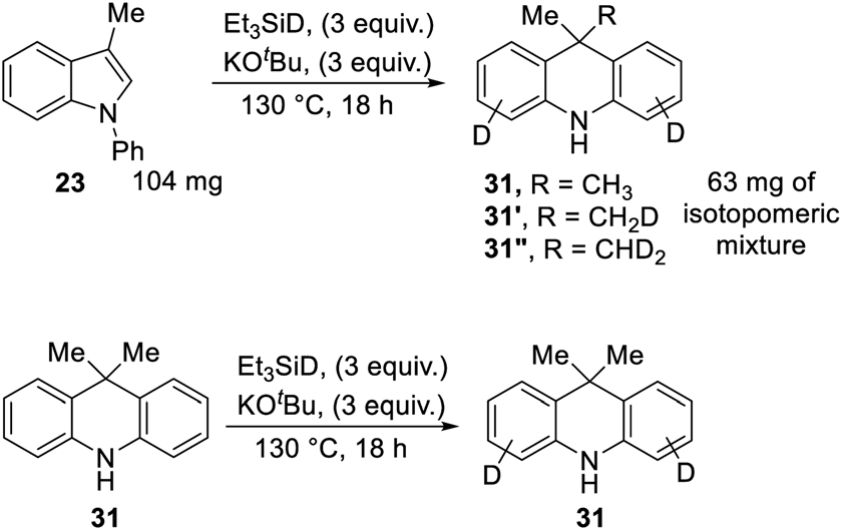


We also subjected 3-methyl-*N*-phenylindole **23** to reaction with Et_3_SiD/KO^*t*^Bu, focusing on deuterium incorporation into the methyl group of **31** (Scheme 11). In this case, we observed CH_3_, CH_2_D and CHD_2_ groups from the ^13^C{^1^H} and ^13^C{^1^H,^2^H} NMR spectra. This is in line with the proposed mechanism. The formation of some unlabelled methyl group and some monodeuterated CH_2_D group supports the exchange reactions mentioned above, where Ar–H hydrogen atoms on the substrate undergo exchange with R_3_Si–D bonds, and the resulting R_3_Si–H allows the formation of some unlabeled and monodeuterated methyl group in the dihydroacridine product. Consistent with the exchange reactions, deuteration of Ar–H positions was indeed seen in these reactions.

In a further experiment, unlabeled product **31** was subjected to the reaction conditions, to check whether the methyl groups were stable to the conditions or would undergo exchange. No exchange was seen in the methyl groups, although deuteration was seen on the aryl rings.

Additionally, and mindful of reports that H_2_ gas is produced on reaction of Et_3_SiH with KO^*t*^Bu,^2–7^ those two reagents were heated for 1 hour, and then the headspace in the vessel, containing hydrogen, was replaced by deuterium gas and substrate **23** was introduced. This led to formation of the dihydroacridine **31**. Examination of the ^2^H NMR spectrum showed deuterium incorporation into the methyl group of the product [and a much lesser degree of deuterium labelling of the Ar–H positions *ortho* to the NH group]. The methyl group carbon appeared as a singlet plus a 1:1:1 triplet, resulting from the presence of CH_3_ and CH_2_D (the combined yield of the products was 72%). Therefore, the gas in the headspace is not inert to the reaction. We further examined what happened when the hydrogen gas was removed and replaced by inert gas (argon). In this case, the transformation from indole substrate to dihydroacridine products was completely suppressed to < 1% yield.

As a final point, we investigated whether the reaction was specific to triethylsilane and to potassium *tert*-butoxide. Other silanes, notably Me_2_PhSiH, MePh_2_SiH and Ph_3_SiH were also successful in the conversion of **23** to **31**. However, upon replacement of KO^*t*^Bu with NaO^*t*^Bu (Table S1,† entry 5), no reaction took place.

## Conclusions

In summary, the Stoltz–Grubbs reducing system transforms *N*-arylindoles into 9,10-dihydroacridines in moderate to excellent yields. The ring-opening of the indole is triggered by fragmentation of intermediate indole radical anions; the styrenes formed in this fragmentation are then activated by potassium-ion-dependent hydrogen atom transfer to afford the dihydroacridine products. The transformation provides important information about the nature of chemistry that is undertaken by the Et_3_SiH + KO^*t*^Bu reagent.

## Conflicts of interest

There are no conflicts to declare.

## Supplementary Material

SC-011-D0SC00361A-s001
