# Supplementary material for: New reductive rearrangement of N-arylindoles triggered by the Grubbs–Stoltz reagent Et3SiH/KOtBu
Source: Chem Sci. 2020 Mar 11;11(14):3719–26. doi: 10.1039/d0sc00361a (PMC8152433; doi:10.1039/d0sc00361a)
Supplement: SC-011-D0SC00361A-s001 [file SC-011-D0SC00361A-s001.pdf]

Electronic Supplementary Material (ESI) for Chemical Science.
This journal is © The Royal Society of Chemistry 2020

Supplementary Information for:

New Reductive Rearrangement of N-Arylindoles Triggered by the Grubbs-Stoltz Reagent Et_3SiH/KOtBu

Andrew J. Smith, Daniela Dimitrova, Jude N. Arokianathar, Krystian Kolodziejczak, Allan Young, Mark Allison, Darren L. Poole, Stuart G. Leach, John A. Parkinson, Tell Tuttle and John A. Murphy

Table of Contents

Table of Contents S1

General Experimental S5

Computational Details S5

General Procedure A – Ullmann Coupling S6

General Procedure B – Treatment of Substrates under Et_3SiH/KO*t*Bu Conditions S6

General Procedure C – Fischer Indole Synthesis S6

Preparation of Substrates S7

Preparation of 1-Phenyl-1*H*-indole (**22**) S7

Preparation of 3-Methyl-1-phenyl-1*H*-indole (**23**) S7

Preparation of 3-Ethyl-1-phenyl-1*H*-indole (**24**) S8

Preparation of 2,3-Dimethyl-1-phenyl-1*H*-indole (**25**) S8

Preparation of 1,3-Diphenyl-1*H*-indole (**26**) S9

Preparation of 3-Octyl-1-phenyl-1*H*-indole (**27**) S10

Preparation of 3-Butyl-1-phenyl-1*H*-indole (**28**) S11

Preparation of 1-(*p*-Tolyl)-1*H*-indole (**29**) S11

Preparation of 3-Methyl-1-(naphthalen-1-yl)-1*H*-indole (**47**) S12

Preparation of 1-(Pyridin-3-yl)-1*H*-indole (**48**) S12

Preparation of 1-(Pyridin-2-yl)-1*H*-indole (**49**) S13

Preparation of 3-(But-3-en-1-yl)-1-phenyl-1*H*-indole (**50**) S14

Preparation of 3-(Pent-4-en-1-yl)-1-phenyl-1*H*-indole (**51**) S15

Preparation of 3-Cyclopropyl-1-phenyl-1*H*-indole (**61**) S16

Preparation of 2-Cyclopropyl-1-phenyl-1*H*-indole (**73**) S18

Preparation of *N*-Phenyl-2-vinylaniline (**92**) S20

Treating Substrates with Et_3SiH/KO*t*Bu S21

Treatment of 1-Phenyl-1*H*-indole (**22**) with Et_3SiH/KO*t*Bu S21

Treatment of 1-Phenyl-1*H*-indole (**22**) with Et_3SiH/NaO*t*Bu S21

Treatment of 1-Phenyl-1*H*-indole (**22**) with KO*t*Bu Alone S22

Treatment of 3-Methyl-1-phenyl-1*H*-indole (**23**) with Et_3SiH/KO*t*Bu S22

Treatment of 3-Ethyl-1-phenyl-1*H*-indole (**24**) with Et_3SiH/KO*t*Bu S22

Treatment of 2,3-Dimethyl-1-phenyl-1*H*-indole (**25**) with Et_3SiH/KO*t*Bu S23

Treatment of 1,3-Diphenyl-1*H*-indole (**26**) with Et_3SiH/KO*t*Bu S23

Treatment of 3-Octyl-1-phenyl-1*H*-indole (**27**) with Et_3SiH/KO*t*Bu S24

Treatment of 3-Butyl-1-phenyl-1*H*-indole (**28**) with Et_3SiH/KO*t*Bu S24

Treatment of 1-(*p*-Tolyl)-1*H*-indole (**29**) with Et_3SiH/KO*t*Bu S25

Treatment of 3-Methyl-1-(naphthalen-1-yl)-1*H*-indole (**47**) with Et_3SiH/KO*t*Bu S26

Treatment of 1-(Pyridin-3-yl)-1*H*-indole (**48**) with Et_3SiH/KO*t*Bu S26

Treatment of 1-(Pyridin-2-yl)-1*H*-indole (**49**) with Et_3SiH/KO*t*Bu S26

Treatment of 3-(But-3-en-1-yl)-1-phenyl-1*H*-indole (**50**) with Et_3SiH/KO*t*Bu S27

Treatment of 3-(But-3-en-1-yl)-1-phenyl-1*H*-indole (**50**) with KO*t*Bu Alone S27

Treatment of 3-(Pent-4-en-1-yl)-1-phenyl-1*H*-indole (**51**) with Et_3SiH/KO*t*Bu S29

Treatment of 3-(Pent-4-en-1-yl)-1-phenyl-1*H*-indole (**51**) with KO*t*Bu Alone S31

Treatment of 3-Cyclopropyl-1-phenyl-1*H*-indole (**61**) with Et_3SiH/KO*t*Bu S32

Treatment of 2-Cyclopropyl-1-phenyl-1*H*-indole (**73**) with Et_3SiH/KO*t*Bu S34

Treatment of *N*-Phenyl-2-vinylaniline (**92**) with Et_3SiH/KO*t*Bu (Fast Addition) S35

Treatment of *N*-Phenyl-2-vinylaniline (**92**) with Et_3SiH/KO*t*Bu (Slow Addition) S35

Treatment of *N*-Phenyl-2-vinylaniline (**92**) with Et_3SiH/NaOtBu (Slow Addition) S35

Treatment of 3-Methyl-1-phenyl-1*H*-indole (**23**) with 0.5 eq. Et_3SiH/ 0.5 eq. KO*t*Bu S36

Treatment of 3-Methyl-1-phenyl-1*H*-indole (**23**) with 0.5 eq. Et_3SiH/ 3.0 eq. KO*t*Bu S36

Further Mechanistic Studies S37

Treatment of 1-Phenyl-1*H*-indole (**22**) with **99**, $Me_3SiSiMe_3$ and KO^{t}Bu S37

Treatment of 1-Phenyl-1*H*-indole (**22**) with **99** and KO^{t}Bu S38

Treatment of 1-Phenyl-1*H*-indole (**22**) with $Me_3SiSiMe_3$ and KO^{t}Bu S38

Treatment of 1-Phenyl-3-methyl-1*H*-indole (**23**) with Et_3SiD/KO*t*Bu S39

Treatment of 9,9-Dimethyl-9,10-dihydroacridine (**31**) with Et_3SiD/KO*t*Bu S42

Treatment of 3-Methyl-1-phenyl-1*H*-indole (**23**) with Et_3SiH/KO*t*Bu with the Replacement of H_2 with D_2 .... S44

Treatment of 3-Methyl-1-phenyl-1*H*-indole (**23**) with Different Silanes S45

Treatment of 1,3-Diphenylindole (**26**) with K/KO*t*Bu S45

Treatment of *N*-phenyl-2-vinylaniline (**92**) with Et_3SiD/KO*t*Bu S46

References S47

NMR Spectra S50

^{1}H and ^{13}C NMR Spectra of 1-Phenyl-1*H*-indole (**22**) S50

^{1}H and ^{13}C NMR Spectra of 3-Methyl-1-phenyl-1*H*-indole (**23**) S51

^{1}H and ^{13}C NMR Spectra of 3-Ethyl-1-phenyl-1*H*-indole (**24**) S52

^{1}H and ^{13}C NMR Spectra of 2,3-Dimethyl-1-phenyl-1*H*-indole (**25**) S53

^{1}H and ^{13}C NMR Spectra of 3-Phenyl-1*H*-indole S54

^{1}H and ^{13}C NMR Spectra of 1,3-Diphenyl-1*H*-indole (**26**) S55

^{1}H and ^{13}C NMR Spectra of 3-Octyl-1*H*-indole S56

^{1}H and ^{13}C NMR Spectra of 3-Octyl-1-phenyl-1*H*-indole (**27**) S57

^{1}H and ^{13}C NMR Spectra of 3-Butyl-1*H*-indole S58

^{1}H and ^{13}C NMR Spectra of 3-Butyl-1-phenyl-1*H*-indole (**28**) S59

^{1}H and ^{13}C NMR Spectra of 1-(*p*-Tolyl)-1*H*-indole (**29**) S60

^{1}H and ^{13}C NMR Spectra of 3-Methyl-1-(naphthalen-1-yl)-1*H*-indole (**47**) S61

^{1}H and ^{13}C NMR Spectra of 1-(Pyridin-3-yl)-1*H*-indole (**48**) S62

^{1}H and ^{13}C NMR Spectra of 1-(Pyridin-2-yl)-1*H*-indole (**49**) S63

^{1}H and ^{13}C NMR Spectra of 3-(But-3-en-1-yl)-1-phenyl-1*H*-indole (**50**) S64

^{1}H and ^{13}C NMR Spectra of 3-(Pent-4-en-1-yl)-1-phenyl-1*H*-indole (**51**) S65

^{1}H and ^{13}C NMR Spectra of 3-(1-(Trimethylsilyl)cyclopropyl)-1H-indole S66

^{1}H and ^{13}C NMR Spectra of 3-Cyclopropyl -1*H*-indole S67

^{1}H and ^{13}C NMR Spectra of 3-Cyclopropyl-1-phenyl-1*H*-indole (**61**) S68

^{1}H and ^{13}C NMR Spectra of 2-(Cyclopropylethynyl)aniline S69

^{1}H and ^{13}C NMR Spectra of 2-Cyclopropyl-1*H*-indole S70

^{1}H and ^{13}C NMR Spectra of 2-Cyclopropyl-1-phenyl-1*H*-indole (**73**) S71

^{1}H and ^{13}C NMR Spectra of (2-(Phenylamino)phenyl)methanol S72

^{1}H and ^{13}C NMR Spectra of 2-(Phenylamino)benzaldehyde S73

^{1}H and ^{13}C NMR Spectra of *N*-Phenyl-2-vinylaniline (**92**) S74

^{1}H and ^{13}C NMR Spectra of 9-Methyl-9,10-dihydroacridine (**30**) S75

^{1}H and ^{13}C NMR Spectra of 9,9-Dimethyl-9,10-dihydroacridine (**31**) S76

^{1}H and ^{13}C NMR Spectra of 9-Ethyl-9-methyl-9,10-dihydroacridine (**32**) S77

^{1}H and ^{13}C NMR Spectra of 9-Methyl-9-phenyl-9,10-dihydroacridine (**33**) S78

^{1}H and ^{13}C NMR Spectra of 9-Methyl-9-octyl-9,10-dihydroacridine (**34**) S79

^{1}H and ^{13}C NMR Spectra of 9-Butyl-9-methyl-9,10-dihydroacridine (**35**) S80

^{1}H and ^{13}C NMR Spectra of 2,9-Dimethyl-9,10-dihydroacridine (**36**) S81

^{1}H and ^{13}C NMR Spectra of (1*R**,2*R**)-2-Methyl-1'-phenylspiro[cyclopentane-1,3'-indoline] (**52**) S82

COSY NMR Spectra of (1*R**,2*R**)-2-Methyl-1'-phenylspiro[cyclopentane-1,3'-indoline] (**52**) S83

HSQC NMR Spectra of (1*R**,2*R**)-2-Methyl-1'-phenylspiro[cyclopentane-1,3'-indoline] (**52**) S84

HMBC NMR Spectra of (1*R**,2*R**)-2-Methyl-1'-phenylspiro[cyclopentane-1,3'-indoline] (**52**) S85

^{1}H and ^{13}C NMR Spectra of (1*R**,2*S**)-2-Methyl-1'-phenylspiro[cyclopentane-1,3'-indoline] (**53**)....................S86

COSY NMR Spectrum of (1*R**,2*S**)-2-Methyl-1'-phenylspiro[cyclopentane-1,3'-indoline] (**53**) S87

HSQC NMR Spectrum of (1*R**,2*S**)-2-Methyl-1'-phenylspiro[cyclopentane-1,3'-indoline] (**53**) S88

HMBC NMR Spectrum of (1*R**,2*S**)-2-Methyl-1'-phenylspiro[cyclopentane-1,3'-indoline] (**53**) S89

^{1}H and ^{13}C NMR Spectra of the Mixture of 9-Cyclopropyl-9-methyl-9,10-dihydroacridine (**62**) and 9-Methyl-9-propyl-9,10-dihydroacridine (**63**)....................S90

^{1}H and ^{13}C NMR Spectra of Acridin-9-yl(cyclopropyl)methanone (**76**) S91

^{1}H and ^{13}C NMR Spectra of 9-Methylacridine (**96**) S92

^{1}H and ^{13}C NMR Spectra of Indole (**100**)....................S93

^{1}H and ^{13}C NMR Spectra of 9-Phenylacridine (**101**)....................S94

^{1}H, ^{2}D, and ^{13}C NMR Spectra of the Mixture of 9-Methyl-9,10-dihydroacridine (**30**), 9-(Methyl-*d*)-9,10-dihydroacridine (**30'**), and 9-Methyl-9,10-dihydroacridine-9-*d* (**30''**) S95

^{1}H, ^{2}D, and ^{13}C NMR Spectra of the Mixture of 9,9-Dimethyl-9,10-dihydroacridine (**31**) and 9-Methyl-9-(methyl-*d*)-9,10-dihydroacridine (**31'**) S96

Computational Analysis of radical anions from Figure 1....................S98

Computational Analysis of the conversion of **43** to **44**....................S104

General Experimental

All reagents and solvents were obtained from commercial suppliers and were used without further purification. The glovebox was supplied by Innovative Technology Inc., USA, and the atmosphere used is nitrogen. DMF, DMSO and Et_3N were dried over 3 Å molecular sieves (10 % w/v) [which were activated by microwave heating (3 x 5 min)] and degassed by bubbling argon through the solvent for 30 min. The solvents were then left to dry for 24 h before use. THF was dried using a Pure-Solv 400 solvent purification system (Innovative Technology Inc., USA). Sodium hydride was supplied as a 60 % dispersion in mineral oil and was washed with hexane to remove this oil before use, unless otherwise specified. Thin layer chromatography was carried out using Merck silica plates coated with fluorescent indicator UV254. These were analysed under 254 nm UV light. Flash chromatography was carried out using ZEOprep 60 HYD 40-63 µm silica gel. Fourier Transform Infra-Red (FTIR) spectra were obtained on a Shimadzu IRAffinity-1 instrument. ^{1}H and ^{13}C NMR spectra were obtained on a Bruker DPX 400 spectrometer at 400 and 101 MHz, respectively. Chemical shifts are reported in ppm and coupling constants are reported in Hz, accurate to 0.3 Hz, with $CDCl_3$ referenced at 7.27 (^{1}H) and 77.00 ppm (^{13}C). The following abbreviations are used for the multiplicities: s, singlet; d, doublet; t, triplet; q, quartet; quin, quintet; sxt, sextet; m, multiplet; br, broad. High resolution mass spectrometry (HRMS) was performed at the University of Swansea in the EPSRC National Mass Spectrometry Centre. Accurate mass was obtained using LTQ Orbitrap XL using Atmospheric Pressure Chemical Ionisation (APCI) or High Resolution Nano-Electrospray (HNESP) using Electrospray Ionisation (ESI). GC-MS spectra were obtained on a Thermo Finnigan Polaris Q, mass range 50-650 Da. The column temperature was 320 °C, and the carrier gas was helium with a flow rate of 1 mL/min. The adsorbent was Crossbond® (0.25 µm) with column dimensions of 30 m x 0.25 mm. Results are reported as *m/z*. All samples were prepared in $CHCl_3$ and electron ionisation (EI) was used as the ionisation method.

Computational Details

All geometry optimizations and frequency calculations were performed in Gaussian09,[1] with the M06-2X functional[2-3] and the 6-31++G(d,p) basis set.[4-6] The effects of solvation were modelled implicitly using the conductor-like polarisable continuum model (CPCM),[7-8] with triethylamine (ε=2.3832) as solvent (unless otherwise specified). Since triethylsilane is not defined in Gaussian09, triethylamine was deemed the most suitable alternative, with a dielectric constant similar to that of triethylsilane (ε=2.323). All calculations were carried out at 298.15 K.

General Procedure A – Ullmann Coupling

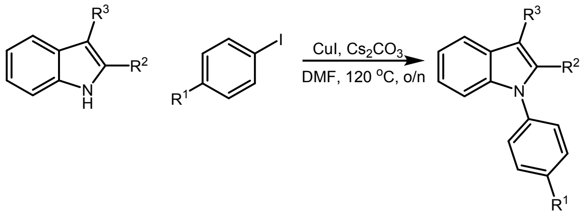

These reactions were carried out according to a literature procedure.[9] To a three-necked flask, under argon, was added the appropriate heterocycle (1.1 or 1.4 equiv.), appropriate aryl iodide (1 equiv.), copper(I) iodide (0.2 equiv.), cesium carbonate (2 equiv.) and dry DMF (0.5 M). The resulting mixture was stirred overnight at 120 °C. After cooling to room temperature, the reaction mixture was diluted with EtOAc and washed with water. The organic layer was then dried over Na_2SO_4, filtered and concentrated under reduced pressure. Purification by column chromatography afforded the relevant *N*-arylindole.

General Procedure B – Treatment of Substrates under Et_3SiH/KO*t*Bu Conditions

Substrate (0.5 mmol, 1 equiv., unless otherwise specified), triethylsilane (0.24 mL, 1.5 mmol, 3 equiv., unless otherwise specified) and potassium *tert*-butoxide (168 mg, 1.5 mmol, 3 equiv., unless otherwise specified) were sealed in a pressure tube in a glovebox under nitrogen. The tube was removed and heated at 130 °C for 18 h behind a safety shield. After cooling to room temperature, the mixture was diluted with water (50 mL) and extracted into diethyl ether (3 x 50 mL). The combined organic layers were dried over Na_2SO_4, filtered and concentrated under reduced pressure.

General Procedure C – Fischer Indole Synthesis

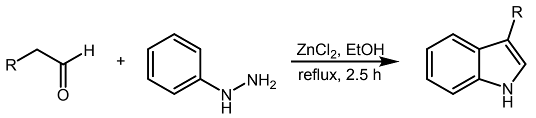

These reactions were carried out according to a literature procedure.[10] To a three-necked flask under argon and equipped with a stirrer bar and fitted with a condenser was added the appropriate aldehyde (10 mmol, 1 equiv.) and phenylhydrazine (0.98 mL, 10 mmol, 1 equiv.). This mixture was stirred for 1 h at room temperature then for 30 min at 100 °C. Zinc chloride (2.45 g, 18 mmol, 1.8 equiv.) in ethanol (11 mL) was then added and the mixture was heated under reflux for a further 1 h. After cooling to room temperature, the mixture was filtered, and the solvent was removed under reduced pressure. Hydrochloric acid (2 M) was added to the crude residue and the organic products were extracted into DCM. The organic layers were dried over Na_2SO_4, filtered and concentrated under reduced pressure. Purification by column chromatography and/or recrystallisation afforded the appropriate indole.

Preparation of Substrates

Preparation of 1-Phenyl-1*H*-indole (22)

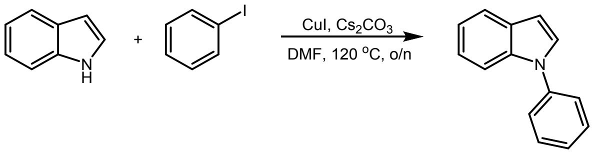

22, 89 %

This substrate was prepared according to General Procedure A from 1*H*-indole (1.64 g, 14 mmol, 1.4 equiv.) and iodobenzene (1.12 mL, 10 mmol, 1 equiv.) with copper (I) iodide (381 mg, 2 mmol, 0.2 equiv.) and cesium carbonate (6.52 g, 20 mmol, 2 equiv.) in DMF (20 mL). Purification by column chromatography (hexane) afforded 1-phenyl-1*H*-indole **22** as a colourless oil (1.72 g, 89 %). **^{1}H-NMR** (400 MHz, $CDCl_3$) 6.71 (dd, *J* = 3.3, 0.8 Hz, 1 H, ArH), 7.15 - 7.22 (m, 1 H, ArH), 7.22 - 7.27 (m, 1 H, ArH), 7.34 - 7.43 (m, 2 H, 2 x ArH), 7.50 - 7.56 (m, 4 H, 4 x ArH), 7.57 - 7.63 (m, 1 H, ArH), 7.69 - 7.74 (m, 1 H, ArH). **^{13}C-NMR** (101 MHz, $CDCl_3$) 103.5, 110.5, 120.3, 121.1, 122.3, 124.3, 126.4, 127.9, 129.2, 129.6, 135.8, 139.8. **ATR-IR** v_{max} (neat)/cm^{-1} 3051, 1595, 1512, 1494, 1454, 1329, 1230, 1211, 1134, 1072, 1012, 950, 906, 881, 781. ***m/z* (EI)** 193.1 (M^+, 100), 165.1 (28), 89.1 (17), 77.1 (7), 63.1 (7), 51.1 (12). The data for this compound are consistent with those reported in the literature.[11]

Preparation of 3-Methyl-1-phenyl-1*H*-indole (23)

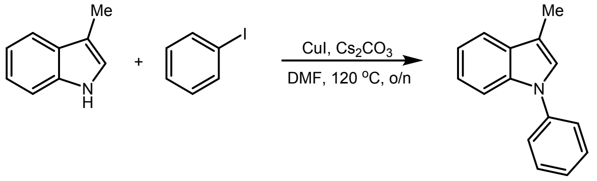

23, 62 %

This substrate was prepared according to General Procedure A from 3-methyl-1*H*-indole (367 mg, 2.8 mmol, 1.4 equiv.) and iodobenzene (0.22 mL, 2 mmol, 1 equiv.) with copper(I) iodide (76 mg, 0.4 mmol, 0.2 equiv.) and cesium carbonate (1.30 g, 4 mmol, 2 equiv.) in DMF (4 mL). Purification by column chromatography (hexane) afforded 3-methyl-1-phenyl-1*H*-indole **23** as a yellow oil (257 mg, 62 %). **^{1}H-NMR** (400 MHz, $CDCl_3$) 2.49 (d, *J* = 1.3 Hz, 3 H, $ArCH_3$), 7.22 (q, *J* = 1.0 Hz, 1 H, ArH), 7.28 (td, *J* = 7.5, 1.3 Hz, 1 H, ArH), 7.32 (td, *J* = 8.0, 1.5 Hz, 1 H, ArH), 7.36 - 7.43 (m, 1 H, ArH), 7.57 (m, 4 H, 4 x ArH), 7.66 (dt, *J* = 8.2, 0.9 Hz, 1 H, ArH), 7.71 - 7.76 (m, 1 H, ArH). **^{13}C-NMR** (101 MHz, $CDCl_3$) 9.6, 110.3, 112.8, 119.2, 119.8, 122.3, 124.0, 125.4, 125.9, 129.5, 129.8, 135.9, 140.0. **ATR-IR** v_{max} (neat)/cm^{-1} 3045, 2912, 1597, 1499, 1454, 1371, 1227, 1125, 1070, 1015, 904, 773, 692, 655. ***m/z* (EI)** 207.1 (M^+, 92), 206.1 (100), 178.1 (8), 128.1 (18), 102.1 (9), 77.1 (44), 63.1 (6), 51.1 (24). The data for this compound are consistent with those reported in the literature.[12]

Preparation of 3-Ethyl-1-phenyl-1*H*-indole (24)

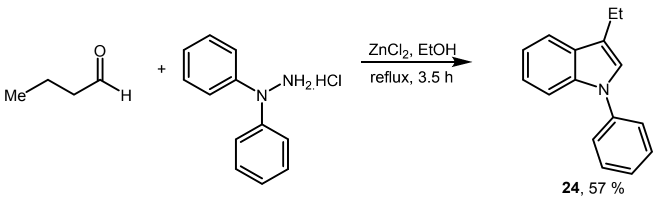

24, 57 %

This substrate was prepared according to a modified literature procedure.[10] A mixture of butyraldehyde (0.45 mL, 5 mmol, 1 equiv.) and 1,1-diphenylhydrazine hydrochloride (1.1 g, 5 mmol, 1 equiv.) was stirred for 1 h under argon. The reaction mixture was then heated to 100 °C and heated for a further 30 min at this temperature. A solution of zinc chloride (1.23 g, 9 mmol, 1.9 equiv.) in ethanol (5.5 mL) was then added and the mixture stirred at reflux for 2 h. After cooling to room temperature, the mixture was filtered, and the solvent was removed under reduced pressure. 1 M HCl was added to the residue and organic material was extracted into DCM. The combined organic layers were then dried over Na_2SO_4, filtered and concentrated under reduced pressure. Purification by column chromatography (hexane:diethyl ether, 49:1) afforded 3-ethyl-1-phenyl-1*H*-indole **24** as a yellow oil (627 mg, 57 %). **^{1}H-NMR** (400 MHz, $CDCl_3$) 1.41 (t, *J* = 7.5 Hz, 3 H, CH_3), 2.88 (qd, *J* = 7.5, 1.0 Hz, 2 H, CH_2), 7.15 - 7.17 (m, 1 H, ArH), 7.17 - 7.22 (m, 1 H, ArH), 7.22 - 7.27 (m, 1 H, ArH), 7.30 - 7.38 (m, 1 H, ArH), 7.48 - 7.55 (m, 4 H, 4 x ArH), 7.56 - 7.62 (m, 1 H, ArH), 7.65 - 7.72 (m, 1 H, ArH). **^{13}C-NMR** (101 MHz, $CDCl_3$) 14.3, 18.3, 110.5, 119.3, 119.7, 119.8, 122.3, 124.0, 124.4, 125.9, 129.0, 129.5, 136.1, 140.1. **ATR-IR** ν_{max} (neat)/cm^{-1} 3049, 2960, 1595, 1499, 1454, 1377, 1315, 1298, 1223, 1132, 1072, 1014, 960, 925, 904, 773, 694. ***m/z* (EI)** 221.1 (M^+, 47) 206.1 (100), 178.1 (9), 128.0 (14), 115.1 (12), 102.1 (10), 89.0 (5), 77.1 (40), 63.0 (4), 51.1 (19). The data for this compound are consistent with those reported in the literature.[12]

Preparation of 2,3-Dimethyl-1-phenyl-1*H*-indole (25)

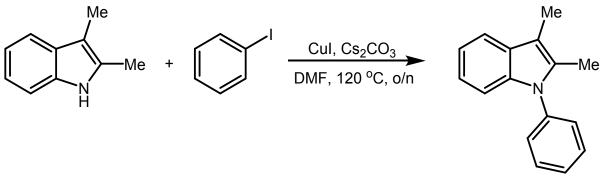

25, 47 %

This substrate was prepared according to General Procedure A from 2,3-dimethyl-1*H*-indole (290 mg, 2.8 mmol, 1.4 equiv.) and iodobenzene (0.22 mL, 2 mmol, 1 equiv.) with copper (I) iodide (76 mg, 0.4 mmol, 0.2 equiv.) and cesium carbonate (1.30 g, 4 mmol, 2 equiv.) in DMF (4 mL). Purification by column chromatography (hexane) afforded 2,3-dimethyl-1-phenyl-1*H*-indole **25** as a colourless oil (207 mg, 47 %). **^{1}H-NMR** (400 MHz, $CDCl_3$) 2.26 (s, 3 H, $ArCH_3$), 2.35 (d, *J* = 0.5 Hz, 3 H, $ArCH_3$), 7.08 - 7.18 (m, 3 H, 3 x ArH), 7.32 - 7.38 (m, 2 H, 2 x ArH), 7.45 (tt, *J* = 7.5, 2.0 Hz, 1 H, ArH), 7.50 - 7.59 (m, 3 H, 3 x ArH). **^{13}C-NMR** (101 MHz, $CDCl_3$) 8.9, 11.0, 107.9, 109.7, 117.8, 119.4, 121.1, 127.4, 128.0, 128.7, 129.3, 132.8, 137.2, 138.3. **ATR-IR** ν_{max} (neat)/cm^{-1} 3041, 2915, 2858, 1597, 1499, 1458, 1400, 1361, 1234, 1217, 1134,

1072, 1015, 914. ***m/z* (EI)** 221.1 (M^+, 100), 206.1 (38), 178.1 (6), 143.1 (9), 128.1 (9), 115.0 (15), 102.1 (12), 77.0 (57), 63.0 (8), 51.0 (34). The data for this compound are consistent with those reported in the literature.[13]

Preparation of 1,3-Diphenyl-1*H*-indole (26)

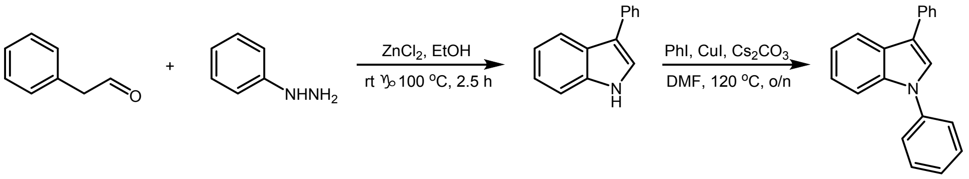

26, 65 %

The first step was carried out according to a literature procedure.[10] A mixture of phenylacetaldehyde (1.17 mL, 10 mmol, 1 equiv.) and phenylhydrazine (0.98 mL, 10 mmol, 1 equiv.) was stirred at room temperature for 1 h under argon. The mixture was then heated to 100 °C and stirred for 30 min. A solution of zinc chloride (2.45 g, 18 mmol, 1.8 equiv.) in ethanol (11 mL) was added to the reaction mixture and this was refluxed at 100 °C for 1 h. After cooling to room temperature, the mixture was filtered, and the solvent was removed under reduced pressure. 2 M HCl was added to the crude residue and the organic products were extracted into DCM. The organic layers were dried over Na_2SO_4, filtered and concentrated under reduced pressure. Purification by column chromatography (hexane:diethyl ether, 3:1) afforded 3-phenyl-1*H*-indole as an orange solid (1.34 g, 69%). **Mp** = 81-83 °C (lit. mp = 83-85 °C).[14] **^{1}H-NMR** (400 MHz, $CDCl_3$) 7.15 - 7.38 (m, 4H, 4 x ArH), 7.41 (d, *J* = 7.9 Hz, 1H, ArH), 7.50 (t, *J* = 7.6 Hz, 2H, 2 x ArH), 7.72 (d, *J* = 7.5 Hz, 2H, 2 x ArH), 8.01 (d, *J* = 7.9 Hz, 1H, ArH), 8.08 (br. s., 1H, NH). **^{13}C-NMR** (101 MHz, $CDCl_3$) 111.4, 118.2, 119.8, 120.3, 121.8, 122.4, 125.7, 126.0, 127.4, 128.7, 135.5, 136.6. **ATR-IR** v_{max} (neat)/cm^{-1} 3400, 1597, 1541, 1456, 1417, 1338, 1259, 1236, 1112, 1101, 1010, 823, 769, 694, 632. **m/z (EI)** 193.2 (M^+, 100), 165.1 (37). The data for this compound are consistent with those reported in the literature.[15]

The second step was carried out according to General Procedure A from 3-phenyl-1*H*-indole (1.06 g, 5.5 mmol, 1.1 equiv.), and iodobenzene (0.56 mL, 5 mmol, 1 equiv.), with copper (I) iodide (190 mg, 1 mmol, 0.2 equiv.), and cesium carbonate (3.26 g, 10 mmol, 2 equiv.) in DMF (10 mL). Purification by column chromatography (hexane:diethyl ether, 49:1) afforded 1,3-diphenyl-1*H*-indole **26** as a white solid (878 mg, 65 %). **Mp** = 105-108 °C (lit. mp = 107-108.5 °C).[16] **^{1}H-NMR** (400 MHz, $CDCl_3$) 7.25 - 7.33 (m, 2 H, 2 x ArH), 7.33 - 7.39 (m, 1 H, ArH), 7.39 - 7.45 (m, 1 H, ArH), 7.47 - 7.53 (m, 2 H, 2 x ArH), 7.53 - 7.56 (m, 1 H, ArH), 7.56 - 7.62 (m, 4 H, 4 x ArH), 7.62 - 7.67 (m, 1 H, ArH), 7.72 - 7.78 (m, 2 H, 2 x ArH), 8.01 - 8.05 (m, 1 H, ArH). **^{13}C-NMR** (101 MHz, $CDCl_3$) 110.8, 119.1, 120.1, 120.9, 122.8, 124.5, 125.5, 126.2, 126.7, 127.1, 127.6, 128.8, 129.7, 135.1, 136.7, 139.5. **ATR-IR** v_{max} (neat)/cm^{-1} 3018, 1593, 1552, 1500, 1454, 1379, 1226, 1076, 1022, 972, 908, 810, 734, 639. ***m/z* (EI)** 269.2 (M^+,

94), 190.1 (14), 165.1 (86), 139.1 (11), 77.1 (100), 51.1 (53). The data for this compound are consistent with those reported in the literature.[14]

Preparation of 3-Octyl-1-phenyl-1*H*-indole (27)

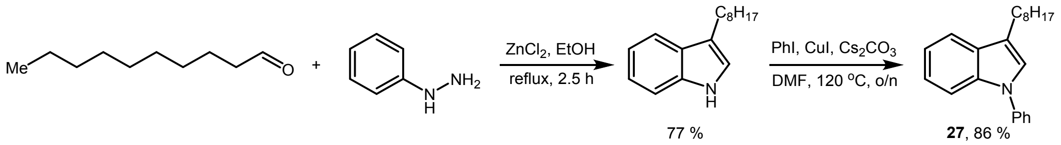

Step 1 was carried out according to General Procedure C from decanal (1.88 mL, 10 mmol, 1 equiv.). Purification by column chromatography (hexane:diethyl ether, 9:1) afforded 3-octyl-1-phenyl-1*H*-indole as orange crystals (1.76g, 77 %). **Mp** = 30-32 °C (lit. mp = 32 °C).[17] **^{1}H-NMR** (400 MHz, $CDCl_3$) 0.90 (t, *J* = 6.8 Hz, 3 H, CH_3), 1.16 - 1.46 (m, 10 H, 5 x CH_2), 1.73 (quin, *J* = 7.5 Hz, 2 H, CH_2), 2.76 (td, *J* = 7.3, 0.8 Hz, 2 H, CH_2), 6.96 - 7.01 (m, 1 H, ArH), 7.12 (ddd, *J* = 7.85, 7.0, 1.0 Hz, 1 H, ArH), 7.20 (ddd, *J* = 8.3, 7.0, 1.3 Hz, 1 H, ArH), 7.36 (dt, *J* = 8.0, 0.9 Hz, 1 H, ArH), 7.56 - 7.69 (m, 1 H, ArH), 7.89 (br. s., 1 H, NH). **^{13}C-NMR** (101 MHz, $CDCl_3$) 14.1, 22.7, 25.1, 29.3, 29.5, 29.7, 30.2, 31.9, 111.0, 117.1, 119.0 (2 carbons), 121.0, 121.8, 127.6, 136.3. **ATR-IR** ν_{max} (neat)/cm^{-1} 3419, 3001, 2922, 1595, 1496, 1454, 1309, 1220, 1134, 1074, 1020, 871, 773, 694. ***m/z* (EI)** 229.3 (M^+, 9), 143.1 (5), 130.1 (100), 103.1 (7), 77.7 (7). The data for this compound are consistent with those reported in the literature.[17]

Step 2 was carried out according to General Procedure A from 3-octyl-1-phenyl-1*H*-indole (1.26 g, 5.5 mmol, 1,1 equiv.) and, iodobenzene (0.56 mL, 5 mmol, 1 equiv.), copper (I) iodide (190 mg, 1 mmol, 0.2 equiv.), and cesium carbonate (3.26 g, 10 mmol, 2 equiv.) in DMF (10 mL). Purification by column chromatography (hexane) afforded 3-octyl-1-phenyl-1*H*-indole **27** as an orange oil (1.32 g, 86 %). **^{1}H-NMR** (400 MHz, $CDCl_3$) 0.91 (t, *J* = 6.8 Hz, 3 H, CH_3), 1.22 - 1.52 (m, 10 H, 5 x CH_2), 1.78 (quin, *J* = 7.5 Hz, 2 H, CH_2), 2.82 (t, *J* = 7.5 Hz, 2 H, CH_2), 7.14 - 7.17 (m, 1 H, ArH), 7.19 (dd, *J* = 7.5, 1.3 Hz, 1 H, ArH), 7.24 (td, *J* = 7.0, 1.3 Hz, 1 H, ArH), 7.33 (m, 1 H, ArH), 7.52 (m, 4 H, 4 x ArH), 7.59 (d, *J* = 8.0 Hz, 1 H, ArH), 7.68 (d, *J* = 7.5 Hz, 1 H, ArH). **^{13}C-NMR** (101 MHz, $CDCl_3$) 14.1, 22.7, 25.1, 29.3, 29.5, 29.7, 30.1, 31.9, 110.4, 118.3, 119.3, 119.7, 122.3, 124.0, 124.9, 125.9, 129.2, 129.5, 136.0, 140.1. **ATR-IR** ν_{max} (neat)/cm^{-1} 2920, 2850, 1595, 1498, 1454, 1377, 1317, 1224, 1132, 773, 694. ***m/z* (EI)** 305.3 (M^+, 33), 206.2 (100), 178.1 (18), 128.1 (20), 77.1 (14), 57.1 (7). **HRMS (CI)** calcd. for $C_{22}H_{28}N^+$ ($[M+H]^+$): 306.2216, found: 306.2213.

Preparation of 3-Butyl-1-phenyl-1*H*-indole (28)

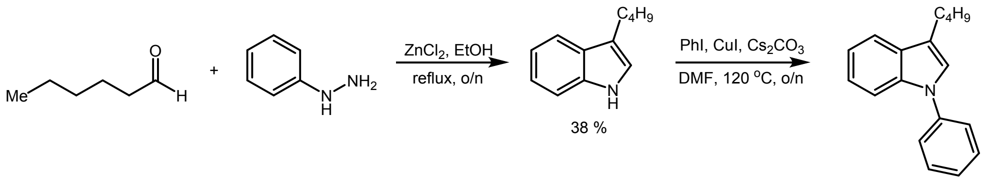

28, 83 %

Step 1 was carried out according to General Procedure C from hexanal (1.23 mL, 10 mmol, 1 equiv.). Purification by column chromatography (hexane:diethyl ether, 19:1 ♑ 9:1) afforded 3-butyl-1*H*-indole as an orange oil (665 mg, 38 %). **^{1}H-NMR** (400 MHz, $CDCl_3$) 0.97 (t, *J* = 7.3 Hz, 3 H, CH_3), 1.44 (sxt, *J* = 7.3 Hz, 2 H, CH_2), 1.58 - 1.81 (m, 2 H, CH_2), 2.57 - 2.87 (m, 2 H, CH_2), 6.96 - 7.00 (m, 1 H, ArH), 7.09 - 7.16 (m, 1 H, ArH), 7.20 (td, *J* = 7.5, 1.5 Hz, 1 H, ArH), 7.36 (dt, *J* = 8.1, 1.0 Hz, 1 H, ArH), 7.60 - 7.65 (m, 1 H, ArH), 7.88 (br. s., 1 H, NH). **^{13}C-NMR** (101 MHz, $CDCl_3$) 14.0, 22.7, 24.8, 32.3, 111.0, 117.2, 119.0 (2 carbons), 121.0, 121.8, 127.7, 136.3. **ATR-IR** ν_{max} (neat)/cm^{-1} 3414, 2954, 2926, 2845, 1618, 1454, 1419, 1338, 1222, 1089, 1008, 802, 763. ***m/z* (EI)** 173.2 (M^+, 71), 143.1 (21), 130.2 (100), 115.1 (26), 103.1 (42), 89.1 (15), 77.1 (51), 63.1 (11), 51.1 (11). The data for this compound are consistent with those reported in the literature.[17]

Step 2 was carried out according to General Procedure A from 3-butyl-1*H*-indole (665 mg, 3.83 mmol, 1.1 equiv.), and iodobenzene (0.39 mL, 3.45 mmol, 1 equiv.), with copper (I) iodide (131 mg, 0.69 mmol, 0.2 equiv.), and cesium carbonate (2.25 g, 6.9 mmol, 2 equiv.) in DMF (7 mL). Purification by column chromatography (hexane) afforded 3-butyl-1-phenyl-1*H*-indole **28** as an off-white solid (715 mg, 83 %). **Mp** = 44-45 °C. **^{1}H-NMR** (400 MHz, $CDCl_3$) 0.99 (t, *J* = 7.4 Hz, 3 H, CH_3), 1.48 (sxt, *J* = 7.5 Hz, 2 H, CH_2), 1.70 - 1.84 (m, 2 H, CH_2), 2.76 - 2.88 (m, 2 H, CH_2), 7.13 - 7.20 (m, 2 H, 2 x ArH), 7.20 - 7.26 (m, 1 H, ArH), 7.33 (m, 1 H, ArH), 7.51 (m, 4 H, 4 x ArH), 7.55 - 7.61 (m, 1 H, ArH), 7.67 (dt, *J* = 8.1, 0.8 Hz, 1 H, ArH). **^{13}C-NMR** (101 MHz, $CDCl_3$) 14.0, 22.7, 24.7, 32.2, 110.4, 118.2, 119.3, 119.7, 122.3, 124.0, 124.9, 125.9, 129.2, 129.5, 136.0, 140.0. **ATR-IR** ν_{max} (neat)/cm^{-1} 3053, 2956, 2870, 1593, 1500, 1454, 1373, 1311, 1213, 1132, 1074, 1016, 898, 773, 686, 669. ***m/z* (EI)** 249.2 (M^+, 20), 206.2 (100), 178.2 (11), 128.1 (19), 115.0 (8), 102.3 (7), 89.0 (5), 77.1 (30), 51.0 (8). **HRMS (CI)** calcd. for $C_{18}H_{20}N^+$ ($[M+H]^+$): 250.1596, found: 250.1595.

Preparation of 1-(*p*-Tolyl)-1*H*-indole (29)

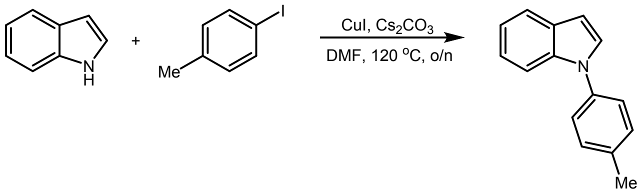

29, 55 %

This substrate was prepared according to General Procedure A from 1*H*-indole (234 mg, 2.8 mmol, 1.4 equiv.) and 4-iodotoluene (436 mg, 2 mmol, 1 equiv.) with copper (I) iodide

(76 mg, 0.4 mmol, 0.2 equiv.) and cesium carbonate (1.30 g, 4 mmol, 2 equiv.) in DMF (4 mL). Purification by column chromatography (hexane) afforded 1-(*p*-tolyl)-1*H*-indole **29** as a yellow oil (226 mg, 55 %). **^{1}H-NMR** (400 MHz, $CDCl_3$) 2.46 (s, 3 H, $ArCH_3$), 6.70 (dd, *J* = 3.3, 0.8 Hz, 1 H, ArH), 7.16 - 7.21 (m, 1 H, ArH), 7.22 - 7.27 (m, 1 H, ArH), 7.31 - 7.37 (m, 3 H, 3 x ArH), 7.39 - 7.44 (m, 2 H, 2 x ArH), 7.53 - 7.59 (m, 1 H, ArH), 7.69 - 7.75 (m, 1 H, ArH). **^{13}C-NMR** (101 MHz, $CDCl_3$) 21.0, 103.2, 110.5, 120.2, 121.0, 122.2, 124.3, 128.0, 129.2, 130.1, 136.0, 136.3, 137.3. **ATR-IR** ν_{max} (neat)/cm^{-1} 3030, 2916, 1606, 1514, 1454, 1330, 1315, 1230, 1211, 1134, 1107, 1012, 952, 881, 817, 710. ***m/z* (EI)** 207.1 (M^+,100), 191.1 (7), 178.1 (8), 165.1 (11), 116.1 (10), 102.5 (8), 89.0 (29), 77.0 (5), 63.0 (15), 51.0 (7). The data for this compound are consistent with those reported in the literature.[9]

Preparation of 3-Methyl-1-(naphthalen-1-yl)-1*H*-indole (**47**)

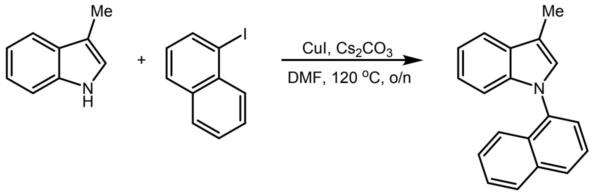

47, 89 %

This substrate was prepared according to General Procedure A from 3-methyl-1*H*-indole (918 mg, 7 mmol, 1.4 equiv.) and 1-iodonaphthalene (0.73 mL, 5 mmol, 1 equiv.) with copper (I) iodide (190 mg, 1 mmol, 0.2 equiv.) and cesium carbonate (3.26 g, 10 mmol, 2 equiv.) in DMF (10 mL). Purification by column chromatography (hexane:diethyl ether, 19:1) afforded 3-methyl-1-(naphthalen-1-yl)-1*H*-indole **47** as an orange solid (1.14 g, 89 %). **Mp** = 93-95 °C. **^{1}H-NMR** (400 MHz, $CDCl_3$) 2.47 (d, *J* = 1.0 Hz, 3 H, $ArCH_3$), 6.98 - 7.05 (m, 1 H, ArH), 7.09 - 7.23 (m, 3 H, 3 x ArH), 7.42 (td, *J* = 7.3, 1.3 Hz, 1 H, ArH), 7.49 - 7.62 (m, 4 H, 4 x ArH), 7.67 - 7.74 (m, 1 H, ArH), 7.90 - 8.01 (m, 2 H, 2 x ArH). **^{13}C-NMR** (101 MHz, $CDCl_3$) 9.7, 110.7, 112.0, 119.0, 119.5, 122.1, 123.5, 124.9, 125.5, 126.5, 126.7, 127.4, 128.0, 128.2, 128.9, 130.5, 134.4, 136.2, 138.2. **ATR-IR** ν_{max} (neat)/cm^{-1} 3043, 2912, 1595, 1573, 1506, 1469, 1452, 1406, 1303, 1232, 1008, 862, 800, 761, 686, 642. ***m/z* (EI)** 257.2 (M^+, 100), 241.2 (33), 127.1 (34), 102.1 (15), 77.1 (35), 63.1 (11), 51.1 (13). **HRMS (CI)** calcd. for $C_{19}H_{16}N^+$ ($[M+H]^+$): 258.1283, found: 258.1282.

Preparation of 1-(Pyridin-3-yl)-1*H*-indole (**48**)

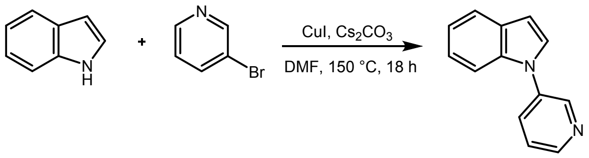

48, 33%

This substrate was prepared according to a literature procedure.[18] Indole (820 mg, 7 mmol, 1.4 equiv.), copper iodide (190 mg, 1 mmol, 0.2 equiv.) and cesium carbonate (3.26 g, 10 mmol,

2 equiv.) were added to a three-necked round-bottom flask under argon. 3-Bromopyridine (482 µL, 5 mmol, 1 equiv.) and dry DMF (10 mL) were also added and the contents of the flask were stirred at 120 °C for 16 h. Copper iodide (95 mg, 0.5 mmol, 0.1 equiv.) was further added and the reaction was stirred at 150 °C for 17.5 h. The reaction was then cooled to room temperature and diluted with EtOAc and sat. LiCl solution. The sat. LiCl layer was separated and washed with EtOAc multiple times until an aqueous layer of light turquoise colour was obtained. The organic phases were combined and concentrated under reduced pressure. Purification by column chromatography (hexane:ethyl acetate, 95:5 ♑ 80:20) afforded 1-(pyridin-3-yl)-1H-indole **48** as a brown oil (318 mg, 33 %). **^{1}H-NMR** (400 MHz, $CDCl_3$) 6.75 (d, *J* = 3.3 Hz, 1 H, ArH), 7.30 – 7.17 (m, 2 H, 2 x ArH), 7.33 (d, *J* = 3.3 Hz, 1 H, ArH), 7.50 (dd, *J* = 8.2, 4.8 Hz, 1 H, ArH), 7.53 (d, *J* = 8.1 Hz, 1 H, ArH), 7.71 (d, *J* = 7.7 Hz, 1 H, ArH), 7.88 (dt, *J* = 8.2, 2.5, 1.5 Hz, 1 H, ArH), 8.62 (d, *J* = 3.9 Hz, 1 H, ArH), 8.85 (d, *J* = 2.3 Hz, 1 H, ArH). **^{13}C-NMR** (101 MHz, $CDCl_3$) 105.0, 110.1, 121.1, 121.6, 123.1, 124.3, 127.5, 129.7, 131.7, 135.9, 136.7, 145.7, 147.5. **ATR-IR** ν_{max} (neat)/cm^{-1}. 3102, 3048, 1584, 1516, 1483, 1454, 1331, 1211, 760, 739, 706. **m/z (EI)** 194.1 (M^+, 100), 167.1 (12), 139.0 (9), 116.0 (8), 89.0 (25), 78.0 (9) 63.0 (12), 51.0 (20). The data for this compound are consistent with those reported in the literature.[18]

Preparation of 1-(Pyridin-2-yl)-1*H*-indole (49)

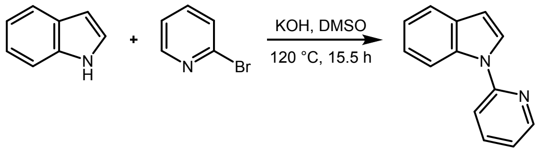

49, 60%

This substrate was prepared according to a literature procedure.[19] Indole (351 mg, 3 mmol, 1 equiv.), 2-bromopyridine (286 µL, 3 mmol, 1 equiv.), KOH (421 mg, 7.5 mmol, 2.5 equiv.) and dry DMSO (4.5 mL) were sealed in a pressure tube in a nitrogen-filled glovebox. The tube was removed, and the reaction mixture was stirred at 120 °C for 15.5 h. The reaction was then cooled to room temperature, poured onto sat. NH_4Cl solution (30 mL) and extracted into EtOAc (3 x 30 mL). The organic phases were combined, dried over $MgSO_4$, filtered and concentrated under reduced pressure. Purification by column chromatography (toluene) afforded 1-(pyridin-2-yl)-1*H*-indole **49** as a yellow oil (351 mg, 60 %). **^{1}H-NMR** (400 MHz, $CDCl_3$) 6.72 (dd, *J* = 3.5, 0.7 Hz, 1 H, ArH), 7.17 (ddd, *J* = 7.3, 4.9, 0.6 Hz, 1 H, ArH), 7.24 – 7.19 (m, 1 H, ArH), 7.30 (ddd, *J* = 8.4, 7.2, 1.3 Hz, 1 H, ArH), 7.51 (d, *J* = 8.2 Hz, 1 H, ArH), 7.67 (d, *J* = 7.8, 0.8 Hz, 1 H, ArH), 7.74 (d, *J* = 3.5 Hz, 1 H, ArH), 7.83 (ddd, *J* = 8.2, 7.4, 1.9 Hz, 1 H, ArH), 8.21 (dd, *J* = 8.4, 0.8 Hz, 1 H, ArH), 8.58 (d, *J* = 3.8 Hz, 1 H, ArH). **^{13}C-NMR** (101 MHz, $CDCl_3$) 105.7, 113.1, 114.7, 120.2, 121.2, 121.4, 123.3, 126.1, 130.6, 135.3, 138.5, 149.1, 152.7. **ATR-IR** ν_{max} (neat)/cm^{-1} 3107, 3051, 2926, 1691, 1587, 1522, 1470, 1450, 1435, 1344, 1209, 687. ***m/z*** **(EI)** 194.2 (M^+, 100), 167.1 (35), 139.1 (11), 116.1 (12), 89.1 (27), 78.1 (12), 63.1 (16), 51.1 (23). The data for this compound are consistent with those reported in the literature.[19]

Preparation of 3-(But-3-en-1-yl)-1-phenyl-1*H*-indole (**50**)

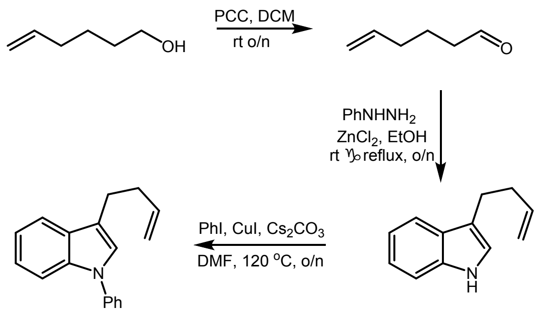

50, 5 % (3 steps)

The first step was carried out according to a literature procedure.[20] A solution of 5-hexen-1-ol (5 mL, 42 mmol, 1 equiv.) in DCM (20 mL) was added to a suspension of pyridinium chlorochromate (13.44 g, 62 mmol, 1.5 equiv.) in DCM (60 mL) at 0 °C. The mixture was then stirred at room temperature overnight before diluting with diethyl ether and filtering through a pad of silica gel. Careful removal of solvent under reduced pressure afforded 5-hexenal as a colourless oil, which was used crude with no further purification.

To a three-necked flask under argon and equipped with a stirrer bar and fitted with a condenser was added crude 5-hexenal and phenylhydrazine (4.13 mL, 42 mmol, 1 equiv.). This mixture was stirred for 1 h at room temperature then for 30 min at 100 °C. Zinc chloride (10.3 g, 75.6 mmol, 1.8 equiv.) in ethanol (46 mL) was then added and the mixture refluxed overnight. After cooling to room temperature, the mixture was filtered and the solvent was removed under reduced pressure. Hydrochloric acid (2 M) was added to the crude residue and the organic products were extracted into DCM. The organic layers were dried over Na_2SO_4, filtered and concentrated under reduced pressure. Purification by column chromatography (hexane:diethyl ether, 19:1 → 9:1) afforded 3-(but-3-en-1-yl)-1*H*-indole as an impure red oil (526 mg). The crude ^{1}H NMR and GCMS data were consistent with those reported in the literature.[21] **^{1}H-NMR** (400 MHz, $CDCl_3$) 2.51 (m, 2 H, CH_2), 2.85 - 2.95 (m, 2 H, CH_2), 5.02 (ddt, *J* = 10.1, 2.1, 1.2 Hz, 1 H, CH), 5.11 (dq, *J* = 17.2, 1.7 Hz, 1 H, CH), 5.97 (ddt, *J* = 17.0, 10.3, 6.5 Hz, 1 H, CH), 6.98 - 7.03 (m, 1 H, ArH), 7.11 - 7.17 (m, 1 H, ArH), 7.18 - 7.25 (m, 1 H, ArH), 7.37 (dt, *J* = 8.1, 0.8 Hz, 1 H, ArH), 7.61 - 7.67 (m, 1 H, ArH), 7.92 (br. s., 1 H, NH). GCMS (*m/z* = 171.2, retention time = 13.2 min).

Ullmann coupling was carried out according to General Procedure A from 3-(but-3-en-1-yl)-1*H*-indole (526 mg, 3.07 mmol, 1.1 equiv.) and iodobenzene (0.31 mL, 2.79 mmol, 1 equiv.) with copper (I) iodide (106 mg, 0.56 mmol, 0.2 equiv.) and cesium carbonate (1.82 g, 5.58 mmol, 2 equiv.) in DMF (6 mL). Purification by column chromatography (hexane) afforded 3-(but-3-en-1-yl)-1-phenyl-1*H*-indole **50** as a colourless oil (438 mg, 5 % over three steps). **^{1}H-NMR** (400 MHz, $CDCl_3$) 2.48 - 2.62 (m, 2 H, CH_2), 2.88 - 2.99 (m, 2 H, CH_2), 5.04 (ddt, *J* = 10.3, 2.1, 1.1, Hz, 1 H, CH), 5.14 (dm, *J* = 17.1, 1.8 Hz, 1 H, CH), 5.99 (ddt, *J* = 17.0, 10.3, 6.5 Hz, 1 H, CH), 7.16 - 7.21 (m, 2 H, 2 x ArH), 7.22 - 7.27 (m, 1 H, ArH), 7.29 - 7.39 (m, 1 H, ArH), 7.48 - 7.54 (m, 4 H, 4 x ArH), 7.56 - 7.61 (m, 1 H, ArH), 7.68 (dq, *J* = 7.8, 0.7 Hz, 1 H, ArH). **^{13}C-NMR**

(101 MHz, $CDCl_3$) 24.6, 34.1, 110.5, 114.8, 117.3, 119.2, 119.8, 122.4, 124.1, 125.1, 126.0, 129.0, 129.5, 136.0, 138.6, 140.0. **ATR-IR** ν_{max} (neat)/cm^{-1} 3057, 2918, 1639, 1595, 1498, 1454, 1379, 1379, 1317, 1228, 1134, 1072, 1014, 993, 906, 773, 694. ***m/z* (EI)** 247.2 (M^+, 12), 206.2 (100), 178.2 (13), 128.1 (21), 115.1 (6), 102.1 (8), 77.1 (34), 51.0 (18). **HRMS (CI):** calcd. for $C_{18}H_{18}N^+$ ($[M+H]^+$): 248.1439, found: 248.1435.

Preparation of 3-(Pent-4-en-1-yl)-1-phenyl-1*H*-indole (51)

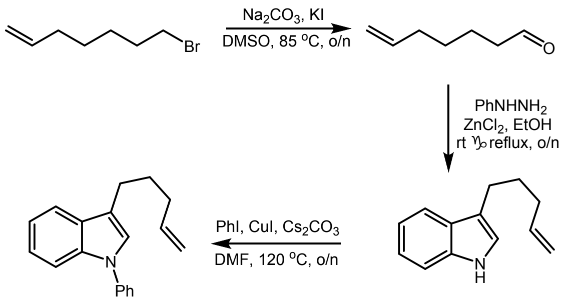

51, 7 % (3 steps)

The first step was carried out according to a literature procedure.[22] A solution of 7-bromoheptene (1.52 mL, 10 mmol, 1 equiv.), sodium carbonate (1.06 g, 10 mmol, 1 equiv.) and potassium iodide (1.66 g, 10 mmol, 1 equiv.) in DMSO (50 mL) was heated at 85 °C overnight. After cooling to room temperature, the mixture was diluted with diethyl ether and washed with sodium carbonate, water and brine. The combined organic layers were dried over Na_2SO4 and filtered. Careful removal of solvent under reduced pressure afforded 6-heptenal as a colourless oil which was used with no further purification.

To a three-necked flask under argon, equipped with a stirrer bar and fitted with a condenser was added crude 6-heptenal and phenylhydrazine (0.98 mL, 10 mmol, 1 equiv.). This mixture was stirred for 1 h at room temperature then for 30 min at 100 °C. Zinc chloride (2.45 g, 18 mmol, 1.8 equiv.) in ethanol (11 mL) was then added and the mixture refluxed overnight. After cooling to room temperature, the mixture was filtered and the solvent was removed under reduced pressure. Hydrochloric acid (2 M) was added to the crude residue and the organic products were extracted into DCM. The organic layers were dried over Na_2SO_4, filtered and concentrated under reduced pressure. Purification by column chromatography (hexane:diethyl ether, 19:1 → 9:1) afforded 3-(pent-4-en-1-yl)-1*H*-indole as an impure orange oil (395 mg) which was used without purification. The crude ^{1}H NMR and GCMS data of this compound were consistent with those reported in the literature.[23] **^{1}H-NMR** (400 MHz, $CDCl_3$) 1.76 - 1.94 (m, 2 H, CH_2), 2.12 - 2.30 (m, 2 H, CH_2), 2.73 - 2.89 (m, 2 H, CH_2), 5.00 - 5.06 (m, 1 H, CH), 5.10 (dq, *J* = 17.2, 1.7 Hz, 1 H, CH), 5.93 (ddt, *J* = 17.0, 10.3, 6.7 Hz, 1 H, CH), 6.97 - 7.01 (m, 1 H, ArH), 7.13 - 7.19 (m, 1 H, ArH), 7.21 - 7.26 (m, 1 H, ArH), 7.60 - 7.71 (m, 1 H, ArH), 7.88 (br. s., 1 H, NH). GCMS (*m/z* = 185.2, retention time = 13.727 min).

The Ullmann coupling was carried out according to General Procedure A from 3-(pent-4-en-1-yl)-1*H*-indole (395 mg, 2.13 mmol, 1.1 equiv.) and iodobenzene (0.22 mL,

1.94 mmol, 1 equiv.) with copper (I) iodide (74 mg, 0.39 mmol, 0.2 equiv.) and cesium carbonate (1.26 g, 3.88 mmol, 2 equiv.) in DMF (4 mL). Purification by column chromatography (hexane) afforded 3-(pent-4-en-1-yl)-1-phenyl-1*H*-indole **51** as a colourless oil (184 mg, 7 % over three steps). **^{1}H-NMR** (400 MHz, $CDCl_3$) 1.88 (quin, *J* = 7.5 Hz, 2 H, CH_2), 2.14 - 2.31 (m, 2 H, CH_2), 2.75 - 2.92 (m, 2 H, CH_2), 5.01 (ddt, *J* = 10.3, 2.1, 1.2 Hz, 1 H, CH), 5.08 (dm, *J* = 17.2, 1.7 Hz, 1 H, CH), 5.77 - 6.01 (m, 1 H, CH), 7.16 (s, 1 H, ArH), 7.19 (dd, *J* = 7.7, 1.1 Hz, 1 H, ArH), 7.21 - 7.26 (m, 1 H, ArH), 7.30 - 7.39 (m, 1 H, ArH), 7.49 - 7.55 (m, 4 H, 4 x ArH), 7.58 (dt, *J* = 8.1, 1.0 Hz, 1 H, ArH), 7.67 (dq, *J* = 7.7, 0.7 Hz, 1 H, ArH). **^{13}C-NMR** (101 MHz, $CDCl_3$) 24.5, 29.2, 33.6, 110.5, 114.7, 117.7, 119.3, 119.7, 122.3, 124.0, 125.0, 125.9, 129.1, 129.5, 136.1, 138.8, 140.0. **ATR-IR** ν_{max} (neat)/cm^{-1} 3057, 2926, 1637, 1595, 1498, 1454, 1377, 1317, 1228, 1132, 1014, 989, 906, 773, 694. ***m/z* (EI)** 261.2 (M^+, 17), 219.1 (11), 206.2 (100), 178.2 (14), 128.1 (21), 115.1 (9), 102.2 (7), 77.1 (27), 55.1 (36). **HRMS (CI)** calcd. for $C_{19}H_{20}N^+$ ($[M+H]^+$): 262.1590, found: 262.1595.

Preparation of 3-Cyclopropyl-1-phenyl-1*H*-indole (**61**)

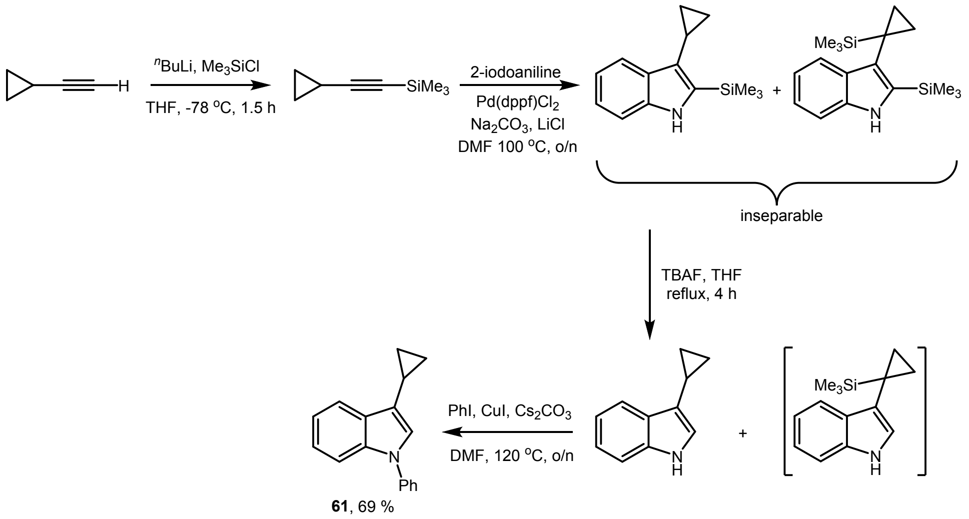

The first three steps were carried out according to a literature procedure.[24] To a stirring solution of cyclopropylacetylene (1.27 mL, 15 mmol, 1 equiv.) in dry THF (5 mL) at -78 °C was added ^{n}BuLi (10.7 mL of a 1.45 M solution in hexane, 15.45 mmol, 1.03 equiv.) dropwise. The solution was allowed to stir for 30 min at this temperature, after which chlorotrimethylsilane (1.98 mL, 15.6 mmol, 1.04 equiv.) was added. The reaction mixture was then stirred at -78 °C for 1 h. The reaction mixture was then warmed to room temperature, diluted with Et_2O and filtered through a pad of Na_2SO_4 layered on silica gel, eluting with pentane: diethyl ether (4:1). The filtrate was concentrated under reduced pressure to afford (cyclopropylethynyl)trimethylsilane, which was used without further purification.

$Pd(dppf)Cl_2$ (817 mg, 10 mmol, 0.1 equiv.), (cyclopropylethynyl)trimethylsilane (1.52 g, 11 mmol, 1.1 equiv.), 2-iodoaniline (2.19 g, 10 mmol, 1 equiv.), sodium carbonate (3.18 g, 30 mmol, 3 equiv.) and lithium chloride (445 mg, 10.5 mmol, 1.05 equiv.) in DMF (200 mL) under argon

were heated at 100 °C overnight. The reaction mixture was then cooled to room temperature, diluted with Et_2O and washed with aqueous ammonium chloride and water. The organic layer was then dried over Na_2SO_4, filtered and concentrated to afford an inseparable mixture of 2-(trimethylsilyl)-3-(1-(trimethylsilyl)cyclopropyl)-1*H*-indole and 3-cyclopropyl-2-(trimethylsilyl)-1*H*-indole (1.5:1 ratio, 777 mg) which was carried on to the next step as a mixture (GCMS retention times 15.105 and 14.276 min respectively). The ratio was calculated by comparing the integration of aromatic doublets in the proton NMR (7.70-7.75 ppm and 7.30-7.36 ppm). The relevant part of the ^{1}H NMR spectrum is shown below.

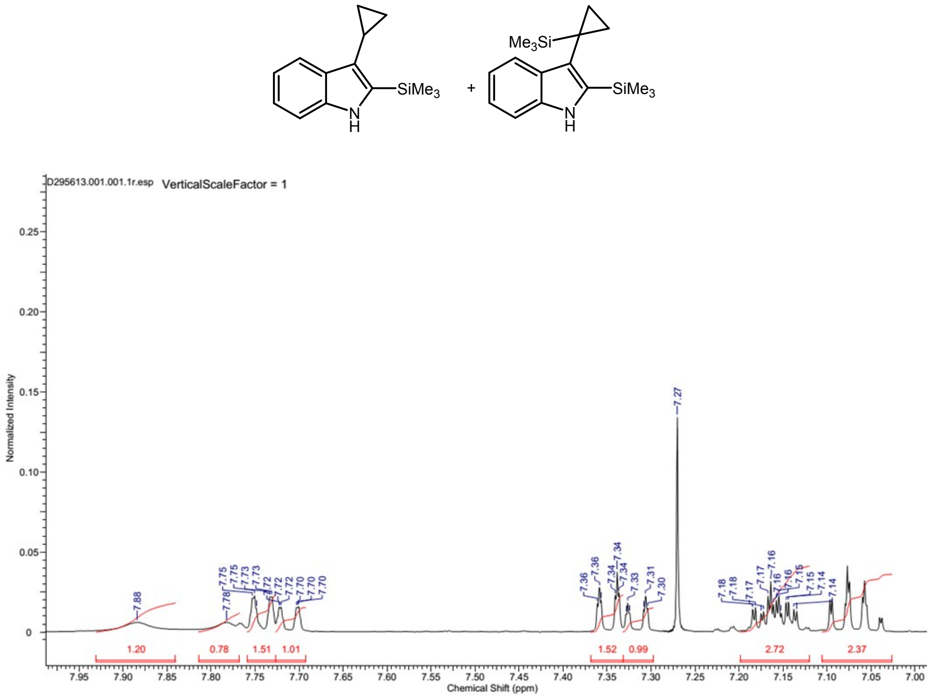

*Figure S1 - ^{1}H NMR Spectrum of the Mixture of 2-(trimethylsilyl)-3-(1-(trimethylsilyl)cyclopropyl)-1*H*-indole and 3-cyclopropyl-2-(trimethylsilyl)-1*H*-indole*

Also isolated after column chromatography was 3-cyclopropyl-1*H*-indole as an orange solid (97 mg, 6 %). **Mp** = 55-57 °C (lit. mp = 59-60 °C).[25] **^{1}H-NMR** (400 MHz, $CDCl_3$) 0.53 - 0.71 (m, 2 H, 2 x CH), 0.83 - 0.95 (m, 2 H, 2 x CH), 1.87 - 2.04 (m, 1 H, CH), 6.87 – 6.93 (m, 1 H, ArH), 7.13 (td, *J* = 7.4, 1.0 Hz, 1 H, ArH), 7.20 (td, *J* = 7.5, 1.3 Hz, 1 H, ArH), 7.35 (d, *J* = 8.0, 1 H, ArH), 7.75 (d, *J* = 8.0 Hz, 1 H, ArH), 7.84 (br. s., 1 H, NH). **^{13}C-NMR** (101 MHz, $CDCl_3$) 5.9, 6.1, 111.0, 119.1, 119.2 (two signals), 120.4, 122.1, 128.1, 136.3. **ATR-IR** v_{max} (neat)/cm^{-1} 3398, 3053, 2906, 1598, 1490, 1454, 1336, 1222, 1087, 1074, 1008, 798, 707. **m/z (EI)** 157.2 (M+, 93), 130.1 (100), 115.1 (14), 102.1 (21), 89.1 (15), 77.2 (33), 63.1 (22), 51.1 (21). The data for this compound are consistent with those reported in the literature.[25]

To the inseparable mixture of 2-(trimethylsilyl)-3-(1-(trimethylsilyl)cyclopropyl)-1*H*-indole and 3-cyclopropyl-2-(trimethylsilyl)-1*H*-indole (777 mg, ~3.39 mmol, 1 equiv.) was added tetra-*n*-butylammonium fluoride (464 mg, 4.98 mmol, 1.5 equiv.) in THF (7 mL). This mixture was refluxed for 4 h before quenching with water and extracting into DCM. The combined

organic layers were dried over Na_2SO_4, filtered and concentrated under reduced pressure. Purification by column chromatography (hexane:diethyl ether, 49:1 → 19:1) afforded 3-(1-(trimethylsilyl)cyclopropyl)-1H-indole as a yellow solid (141 mg, 6 %). **Mp** = 100-103 °C. **^{1}H-NMR** (400 MHz, $CDCl_3$) –0.03 (s, 9 H, $SiMe_3$), 0.77 - 0.90 (m, 4 H, 2 x CH_2), 6.94 (d, *J* = 2.3 Hz, 1 H, ArH), 7.12 (t, *J* = 7.5 Hz, 1 H, ArH), 7.19 (t, *J* = 7.5 Hz, 1 H, ArH), 7.33 (d, *J* = 8.1 Hz, 1 H, ArH), 7.73 (d, *J* = 7.9 Hz, 1 H, ArH), 7.82 (br. s., 1 H, NH). **^{13}C-NMR** (101 MHz, $CDCl_3$) –2.9, 4.2, 9.3, 110.9, 118.8, 120.1, 121.4, 121.6, 123.0, 128.4, 135.9. **ATR-IR** ν_{max} (neat)/cm^{-1} 3414, 3068, 2995, 2953, 2895, 1616, 1454, 1328, 1242, 1157, 1089, 904, 804, 763. ***m/z* (EI)** 229.2 (M^+, 36), 214.2 (13), 200.1 (6), 174.1 (15), 156.2 (98), 141.1 (7), 129.1 (38), 115.1 (15), 73.1 (100), 58.1 (13). **HRMS (CI)** calcd. for $C_{14}H_{20}NSi^+$ ($[M+H]^+$): 230.1365, found: 230.1366. Also isolated was 3-cyclopropyl-1*H*-indole (160 mg, 10 % over three steps), with data consistent with those reported above.

The Ullmann coupling was carried out according to General Procedure A from 3-cyclopropyl-1*H*-indole (250 mg, 1.6 mmol, 1.1 equiv.) and iodobenzene (0.16 mL, 1.44 mmol, 1 equiv.), with copper (I) iodide (94 mg, 0.29 mmol, 0.2 equiv.) and cesium carbonate (548 mg, 2.88 mmol, 2 equiv.) in DMF (1.5 mL). Purification by column chromatography (hexane) afforded 3-cyclopropyl-1-phenyl-1*H*-indole **61** as a colourless oil, which solidified to a waxy solid (233 mg, 69 %). **Mp** = 37-40 °C. **^{1}H-NMR** (400 MHz, $CDCl_3$) 0.67 - 0.75 (m, 2 H, CH_2), 0.89 - 1.00 (m, 2 H, CH_2), 1.94 - 2.08 (m, 1 H, CH), 7.06 (d, *J* = 1.0 Hz, 1 H, ArH), 7.19 (td, *J* = 7.5, 1.5 Hz, 1 H, ArH), 7.24 (td, *J* = 7.0, 1.5 Hz, 1 H, ArH), 7.30 - 7.37 (m, 1 H, ArH), 7.45 - 7.54 (m, 4 H, 4 x ArH), 7.56 (dt, *J* = 8.2, 0.9 Hz, 1 H, ArH), 7.76 - 7.83 (m, 1 H, ArH). **^{13}C-NMR** (101 MHz, $CDCl_3$) 6.0, 6.1, 110.5, 119.5, 119.8, 120.2, 122.5, 124.0, 124.2, 126.0, 129.5, 129.6, 136.1, 139.9. **ATR-IR** ν_{max} (neat)/cm^{-1} 3041, 3001, 1595, 1496, 1454, 1398, 1373, 1309, 1220, 1134, 1020, 869, 773, 648. ***m/z* (EI)** 233.2 (M^+, 70), 218.2 (7), 206.2 (37), 154.1 (20), 128.1 (45), 116.1 (13), 104.1 (15), 89.1 (15), 77.1 (100), 63.0 (12), 51.1 (66). **HRMS (CI)** calcd. for $C_{17}H_{16}N^+$ ($[M+H]^+$): 234.1283, found: 234.1280.

Preparation of 2-Cyclopropyl-1-phenyl-1*H*-indole (**73**)

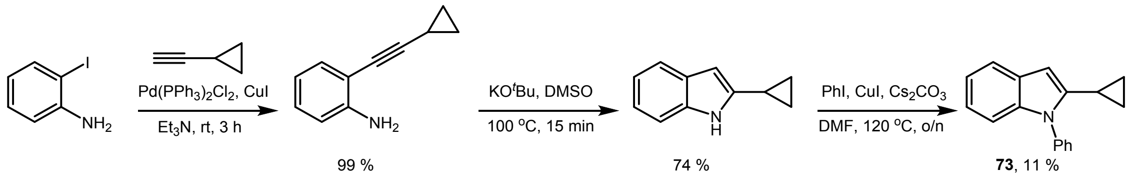

The first step was carried out according to a literature procedure.[26] Cyclopropylacetylene (1.86 mL, 22 mmol, 1.1 equiv.) was added to a stirred suspension of $Pd(PPh_3)_2Cl_2$ (350 mg, 0.5 mmol, 0.025 equiv.), CuI (46 mg, 0.24 mmol, 0.012 equiv.) and 2-iodoaniline 758 (4.38 g, 20 mmol, 1 equiv.) in triethylamine (100 mL). The mixture was stirred at room temperature for 3 h. After this time, the triethylamine was removed under reduced pressure and the crude mixture was diluted with water and DCM. The organic layer was separated, and the aqueous

layer was further extracted into DCM. The combined organic layers were dried over Na_2SO_4, filtered, and concentrated under reduced pressure. Purification by column chromatography (hexane:ethyl acetate, 0:100 ♑ 4:1) afforded 2-(cyclopropylethynyl)aniline as an orange oil (3.106 g, 99 %). **^{1}H-NMR** (400 MHz, $CDCl_3$) 0.78 - 0.96 (m, 4 H, 2 x CH_2), 1.46 - 1.57 (m, 1 H, CH), 6.72 (td, *J* = 7.5, 1.0 Hz, 1 H, ArH), 6.79 (d, *J* = 8.0 Hz, 1 H, ArH), 7.10 (td, *J* = 7.7, 1.5 Hz, 1 H, ArH), 7.25 (dd, *J* = 7.7, 1.5 Hz, 1 H, ArH) – NH_2 not observed. **^{13}C-NMR** (101 MHz, $CDCl_3$) 0.3, 8.9, 72.1, 98.8, 108.8, 114.1, 117.8, 128.8, 132.2, 147.9. **ATR-IR** ν_{max} (neat)/cm^{-1} 3396, 3311, 3082, 3053, 3005, 2360, 2341, 1685, 1614, 1487, 1321, 1309, 1151, 1095, 1051, 1020, 972, 883, 775, 675. **m/z (EI)** 150.0 (M^+, 87), 140.0 (4), 130.0 (100), 115.0 (15), 102.0 (26), 89.0 (17), 77.0 (28), 63.0 (29), 52.0 (22). **HRMS (CI)** calcd. for $C_{11}H_{12}N^+$ ($[M+H]^+$): 158.0964, found: 158.0963. The ^{1}H NMR for this compound is consistent with that reported in the literature.[27]

The second step was carried out according to a literature procedure.[28] Potassium *tert*-butoxide (5.55 g, 49.43 mmol, 2.5 equiv.) was added to a solution of 2-(cyclopropylethynyl)aniline (3.10 g, 19.77 mmol, 1 equiv.) in DMSO (20 mL). The mixture was stirred at 100 °C under argon for 15 min. After cooling to room temperature, brine was added to the reaction mixture and the product was extracted into DCM. The combined organic layers were dried over Na_2SO_4, filtered, and concentrated under reduced pressure. Purification by column chromatography (hexane:ethyl acetate, 100:0 ♑ 4:1) afforded 2-cyclopropyl-1*H*-indole as a white solid (2.285 g, 74 %). **Mp** = 64-67 °C (lit. mp = 57 °C).[28] **^{1}H-NMR** (400 MHz, $CDCl_3$) 0.72 - 0.85 (m, 2 H, 2 x CH), 0.91 - 1.04 (m, 2 H, 2 x CH), 1.91 - 2.04 (m, 1 H, CH), 6.16 (s, 1 H, ArH), 7.07 (t, *J* = 7.2 Hz, 1 H, ArH), 7.11 (t, *J* = 6.8 Hz, 1 H, ArH), 7.29 (d, *J* = 8.0 Hz, 1 H, ArH), 7.51 (d, *J* = 7.6 Hz, 1 H, ArH), 7.94 (br. s., 1 H, NH). **^{13}C-NMR** (101 MHz, $CDCl_3$) 7.3, 8.8, 97.7, 110.2, 119.6, 119.7, 121.0, 128.7, 135.7, 141.7. **ATR-IR** ν_{max} (neat)/cm^{-1} 3340, 1681, 1612, 1458, 1321, 1238, 1195, 1145, 1101, 1060, 1018, 970, 925, 823, 771, 702, 677 **m/z (EI)** 150.0 (M^+, 77), 140.1 (2), 130.0 (100), 117.0 (9), 102.0 (12), 89.0 (14), 77.0 (20), 63.0 (19), 51.0 (15). The data for this compound are consistent with those reported in the literature.[28]

The final step was carried out according to General Procedure A from 2-cyclopropyl-1*H*-indole (865 mg, 5.5 mmol, 1.1 equiv.) and iodobenzene (0.56 mL, 5 mmol, 1 equiv.) with copper (I) iodide (190 mg, 1 mmol, 0.2 equiv.) and cesium carbonate (3.26 g, 10 mmol, 2 equiv.) in DMF (10 mL). Purification by column chromatography (hexane) afforded 2-cyclopropyl-1-phenyl-1*H*-indole **73** as a yellow oil (146 mg, 11 %). **^{1}H-NMR** (400 MHz, $CDCl_3$) 0.74 - 0.83 (m, 2 H, CH_2), 0.83 - 0.94 (m, 2 H, CH_2), 1.65 - 1.77 (m, 1 H, CH), 6.21 (br. s., 1 H, ArH), 7.06 - 7.17 (m, 3 H, 3 x ArH), 7.41 - 7.51 (m, 3 H, 3 x ArH), 7.52 - 7.60 (m, 3 H, 3 x ArH). **^{13}C-NMR** (101 MHz, $CDCl_3$) 8.3, 8.3, 97.3, 109.9, 119.8, 120.0, 121.1, 127.5, 128.0, 128.1, 129.3, 138.2, 138.2, 144.0. **ATR-IR** ν_{max} (neat)/cm^{-1} 3080, 3051, 3007, 1595, 1552, 1498, 1454, 1400, 1346, 1296, 1217, 1153, 1014, 883, 827, 758, 744, 715, 663, 628. ***m/z* (EI)** 233.1 (M^+, 100), 218.1 (29), 206.1 (49), 190.1 (3), 178.1 (4), 165.1 (4), 154.1 (8), 140.1 (3), 130.0 (13), 118.1 (10), 108.6

(16), 89.0 (6), 77.0 (16), 63.0 (7), 51.0 (12). The data for this compound are consistent with those reported in the literature.[29]

Preparation of *N*-Phenyl-2-vinylaniline (92)

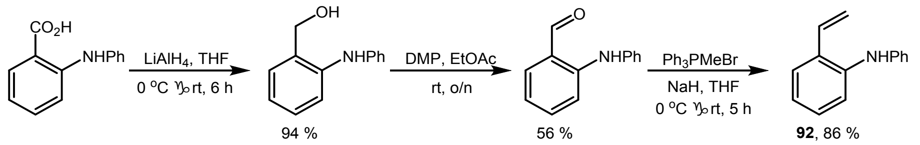

The first step was carried out according to a literature procedure.[30] A solution of *N*-phenylanthranilic acid (2.13 g, 10 mmol, 1 equiv.) in dry THF (40 mL) was added to a stirred solution of $LiAlH_4$ in THF (10 mL) at 0 °C. The reaction mixture was then stirred at room temperature for 6 h before quenching with EtOAc followed by NaOH. The organic layer was separated and dried over Na_2SO_4, filtered and concentrated under reduced pressure. Purification by column chromatography (hexane:diethyl ether, 9:1) afforded (2-(phenylamino)phenyl)methanol as an orange solid (1.88 g, 94 %). **Mp** = 55-56 °C (lit. mp = 59-62 °C).[31] **^{1}H-NMR** (400 MHz, $CDCl_3$) 4.74 (s, 2 H, CH_2), 6.87 - 6.98 (m, 2 H, 2 x ArH), 7.06 - 7.12 (m, 2 H, 2 x ArH), 7.19 - 7.33 (m, 4 H, 4 x ArH), 7.39 (dd, *J* = 8.0, 1.0 Hz, 1 H, ArH). **^{13}C-NMR** (101 MHz, $CDCl_3$) 64.6, 117.0, 118.2, 120.5, 120.9, 128.5, 129.2, 129.3, 129.6, 143.0, 143.1. **ATR-IR** v_{max} (neat)/cm^{-1} 3401, 1591, 1516, 1494, 1454, 1294, 1172, 1040, 880, 694. ***m/z* (EI)** 199.1 (M^+, 7), 180.2 (100), 167.0 (6), 101.9 (11), 90.1 (9), 77.0 (27), 64.5 (15), 51.0 (17). The data for this compound are consistent with those reported in the literature.[32]

The second step was carried out according to a modified literature procedure.[30] To a solution of Dess-Martin Periodinane (DMP) (10 g, 23.6 mmol, 2.5 equiv.) in EtOAc (190 mL) was added (2-[(phenylamino)phenyl]methanol (1.88 g, 9.44 mmol, 1 equiv.). The resulting mixture was stirred overnight at room temperature before being filtered and the solid washed with EtOAc. The filtrate was then concentrated under reduced pressure and purified by column chromatography (hexane:diethyl ether, 19:1) to afford 2-(phenylamino)benzaldehyde as a yellow solid (1.05 g, 56 %). **Mp** = 63-65 °C (lit. mp = 71-72 °C).[30] **^{1}H-NMR** (400 MHz, $CDCl_3$) 6.80 - 6.88, (m, 1 H, ArH), 7.12 - 7.20 (m, 1 H, ArH), 7.24 (d, *J* = 8.5 Hz, 1 H, ArH), 7.27 - 7.33 (m, 2 H, 2 x ArH), 7.34 - 7.43 (m, 3 H, 3 x ArH), 7.58 (dd, *J* = 7.7, 1.6 Hz, 1 H, ArH), 9.92 (d, *J* = 1.0 Hz, 1 H, CHO), 10.02 (br. s., 1 H, NH). **^{13}C-NMR** (101 MHz, $CDCl_3$) 112.9, 117.1, 119.4, 123.2, 124.4, 129.4, 135.5, 136.6, 139.7, 147.8, 194.2. **ATR-IR** v_{max} (neat)/cm^{-1} 3271, 3035, 2835, 1649, 1591, 1572, 1517, 1450, 1421, 1396, 1317, 1184, 1157, 1116, 898, 698, 693. ***m/z* (EI)** 197.2 (M^+, 56), 168.2 (100), 139.1 (10), 115.1 (9), 93.1 (11), 77.1 (26), 65.1 (16), 51.1 (39). The data for this compound are consistent with those reported in the literature.[30]

To a suspension of sodium hydride (312 mg, 13 mmol, 2.6 equiv.) in dry THF (50 mL) was added methyltriphenylphosphonium bromide (4.47 g, 12.5 mmol, 2.5 equiv.) at 0 °C. The

mixture was then stirred at this temperature for 30 min before the addition of 2-(phenylamino)benzaldehyde (986 mg, 5 mmol, 1 equiv.). The resulting mixture was stirred at room temperature for 5 h before being quenched with water and extracted into DCM. The combined organic layers were dried over Na_2SO_4, filtered and concentrated under reduced pressure. Purification by column chromatography (hexane:diethyl ether, 49:1) afforded *N*-phenyl-2-vinylaniline **92** as a yellow oil (835 mg, 86 %). **^{1}H-NMR** (400 MHz, $CDCl_3$) 5.33 (dd, J = 11.0, 1.5 Hz, 1 H, CH), 5.54 (br. s., 1 H, NH), 5.70 (dd, J = 17.4, 1.4 Hz, 1 H, CH), 6.85 - 6.98 (m, 4 H, 3 x ArH + CH), 7.00 - 7.06 (m, 1 H, ArH), 7.18 - 7.29 (m, 4 H, 4 x ArH), 7.49 (dd, J = 7.8, 1.5 Hz, 1 H, ArH). **^{13}C-NMR** (101 MHz, $CDCl_3$) 116.2, 117.1, 120.0, 120.4, 122.5, 127.1, 128.6, 129.3, 130.0, 132.8, 140.0, 144.1. **ATR-IR** ν_{max} (neat)/cm^{-1} 3389, 1593, 1494, 1452, 1298, 1174, 993, 910, 690. ***m/z* (EI)** 195.2 (M^+, 100), 194.2 (100), 180.1 (76), 167.2 (25), 152.1 (7), 139.1 (6), 128.1 (5), 118.1 (41), 102.1 (6), 91.1 (29), 83.7 (8), 77.1 (47), 65.1 (23), 51.1 (54). The data for this compound are consistent with those reported in the literature.[33]

Treating Substrates with Et_3SiH/KO^{t}Bu

Treatment of 1-Phenyl-1*H*-indole (22) with Et_3SiH/KO^{t}Bu

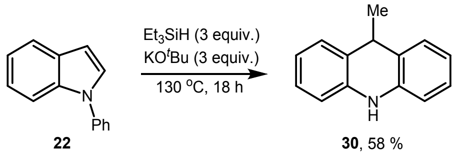

This reaction was carried out according to General Procedure B from 1-phenyl-1*H*-indole **22** (97 mg, 0.5 mmol, 1 equiv.). Purification by column chromatography (hexane:diethyl ether, 19:1) afforded 9-methyl-9,10-dihydroacridine **30** as a yellow solid (57 mg, 58 %). **Mp** = 120-123 °C (lit. mp = 123-125 °C).[34] **^{1}H-NMR** (400 MHz, $CDCl_3$) 1.38 (d, J = 7.3 Hz, 3 H, CH_3), 4.12 (q, J = 7.0 Hz, 1 H, CH), 6.04 (br. s., 1 H, NH), 6.72 (dd, J = 8.0, 1.0 Hz, 2 H, 2 x ArH), 6.91 (td, J = 7.4, 1.3 Hz, 2 H, 2 x ArH), 7.11 (td, J = 7.5, 1.5 Hz, 2 H, 2 x ArH), 7.19 (dd, J = 7.5, 0.8 Hz, 2 H, 2 x ArH). **^{13}C-NMR** (101 MHz, $CDCl_3$) 26.4, 36.7, 113.4, 120.8, 125.7, 126.8, 128.2, 139.0. **ATR-IR** ν_{max} (neat)/cm^{-1} 3374, 2961, 2918, 1601, 1580, 1477, 1445, 1300, 1254, 1157, 1130, 1032, 887, 739. ***m/z* (EI)** 195.1 (M^+, 7), 193.1 (1), 180.0 (100), 152.1 (7). **HRMS (CI)** calcd. for $C_{14}H_{14}N^+$ ($[M+H]^+$): 196.1121, found: 196.1116.

Treatment of 1-Phenyl-1*H*-indole (22) with Et_3SiH/NaO^{t}Bu

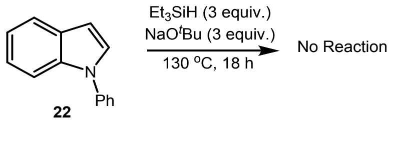

This reaction was carried out according to General Procedure B from 1-phenyl-1*H*-indole **22** (97 mg, 0.5 mmol, 1 equiv.), with NaO^{t}Bu (144 mg, 1.5 mmol, 3 equiv.) instead of KO^{t}Bu. No

reaction was found to occur, and after column chromatography (hexane), **22** was recovered with data consistent with those reported above (86 mg, 89 %).

Treatment of 1-Phenyl-1*H*-indole (22) with KO^{t}Bu Alone

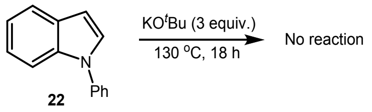

1-Phenyl-1*H*-indole (97 mg, 0.5 mmol, 1 equiv.) and potassium *tert*-butoxide (168 mg, 1.5 mmol, 3 equiv.) were sealed in a pressure tube in a glovebox under nitrogen. The tube was removed and heated at 130 °C for 18 h behind a safety shield. After cooling to room temperature, the mixture was diluted with water (50 mL) and extracted into diethyl ether (3 x 50 mL). The combined organic layers were dried over Na_2SO_4, filtered and concentrated under reduced pressure. No reaction was found to occur, and **22** was recovered with data consistent with those reported above (95 mg, 98 %).

Treatment of 3-Methyl-1-phenyl-1*H*-indole (23) with Et_3SiH/KO^{t}Bu

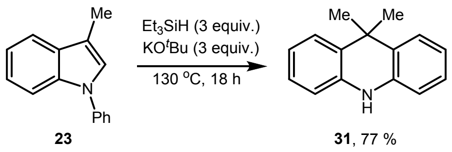

This reaction was carried out according to General Procedure B from 3-methyl-1-phenyl-1*H*-indole **23** (104 mg, 0.5 mmol, 1 equiv.). Purification by column chromatography (hexane:diethyl ether, 9:1 → 4:1) afforded 9,9-dimethyl-9,10-dihydroacridine **31** as a white solid (81 mg, 77 %). **Mp** = 115-118 °C (lit. mp = 120 °C).[35] **^{1}H-NMR** (400 MHz, $CDCl_3$) 1.61 (s, 6 H, 2 x CH_3), 6.14 (br. s., 1 H, NH), 6.71 (d, *J* = 7.8 Hz, 2 H, 2 x ArH), 6.94 (t, *J* = 7.4 Hz, 2 H 2 x ArH), 7.12 (td, *J* = 7.5, 1.5 Hz, 2 H 2 x ArH), 7.40 (dd, *J* = 7.9, 1.4 Hz, 2 H 2 x ArH). **^{13}C-NMR** (101 MHz, $CDCl_3$) 30.5, 36.2, 113.4, 120.6, 125.5, 126.7, 129.1, 138.5. **ATR-IR** ν_{max} (neat)/cm^{-1} 3358, 2957, 1605, 1580, 1477, 1450, 1317, 1242, 1213, 1036, 885, 711. ***m/z* (EI)** 209.1 (M^+, 10), 194.2 (100), 179.1 (5), 96.8 (8). The data for this compound are consistent with those reported in the literature.[35]

Treatment of 3-Ethyl-1-phenyl-1*H*-indole (24) with Et_3SiH/KO^{t}Bu

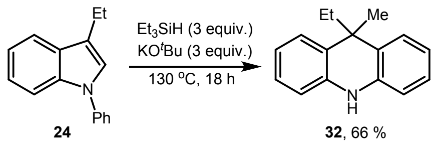

This reaction was carried out according to General Procedure B from 3-ethyl-1-phenyl-1*H*-indole **24** (111 mg, 0.5 mmol, 1 equiv.). Purification by column chromatography (hexane:diethyl ether, 100:0 ♑ 9:1), followed by recrystallisation from hexane, afforded 9-ethyl-9-methyl-9,10-dihydroacridine **32** as a yellow solid (74 mg, 66 %). **Mp** = 93-94 °C (lit. mp = 93-94 °C).[36] **^{1}H-NMR** (400 MHz, $CDCl_3$) 0.63 (t, *J* = 7.4 Hz, 3 H, CH_3), 1.66 (s, 3 H, CH_3), 1.86 (q, *J* = 7.5 Hz, 2 H, CH_2), 6.04 (br. s., 1 H, NH), 6.65 (d, *J* = 7.0 Hz, 2 H, 2 x ArH), 6.89 (m, 2 H, 2 x ArH), 7.09 (t, *J* = 7.2 Hz, 2 H, 2 x ArH), 7.30 (d, *J* = 7.8 Hz, 2 H, 2 x ArH). **^{13}C-NMR** (101 MHz, $CDCl_3$) 9.5, 30.1, 37.5, 40.4, 113.2, 120.3, 126.4, 126.7, 126.9, 138.8. **ATR-IR** ν_{max} (neat)/cm^{-1} 3393, 2960, 2918, 1605, 1578, 1474, 1450, 1321, 1144, 1026, 889, 685. ***m/z* (EI)** 222.9 (M^+, 3), 194.1 (100). The data for this compound are consistent with those reported in the literature.[37]

Treatment of 2,3-Dimethyl-1-phenyl-1*H*-indole (25) with Et_3SiH/KO^tBu

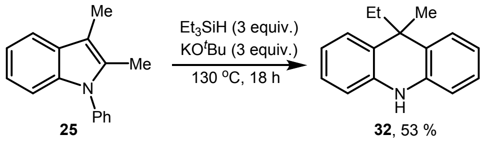

25 **32**, 53 %

This reaction was carried out according to General Procedure B from 2,3-dimethyl-1-phenyl-1*H*-indole **25** (111 mg, 0.5 mmol, 1 equiv.). Purification by column chromatography (hexane:diethyl ether, 100:0 ♑ 19:1) afforded 9-ethyl-9-methyl-9,10-dihydroacridine **32** as a yellow solid, with data consistent with those reported above (59 mg, 53 %).

Treatment of 1,3-Diphenyl-1*H*-indole (26) with Et_3SiH/KO^tBu

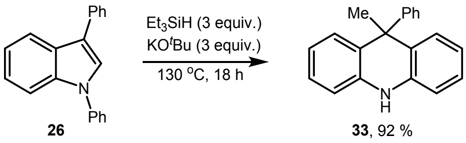

26 **33**, 92 %

This reaction was carried out according to General Procedure B from 1,3-diphenyl-1*H*-indole **26** (135 mg, 0.5 mmol, 1 equiv.). Purification by column chromatography (hexane:diethyl ether, 19:1 ♑ 9:1) afforded 9-methyl-9-phenyl-9,10-dihydroacridine **33** as a yellow solid (125 mg, 92 %). **Mp** = 93-94 °C (lit. mp = 96 °C).[38] **^{1}H-NMR** (400 MHz, $CDCl_3$) 1.85 (s, 3 H, CH_3), 6.23 (s, 1 H, NH), 6.73 (dd, *J* = 7.7, 0.9 Hz, 2 H, 2 x ArH), 6.75 - 6.83 (m, 4 H, 4 x ArH), 7.05 - 7.13 (m, 2 H, 2 x ArH), 7.22 (m, 1 H, ArH), 7.28 - 7.34 (m, 2 H, 2 x ArH), 7.34 - 7.39 (m, 2 H, 2 x ArH). **^{13}C-NMR** (101 MHz, $CDCl_3$) 30.7, 45.6, 113.2, 120.5, 125.9, 126.8, 127.8, 128.8, 128.9, 129.3, 138.0, 149.6. **ATR-IR** ν_{max} (neat)/cm^{-1} 3365, 3039, 2976, 2904, 1602, 1570, 1465, 1450, 1373, 1309, 1269, 1028, 807, 808, 686, 667, 623. ***m/z* (EI)** 271.2 (M^+, 11), 256.2 (100), 194.2 (80), 179.2 (8), 165.1 (6), 77.1 (77), 51.1 (38). **HRMS (CI)** calcd. for $C_{20}H_{18}N^+$ ($[M+H]^+$): 272.1439, found: 272.1436.

Treatment of 3-Octyl-1-phenyl-1*H*-indole (27) with Et_3SiH/KO^tBu

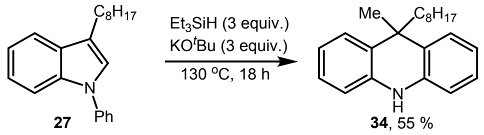

27 **34**, 55 %

This reaction was carried out according to General Procedure B from 3-octyl-1-phenyl-1*H*-indole **27** (153 mg, 0.5 mmol, 1 equiv.). Purification by column chromatography (hexane:diethyl ether, 19:1) afforded 9-methyl-9-octyl-9,10-dihydroacridine **34** as a yellow oil (85 mg, 55 %). This product was found to oxidise rapidly in air to form a stabilized radical, diagnosed by the broadening of the aromatic peaks in the proton NMR spectrum. This radical was reduced by dissolving the radical species in DCM and shaking with a 1 M solution of aqueous sodium dithionite in a separating funnel. The organic layer was then dried over Na_2SO_4, filtered and concentrated under reduced pressure to recover **34**. **^{1}H-NMR** (400 MHz, $CDCl_3$) 0.85 (t, J = 7.2 Hz, 3 H, CH_3), 0.94 - 1.06 (m, 2 H, CH_2), 1.07 - 1.25 (m, 10 H, 5 x CH_2), 1.65 (s, 3 H, CH_3), 1.77 - 1.88 (m, 2 H, CH_2), 6.04 (br. s., 1 H, NH), 6.64 (d, J = 7.8 Hz, 2 H, 2 x ArH), 6.89 (t, J = 7.3 Hz, 2 H, 2 x ArH), 7.04 - 7.14 (m, 2 H, 2 x ArH), 7.31 (dd, J = 7.9, 1.1 Hz, 2 H, 2 x ArH). **^{13}C-NMR** (101 MHz, $CDCl_3$) 14.1, 22.6, 25.0, 29.2, 29.4, 30.0, 30.7, 31.8, 40.0, 44.9, 113.2, 120.3, 126.4, 126.6, 127.3, 138.7. **ATR-IR** v_{max} (neat)/cm^{-1} 3402, 2922, 2852, 1606, 1579, 1481, 1323, 1251, 1097, 1035, 891, 721. ***m/z* (EI)** 307.2 (M^+, 1), 194.2 (100). **HRMS (CI)** calcd. for $C_{22}H_{30}N^+$ ($[M+H]^+$): 308.2373, found: 308.2375.

Treatment of 3-Butyl-1-phenyl-1*H*-indole (28) with Et_3SiH/KO^tBu

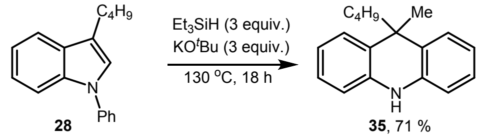

28 **35**, 71 %

This reaction was carried out according to General Procedure B from 3-butyl-1-phenyl-1*H*-indole **28** (125 mg, 0.5 mmol, 1 equiv.). Purification by column chromatography (hexane:diethyl ether, 19:1 ♑ 9:1) afforded 9-butyl-9-methyl-9,10-dihydroacridine **35** as an orange oil (84 mg, 71 %). **^{1}H-NMR** (400 MHz, $CDCl_3$) 0.77 (t, J = 7.4 Hz, 3 H, CH_3), 0.95 - 1.06 (m, 2 H, CH_2), 1.17 (sxt, J = 7.3 Hz, 2 H, CH_2), 1.66 (s, 3 H, CH_3), 1.80 - 1.89 (m, 2 H, CH_2), 6.04 (br. s., 1 H, NH), 6.65 (dd, J = 7.9, 1.1 Hz, 2 H, 2 x ArH), 6.91 (td, J = 7.5, 1.3 Hz, 2 H, 2 x ArH), 7.05 - 7.14 (m, 2 H, 2 x ArH), 7.32 (dd, J = 7.8, 1.0 Hz, 2 H, 2 x ArH). **^{13}C-NMR** 13.9, 23.1, 27.2, 30.7, 39.9, 44.7, 113.2, 120.3, 126.4, 126.6, 127.3, 138.6. **ATR-IR** v_{max} (neat)/cm^{-1} 3404, 2954, 2927, 1606, 1579, 1494, 1323, 1031, 889, 744. ***m/z* (EI)** 251.3 (M^+, 3), 236.3 (2), 194.2 (100), 57.1 (7). **HRMS (CI)** calcd. for $C_{18}H_{22}N^+$ ($[M+H]^+$): 252.1747, found: 252.1749.

Treatment of 1-(*p*-Tolyl)-1*H*-indole (29) with Et_3SiH/KO^tBu

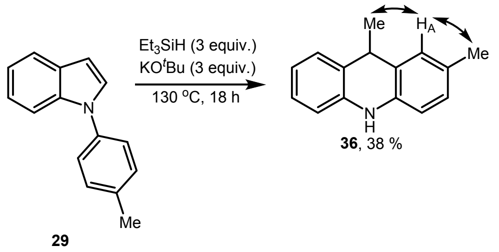

This reaction was carried out according to General Procedure B from 1-(*p*-tolyl)-1*H*-indole **29** (104 mg, 0.5 mmol, 1 equiv.). Purification by column chromatography (hexane:diethyl ether, 100:0 ♑ 9:1) afforded 2,9-dimethyl-9,10-dihydroacridine **36** as a yellow solid (48 mg, 49 %). **Mp** = 94-97 °C. **^{1}H-NMR** (400 MHz, $CDCl_3$) 1.36 (d, *J* = 7.0 Hz, 3 H, CH_3), 2.30 (s, 3 H, CH_3), 4.07 (q, *J* = 7.0 Hz, 1 H, CH), 5.96 (br. s., 1 H, NH), 6.63 (d, *J* = 8.0 Hz, 1 H, ArH), 6.70 (dd, *J* = 7.9, 1.1 Hz, 1 H, ArH), 6.88 (td, *J* = 7.5, 1.0 Hz, 1 H, ArH), 6.92 (dd, *J* = 8.0, 1.5 Hz, 1 H, ArH), 6.99 (s, 1 H, ArH), 7.10 (td, *J* = 7.8, 1.5 Hz, 1 H, ArH), 7.17 (d, *J* = 7.5 Hz, 1 H, ArH). **^{13}C-NMR** (101 MHz, $CDCl_3$) 20.7, 26.4, 36.8, 113.3, 113.4, 120.5, 125.6, 125.6, 126.8, 127.4, 128.2, 128.6, 130.0, 136.7, 139.3. **ATR-IR** v_{max} (neat)/cm^{-1} 3379, 2943, 2914, 2860, 1608, 1585, 1508, 1483, 1448, 1303, 1255, 1155, 1139, 1107, 1033, 929, 889, 867, 854, 808, 700, 613. ***m/z* (EI)** 209.0 (M^+, 6), 207.0 (4), 194.0 (100). **HRMS (CI)** calcd. for $C_{15}H_{16}N^+$ ($[M+H]^+$): 210.1277, found: 210.1274. The presence of this isomer was confirmed by NOESY, where H_A (6.99 pm) correlates to both methyl groups (1.36 and 2.30 ppm).

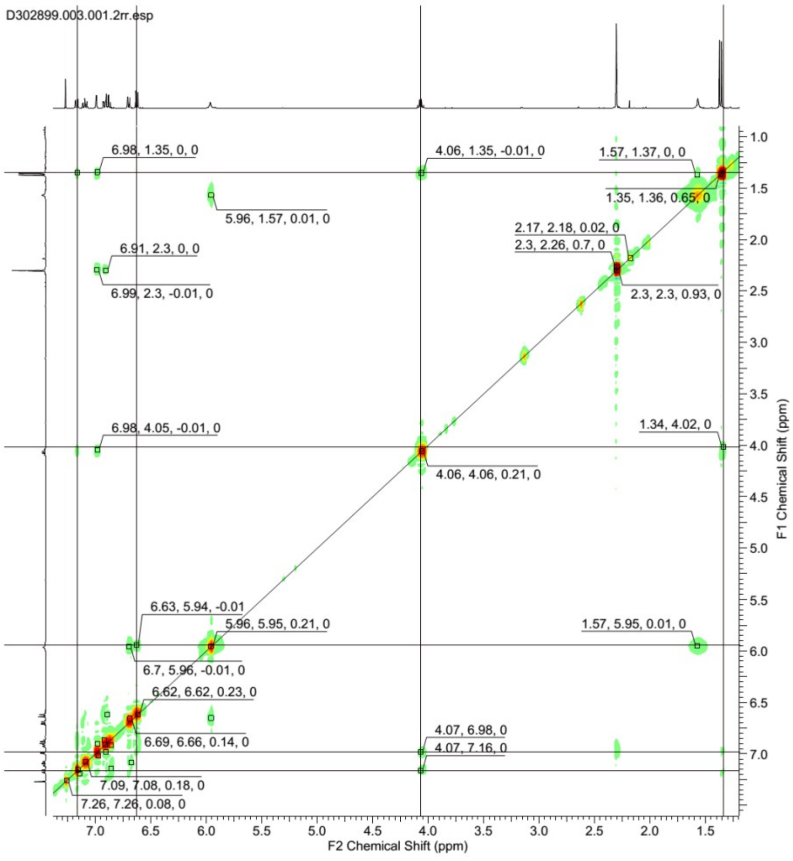

Figure S2 - NOESY Spectrum of ***37***

Treatment of 3-Methyl-1-(naphthalen-1-yl)-1*H*-indole (**47**) with Et_3SiH/KO^tBu

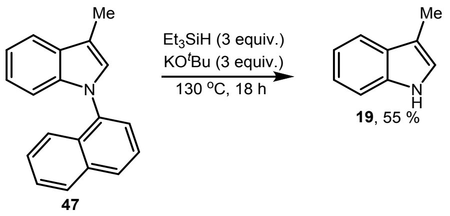

This reaction was carried out according to General Procedure B from 3-methyl-1-(naphthalen-1-yl)-1*H*-indole **47** (129 mg, 0.5 mmol, 1 equiv.). Purification by column chromatography (hexane:diethyl ether, 100:0 ♑ 9:1) afforded 3-methyl-1*H*-indole **19** as a white solid (36 mg, 55 %). **Mp** = 91-93 °C (lit. mp = 97 °C).[39] **^{1}H-NMR** (400 MHz, $CDCl_3$) 2.36 (d, *J* = 1.0 Hz, 3 H, CH_3), 6.98 (q, *J* = 1.0 Hz, 1 H, ArH), 7.14 (ddd, *J* = 7.8, 7.0, 1.0 Hz, 1 H, ArH), 7.20 (td, *J* = 7.0, 1.3 Hz, 1 H, ArH), 7.36 (dt, *J* = 8.0, 0.9 Hz, 1 H, ArH), 7.54 - 7.66 (m, 1 H, ArH), 7.87 (br. s., 1 H, NH). **^{13}C-NMR** (101 MHz, $CDCl_3$) 9.6, 110.9, 111.8, 118.8, 119.1, 121.5, 121.9, 128.3, 136.3. **ATR-IR ν_{max}** (neat)/cm^{-1} 3396, 3052, 2916, 1616, 1456, 1333, 1088, 1010, 738. **m/z (EI)** 131.1 (M^+, 55), 130.0 (100), 103.0 (8), 77.0 (19), 63.0 (6), 51.0 (9). The data for this compound are consistent with those reported in the literature.[40]

Treatment of 1-(Pyridin-3-yl)-1*H*-indole (**48**) with Et_3SiH/KO^tBu

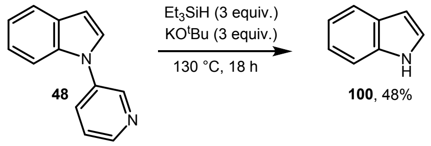

This reaction was carried out according to General Procedure B from 1-(pyridin-3-yl)-1*H*-indole (97 mg, 0.5 mmol, 1 equiv.). Purification by column chromatography (petroleum ether:ethyl acetate, 100:0 ♑ 95:5) afforded 1*H*-indole **100** as a white solid (27 mg, 48 %). **Mp** = 48-51 °C (lit. mp = 51-53 °C).[41] **^{1}H-NMR** (400 MHz, $CDCl_3$) 6.58 – 6.50 (m, 1 H, ArH), 7.15 – 7.05 (m, 1 H, ArH), 7.22 – 7.16 (m, 2 H, 2 x ArH), 7.40 (dd, *J* = 8.1, 0.9 Hz, 1 H, ArH), 7.66 (dd, *J* = 7.9, 1.0 Hz, 1 H, ArH), 8.14 (br. s, 1 H, NH). **^{13}C-NMR** (101 MHz, $CDCl_3$) 102.8, 111.1, 120.0, 120.9, 122.1, 124.2, 128.0, 135.9. **ATR-IR** ν_{max} (neat)/cm^{-1} 3399, 3100, 3050, 1454, 1414, 1333, 1246, 1059, 741, 608. ***m/z* (EI)** 117.1 (M^+, 100), 90.0 (46), 63.0 (25), 58.6 (8), 50.1 (11). The data for this compound are consistent with those reported in the literature.[42]

Treatment of 1-(Pyridin-2-yl)-1*H*-indole (**49**) with Et_3SiH/KO^tBu

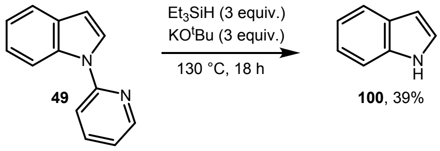

This reaction was carried out according to General Procedure B from 1-(pyridin-2-yl)-1*H*-indole **49** (97 mg, 0.5 mmol, 1 equiv.). Purification by column chromatography (hexane:ethyl acetate, 100:0 → 98:2) afforded 1*H*-indole **100** as a yellow oil, with data consistent with those reported above (22 mg, 39 %).

Treatment of 3-(But-3-en-1-yl)-1-phenyl-1*H*-indole (50) with Et_3SiH/KO^{t}Bu

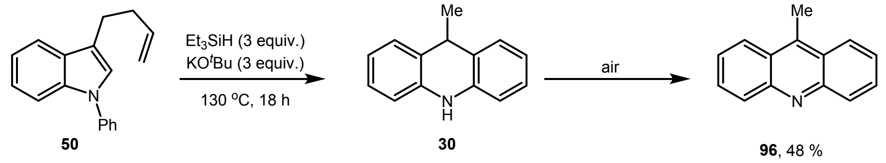

This reaction was carried out according to General Procedure B from 3-(but-3-en-1-yl)-1-phenyl-1*H*-indole **50** (124 mg, 0.5 mmol, 1 equiv. Purification by column chromatography (hexane:diethyl ether, 100:0 ♑ 9:1) afforded an impure sample which included dihydroacridine **30** (67 mg) . To facilitate isolation, the sample was intentionally oxidised by stirring in air for two weeks. Purification by column chromatography (hexane:diethyl ether, 4:1) afforded 9-methylacridine **96** as a yellow solid, with data consistent with those reported above (46 mg, 48 %).

Treatment of 3-(But-3-en-1-yl)-1-phenyl-1*H*-indole (50) with KO^{t}Bu Alone

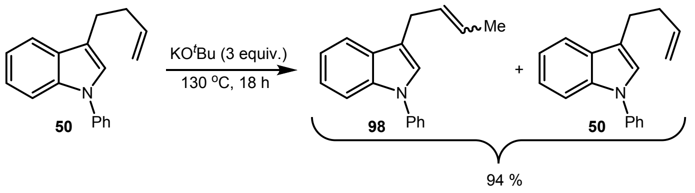

3-(But-3-en-1-yl)-1-phenyl-1*H*-indole **50** (124 mg, 0.5 mmol, 1 equiv.) and potassium *tert*-butoxide (168 mg, 1.5 mmol, 3 equiv.) were sealed in a pressure tube in a glovebox under nitrogen. The tube was removed and heated at 130 °C for 18 h behind a safety shield. After cooling to room temperature, the mixture was diluted with water (50 mL) and extracted into diethyl ether (3 x 50 mL). The combined organic layers were dried over Na_2SO_4, filtered and concentrated under reduced pressure to afford a mixture of (*E*/*Z*)-3-(but-2-en-1-yl)-1-phenyl-1*H*-indole **98** and 3-(but-3-en-1-yl)-1-phenyl-1*H*-indole **50** as a colourless oil (117 mg, 94 %). Evidence for this isomerisation can be seen in the ^{1}H-NMR spectra in Figure S3, where the spectrum from this reaction (top) is compared with the spectrum of the starting material (bottom). It can be seen that as well as the terminal alkene present in the starting material, a new peak has developed at 5.75 ppm indicative of an internal alkene.

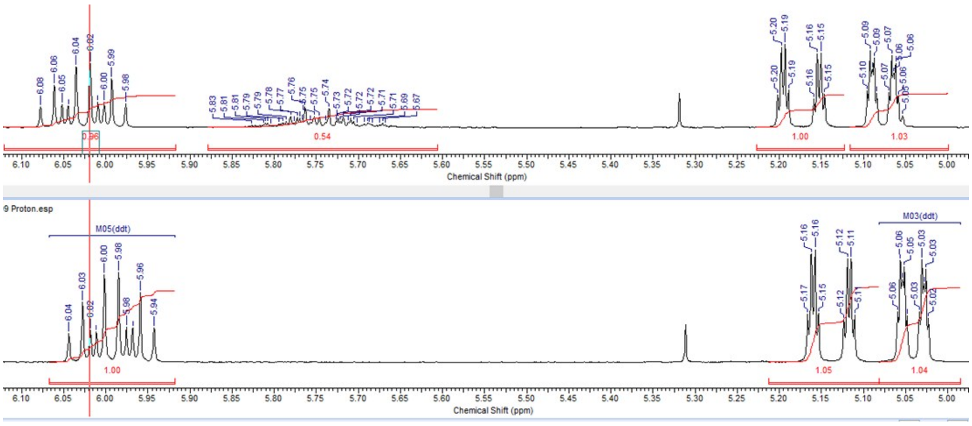

*Figure S3 - Isomerisation of **50** (bottom) to a Mixture of Isomers (top)*

Similarly, in the spectrum from this reaction (Figure S4, top), two isomeric methyl groups (1.76 and 1.85 ppm) can be seen which are not present in the starting material (bottom). This is further evidence for the isomerisation having occurred.

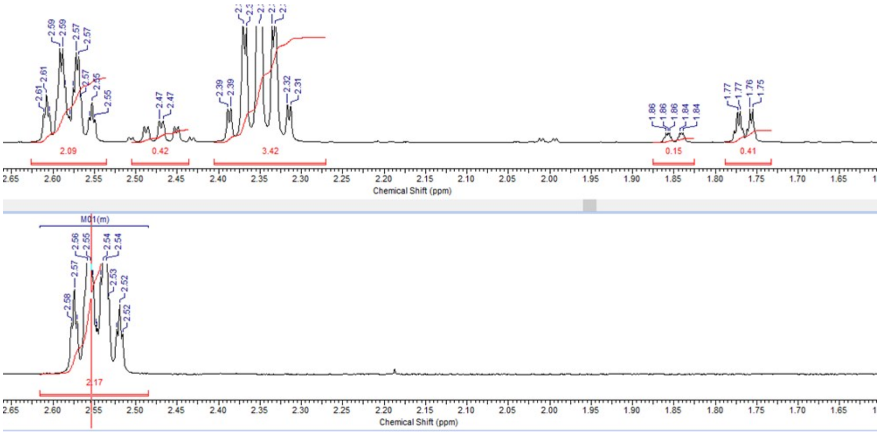

*Figure S4 - Isomerisation of **50** (bottom) to a Mixture of Isomers (top)*

GCMS also indicates the presence of isomers (*m/z* = 247.2), with retention times of 15.614, 15.721, 15.810 and 16.176 min. **^{13}C-NMR** (101 MHz, $CDCl_3$) 17.9, 23.2, 24.6, 26.6, 28.5, 30.3, 34.1, 110.4, 110.5, 110.6, 114.8, 116.3, 117.3, 118.3, 119.2, 119.3, 119.4, 119.8, 120.2, 120.4, 120.5, 120.6, 122.4, 122.7, 122.8, 124.0, 124.3, 124.4, 125.1, 125.6, 126.0, 126.1, 126.4, 126.5, 127.2, 129.0, 129.5, 129.6, 129.6, 130.6, 132.4, 136.0, 136.6, 138.6, 139.6, 140.0. **ATR-IR** ν_{max} (neat)/cm^{-1} 3047, 2960, 2926, 1718, 1595, 1498, 1454 1377, 1319, 1298, 1219, 1134, 1074, 1014, 960, 908, 773, 604.

Treatment of 3-(Pent-4-en-1-yl)-1-phenyl-1*H*-indole (51) with Et_3SiH/KO^{t}Bu

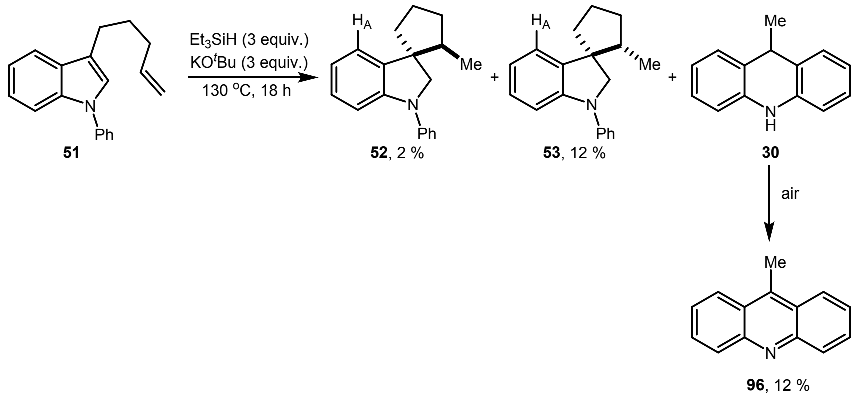

This reaction was carried out according to General Procedure B from 3-(pent-4-en-1-yl)-1-phenyl-1*H*-indole **51** (131 mg, 0.5 mmol, 1 equiv.). Purification by column chromatography (hexane:diethyl ether, 100:0 ♑ 9:1) afforded 2-methyl-1'-phenylspiro[cyclopentane-1,3'-indoline] **52** and **53** as a pair of diastereomers, which were inseparable from a small amount of starting material **51** by column chromatography (ratio of [**52** and **53**]:**51** = 10:1 by proton NMR, combined yield = 22 mg), and an impure sample containing 9-methyl-9,10-dihydroacridine **30** as a yellow solid (41 mg), with data consistent with those reported above. The mixture of diastereomers was then dissolved in 1:1 MeOH:DMSO (1 mL) and purified by Mass Directed AutoPrep on an Xbridge column using acetonitrile:water with an ammonium carbonate modifier to afford (1*R**,2*S**)-2-methyl-1'-phenylspiro[cyclopentane-1,3'-indoline] **53** as a colourless oil (16 mg, 12 %); **^{1}H-NMR** (600 MHz, $CDCl_3$) 0.84 (d, *J* = 7.0 Hz, 3 H, CH_3), 1.25 - 1.41 (m, 1 H, CH), 1.65 - 1.77 (m, 1 H, CH), 1.79 - 1.86 (m, 1 H, CH), 1.88 - 1.93 (m, 1 H, CH), 1.95 - 2.01 (m, 1 H, CH), 2.01 - 2.10 (m, 2 H, CH_2), 3.59 (d, *J* = 9.2 Hz, 1 H, CH), 3.85 (d, *J* = 9.5 Hz, 1 H, CH), 6.80 (td, *J* = 7.5, 0.7 Hz, 1 H, ArH), 6.95 (t, *J* = 7.3 Hz, 1 H, ArH), 7.08 - 7.13 (m, 2 H, 2 x ArH), 7.16 (d, *J* = 8.1 Hz, 1 H, ArH), 7.25 (d, *J* = 7.7 Hz, 2 H, 2 x ArH), 7.29 - 7.44 (m, 2 H, 2 x ArH). **^{13}C-NMR** (151 MHz, $CDCl_3$) 13.8, 21.8, 32.2, 40.1, 44.9, 54.0, 59.6, 108.2, 117.4, 118.9, 120.6, 122.4, 127.2, 129.1, 137.1, 144.0, 146.8. **ATR-IR** v_{max} (neat)/cm^{-1} 2954, 2875, 1591, 1501, 1484, 1385, 1281, 742, 695. ***m/z* (CI)** 264.0 $(M+H)^+$ **HRMS (CI)** calcd. for $C_{19}H_{22}N^+$ ($[M+H]^+$): 264.1752, found: 264.1757; and (1*R**,2*R**)-2-methyl-1'-phenylspiro[cyclopentane-1,3'-indoline] **52** as a colourless oil (2 mg, 2 %). **^{1}H-NMR** (600 MHz, $CDCl_3$) 0.79 (d, *J* = 7.0 Hz, 3 H, CH_3), 1.76 - 2.04 (m, 6 H, 3 x CH_2), 2.14 - 2.21 (m, 1 H, CH), 3.70 (d, *J* = 9.2 Hz, 1 H, CH), 3.94 (d, *J* = 9.2 Hz, 1 H, CH), 6.77 (td, *J* = 7.8, 0.7 Hz, 1 H, ArH), 6.95 (t, *J* = 7.7 Hz, 1 H, ArH), 7.06 - 7.11 (m, 2 H, 2 x ArH), 7.15 (dd, *J* = 8.8, 1.1 Hz, 1 H, ArH), 7.21 - 7.24 (m, 2 H, 2 x ArH), 7.31 - 7.37 (m, 2 H, 2 x ArH). **^{13}C-NMR** (151 MHz, $CDCl_3$) 15.6, 22.3, 32.9, 38.4, 44.6, 53.9, 65.3, 107.9, 117.5, 118.6, 120.7, 124.8, 127.1, 129.1, 136.3, 144.1, 147.0. **ATR-IR** v_{max} (neat)/cm^{-1} 2954, 2875, 1591, 1501, 1481, 1384, 1286, 742, 695. ***m/z* (CI)** 264.0 $(M+H)^+$. **HRMS (CI)** calcd. for $C_{19}H_{22}N^+$ ($[M+H]^+$): 264.1752, found: 264.1758. The relative stereochemistry of the minor diastereomer **52** was determined by NOESY, where there is a correlation between H_A and the methyl group as shown in the spectra below (Figure S5). This is not present in the major diastereomer **53** (Figure S6). To facilitate isolation of the 9,10-dihydroacridine **30**, the sample was intentionally oxidised in air. After stirring in air for 2 weeks,

the sample was found to have oxidised to 9-methylacridine **96**. Purification by column chromatography (hexane:diethyl ether, 4:1) afforded 9-methylacridine **96** as an orange solid (12 mg, 12 %). **Mp** = 109-111 °C (lit. mp = 113-114 °C). **^{1}H-NMR** (400 MHz, $CDCl_3$) 3.15 (s, 3 H, CH_3), 7.57 (ddd, *J* = 8.8, 6.5, 1.3 Hz, 2 H, 2 x ArH), 7.78 (ddd, *J* = 8.7, 6.6, 1.3 Hz, 2 H, 2 x ArH), 8.24 (d, *J* = 8.5 Hz, 1 H, 2 x ArH), 8.26 - 8.30 (m, 2 H, 2 x ArH). **^{13}C-NMR** (101 MHz, $CDCl_3$) 13.6, 124.5, 125.4, 125.5, 129.8, 130.2, 142.3, 148.4. **ATR-IR** v_{max} (neat)/cm^{-1} 3061, 3049, 2922, 1627, 1610, 1556, 1519, 1413, 1379, 1151, 943, 906, 858, 823, 686, 650. ***m/z* (EI)** 193.1 (M^+, 100), 165.2 (12), 89.2 (15), 74.0 (16), 63.0 (26), 52.0 (17). The data for this compound are consistent with those reported in the literature.[43]

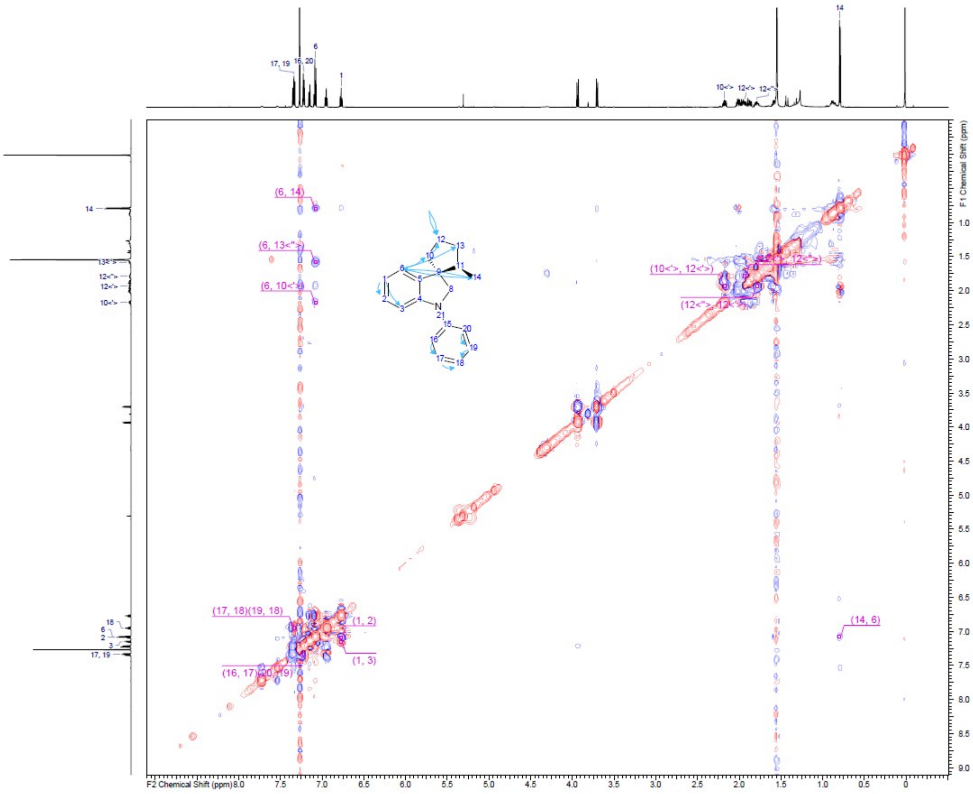

*Figure S5 – NOESY spectrum of **52***

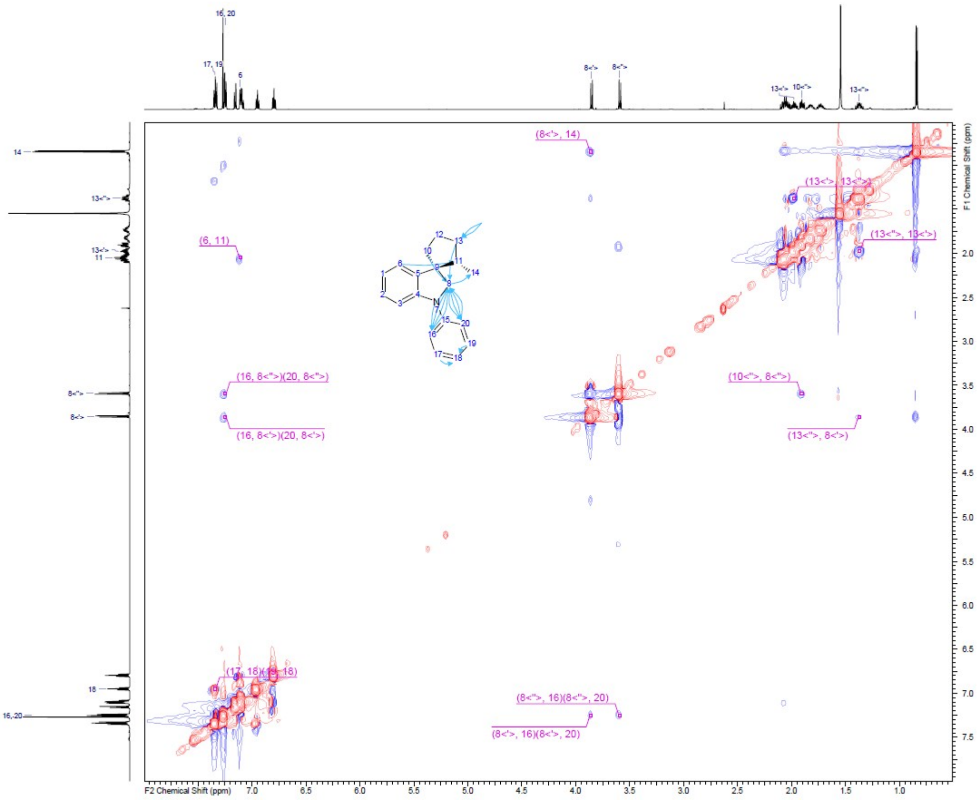

*Figure S6 - NOESY spectrum of **53***

Treatment of 3-(Pent-4-en-1-yl)-1-phenyl-1*H*-indole (**51**) with KO^{t}Bu Alone

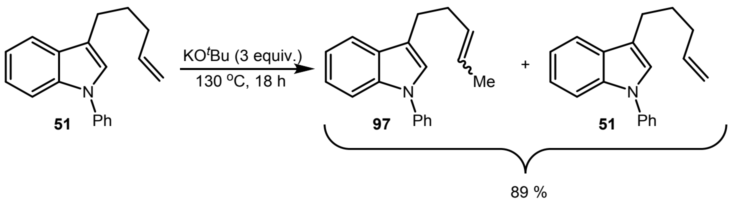

3-(Pent-4-en-1-yl)-1-phenyl-1*H*-indole **51** (131 mg, 0.5 mmol, 1 equiv) and potassium *tert*-butoxide (168 mg, 1.5 mmol, 3 equiv.) were sealed in a pressure tube in a glovebox under nitrogen. The tube was removed and heated at 130 °C for 18 h behind a safety shield. After cooling to room temperature, the mixture was diluted with water (50 mL) and extracted into diethyl ether (3 x 50 mL). The combined organic layers were dried over Na_2SO_4, filtered and concentrated under reduced pressure. Purification by column chromatography (hexane) afforded an inseparable mixture of (*E*/*Z*)-3-(pent-3-en-1-yl)-1-phenyl-1*H*-indole **97** and 3-(pent-4-en-1-yl)-1-phenyl-1*H*-indole **51** as a colourless oil (117 mg, 89 %). Evidence for this isomerisation can be seen in the ^{1}H NMR spectra in Figure S7, where the spectrum from this reaction (top) is compared with the spectrum of the starting material (bottom). It can be seen that as well as the terminal alkene present in the starting material, a new peak has developed at 5.6 ppm indicative of an internal alkene.

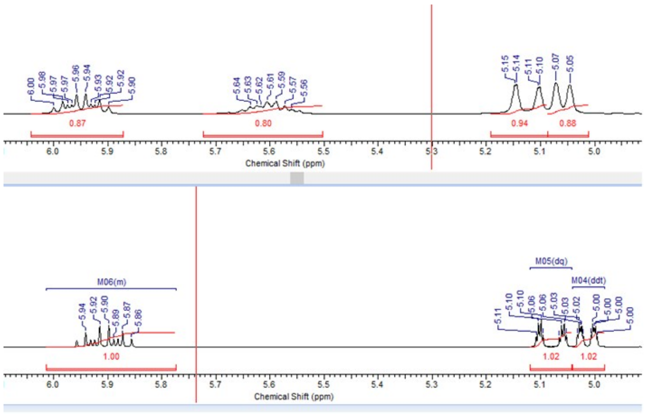

*Figure S7 - ¹H NMR Spectrum Showing the isomerisation of **51** (bottom) to a mixture of isomers (top)*

Similarly, in the spectrum from this reaction (Figure S8, top), two isomeric methyl doublets (1.68 and 1.74 ppm) can be seen which are not present in the starting material (bottom). This is further evidence for the isomerisation having occurred.

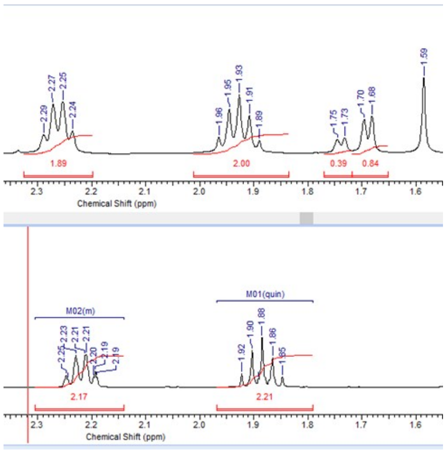

*Figure S8 - ¹H NMR spectrum showing the isomerisation of **51** (bottom) to a mixture of isomers (top)*

GCMS also indicates the presence of isomers from this reaction (*m/z* = 261.2), with retention times of 16.096 and 16.158 min. **^{13}C-NMR** (101 MHz, $CDCl_3$) 12.9, 18.0, 24.4, 25.0, 25.3, 27.4, 29.2, 33.0, 33.6, 110.5, 114.7, 117.6, 117.7, 119.2, 119.3, 119.7, 119.8, 120.3, 121.1, 122.3, 124.0, 124.3, 125.0, 125.3, 125.9, 129.1, 129.5, 130.3, 131.1, 136.0, 136.1, 138.7, 140.0. **ATR-IR** ν_{max} (neat)/cm^{-1} 3045, 2914, 2846, 1637, 1595, 1498, 1454, 1379, 1317, 1228, 1132, 1014, 906, 773, 694.

Treatment of 3-Cyclopropyl-1-phenyl-1*H*-indole (61) with Et_3SiH/KO^{t}Bu

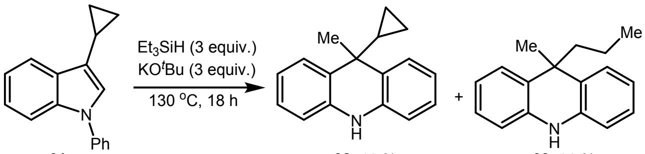

61 **62**, 13 % **63**, 30 %

This reaction was carried out according to General Procedure B from 3-cyclopropyl-1-phenyl-1*H*-indole **61** (116 mg, 0.5 mmol, 1 equiv.). Purification by column chromatography

(hexane:diethyl ether, 9:1) afforded a yellow oil which consisted of an inseparable mixture (1:2.4 ratio based on integration of CH_3 groups at 1.68 and 1.60 ppm) of 9-cyclopropyl-9-methyl-9,10-dihydroacridine **62** (13 %); **^{1}H-NMR** (400 MHz, $CDCl_3$) 0.18 - 0.26 (m, 2 H, CH_2), 0.32 - 0.40 (m, 2 H, CH_2), 0.95 - 1.14 (m, 1 H, CH), 1.54 (s, 3 H, CH_3), 6.09 (br. s., 1 H, NH), 6.71 (dd, *J* = 8.0, 1.3 Hz, 2 H, 2 x ArH), 6.91 - 6.96 (m, 2 H, 2 x ArH), 7.11 - 7.16 (m, 2 H, 2 x ArH), 7.49 (d, *J* = 7.8 Hz, 2 H, 2 x ArH). ***m/z* (EI)** 235.3 (M^+, 8), 220.2 (18), 207.1 (8), 194.1 (100), 178.9 (7), 165.1 (5). **HRMS (CI)** calcd. for $C_{17}H_{18}N^+$ ($[M+H]^+$): 236.1434, found: 236.1437; and 9-methyl-9-propyl-9,10-dihydroacridine **63** (30 %). **^{1}H-NMR** (400 MHz, $CDCl_3$) 0.75 (t, *J* = 7.5 Hz, 3 H, CH_3), 0.94-1.12 (m, 2H, CH_2), 1.66 (s, 3 H, CH_3), 1.75 - 1.85 (m, 2 H, CH_2), 6.03 (br. s, 1 H, NH), 6.64 (dd, *J* = 7.9, 1.1 Hz, 2 H, 2 x ArH), 6.86 - 6.91 (m, 2 H, 2 x ArH), 7.06 - 7.11 (m, 2 H, 2 x ArH), 7.31 (d, *J* = 7.8 Hz, 2 H, 2 x ArH). ***m/z* (EI)** 237.2 (M^+, 3), 222.2 (3), 194.2 (100). **HRMS (CI)** calcd. for $C_{17}H_{20}N^+$ ($[M+H]^+$): 238.1590, found: 238.1594. The ^{1}H NMR signals for compounds **62** and **63** are separately assigned above from a ^{1}H NMR spectrum of the mixture, making use of integrals to differentiate them. The ^{13}C NMR spectrum represents the peaks from both components. **^{13}C-NMR** (101 MHz, $CDCl_3$) 1.2, 14.4, 18.4, 23.6, 24.3, 30.5, 38.9, 40.0, 47.7, 138.2, 113.3, 120.3, 120.4, 126.4, 126.5, 126.7, 126.8, 127.1, 127.3, 138.6, 139.2. **ATR-IR** ν_{max} (neat)/cm^{-1} 3400, 2953, 2927, 2358, 1606, 1577, 1473, 1321, 1029, 694. Compounds **62** and **63** were separated by GCMS, with retention times of 15.650 and 15.159 min respectively. The yields were calculated by addition of 1,3,5-trimethoxybenzene (8.4 mg, 0.05 mmol, 10 mol %) as an internal standard to the sample of the mixture. The signal of the three methoxy groups (3.80 ppm) was integrated to 9 integral units, and the relative intensities of the methyl groups of **62** (1.68 ppm, 3.80 integral units) and **63** (1.60 ppm, 8.99 integral units) were used to calculate yields. For **62**, yield = [3.80/3] x 10 = 13 %. For **63**, yield = [8.99/3] x 10 = 30 %.

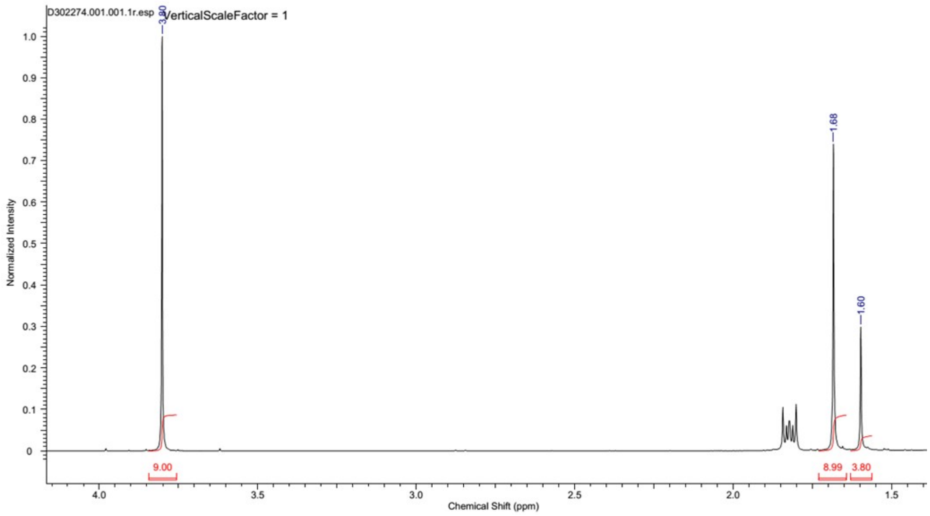

*Figure S9 - Determination of the Yields of **62** and **63** by the Addition of an Internal Standard to the ^{1}H NMR*

Treatment of 2-Cyclopropyl-1-phenyl-1*H*-indole (73) with Et_3SiH/KO^tBu

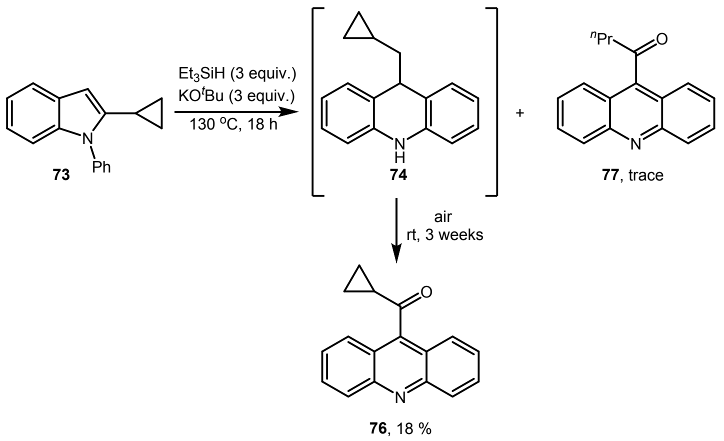

This reaction was carried out according to General Procedure B from 2-cyclopropyl-1-phenyl-1*H*-indole **73** (117 mg, 0.5 mmol, 1 equiv.). Purification by column chromatography (hexane:ethyl acetate, 100:0 ♑ 9:1) afforded an impure mixture of products which contained 9-(cyclopropylmethyl)-9,10-dihydroacridine **74**. **^{1}H-NMR** (400 MHz, $CDCl_3$) – 0.14 - – 0.05 (m, 2 H, 2 x CH), 0.32 - 0.41 (m, 2 H, 2 x CH), 0.55 - 0.67 (m, 1 H, CH), 1.49 (t, *J* = 7.0 Hz, 2 H, CH_2), 4.03 (t, *J* = 6.9 Hz, 1 H, CH), 6.03 (br. s., 1 H, NH), 6.73 (dd, *J* = 7.8, 1.0 Hz, 2 H, 2 x ArH), 6.90 (td, *J* = 7.4, 1.2 Hz, 2 H, 2 x ArH), 7.11 (td, *J* = 7.6, 1.5 Hz, 2 H, CH_2), 7.18 (d, *J* = 7.5 Hz, 2 H, 2 x ArH). GCMS indicated that oxidation had occurred at the temperature of the instrument (300 °C), and showed a mass matching $[M-2H]^+$ (233.1) with a retention time of 15.41 min. The crude mixture was dissolved in EtOAc and stirred at room temperature open to air for three weeks, adding more solvent as necessary. After this time, the mixture was concentrated under reduced pressure and purified by column chromatography (hexane:ethyl acetate, 100:0 ♑ 80:20). This afforded acridin-9-yl(cyclopropyl)methanone **76** as a yellow solid (21 mg, 18 %). **Mp** = 120 – 123 °C. **^{1}H-NMR** (400 MHz, $CDCl_3$) 1.29 - 1.37 (m, 2 H, 2 x CH), 1.62 - 1.69 (m, 2 H, 2 x CH), 2.45 - 2.56 (m, 1 H, CH), 7.62 (t, *J* = 7.5 Hz, 2 H, 2 x ArH), 7.85 (t, *J* = 7.7 Hz, 2 H, 2 x ArH), 7.99 (d, *J* = 8.5 Hz, 2 H, 2 x ArH), 8.25 - 8.44 (m, 2 H, 2 x ArH). **^{13}C-NMR** (101 MHz, $CDCl_3$) 14.0, 24.9, 121.5, 125.2, 126.9, 129.7, 130.6, 146.2, 148.5, 207.3. **ATR-IR** ν_{max} (neat)/cm^{-1} 247.1 (M^+, 100), 232.1 (11), 218.1 (29), 206.0 (50), 191.1 (9), 178.1 (87), 151.1 (42), 125.0 (8), 101.1 (5), 77.0 (10), 69.0 (14). ***m/z* (EI)** 247.1 (M^+, 100), 232.1 (11), 218.1 (30), 206.0 (51), 191.1 (10), 178.1 (88), 163.0 (4), 151.1 (42), 140.1 (2), 125.0 (7), 114.1 (3), 101.1 (5), 87.0 (4), 77.0 (10), 69.0 (9), 63.0 (4), 51.0 (4). **HRMS (CI)** calcd. for $C_{17}H_{14}NO^+$ ($[M+H]^+$): 248.1075, found: 248.1073. Also isolated was an impure fraction from which compound **77** was tentatively identified by GCMS, (*m/z* = 249.1), with a retention time of 15.62 min. The propyl group was also detected by ^{1}H NMR. **^{1}H NMR** (400 MHz, $CDCl_3$) 1.11 (t, *J* = 7.4 Hz, 3 H, CH_3), 1.87 - 1.99 (quin, *J* = 7.3 Hz, 2 H, CH_2), 3.06 (t, *J* = 7.3 Hz, 2 H, CH_2). The compound could not be further purified as there was less than 1 mg of the compound present.

Treatment of *N*-Phenyl-2-vinylaniline (92) with Et_3SiH/KO^{t}Bu (Fast Addition)

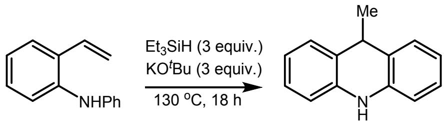

92 **30**, 8 %

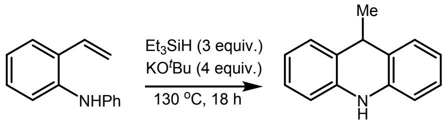

92 **30**, trace

This reaction was carried out according to General Procedure B from *N*-phenyl-2-vinylaniline **92** (98 mg, 0.5 mmol, 1 equiv) with Et_3SiH (0.24 mL, 1.5 mmol, 3 equiv.) and KO^{t}Bu [(168 mg, 1.5 mmol, 3 equiv.) or (224 mg, 2 mmol, 4 equiv.). With three equivalents of KO^{t}Bu, purification by column chromatography afforded 9-methyl-9,10-dihydroacridine **30** as a white solid, with data consistent with those reported above (8 mg, 8 %). With four equivalents of KO^{t}Bu, a complex mixture of products was produced from which **30** was detected by ^{1}H NMR and GCMS (trace amount). The major products in each case appear to be polymerisation products as judged by the broad nature of the ^{1}H NMR spectrum.

Treatment of *N*-Phenyl-2-vinylaniline (92) with Et_3SiH/KO^{t}Bu (Slow Addition)

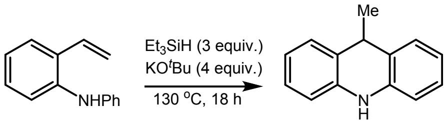

92 **30**, 36 %

In a glovebox under nitrogen, Et_3SiH (0.24 mL, 1.5 mmol, 3 equiv.) and KO^{t}Bu (224 mg, 2 mmol, 4 equiv.) were sealed in a microwave vial equipped with a stirrer bar. The vial was removed and heated to 130 °C behind a shield. *N*-phenyl-2-vinylaniline **92** (98 mg, 0.5 mmol, 1 equiv). was added dropwise by needle and syringe over 1 h, and the resulting mixture was stirred for 18 h at this temperature. After cooling to room temperature, the mixture was diluted with water (50 mL) and extracted into diethyl ether (3 x 50 mL). The combined organic layers were dried over Na_2SO_4, filtered and concentrated under reduced pressure. Purification by column chromatography (hexane:ethyl acetate, 100:0 → 9:1), followed by recrystallisation from hexane afforded 9-methyl-9,10-dihydroacridine **30** as a white solid, with data consistent with those reported above (35 mg, 36 %).

Treatment of *N*-Phenyl-2-vinylaniline (92) with Et_3SiH/NaOtBu (Slow Addition)

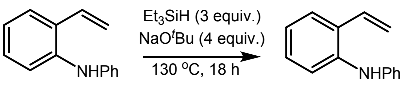

92 **92**, 77 %

In a glovebox under nitrogen, Et_3SiH (0.24 mL, 1.5 mmol, 3 equiv.) and NaO^{t}Bu (192 mg, 2 mmol, 4 equiv.) were sealed in a microwave vial equipped with a stirrer bar. The vial was

removed and heated to 130 °C behind a shield. *N*-phenyl-2-vinylaniline **92** (95 mg, 0.5 mmol, 1 equiv). was added dropwise by needle and syringe over 1 h, and the resulting mixture was stirred for 18 h at this temperature. After cooling to room temperature, the mixture was diluted with water (50 mL) and extracted into diethyl ether (3 x 50 mL). *N*-phenyl-2-vinylaniline **92** was obtained as the sole product without any further purification (73 mg, 77%).

Treatment of 3-Methyl-1-phenyl-1*H*-indole (23) with 0.5 eq. Et_3SiH/ 0.5 eq. KO^tBu

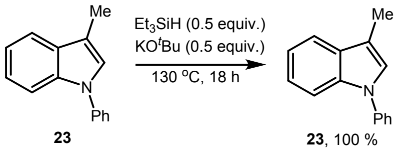

This reaction was carried out according to General Procedure B from 3-methyl-1-phenyl-1*H*-indole **23** (104 mg, 0.5 mmol, 1 equiv.) with Et_3SiH (0.04 mL, 0.25 mmol, 0.5 equiv.) and KO^tBu (28 mg, 0.25 mmol, 0.5 equiv.). 3-Methyl-1-phenyl-1*H*-indole starting material **23** was recovered without any further purification (104 mg, 100%) with data consistent with those reported above.

Treatment of 3-Methyl-1-phenyl-1*H*-indole (23) with 0.5 eq. Et_3SiH/ 3.0 eq. KO^tBu

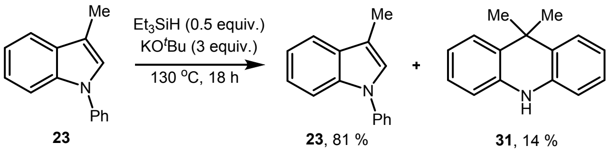

This reaction was carried out according to General Procedure B from 3-methyl-1-phenyl-1*H*-indole **23** (104 mg, 0.5 mmol, 1 equiv.) with Et_3SiH (0.04 mL, 0.25 mmol, 0.5 equiv.) and KO^tBu (168 mg, 1.5 mmol, 3 equiv.). The reaction afforded 3-methyl-1-phenyl-1*H*-indole starting material **23** (81%) and 9,9-dimethyl-9,10-dihydroacridine **31** (14 %). The yields were calculated by addition of 1,3,5-trimethoxybenzene (17 mg, 0.101 mmol, 20.2 mol%) as an internal standard to the sample of the mixture. The signal of the three methoxy groups (3.80 ppm) was integrated to 9 integral units, and the relative intensities of the methyl groups of **23** (2.43 ppm, 12.08 integral units) and **31** (1.62 ppm, 4.02 integral units) were used to calculate yields. For **23**, yield = [12.08/3] x 20.2 = 81 %. For **31**, yield = [4.02/6] x 20.2 = 14 %.

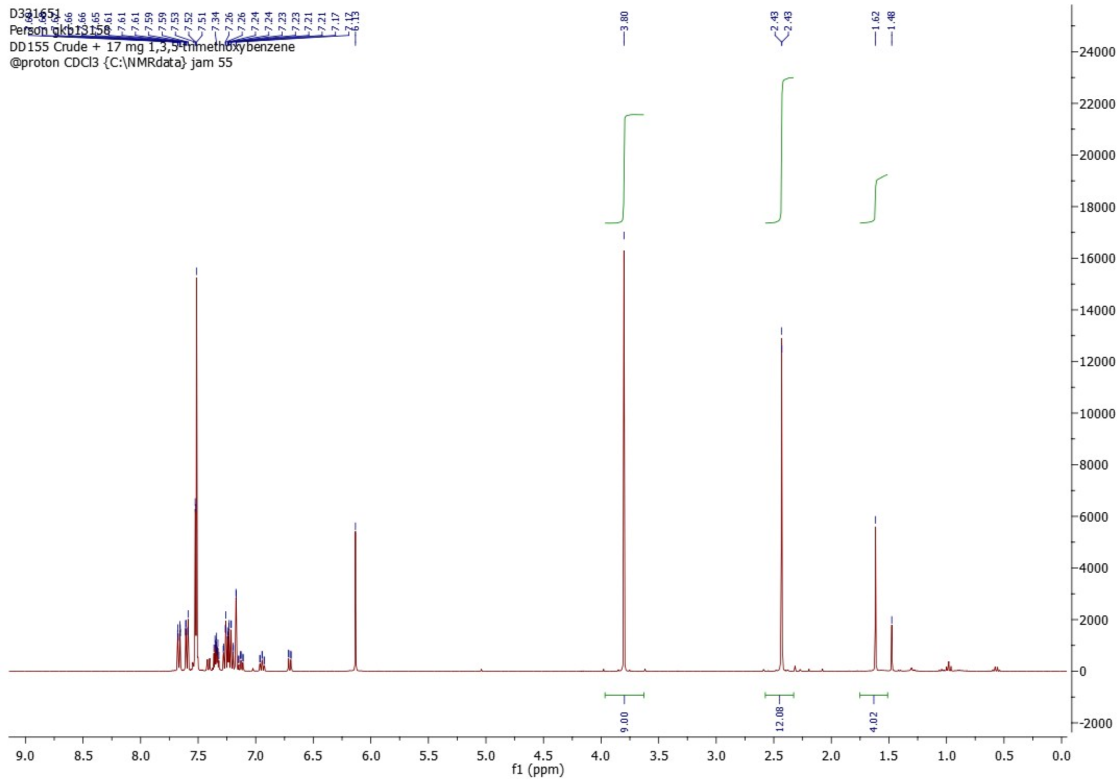

Figure S10 *Determination of the Yields of **23** and **31** by the Addition of an Internal Standard to the ^1H NMR*

Further Mechanistic Studies

Treatment of 1-Phenyl-1*H*-indole (**22**) with **99**, $Me_3SiSiMe_3$ and KO^tBu

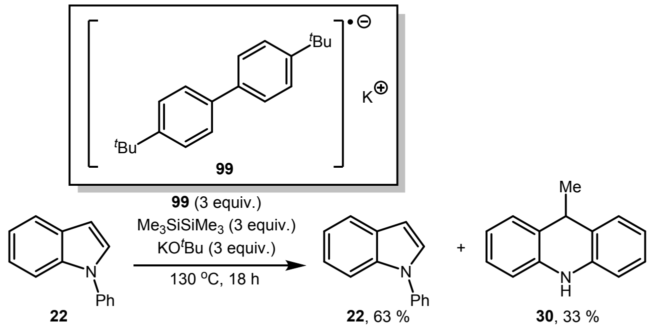

The preparation of **99** was carried out according to a literature procedure.[44] Potassium metal was washed with hexane under argon atmosphere to remove mineral oil. The hexane was removed by needle and syringe and quenched with isopropanol, and the potassium metal was then transferred to the glovebox where the residual hexane was removed under vacuum. The oxide layer was removed from potassium in the glovebox by scraping with a knife, exposing a fresh metallic surface. Potassium metal (177 mg, 4.5 mmol, 1 equiv.) was then added to a solution of 4,4’-di-tert-butylbiphenyl 789 (1.2 g, 4.5 mmol, 1 equiv.) in THF (180 mL). The resulting solution was stirred under nitrogen until an intense green colour formed (~4 h). THF was then evaporated under vacuum in the glovebox, affording a green/white solid (**99**) which was used immediately without further purification. Compound **99** (458 mg, 1.5 mmol, 3 equiv.),

1-phenyl-1*H*-indole **22** (97 mg, 0.5 mmol, 1 equiv.), KO^{t}Bu (168 mg, 1.5 mmol, 3 equiv.) and hexamethyldisilane (0.31 mL, 1.5 mmol, 3 equiv.) were added to a pressure tube equipped with a stirrer bar in the glovebox under nitrogen. The tube was then sealed, removed from the glovebox and heated at 130 °C for 18 h behind a shield. After cooling to room temperature, the mixture was diluted with water (50 mL) and extracted into diethyl ether (3 x 50 mL). The combined organic layers were dried over Na_2SO_4, filtered and concentrated under reduced pressure. Purification by column chromatography afforded 4,4'-di-*tert*-butylbiphenyl as a white solid with data matching the commercial sample, followed by 1-phenyl-1*H*-indole **22** as a colourless oil (61 mg, 63 %), followed by 9-methyl-9,10-dihydroacridine **30** as a yellow solid (32 mg, 33 %). The data for these compounds are consistent with those reported above.

Treatment of 1-Phenyl-1*H*-indole (22) with 99 and KO^{t}Bu

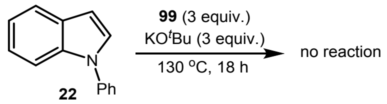

22

Compound **99** (458 mg, 1.5 mmol, 3 equiv.), 1-phenyl-1*H*-indole **22** (97 mg, 0.5 mmol, 1 equiv.) and KO^{t}Bu (168 mg, 1.5 mmol, 3 equiv.) were added to a pressure tube equipped with a stirrer bar in the glovebox under nitrogen. The tube was then sealed, removed from the glovebox and heated at 130 °C for 18 h behind a shield. After cooling to room temperature, the mixture was diluted with water (50 mL) and extracted into diethyl ether (3 x 50 mL). The combined organic layers were dried over Na_2SO_4, filtered and concentrated under reduced pressure. No reaction was found to have taken place by ^{1}H NMR and TLC analysis of the crude reaction mixture.

Treatment of 1-Phenyl-1*H*-indole (22) with $Me_3SiSiMe_3$ and KO^{t}Bu

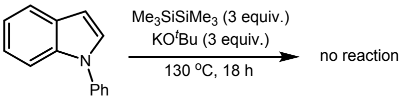

22

Hexamethyldisilane (0.31 mL, 1.5 mmol, 3 equiv.), 1-phenyl-1*H*-indole **22** (97 mg, 0.5 mmol, 1 equiv.) and KO^{t}Bu (168 mg, 1.5 mmol, 3 equiv.) were added to a pressure tube equipped with a stirrer bar in the glovebox under nitrogen. The tube was then sealed, removed from the glovebox and heated at 130 °C for 18 h behind a shield. After cooling to room temperature, the mixture was diluted with water (50 mL) and extracted into diethyl ether (3 x 50 mL). The combined organic layers were dried over Na_2SO_4, filtered and concentrated under reduced pressure. No reaction was found to have taken place by ^{1}H NMR and TLC analysis of the crude reaction mixture.

Treatment of 1-Phenyl-3-methyl-1*H*-indole (23) with Et_3SiD/KO^{t}Bu

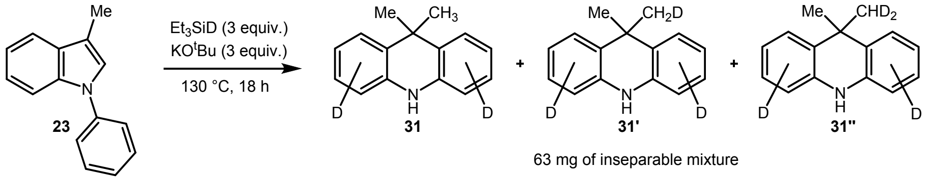

1-Phenyl-3-methyl-1*H*-indole **23** (104 mg, 0.5 mmol, 1 equiv.), Et_3SiD (0.24 mL, 1.5 mmol, 3 equiv.) and potassium *tert*-butoxide (168 mg, 1.5 mmol, 3 equiv.) were sealed in a pressure tube in a glovebox under nitrogen. The tube was removed and heated at 130 °C for 18 h behind a safety shield. After cooling to room temperature, the mixture was diluted with water (50 mL) and extracted into diethyl ether (3 x 50 mL). The combined organic layers were dried over Na_2SO_4, filtered and concentrated under reduced pressure. Purification by column chromatography (hexane:diethyl ether, 100:0 → 98.5:1.5) afforded a mixture of polydeuterated 9,9-dimethyl-9,10-dihydroacridine products, including **31**, **31'** and **31''** as a white solid (63 mg). ***m/z*** **(EI):** 209.1 ($C_{15}H_{15}N^+$, M^+), 210.1 ($C_{15}H_{14}DN^+$, M^+), 210.9 ($C_{15}H_{13}D_2N^+$, M^+), 211.1 ($C_{15}H_{13}D_2N^+$, M^+), 211.9 ($C_{15}H_{12}D_3N^+$, M^+), 212.1 ($C_{15}H_{12}D_3N^+$, M^+), 212.9 ($C_{15}H_{11}D_4N^+$, M^+), 213.1 ($C_{15}H_{11}D_4N^+$, M^+), 214.0 ($C_{15}H_{10}D_5N^+$, M^+). **^{13}C NMR** data provided evidence for the presence of CH_3, CH_2D and CHD_2 groups in the mixture of products. Three different methyl carbon environments were present by $^{13}C\{^1H\}$ NMR – a singlet (CH_3), a triplet (CH_2D) and a quintet (CHD_2). This was also confirmed by $^{13}C\{^1H, ^2H\}$ NMR where three different singlet peaks were observed. Evidence for deuteration of the aromatic carbons also exists by ^{2}H NMR (NMR data are shown below).

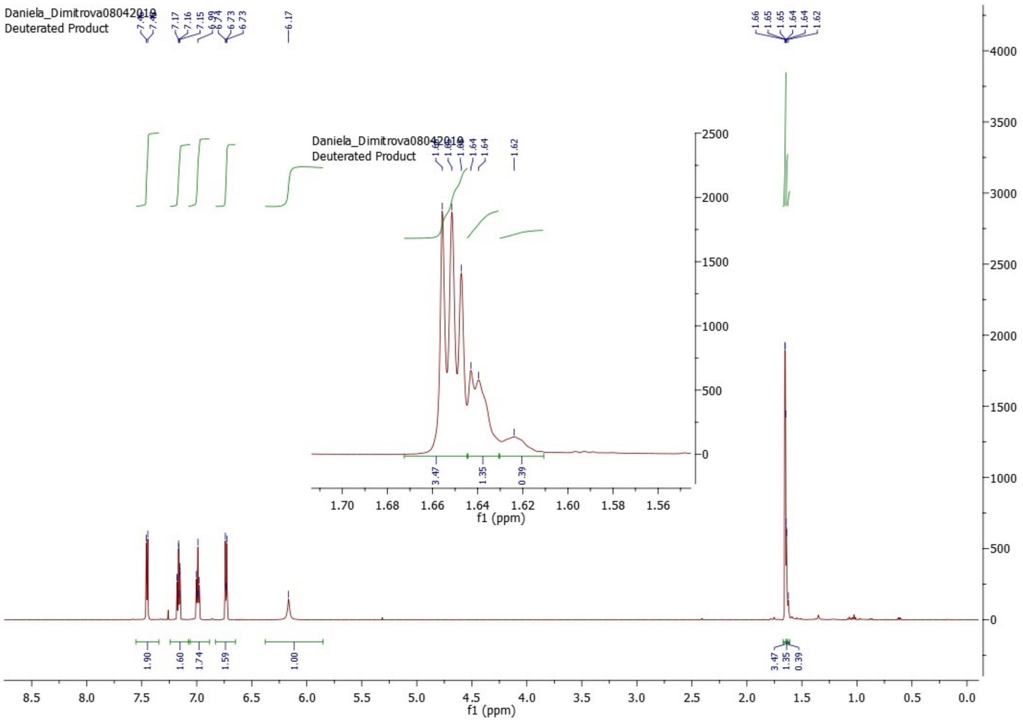

*Figure S11 ^{1}H NMR of the mixture of **30**, **30'** and **30''***

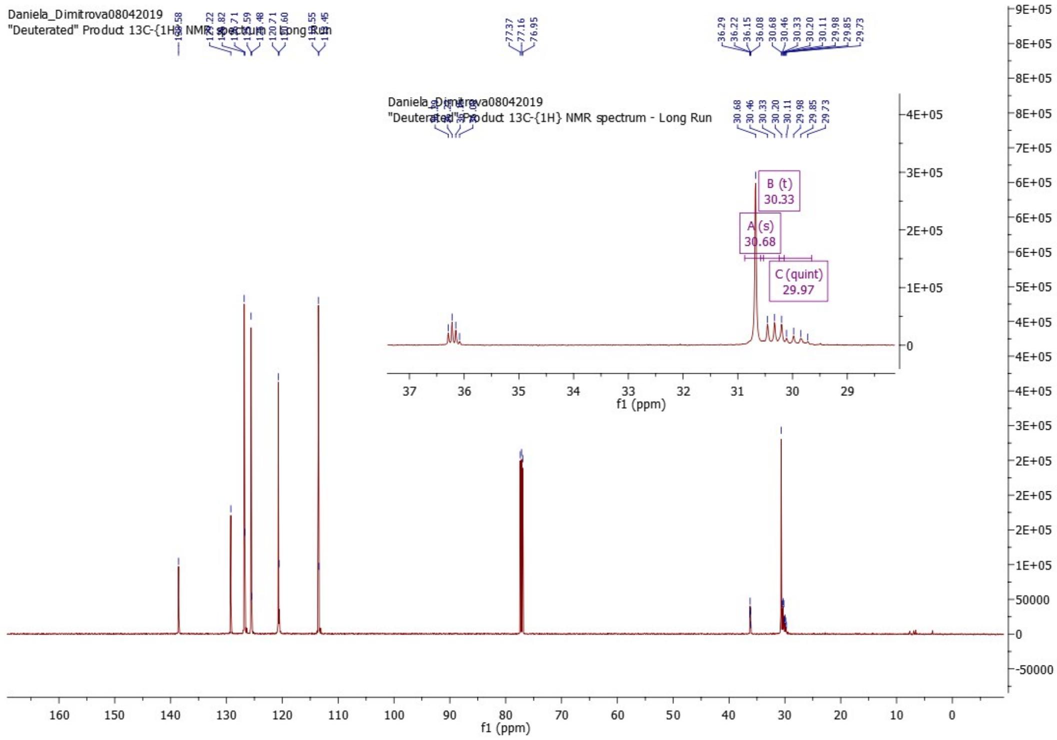

*Figure S12 $^{13}C\{^1H\}$ NMR of the mixture of **30**, **30'** and **30''***

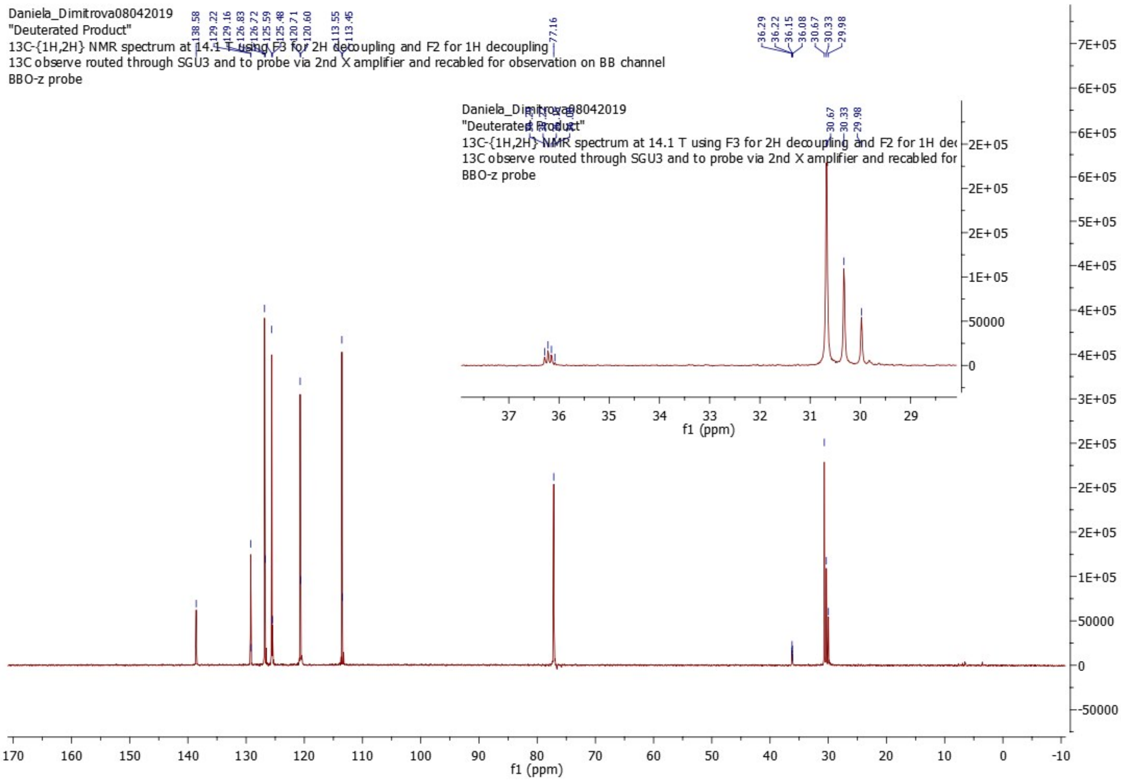

*Figure S13 $^{13}C\{^1H, ^2H\}$ NMR of the mixture of **30**, **30'** and **30''***

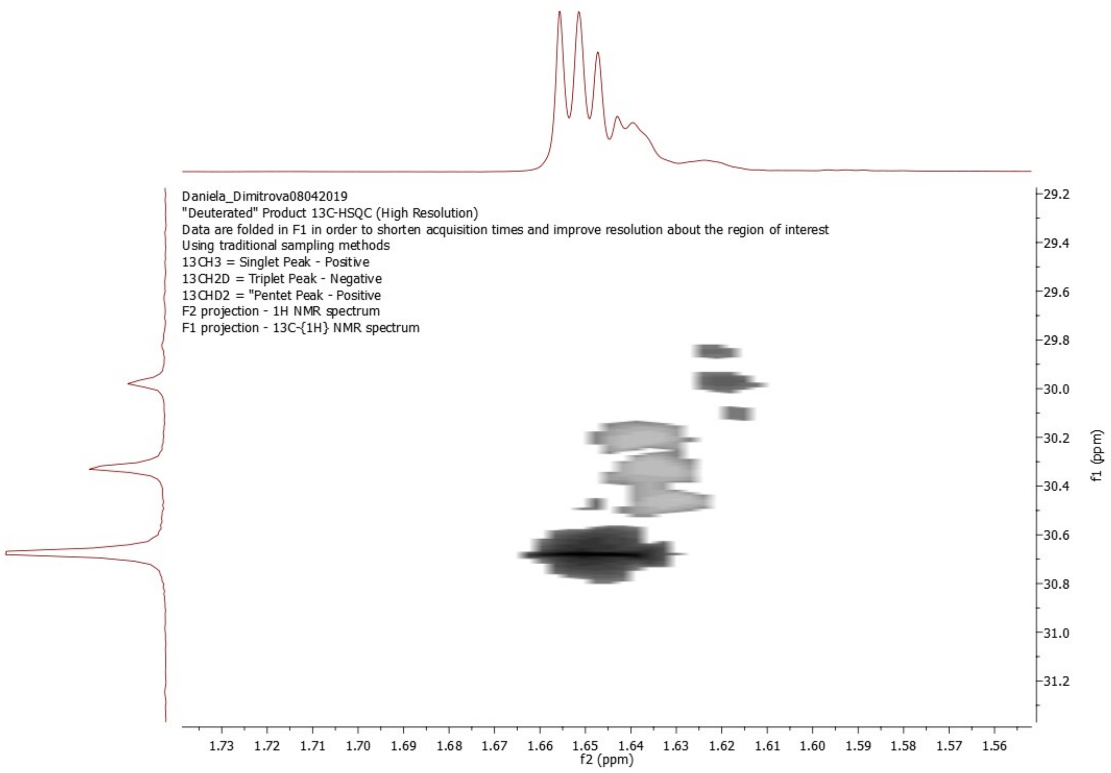

*Figure S14 HSQC [^{1}H, ^{13}C{^{1}H, ^{2}H}] NMR of the mixture of **30**, **30'** and **30''***

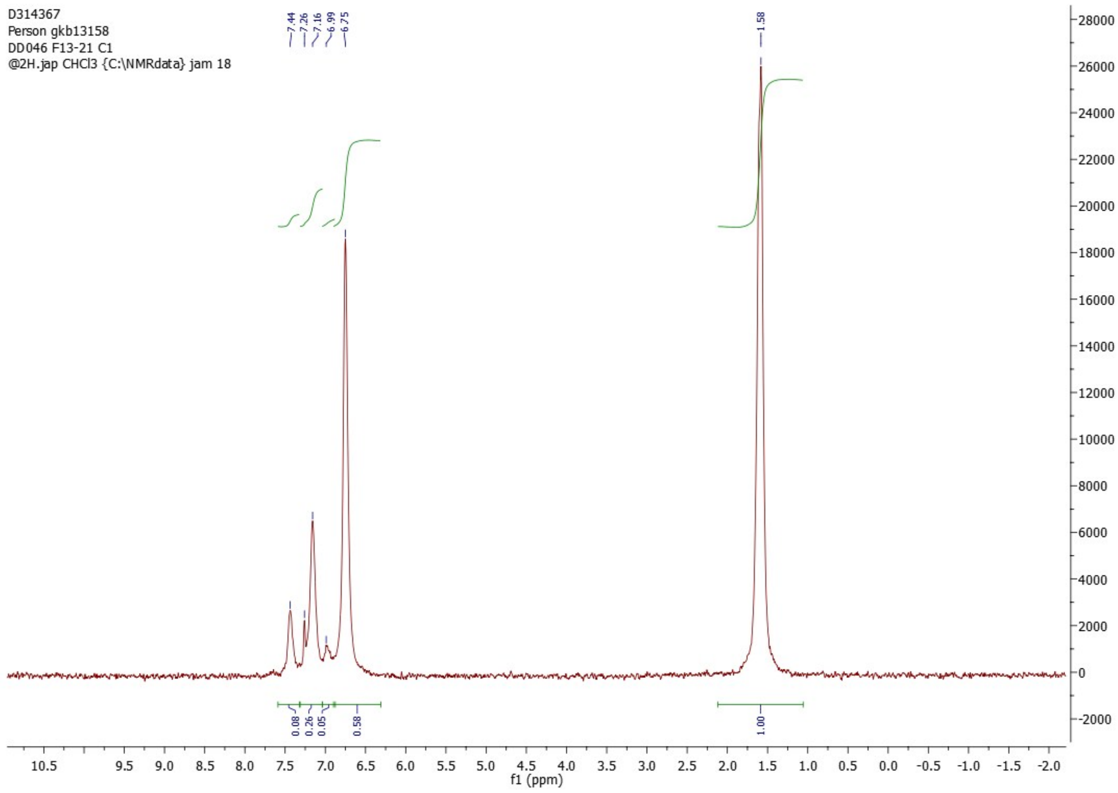

*Figure S15 ^{2}H NMR of the mixture of **30**, **30'** and **30''***

Treatment of 9,9-Dimethyl-9,10-dihydroacridine (**31**) with Et_3SiD/KO^{t}Bu

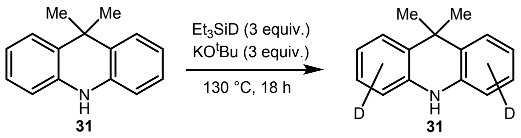

100 mg of inseparable mixture

9,9-Dimethyl-9,10-dihydroacridine indole **31** (105 mg, 0.5 mmol, 1 equiv.), Et_3SiD (0.24 mL, 1.5 mmol, 3 equiv.) and KO^{t}Bu (168 mg, 1.5 mmol, 3 equiv.) were sealed in a pressure tube in a nitrogen-filled glovebox. The reaction mixture was stirred at 130 ℃ for 18 h behind a blast shield. The contents of the pressure tube were then cooled to room temperature and diluted with water (50 mL) and Et_2O (50 mL). The aqueous phase was separated and further washed with Et_2O (2 x 50 mL). The organic phases were combined, dried over Na_2SO_4, filtered and concentrated. Purification by column chromatography (hexane:diethyl ether, 100:0 → 98.5:1.5) afforded a mixture of polydeuterated 9,9-dimethyl-9,10-dihydroacridine products as a white solid (100 mg). **Mp** = 92-94 °C. **ATR-IR** v_{max} (neat)/cm^{-1} 3356, 2957, 1605, 1578, 1474, 1452, 1314, 1242, 1092, 1036, 885, 743, 689, 656. ***m/z* (EI):** 209.1 ($C_{15}H_{15}N^+$, M^+), 210.1 ($C_{15}H_{14}DN^+$, M^+), 211.1 ($C_{15}H_{13}D_2N^+$, M^+), 212.1 ($C_{15}H_{12}D_3N^+$, M^+). O*rtho* deuteration relative to the NH group was predominant. The order of deuteration followed: *ortho* > *meta* > *para* to the NH group (NMR data are shown below).

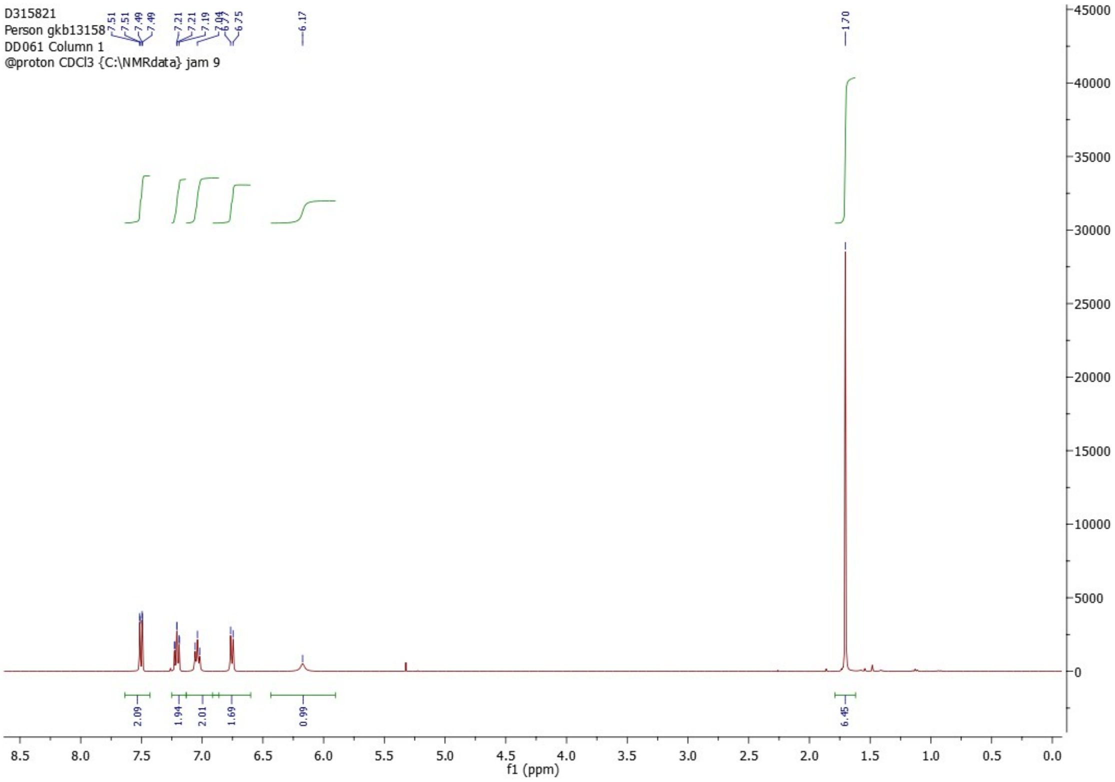

Figure S16 ^{1}H NMR spectrum of the mixture of isotopomers of **30**

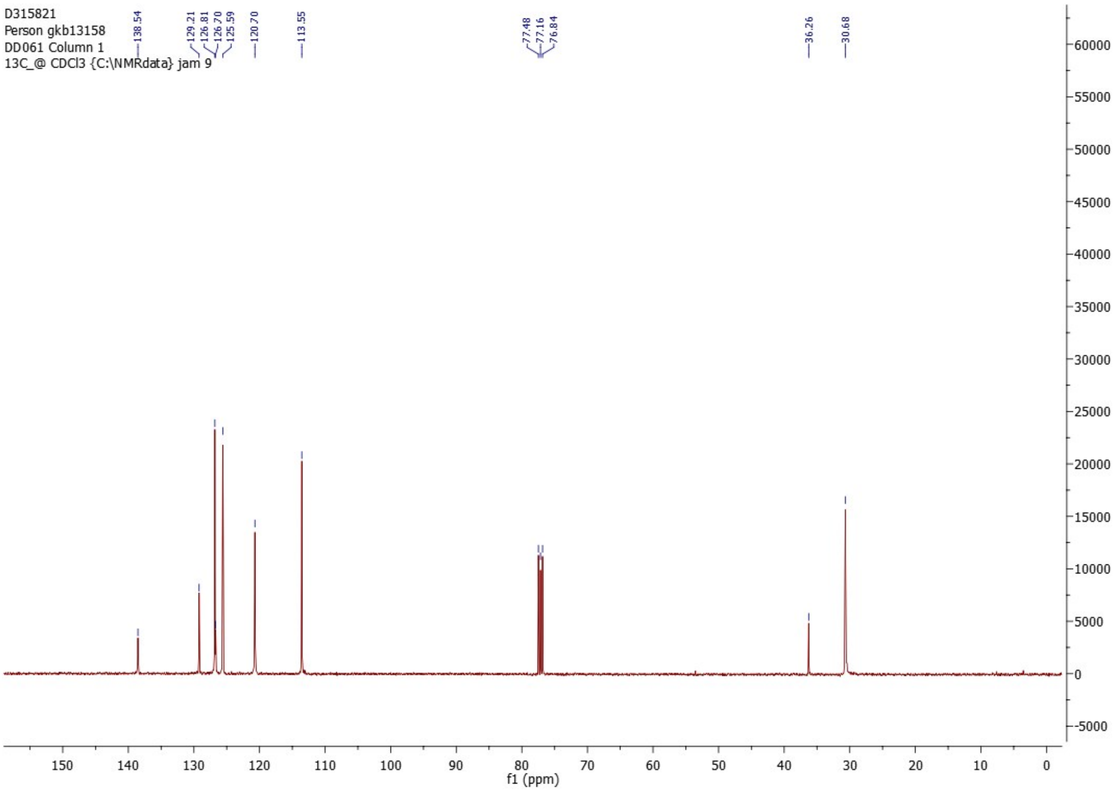

Figure S17 $^{13}C\{^1H\}$ *NMR* spectrum of the mixture of isotopomers of **30**

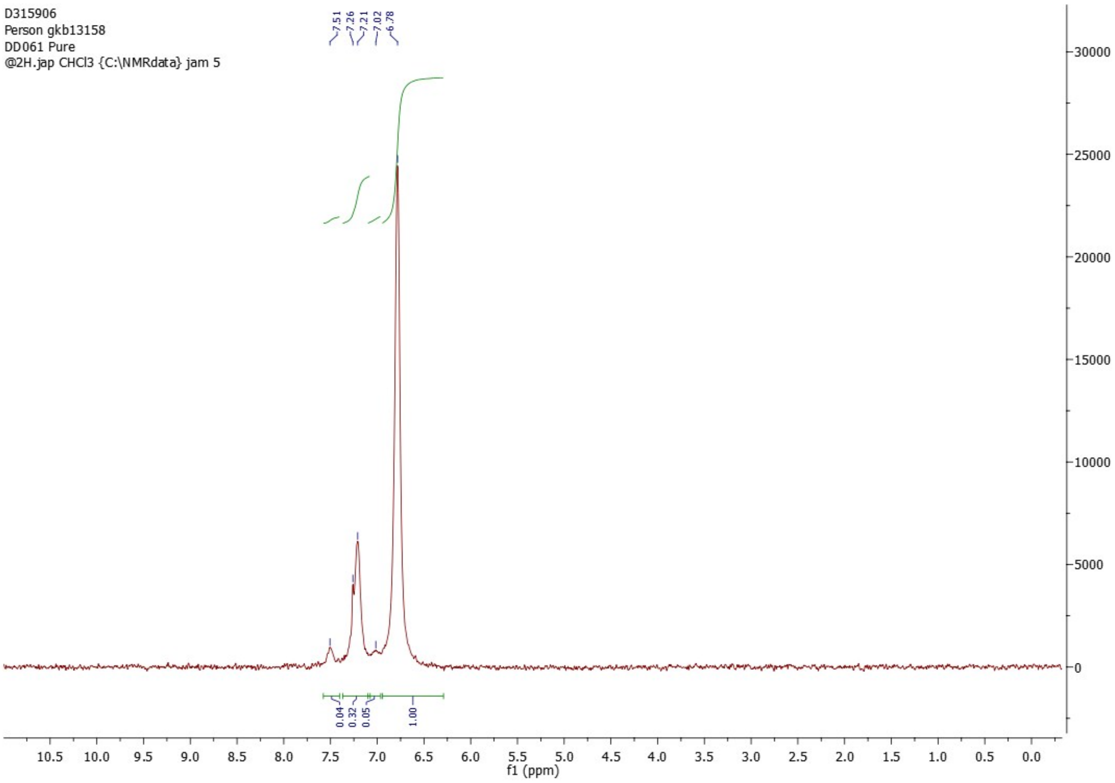

Figure S18 ^2H *NMR* spectrum of the mixture of isotopomers of **30**

Treatment of 3-Methyl-1-phenyl-1*H*-indole (**23**) with Et_3SiH/KO^{t}Bu with the Removal of H_2

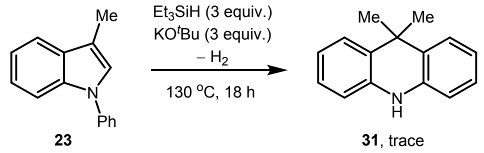

In a microwave vial in the glovebox was added triethylsilane (0.24 mL, 1.5 mmol, 3 equiv.) and potassium *tert*-butoxide (168 mg, 1.5 mmol, 3 equiv.). The tube was sealed, removed from the glovebox, and heated at 130 °C for 1 h behind a shield. After this time, the hydrogen gas generated was removed by purging the vial with argon. 3-Methyl-1-phenyl-1*H*-indole **23** (104 mg, 0.5 mmol, 1 equiv.) was then added and the mixture stirred at 130 °C for 18 h. After cooling to room temperature, the mixture was diluted with water and extracted into diethyl ether, dried over Na_2SO_4, filtered and concentrated under reduced pressure. Purification by column chromatography (hexane:ethyl acetate, 100:0 ♑ 90:10) afforded starting material **23**, with data consistent with those reported above (76 mg, 73 %) and 9,9-dimethyl-9,10-dihydroacridine **31**, which was detected by GCMS, with data consistent with those reported earlier (<1 mg).

Treatment of 3-Methyl-1-phenyl-1*H*-indole (23) with Et_3SiH/KO^{t}Bu with the Replacement of H_2 with D_2

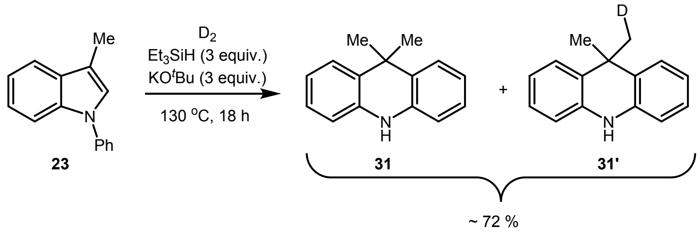

To a microwave vial in the glovebox was added triethylsilane (0.24 mL, 1.5 mmol, 3 equiv.) and potassium *tert*-butoxide (168 mg, 1.5 mmol, 3 equiv.). The vial was sealed and removed from the glovebox and stirred at 130 °C for 1 h behind a shield. After cooling to room temperature, the hydrogen gas generated was removed under vacuum and replaced with deuterium gas from a balloon. The mixture was then heated at 130 °C for 18 h behind a shield. After cooling to room temperature, the atmosphere was removed under vacuum and replaced with argon. The tube was then opened, and the mixture was diluted with water and extracted into diethyl ether, dried over a hydrophobic frit and concentrated under reduced pressure. Purification by column chromatography (hexane:ethyl acetate, 100:0 ♑ 90:10) afforded starting material **23** with data consistent with those reported previously (6 mg, 6 %), and an inseparable mixture of 9,9-dimethyl-9,10-dihydroacridine **31** and 9-methyl-9-(methyl-*d*)-9,10-dihydroacridine **31'** (75 mg, ~72 %). This dihydroacridine mixture was found to quickly oxidise to a radical species in air, and this was reduced by dissolving the sample in DCM and washing with saturated potassium iodide solution. The organic layer was dried over a hydrophobic frit and concentrated under reduced pressure. **^{1}H-NMR** (400 MHz, $CDCl_3$) 1.59 - 1.64 (m, 2 x CH_3 + CH_3 and CH_2D), 6.15 (br. s., 1 H, NH), 6.72 (dd, *J* = 7.9, 1.1 Hz, 2 H, 2 x ArH), 6.96 (td, *J* = 7.5, 1.3 Hz, 2 H, 2 x ArH), 7.14 (td, *J* = 7.6, 1.4 Hz, 2 H, 2 x ArH), 7.42 (dd, *J* = 7.8, 1.0 Hz, 2 H, 2 x ArH). **^{13}C-NMR** (101 MHz,

$CDCl_3$) 30.2 (t, *J* = 19.3 Hz), 30.5, 36.1, 36.2, 113.4, 120.6, 125.5, 126.7, 129.1, 138.4. **$^2D\{^1H\}$-NMR** (61 MHz, $CHCl_3$) 1.63 (s, CH_2D), 6.77 (s, ArD). ***m/z* (EI)** 211.1 ($[M+2D]^+$, 2), 210.1 ($[M+D]^+$, 6), 209.2 (M^+, 10), 208.1 (2), 195.1 (41), 194.1 (100), 193.1 (15), 192.1 (12), 191.1 (7).

Treatment of 3-Methyl-1-phenyl-1*H*-indole (23) with Different Silanes

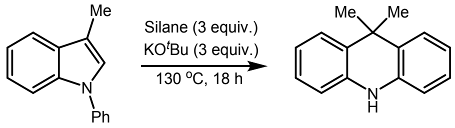

23 **31**

3-Methyl-1-phenyl-1*H*-indole (104 mg, 0.5 mmol, 1 equiv), the appropriate silane [either Et_3SiH (0.24 mL, 1.5 mmol, 3 equiv.), Me_2PhSiH (0.23 mL, 1.5 mmol, 3 equiv.), $MePh_2SiH$ (0.30 mL, 1.5 mmol, 3 equiv.) or Ph_3SiH (391 mg, 1.5 mmol, 3 equiv.)] and potassium *tert*-butoxide (168 mg, 1.5 mmol, 3 equiv.) were sealed in a pressure tube in a glovebox under nitrogen. The tube was removed and heated at 130 °C for 18 h behind a safety shield. After cooling to room temperature, the mixture was diluted with water (50 mL) and extracted into diethyl ether (3 x 50 mL). The combined organic layers were dried over Na_2SO_4, filtered and concentrated under reduced pressure. Purification by column chromatography (hexane:diethyl ether, 9:1 ♑ 4:1) afforded 9,9-dimethyl-9,10-dihydroacridine **31** as a white solid, in the yields shown in Table 1. The data for this compound are consistent with those reported above.

*Table 1 - Yields of **31***

Entry	Silane	Mass of 31	Yield of 31
1	Et_3SiH	81 mg	77 %
2	Me_2PhSiH	69 mg	66 %
3	$MePh_2SiH$	71 mg	68 %
4	Ph_3SiH	68 mg	65 %

Treatment of 1,3-Diphenylindole (26) with K/KO^{t}Bu

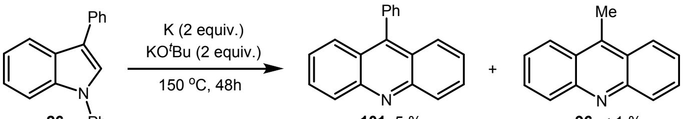

26 Ph **101**, 5 % **96**, < 1 %

To a pressure tube equipped with a stirrer bar, under a nitrogen atmosphere inside a glovebox, was added 1,3-diphenyl-1*H*-indole (1 equiv.), potassium (2 equiv.) and potassium *tert*-butoxide (2 equiv.). The reagents were divided amongst 8 pressure tubes as shown in Table S2. The pressure tubes were sealed and removed from the glovebox. The reactions were stirred at 150 °C for 21 h behind a safety shield. After cooling to room temperature, the reactions were quenched with isopropanol, followed by H_2O when no more effervescence was be observed. The reaction mixtures were brought to neutral pH using HCl (1M) and NaOH (10% w/v). The

reaction mixtures were combined and extracted with Et_2O. The combined organic layers were dried over Na_2SO_4, filtered and concentrated under reduced pressure.

Table S2

Entry	26/mg	26/mmol	K/mg	K/mmol	KO*t*Bu (mg)	KO*t*Bu/mmol
1	250	0.93	76	1.94	211	1.88
2	249	0.92	75	1.92	215	1.92
3	252	0.94	75	1.92	211	1.88
4	252	0.94	73	1.87	213	1.90
5	246	0.91	75	1.92	211	1.89
6	254	0.94	73	1.87	212	1.89
7	249	0.92	77	1.97	216	1.92
8	252	0.94	73	1.87	215	1.92

Purification by chromatography (hexane:EtOAc, 5:1) afforded a mixture of 9-phenylacridine and 9-methylacridine (325 mg). A portion of the mixture (223.6 mg) was further purified by chromatography (PhMe/DCM/EtOAc, 70:30:10) and crystallization, affording 9-phenylaciridine **101** as an orange solid (62 mg,). The yield was worked out based on ratios as follows:

223.6 mg of mixture gave 62 mg 9-phenylacridine

So 325 mg would contain 90 mg 9-phenylacridine

Theoretical Yield of 9-phenylacridine **101** = 1.900 g

Yield of **101** = 100(0.090 g/1.900 g) = 4.7 %

mp = 178 – 180 ˚C (lit mp = 182 – 184 ˚C).[45] **^{1}H-NMR** (400 MHz, $CDCl_3$) 8.35 (d, *J* = 7.7 Hz, ArH, 2 H), 7.82 – 7.78 (m, ArH, 2 H), 7.72 (d, *J* = 8.7 Hz, ArH, 2 H), 7.64 – 7.59 (m, ArH, 3 H), 7.46 –7.43 (m, ArH, 4 H). **^{13}C-NMR** (101 MHz, $CDCl_3$) 148.7, 147.2, 135.9, 130.4, 129.9, 129.5, 128.4, 128.3, 126.8, 125.5, 125.1. **ATR-IR** ν_{max} (neat)/cm^{-1} 3059, 1609, 1555, 1535, 1514, 1476, 1458, 1414, 1356, 1161, 1134, 1013, 856, 750, 704, 648, 608. ***m/z* (EI)** 255.1 (M^+, 100), 226.1 (9), 126.1 (13). The data for this compound are consistent with those reported in the literature.[45] Also detected was 9-methylacridine **96**, with data consistent with those reported above (< 1 mg, < 1 %).

Treatment of *N*-phenyl-2-vinylaniline (92) with Et_3SiD/KO*t*Bu

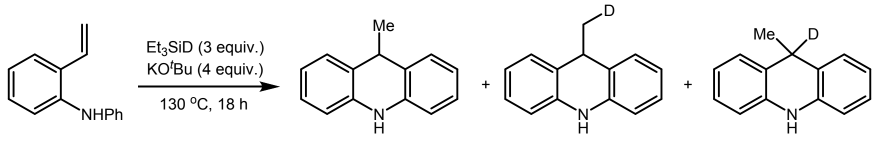

92 **30** **30'** **30''**

To a microwave vial in the glovebox was added Et_3SiD (0.24 mL, 1.5 mmol, 3 equiv.), and KO^tBu (224 mg, 2 mmol, 4 equiv.). The vial was sealed and removed from the glovebox and heated at 130 °C behind a shield. *N*-phenyl-2-vinylaniline **92** (98 mg, 0.5 mmol, 1 equiv.) was added dropwise over 1 h, and the resulting mixture was then stirred at this temperature for 18 h. After cooling to room temperature, the mixture was diluted with water (50 mL) and extracted into diethyl ether (3 x 50 mL). The combined organic layers were dried over Na_2SO_4, filtered and concentrated under reduced pressure. Purification by column chromatography (hexane:ethyl acetate, 100:0 ♑ 90:10) afforded an inseparable mixture of 9-methyl-9,10-dihydroacridine **30**, 9-(methyl-*d*)-9,10-dihydroacridine **30'**, and 9-methyl-9,10-dihydroacridine-9-*d* **30''** (7 mg, ~ 7 %). **^{1}H-NMR** (400 MHz, $CDCl_3$) 1.34 - 1.37 (m, overlapping signals for CH_3 and CH_2D), 4.12 (q, *J* = 6.8 Hz, 1 H, CH), 6.04 (br. s., 1 H, NH), 6.72 (dd, *J* = 8.1, 1.0 Hz, 2 H, 2 x ArH), 6.90 (td, *J* = 7.4, 1.0 Hz, 2 H, 2 x ArH), 7.11 (td, *J* = 7.6, 1.4 Hz, 2 H, 2 x ArH), 7.18 (d, *J* = 7.5 Hz, 2 H, 2 x ArH). **^{13}C-NMR** (101 MHz, $CDCl_3$) 26.1 (t, *J* = 20.0 Hz), 26.4 (s), 36.6 (s), 36.7 (s), 113.4 (s), 120.8 (s), 125.7 (s), 126.8 (s), 128.1 (s), 139.0 (s). **^{2}D{^{1}H}-NMR** (61 MHz, $CHCl_3$) 1.37 (s), 4.10 (s). ***m/z* (CI)** 197.2 $[M+H]^+$ (one deuterium atom), 196.1 $[M+H]^+$ (no deuterium atoms), 195.1 $[M+H]^+$ and 194.2 $[M+H]^+$. Presumably, *m/z* = 195.1 and 194.1 arise from partial oxidation of **30**, **30'**, and **30''**.

References

1 Frisch, M. J.; Trucks, G. W.; Schlegel, H. B.; Scuseria, G. E.; Robb, M. A.; Cheeseman, J. R.; Scalmani, G.; Barone, V.; Petersson, G. A.; Nakatsuji, H.; Li, X.; Caricato, M.; Marenich, A.; Bloino, J.; Janesko, B. G.; Gomperts, R.; Mennucci, B.; Hratchian, H. P.; Ortiz, J. V.; Izmaylov, A. F.; Sonnenberg, J. L.; Williams-Young, D.; Ding, F.; Lipparini, F.; Egidi, F.; Goings, J.; Peng, B.; Petrone, A.; Henderson, T.; Ranasinghe, D.; Zakrzewski, V. G.; Gao, J.; Rega, N.; Zheng, G.; Liang, W.; Hada, M.; Ehara, M.; Toyota, K.; Fukuda, R.; Hasegawa, J.; Ishida, M.; Nakajima, T.; Honda, Y.; Kitao, O.; Nakai, H.; Vreven, T.; Throssell, K.; Montgomery, J. A. Jr.; Peralta, J. E.; Ogliaro, F.; Bearpark, M.; Heyd, J. J.; Brothers, E.; Kudin, K. N.; Staroverov, V. N.; Keith, T.; Kobayashi, R.; Normand, J.; Raghavachari, K.; Rendell, A.; Burant, J. C.; Iyengar, S. S.; Tomasi, J.; Cossi, M.; Millam, J. M.; Klene, M.; Adamo, C.; Cammi, R.; Ochterski, J. W.; Martin, R. L.; Morokuma, K.; Farkas, O.; Foresman, J. B.; Fox, D. J. Gaussian, Inc., Wallingford CT, 2016, Gaussian, Inc., Wallingford CT, 2009.

2 Cramer, C. J.; Truhlar, D. G. *Chem. Rev*., **1999**, *99*, 2161–2200.

3 Cramer, C. J.; Truhlar, D. G., *Phys. Chem. Chem. Phys*., **2009**, *11*, 10757–10816.

4 Rassolov, V. A.; Ratner, M. A. ; Pople, J. A. ; Redfern, P. C. ; Curtiss, L. A. *J. Comput. Chem.,* **2001**, *22*, 976–984.

5 Frisch, M. J.; Pople, J. A.; Binkley, J. S. *J. Chem. Phys*., **1984**, *80*, 3265–3269.

6 McLean, A. D.; Chandler, G. S. *J. Chem. Phys*., **1980**, *72*, 5639–5648.

7 Cossi, M.; Rega, N.; Scalmani, G.; Barone, V. *J. Comput. Chem*. **2003**, *24*, 669–681.

8 Cossi, M.; Barone, V. *J. Chem. Phys*. **1998**, *109*, 6246–6254.

9 Hong, X.; Tan, Q.; Liu, B.; Xu, B. *Angew. Chem. Int. Ed*. **2017**, *56*, 3961–3965.

10 Kandukuri, S. R.; Schiffner, J. A.; Oestreich, M. *Angew. Chem. Int. Ed*. **2012**, *51*, 1265–1269.

11 Wang, H.; Li, Y.; Sun, F.; Feng, Y.; Jin, K.; Wang, X. *J. Org. Chem*. **2008**, *73*, 8639–8642.

12 Ito, Y.; Ueda, M.; Takeda, N.; Miyata, O. *Chem. Eur. J*. **2016**, *22*, 2616–2619.

13 Gehrmann, T.; Fillol, J. L.; Scholl, S. A; Wadepohl, H.; Gade, L. H. *Angew. Chem. Int. Ed*. **2011**, *50*, 5757–5761.

14 Yang, R.; Yu, J. T.; Sun, S.; Zheng, Q.; Cheng, J. *Tetrahedron Lett*. **2017**, *58*, 445–448.

15 Chen, S.; Liao, Y.; Zhao, F.; Qi, H.; Liu, S.; Deng, G.-J. *Org. Lett*. **2014**, *16*, 1618–1621.

16 Willis, M. C.; Brace, G. N.; Findlay, T. J. K.; Holmes, I. P. *Adv. Synth. Catal*. **2006**, *348*, 851–856.

17 Sebastian, I.; Sebastian, B.; Annegret, T.; Kathleen, M.; Lorenz, N.; Matthias, B. *Chem. Eur. J*. **2010**, *16*, 2705–2709.

18 Pawar, G. G.; Wu, H.; De, S.; Ma, D. *Adv. Synth. Catal*. **2017**, *359*, 1631–1636.

19 Cano, R.; Ramón, D. J.; Yus, M. *J. Org. Chem*. **2011**, *76*, 654–660.

20 Marta, R.; Eduardo, A.; Delbrin, A.; Jaime, B.; Gema, D.; Javier, P. *Eur. J. Org. Chem*. **2008**, 3917–3927.

21 Huchet, Q. A.; Kuhn, B.; Wagner, B.; Kratochwil, N. A.; Fischer, H.; Kansy, M.; Zimmerli, D.; Carreira, E. M.; Müller, K. *J. Med. Chem*. **2015**, *58*, 9041–9060.

22 Hock, K. J.; Grimmer, J.; Göbel, D.; Gasaya, G. G. T.; Roos, J.; Maucher, I. V; Kühn, B.; Fettel, J.; Maier, T. J.; Manolikakes, G. *Synthesis* **2017**, *49*, 615–636.

23 Youn, S. W.; Pastine, S. J.; Sames, D. *Org. Lett*. **2004**, *6*, 581–584.

24 Liu, W. B.; Schuman, D. P.; Yang, Y. F.; A. A. Toutov, Y. F.; Liang, Y.; Klare, H. F. T.; Nesnas, N.; Oestreich, M.; Blackmond, D. G.; Virgil, S. C.; Banerjee, S.; Zare, R. N.; Grubbs, R. H.; Houk, K. N.; Stoltz, B. M. *J. Am. Chem. Soc*. **2017**, *139*, 6867–6879.

25 Penoni, A.; Palmisano, G. Zhao,; Y.-L. ; Houk, K. N.; Volkman, J.; Nicholas, K. M. *J. Am. Chem. Soc.* **2009**, *131*, 653–661.

26 Reddy, V.; Anand, R. V. *Org. Lett*. **2015**, *17*, 3390–3393.

27 Peng, C.; Wang, Y.; Liu, L.; Wang, H.; Zhao, J.; Zhu, Q. *Eur. J. Org. Chem.*,**2010**, 818–822.

28 Lessing, T.; Müller, T. J. J*. Chem. Heterocycl. Compd*. **2018**, *54*, 334–338.

29 Gao, J.; Shao, Y.; Zhu, J.; Zhu, J.; Mao, H.; Wang, X.; Lv, X. *J. Org. Chem*. **2014**, *79*, 9000–9008.

30 Zheng, Z.; Dian, L.; Yuan, Y.; Zhang-Negrerie, D.; Du, Y.; K. Zhao, Y. *J. Org. Chem*., **2014**, *79*, 7451–7458.

31 Coppola G. M., *J. Heterocycl. Chem*. **1986**, *23*, 223–224.

32 Roscales, S.; Csákÿ, A. G. *Org. Lett*. **2018**, *20*, 1667–1671.

33 Maity, S.; Zheng, N. *Angew. Chem. Int. Ed*. **2012**, *51*, 9562–9566.

34 Yasusi, Y.; Tetuya, N.; Hitosi, N. *Bull. Chem. Soc. Jpn*. **1971**, *44*, 541–545.

35 Andrew, T. L.; Swager, T. M. *J. Org. Chem*. **2011**, *76*, 2976–2993.

36 Taylor, G. A.; Procter, S. A. *J. Chem. Soc. C* **1971**, 2537–2538.

37 Zi-Yu, Z.; Zhi-Yun, L.; Rui-Ting, G.; Yu-Quan, Z.; Xiang, L.; Xiao-Chen, W. *Angew. Chem. Int. Ed*. **2017**, *56*, 4028–4032.

38 Wolfgang, T.; Ernst, S.; Herbert, K.; Ewald, D. *Chem. Ber*. **2006**, *117*, 2703–2713.

39 Choi, I.; Chung, H.; Park, J. W.; Chung, Y. K. *Org. Lett*. **2016**, *18*, 5508–5511.

40 Cheng, H. G.; Lu, L. Q.; Wang, T.; Yang, Q. Q.; Liu, X. P.; Li, Y.; Deng, Q. H.; Chen, J. R.; Xiao, W. J. *Angew. Chem. Int. Ed*. **2013**, *52*, 3250–3254.

41 Topolski, M. *J. Org. Chem*. **1995**, *60*, 5588–5594.

42 Smith, A. J.; Young, A.; Rohrbach, S.; O'Connor, E. F.; Allison, M.; Wang, H. S.; Poole, D. L.; Tuttle, T.; Murphy, J. A. *Angew. Chem. Int. Ed*. **2017**, *56*, 13747–13751.

43 Wan-Lu, M.; Meirong, W.; Hui-Jing, L.; Deng-Ming, H.; Yi-Yun, Z.; Chao-Yi, L.; Ying, L.; Yan-Chao, W. *Adv. Synth. Catal*. **2017**, *359*, 4250–4257.

44 Freeman, P. K.; Ramnath, N. *J. Org. Chem*. **1991**, *56*, 3646–3651.

45 Wu, H.; Zhang, Z.; Ma, N.; Liu, Q.; Liu, T.; Zhang, G. *J. Org. Chem*., **2018**, *83*, 12880–12886.

NMR Spectra

^{1}H and ^{13}C NMR Spectra of 1-Phenyl-1*H*-indole (**22**)

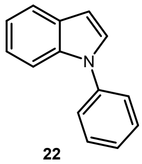

22

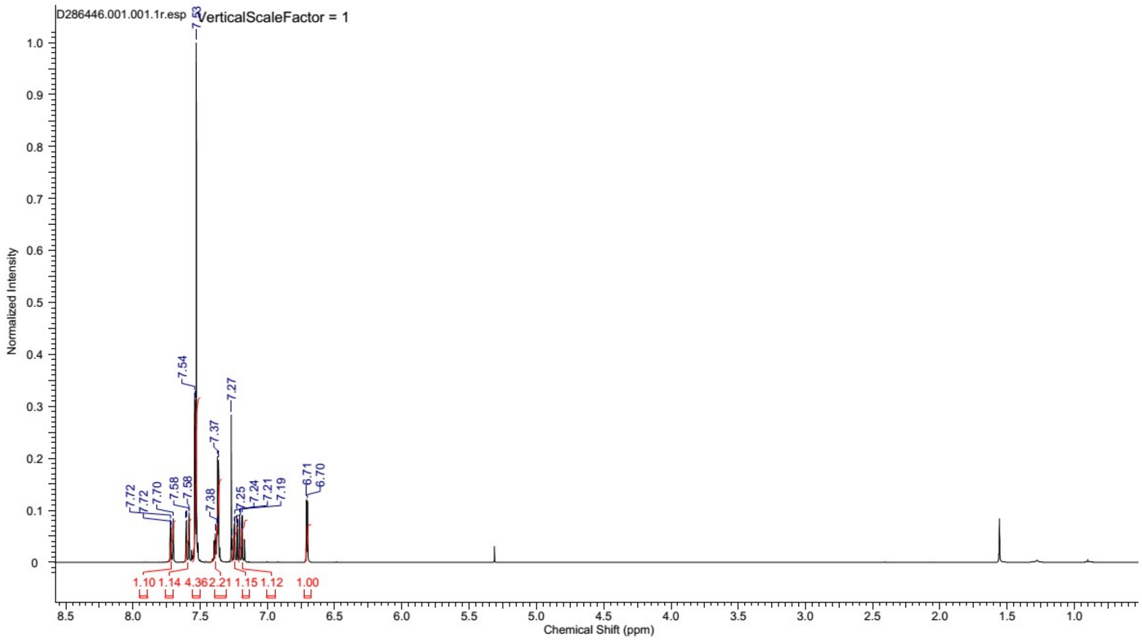

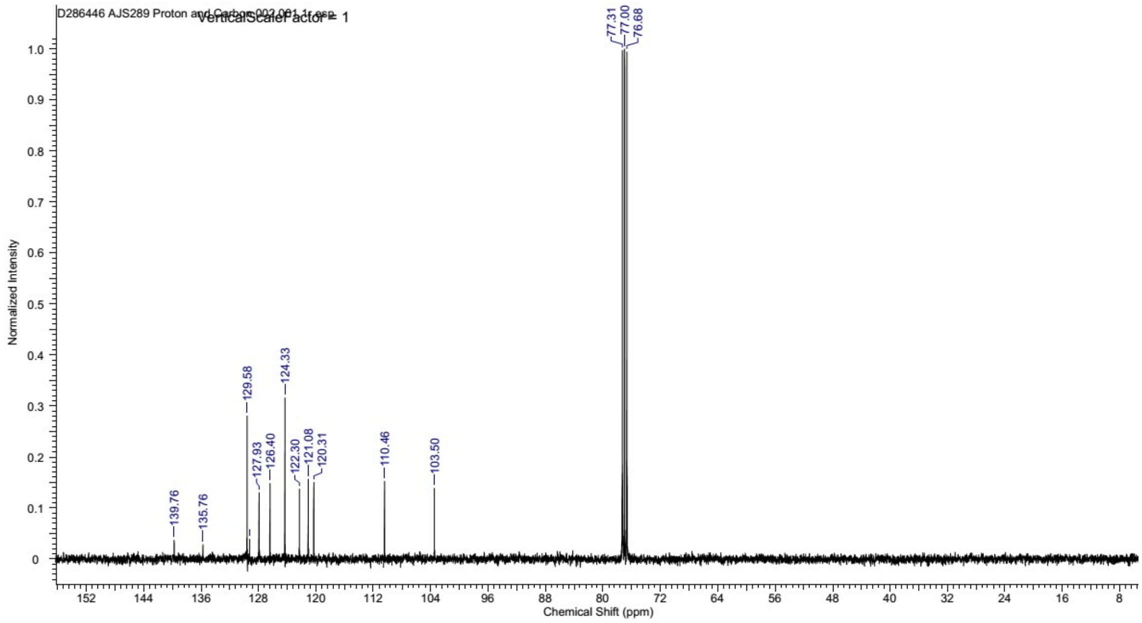

^{1}H and ^{13}C NMR Spectra of 3-Methyl-1-phenyl-1*H*-indole (**23**)

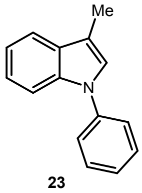

23

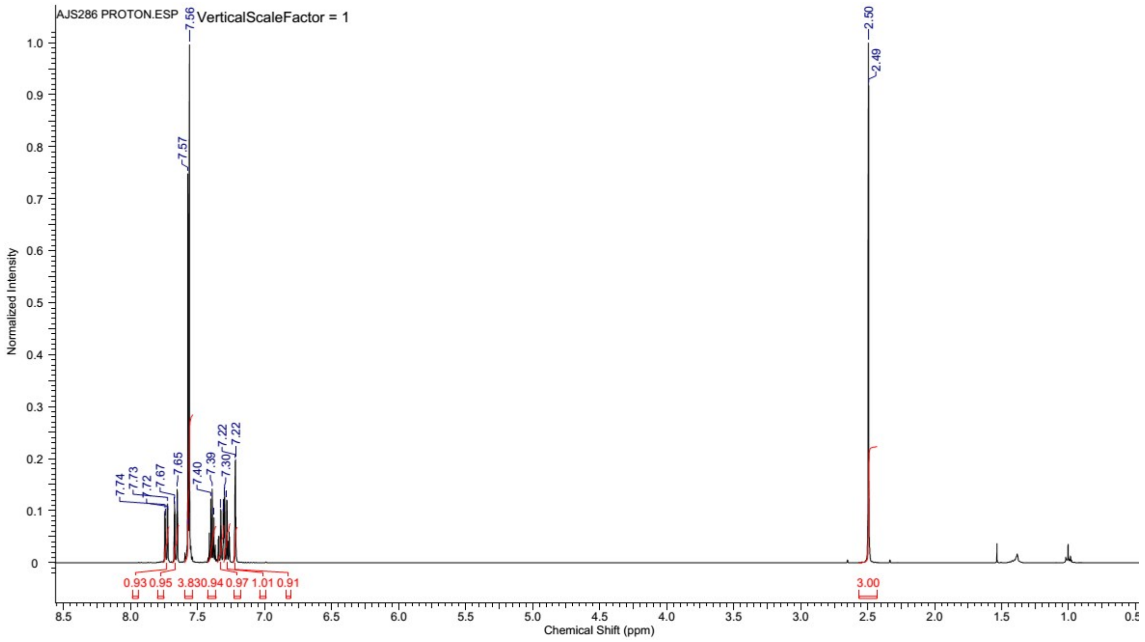

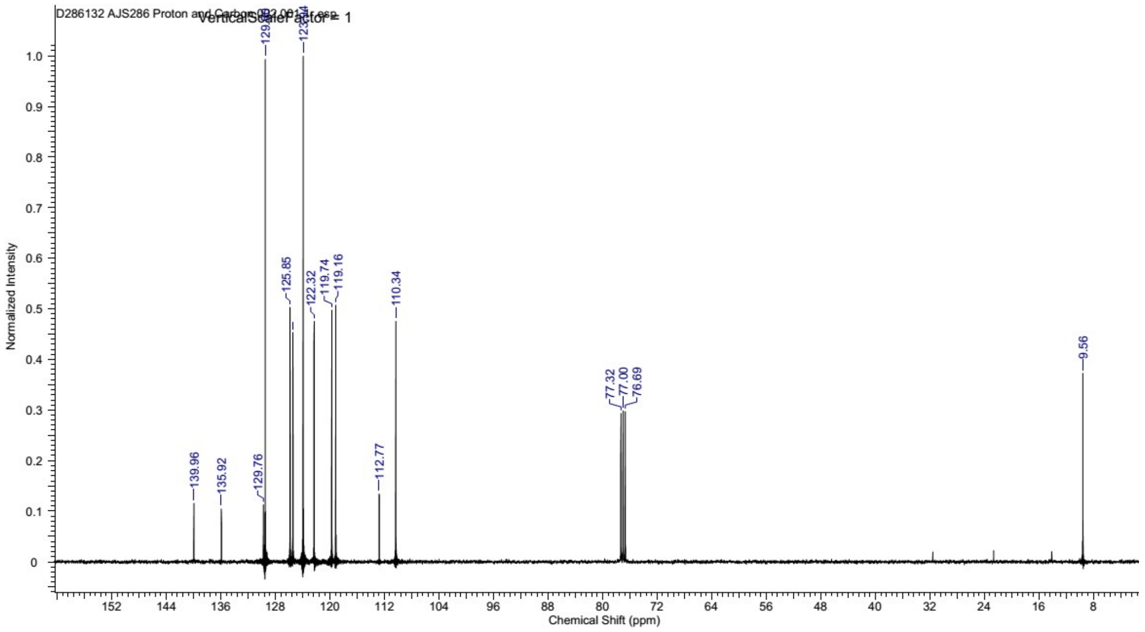

^{1}H and ^{13}C NMR Spectra of 3-Ethyl-1-phenyl-1*H*-indole (**24**)

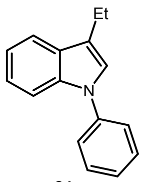

24

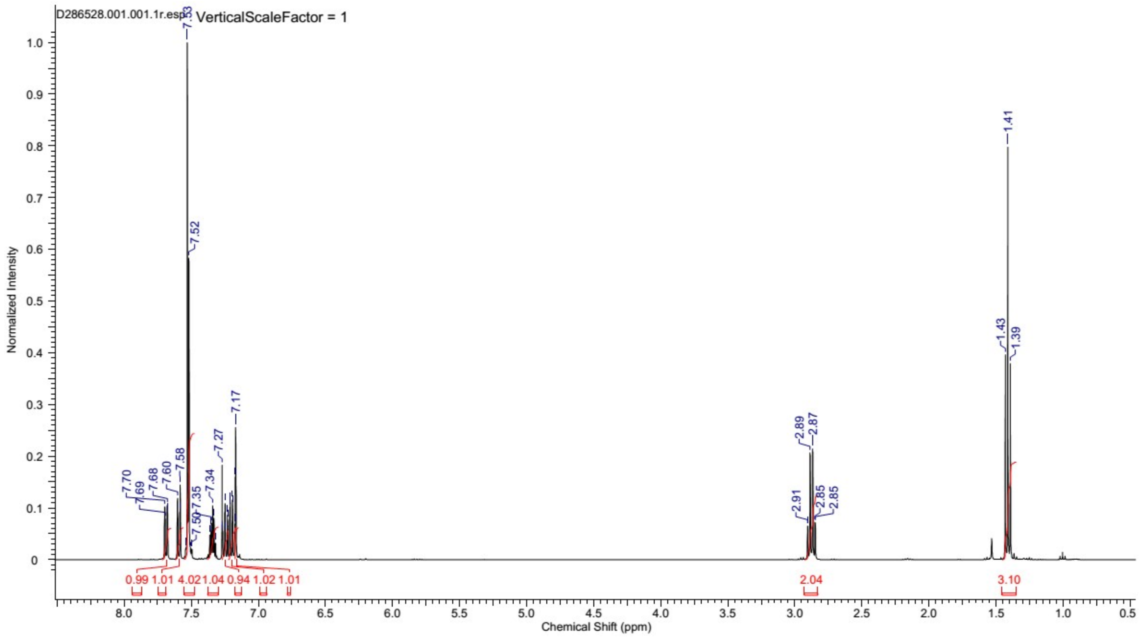

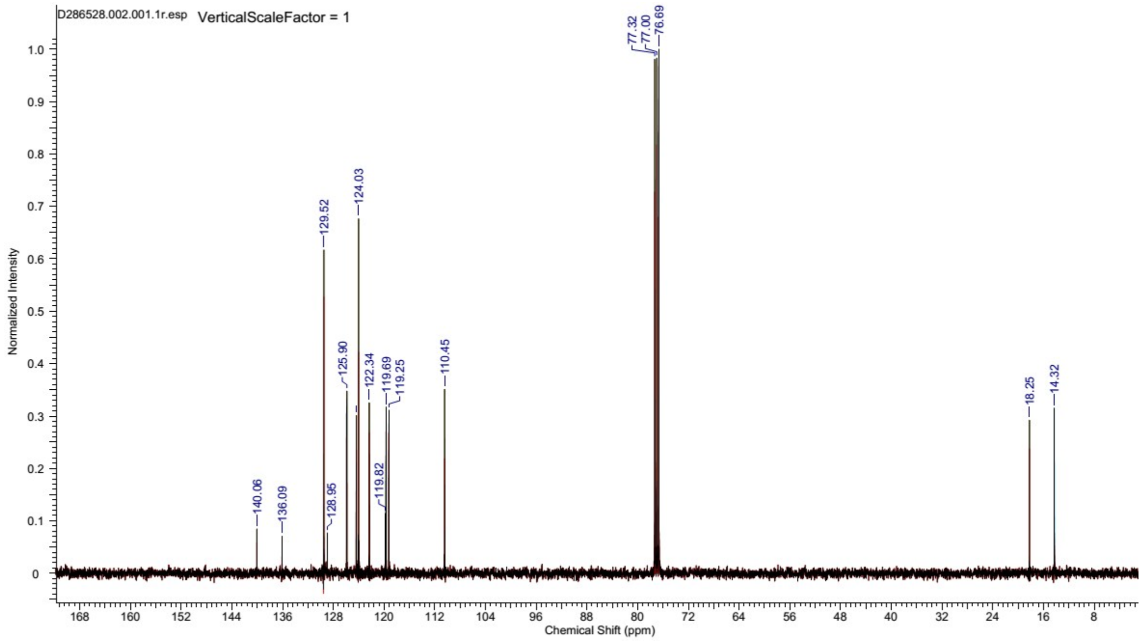

^1H and ^{13}C NMR Spectra of 2,3-Dimethyl-1-phenyl-1*H*-indole (**25**)

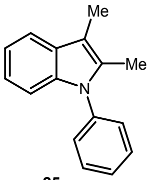

25

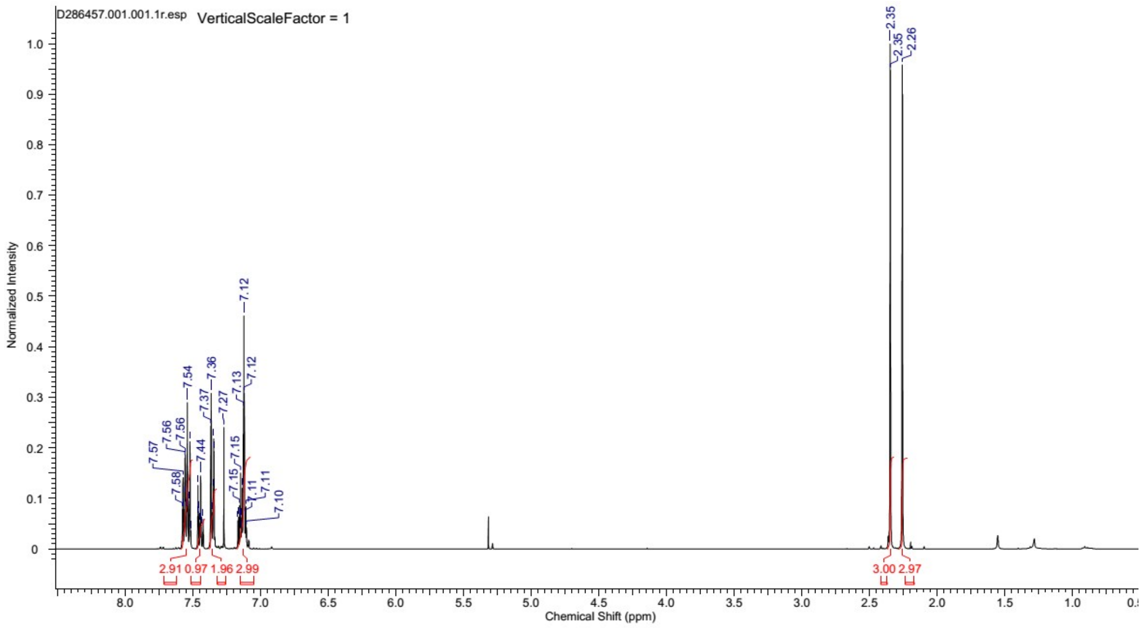

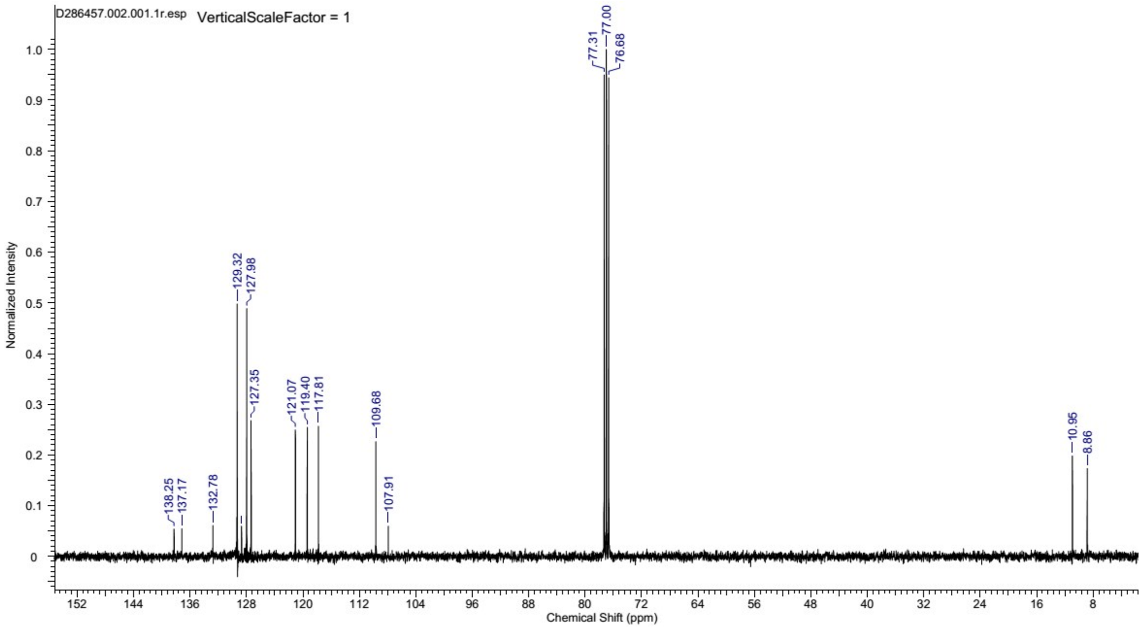

^{1}H and ^{13}C NMR Spectra of 3-Phenyl-1*H*-indole

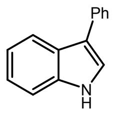

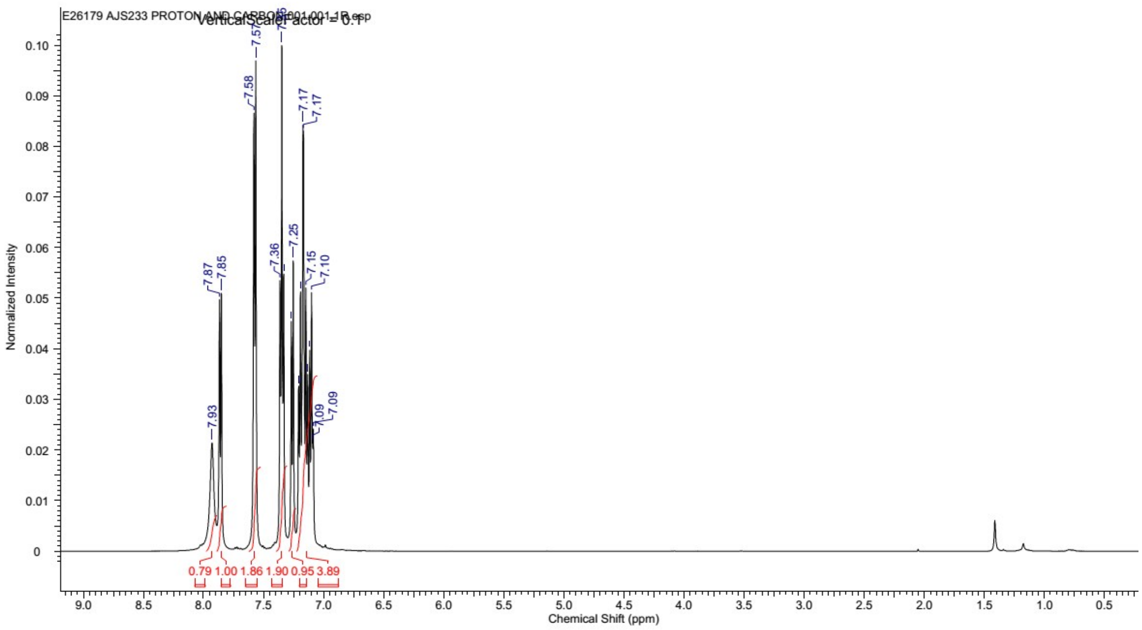

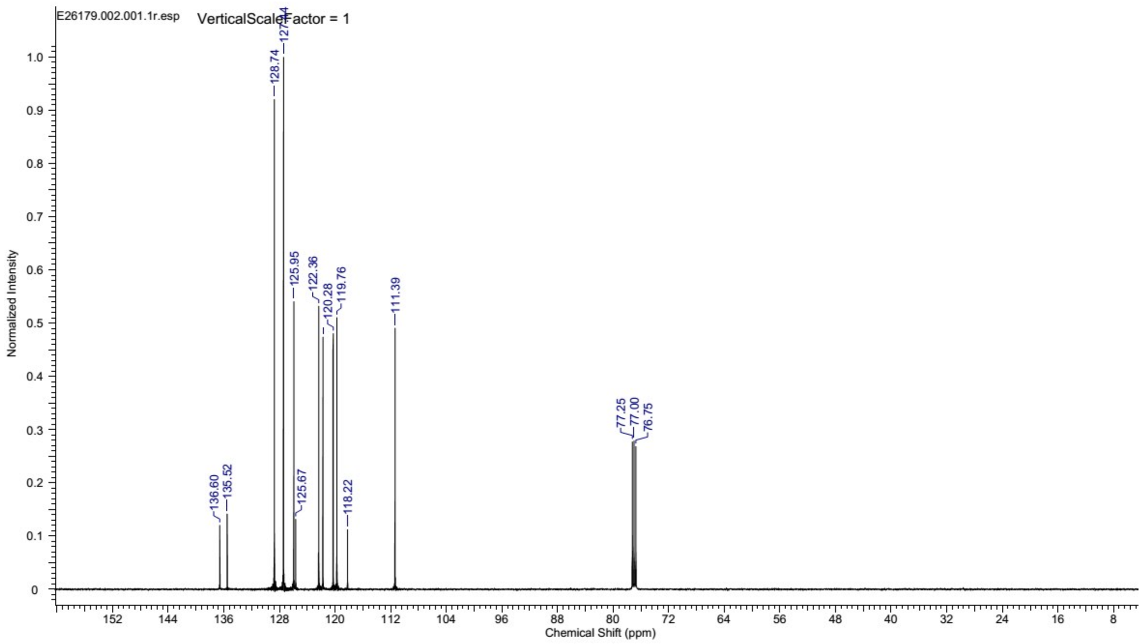

^{1}H and ^{13}C NMR Spectra of 1,3-Diphenyl-1*H*-indole (**26**)

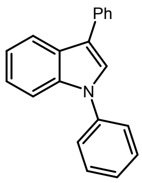

26

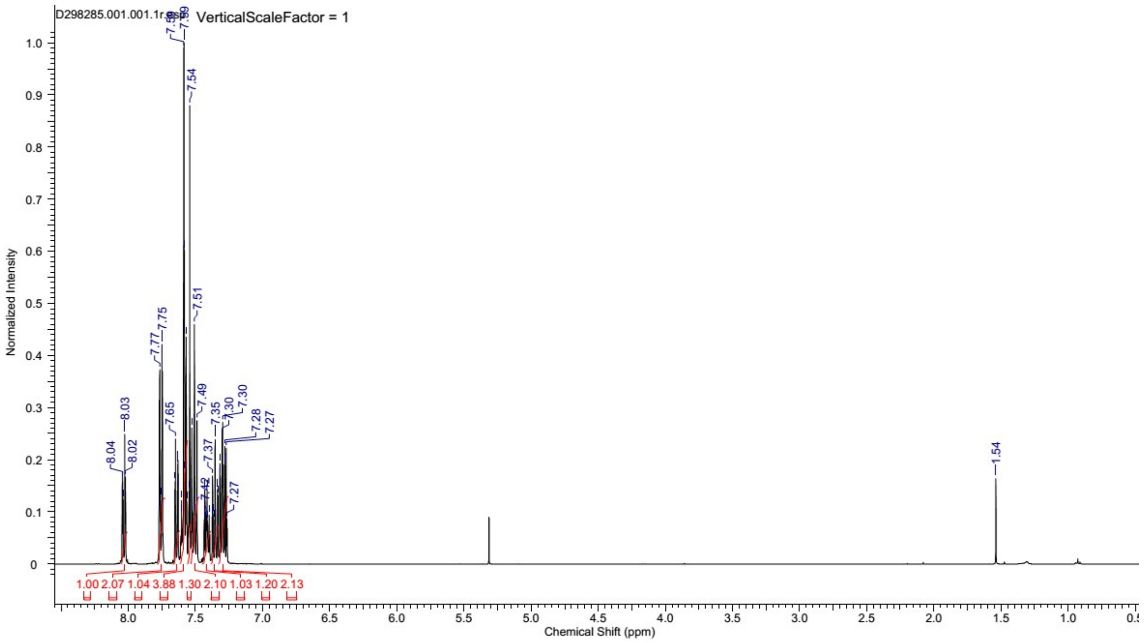

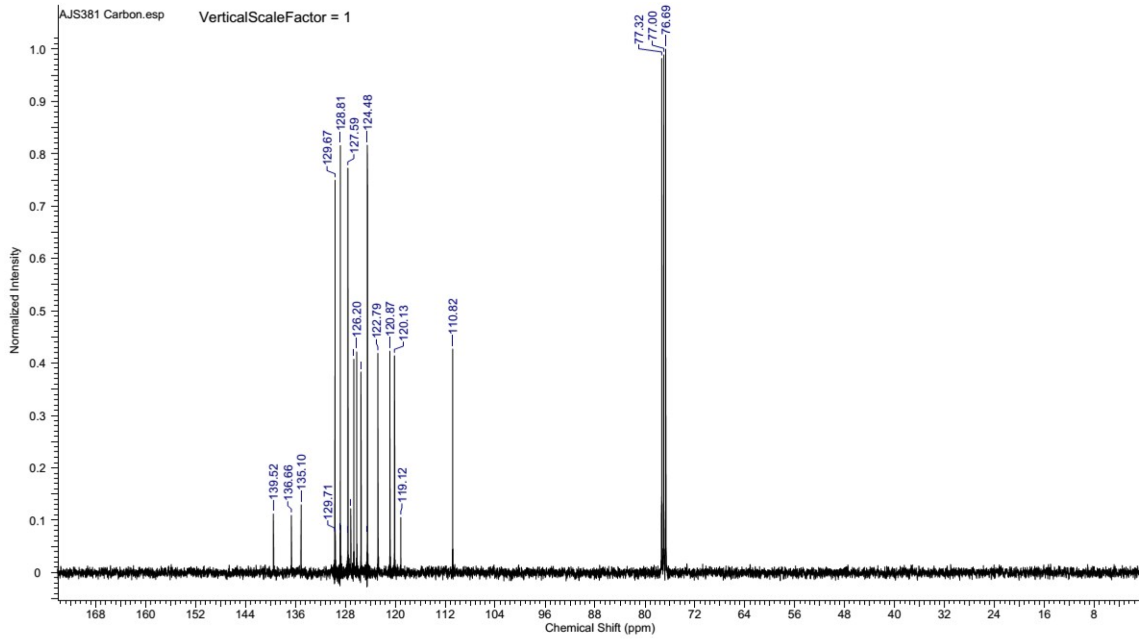

^1H and ^{13}C NMR Spectra of 3-Octyl-1*H*-indole

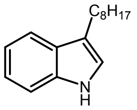

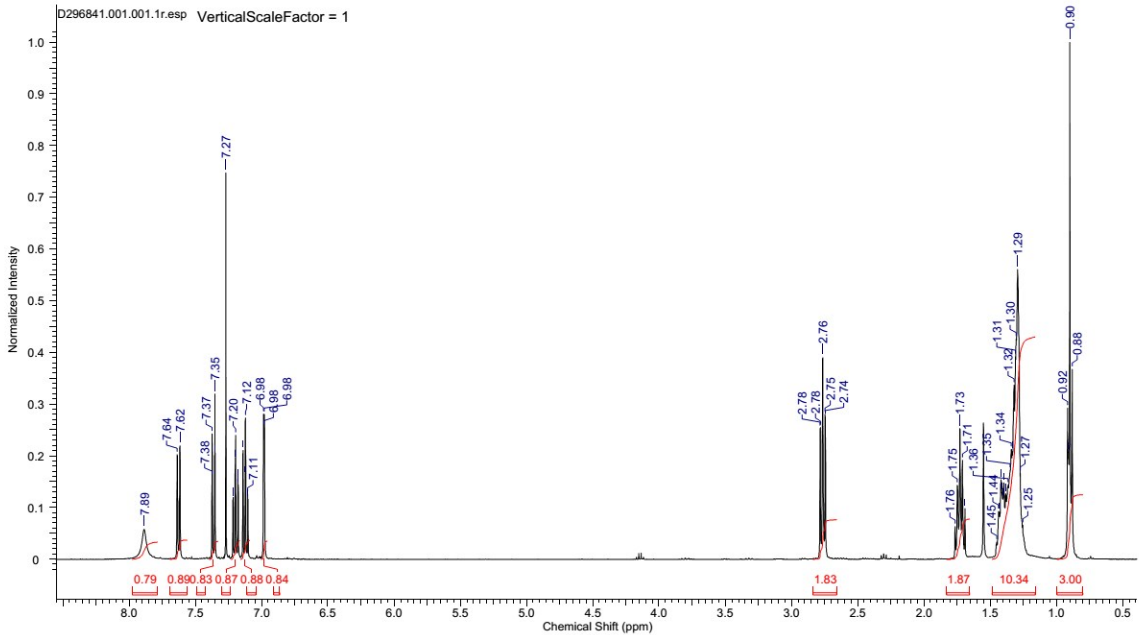

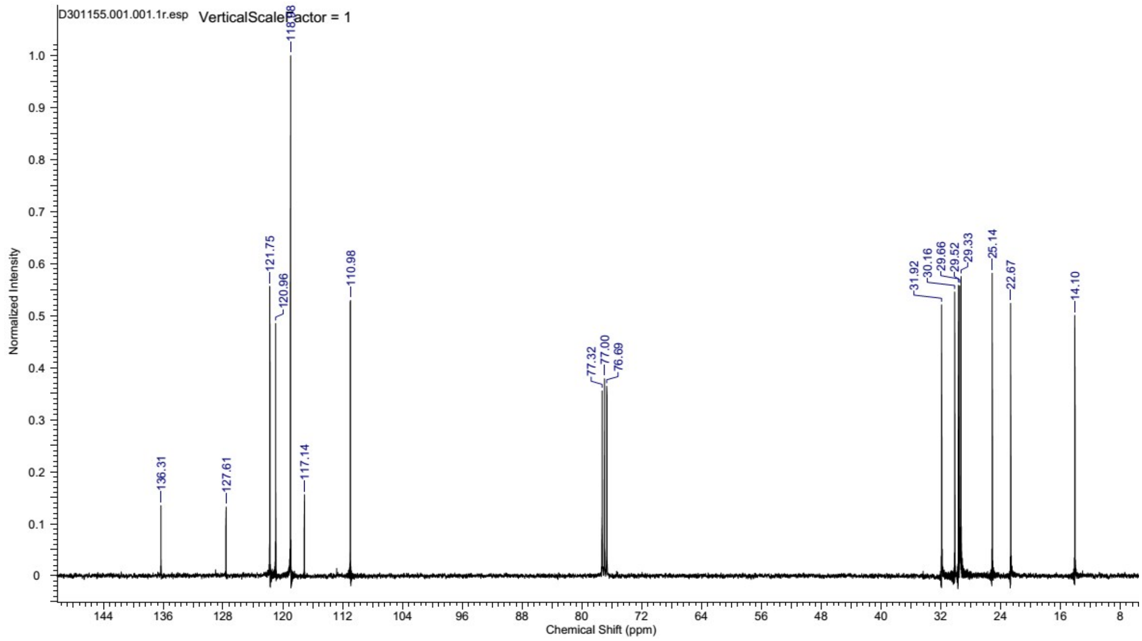

^1H and ^{13}C NMR Spectra of 3-Octyl-1-phenyl-1*H*-indole (**27**)

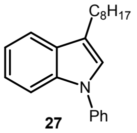

27

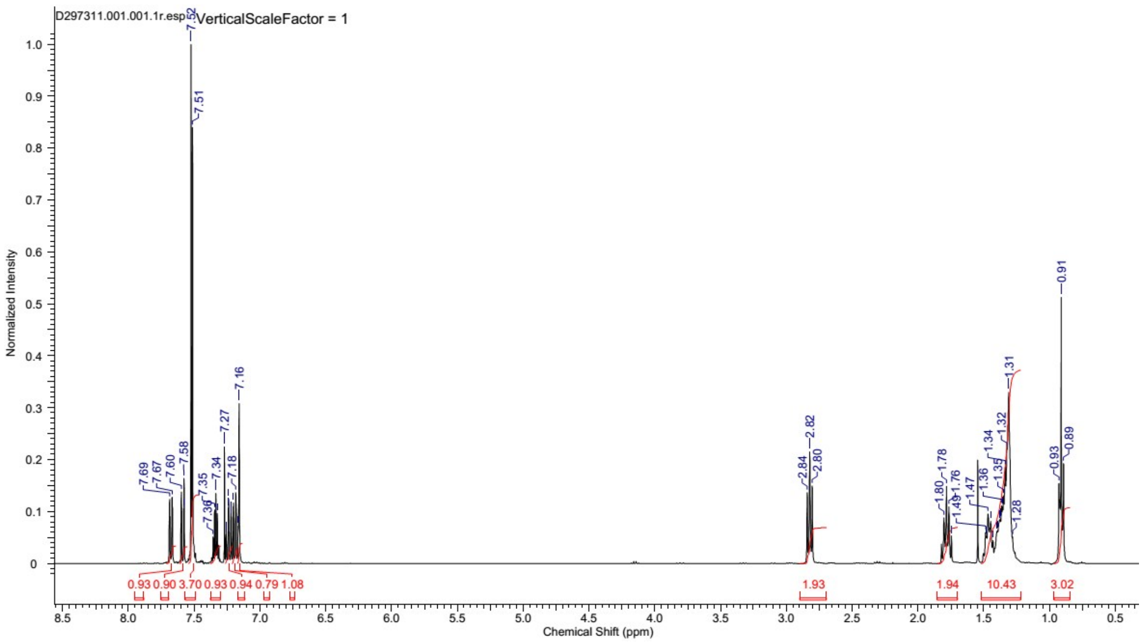

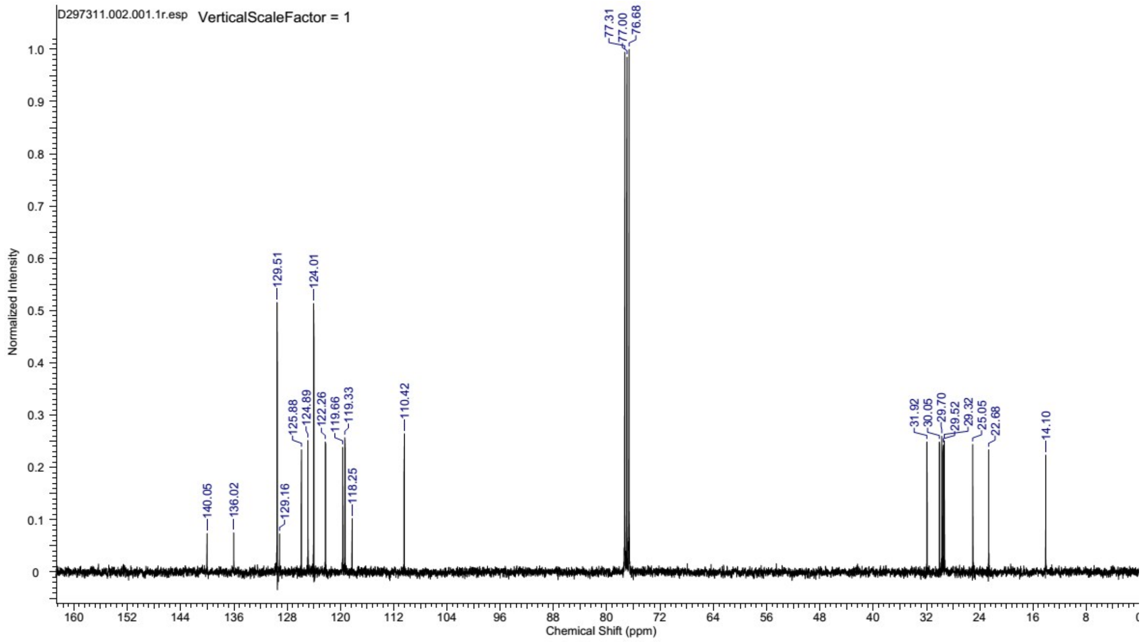

^{1}H and ^{13}C NMR Spectra of 3-Butyl-1*H*-indole

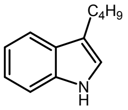

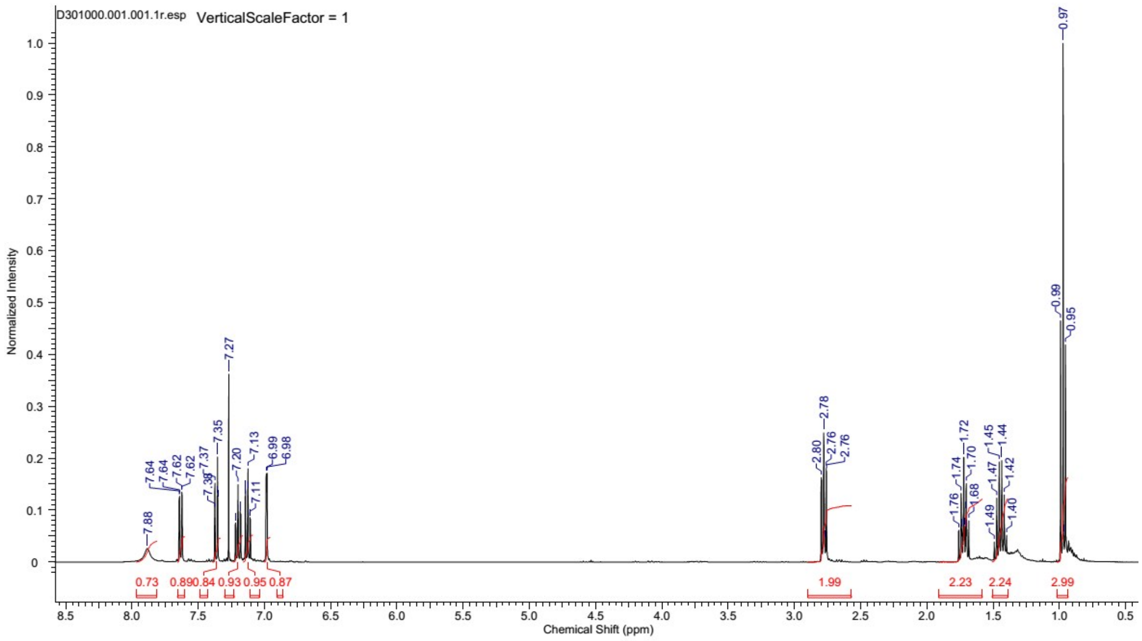

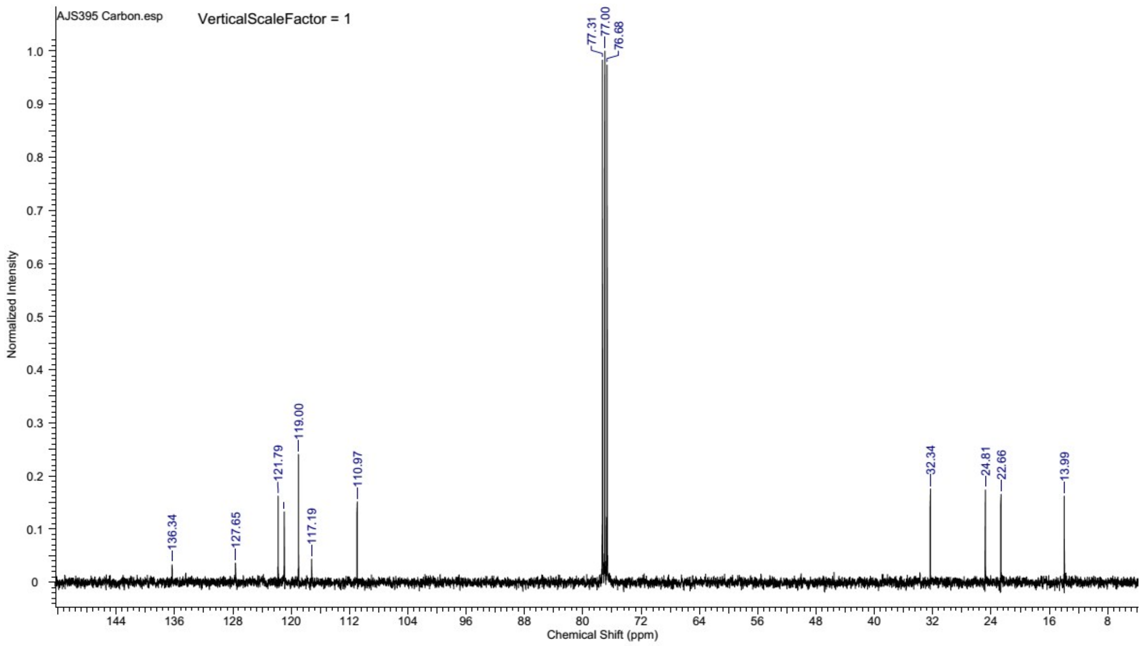

^{1}H and ^{13}C NMR Spectra of 3-Butyl-1-phenyl-1*H*-indole (**28**)

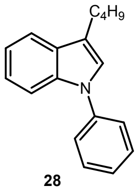

28

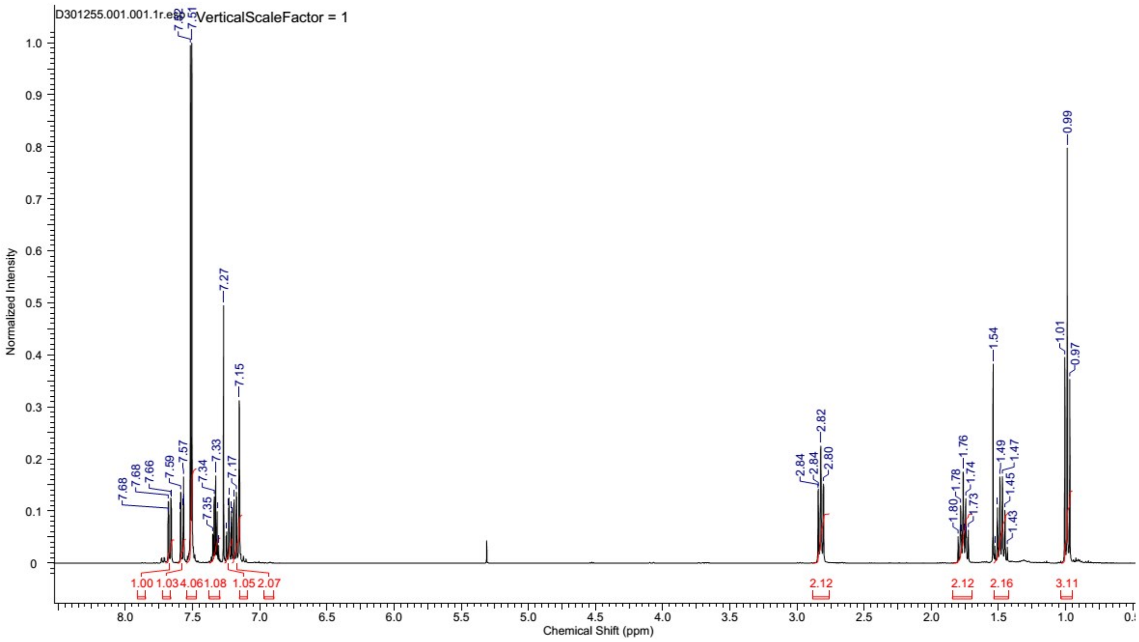

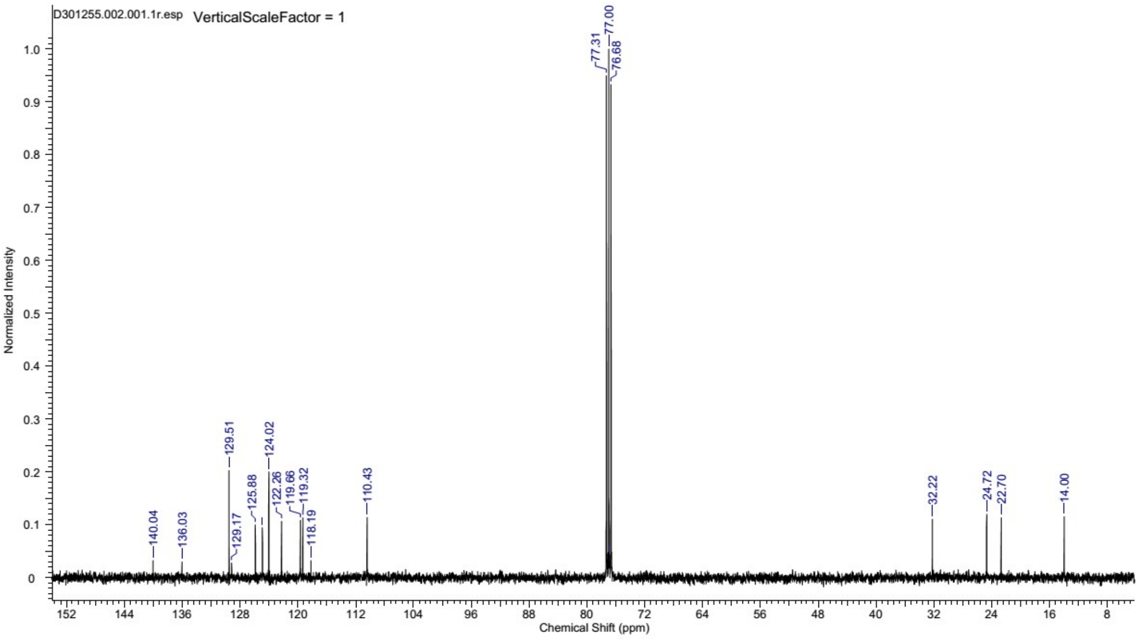

^{1}H and ^{13}C NMR Spectra of 1-(*p*-Tolyl)-1*H*-indole (**29**)

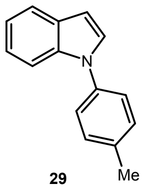

29

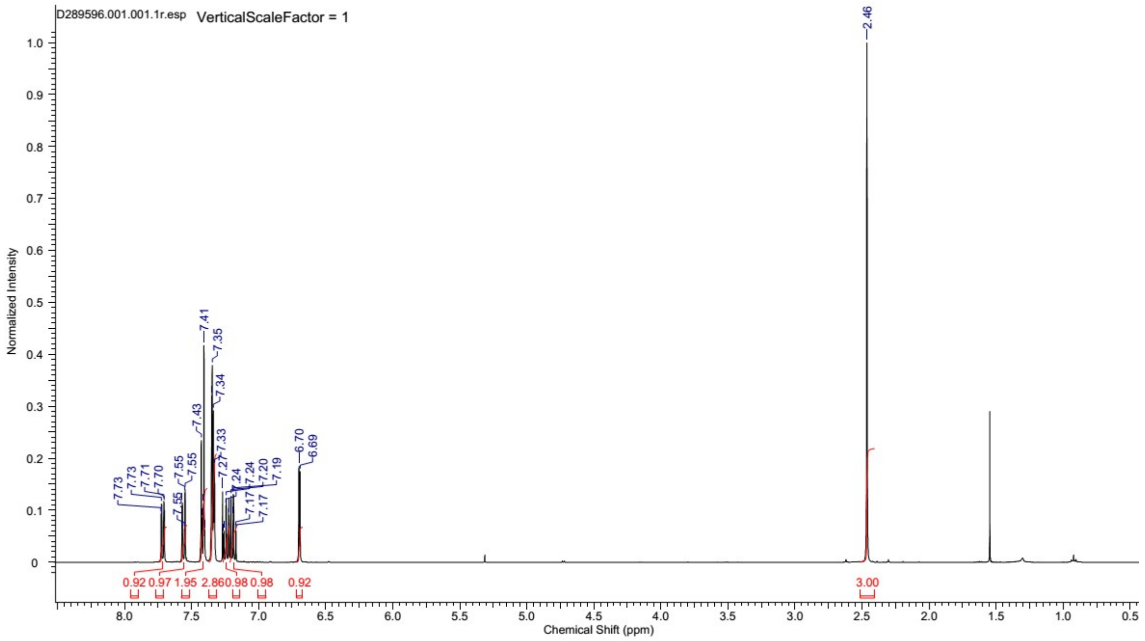

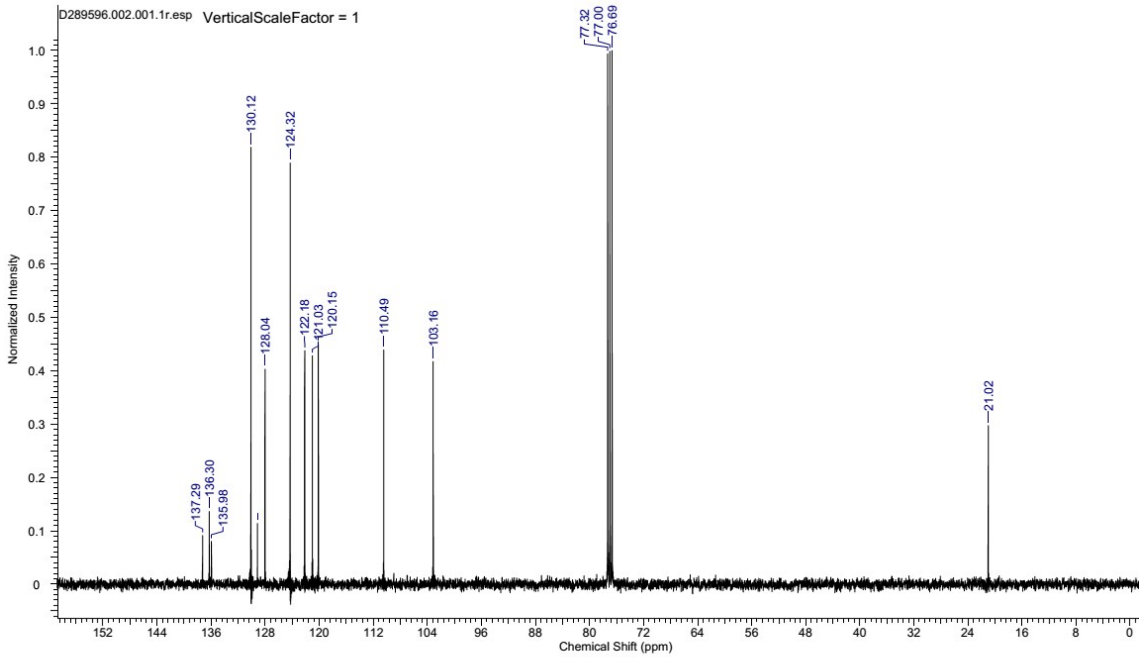

^{1}H and ^{13}C NMR Spectra of 3-Methyl-1-(naphthalen-1-yl)-1*H*-indole (**47**)

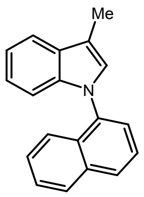

47

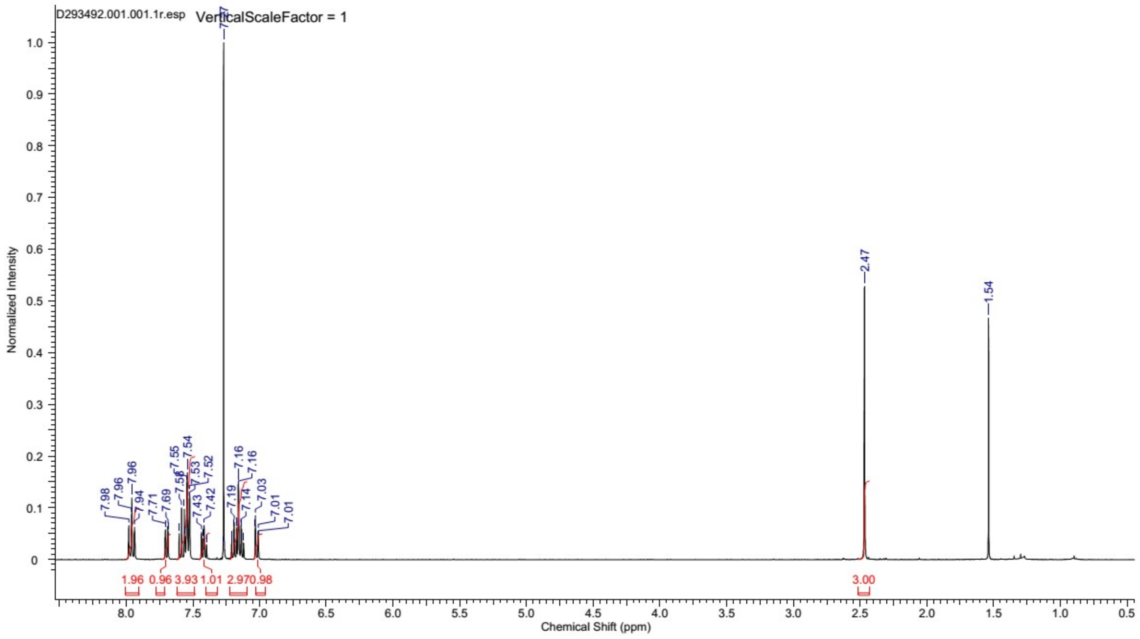

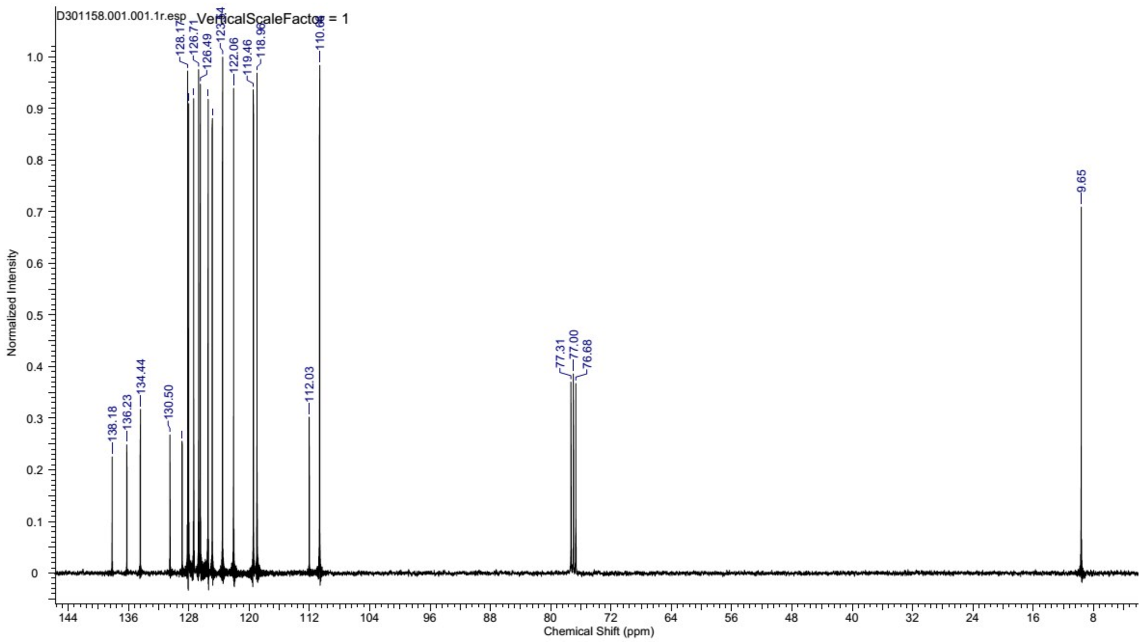

^{1}H and ^{13}C NMR Spectra of 1-(Pyridin-3-yl)-1*H*-indole (**48**)

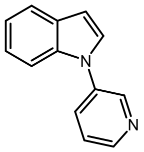

48

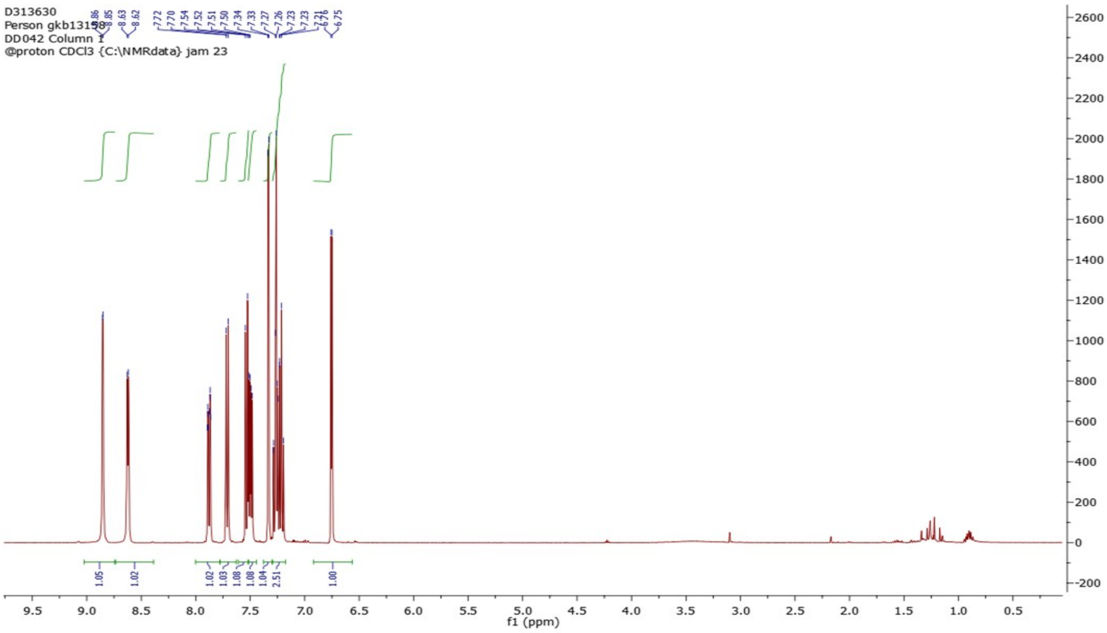

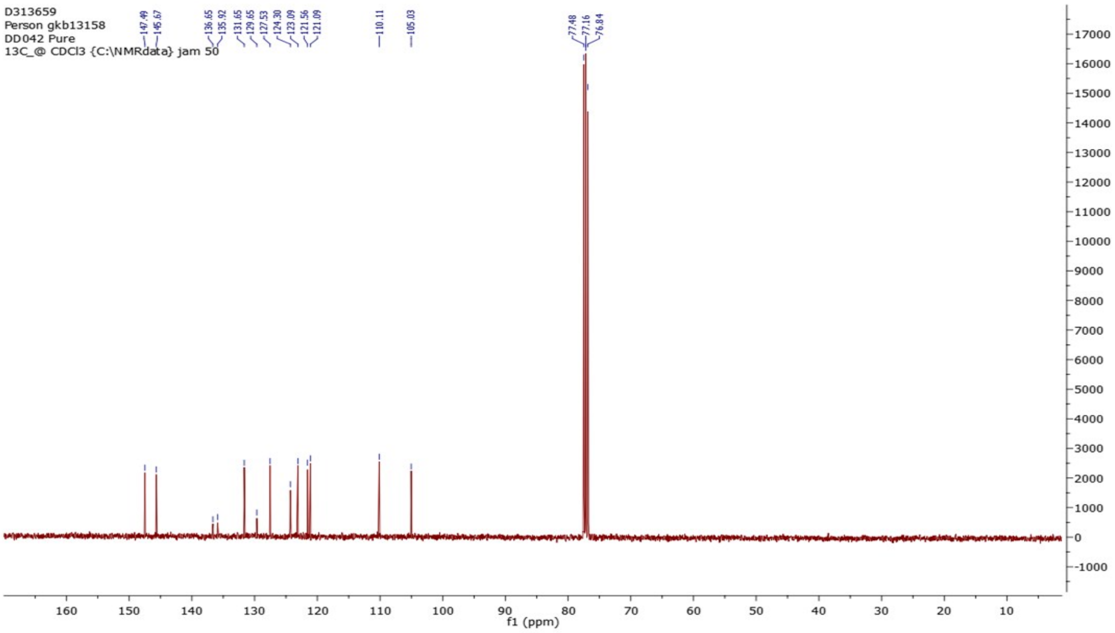

^1H and ^{13}C NMR Spectra of 1-(Pyridin-2-yl)-1*H*-indole (**49**)

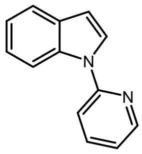

49

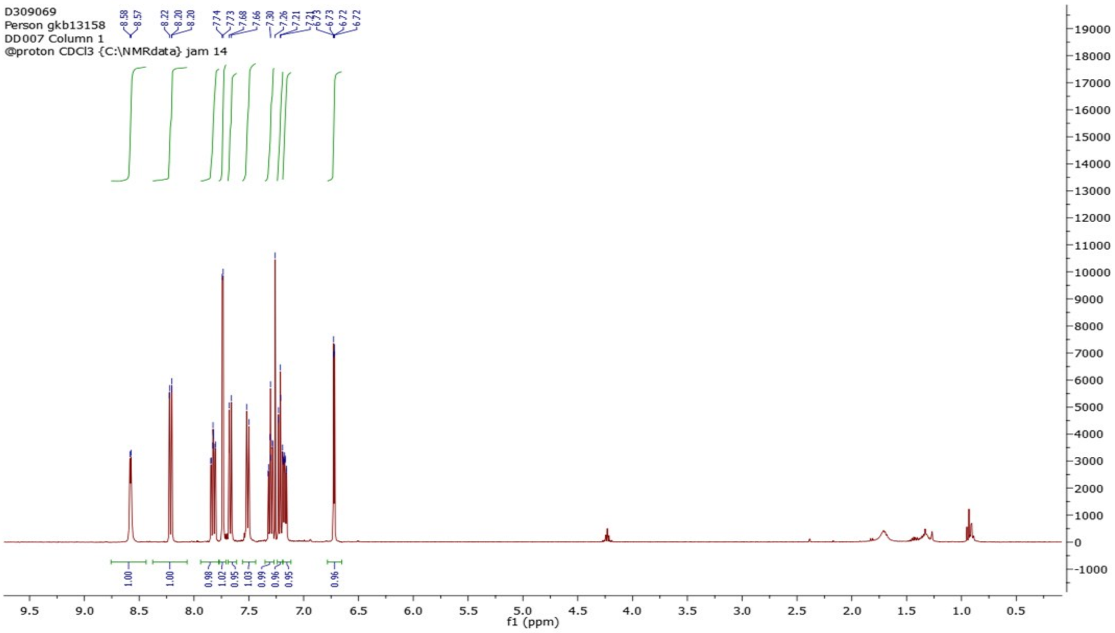

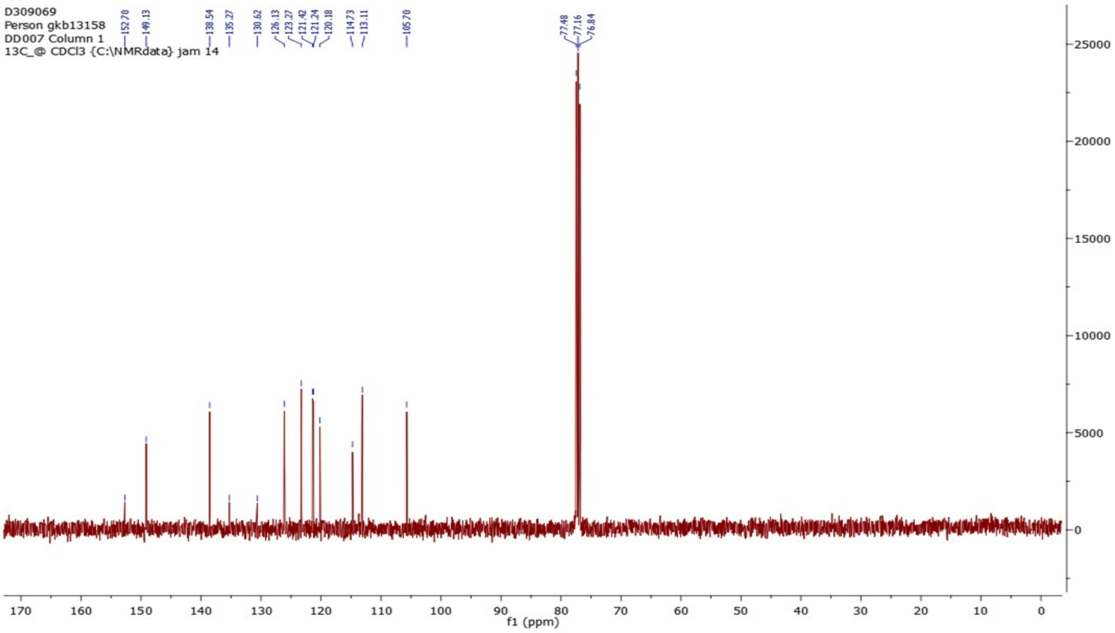

^{1}H and ^{13}C NMR Spectra of 3-(But-3-en-1-yl)-1-phenyl-1*H*-indole (**50**)

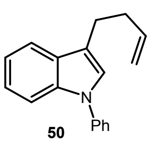

50

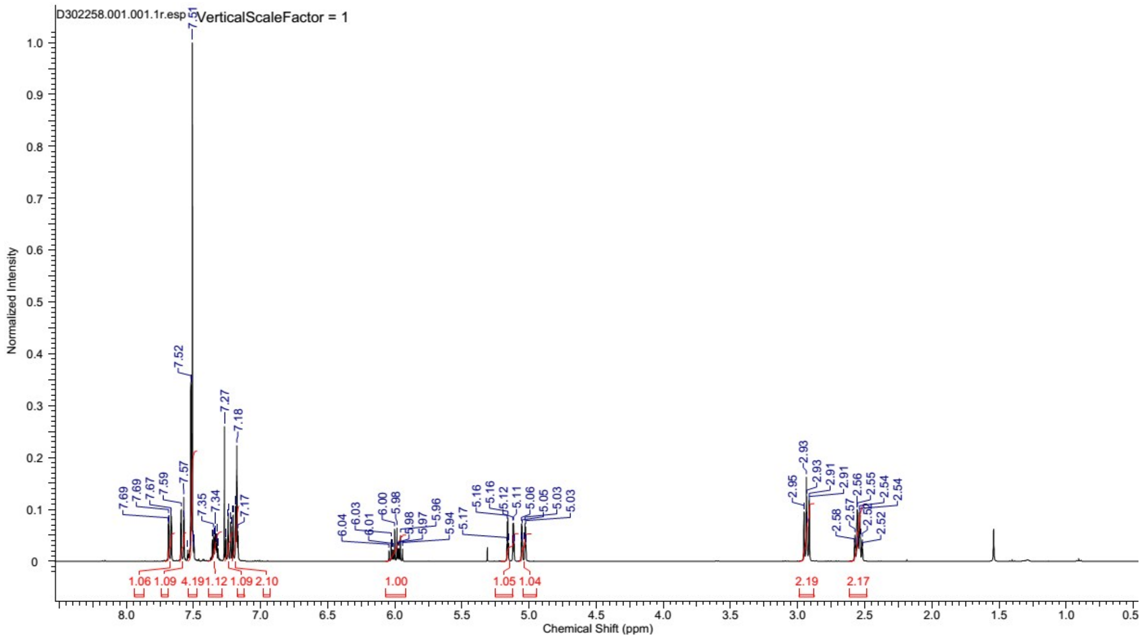

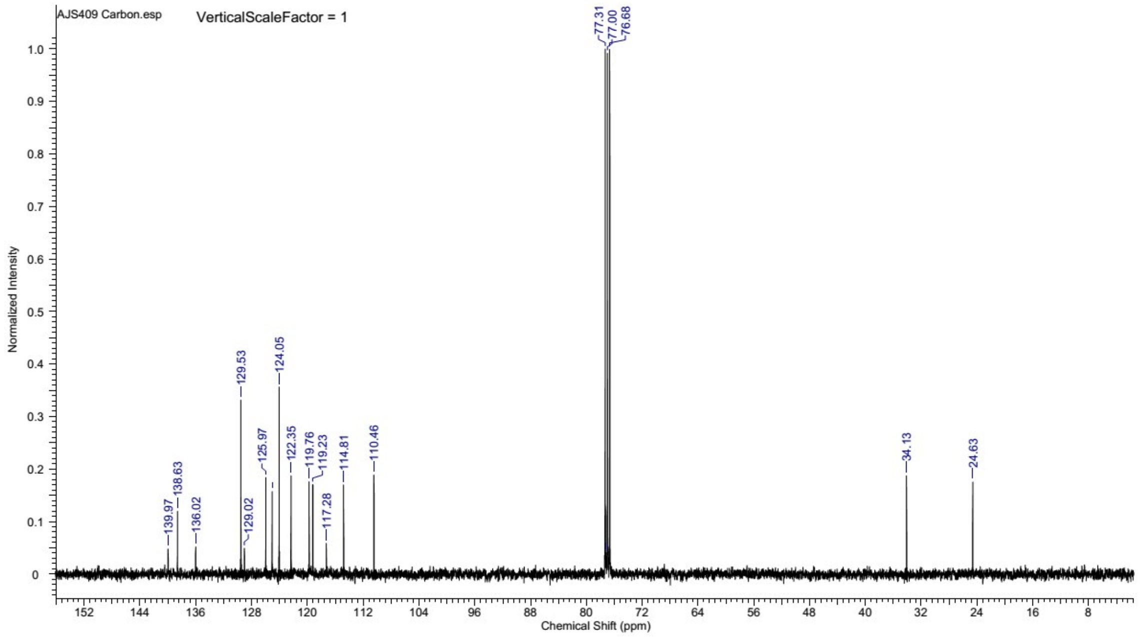

^{1}H and ^{13}C NMR Spectra of 3-(Pent-4-en-1-yl)-1-phenyl-1*H*-indole (**51**)

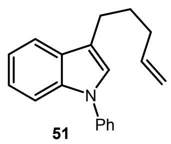

51

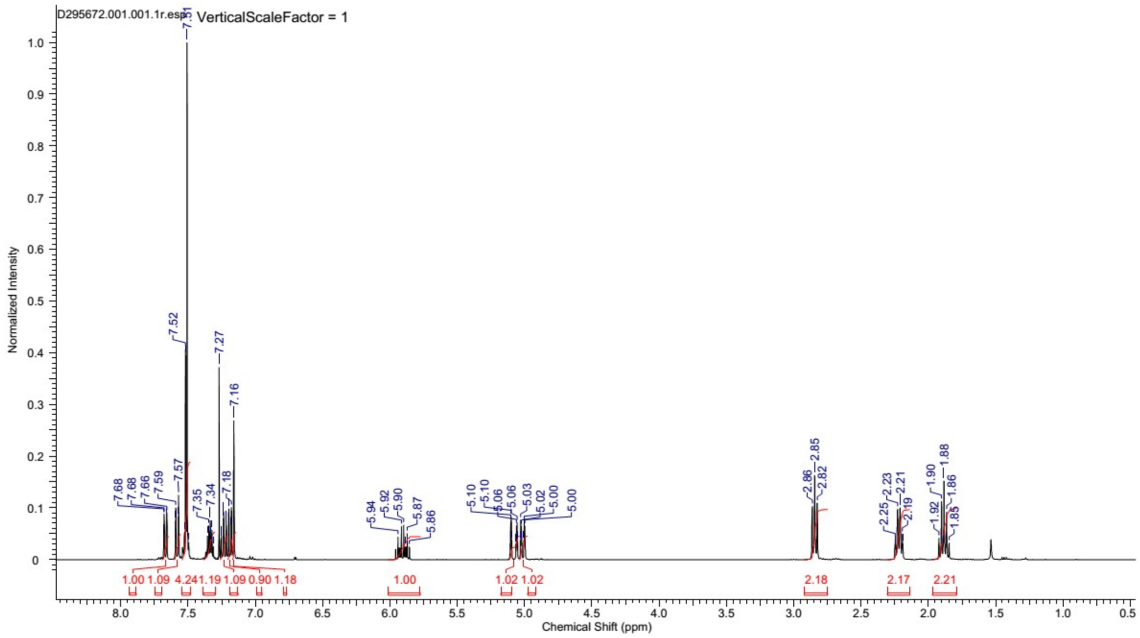

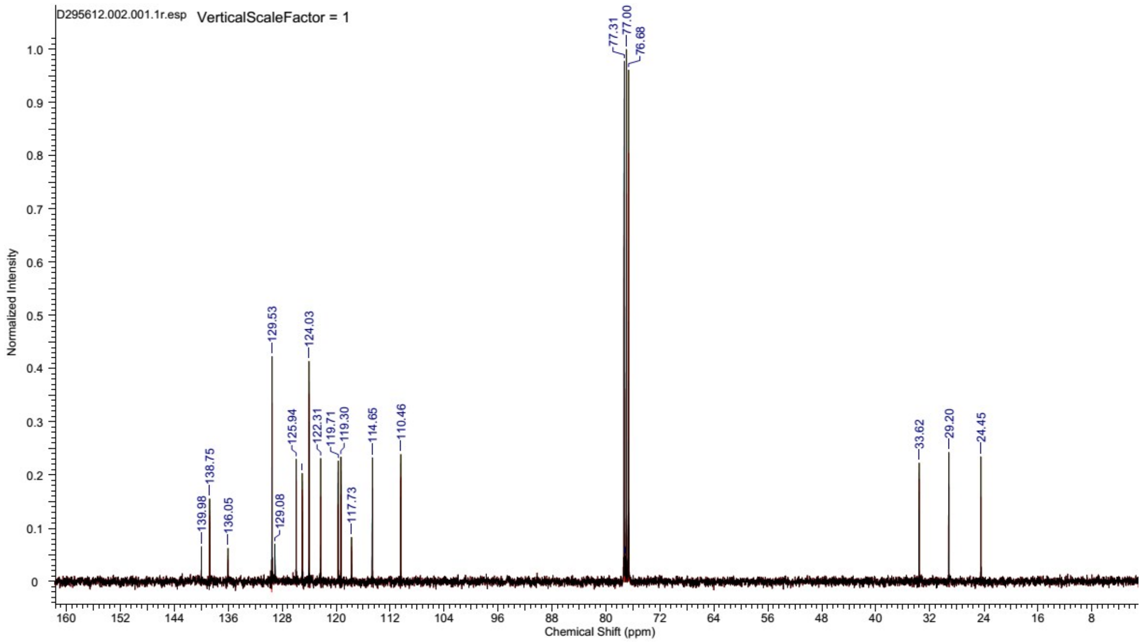

^{1}H and ^{13}C NMR Spectra of 3-(1-(Trimethylsilyl)cyclopropyl)-1H-indole

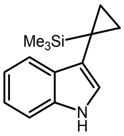

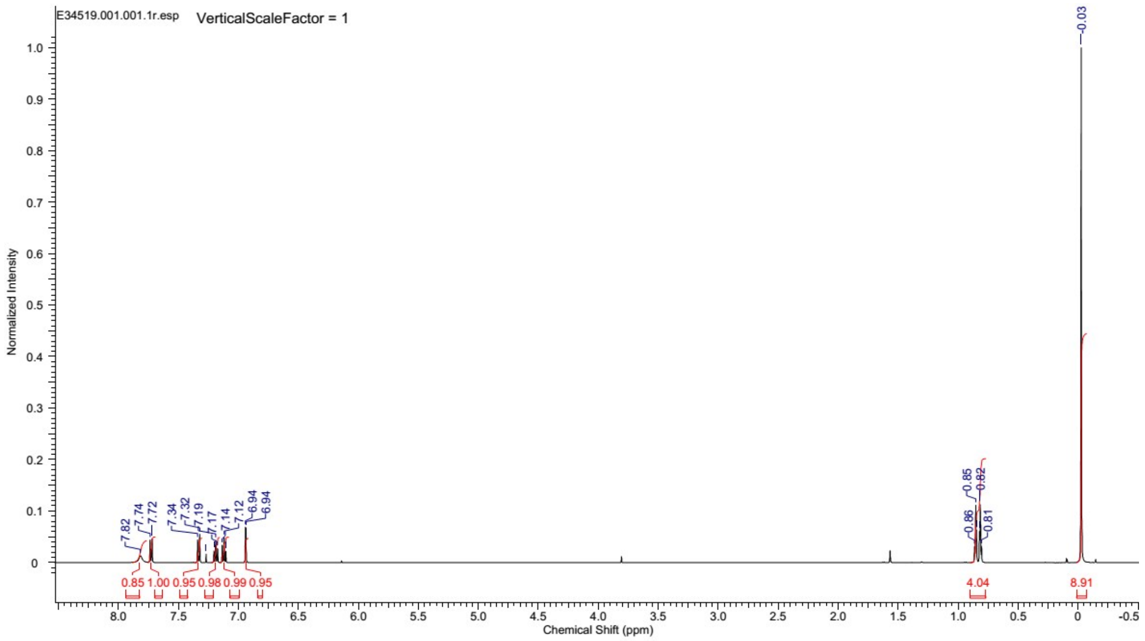

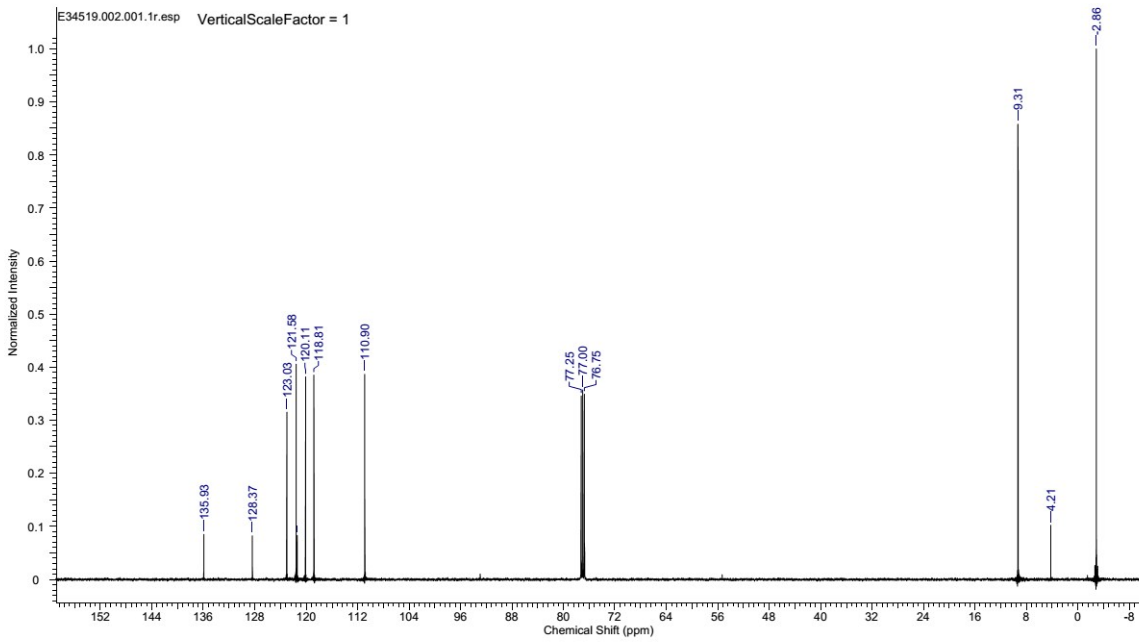

^{1}H and ^{13}C NMR Spectra of 3-Cyclopropyl -1*H*-indole

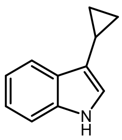

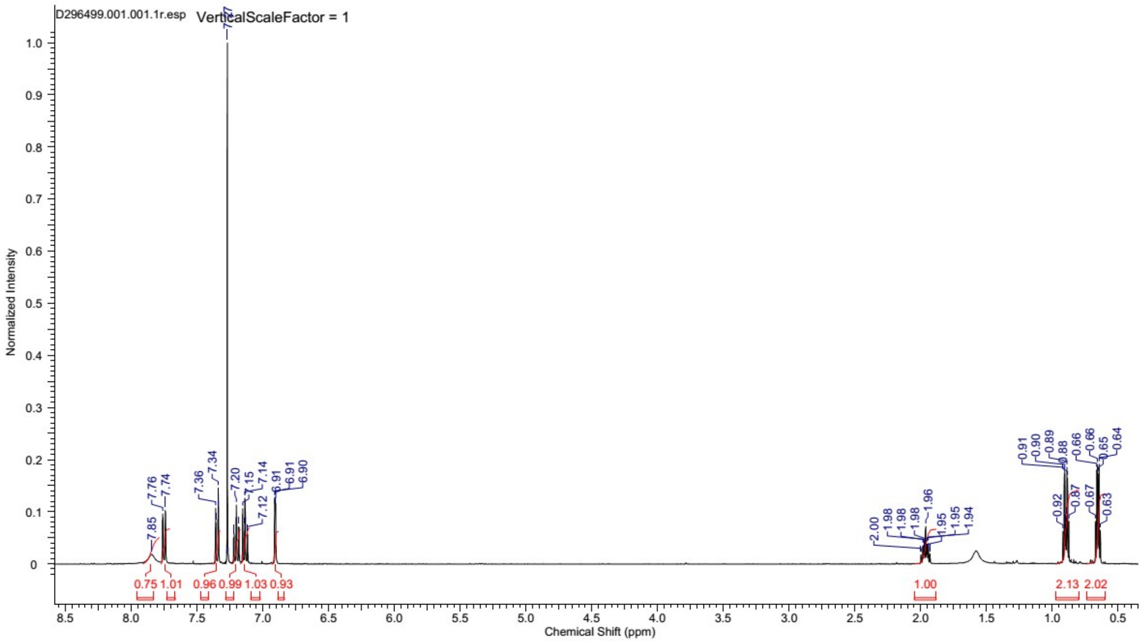

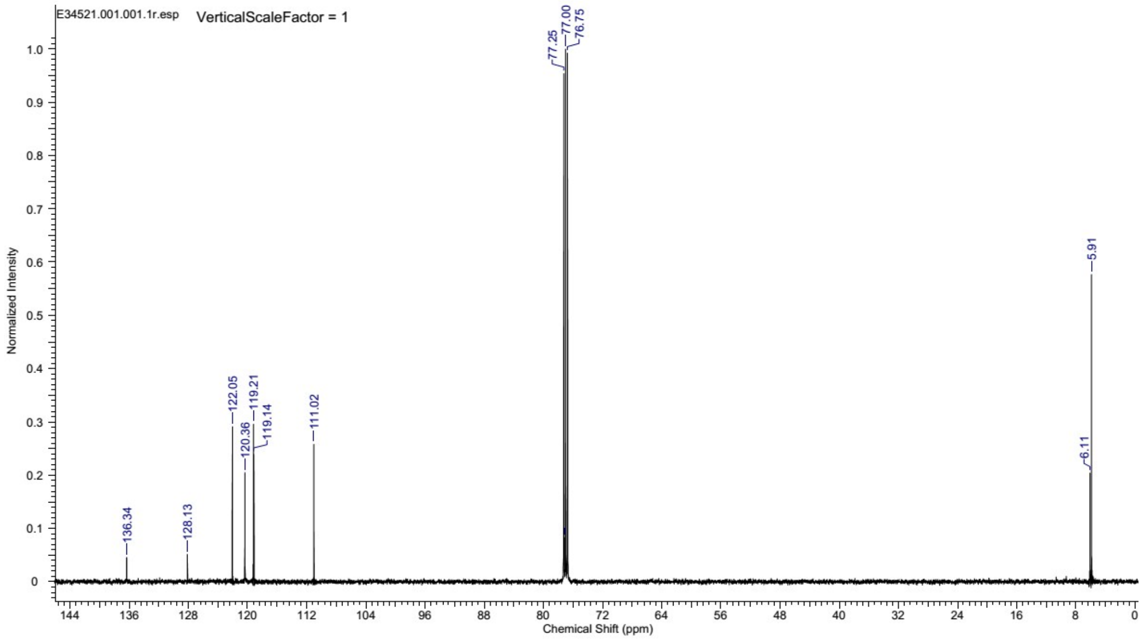

^{1}H and ^{13}C NMR Spectra of 3-Cyclopropyl-1-phenyl-1*H*-indole (**61**)

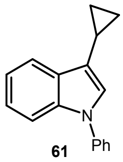

61

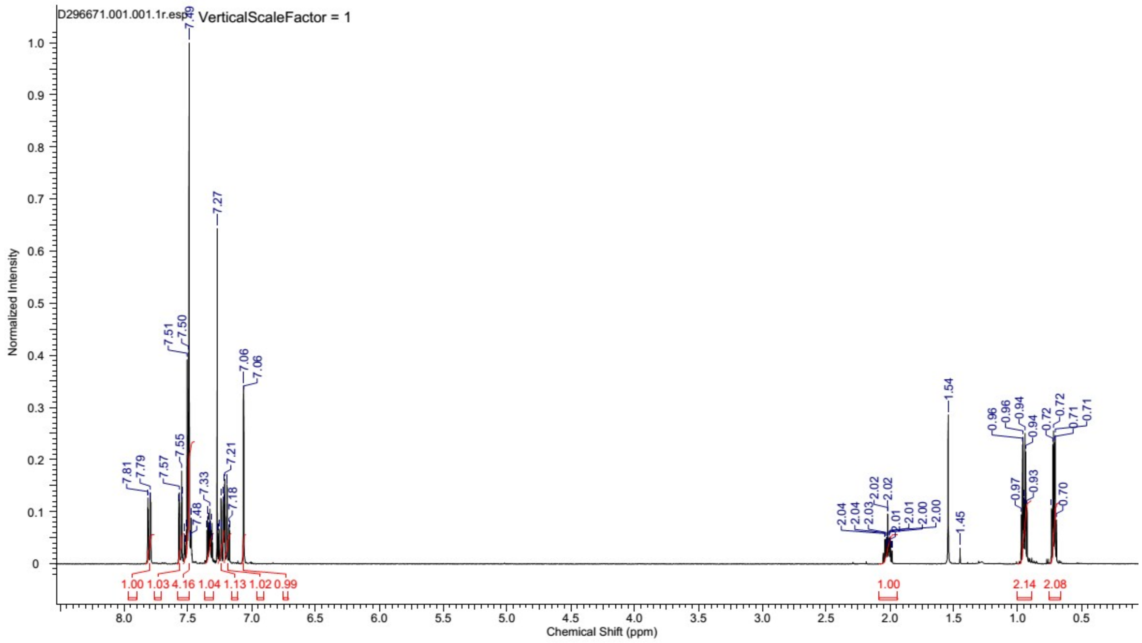

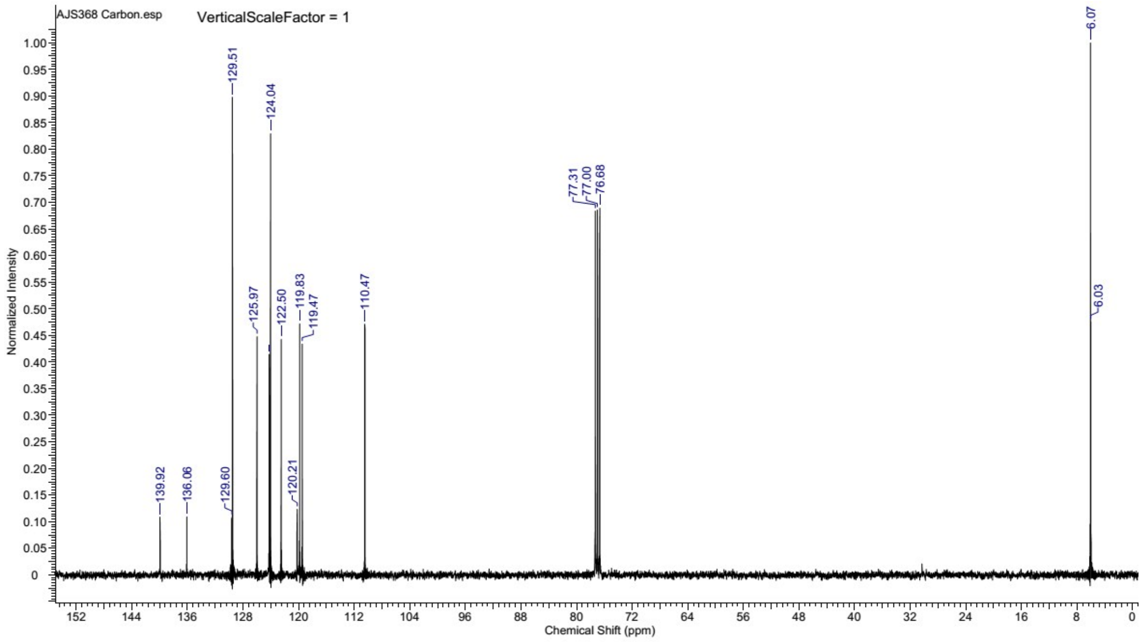

^{1}H and ^{13}C NMR Spectra of 2-(Cyclopropylethynyl)aniline

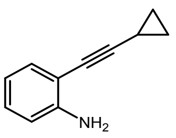

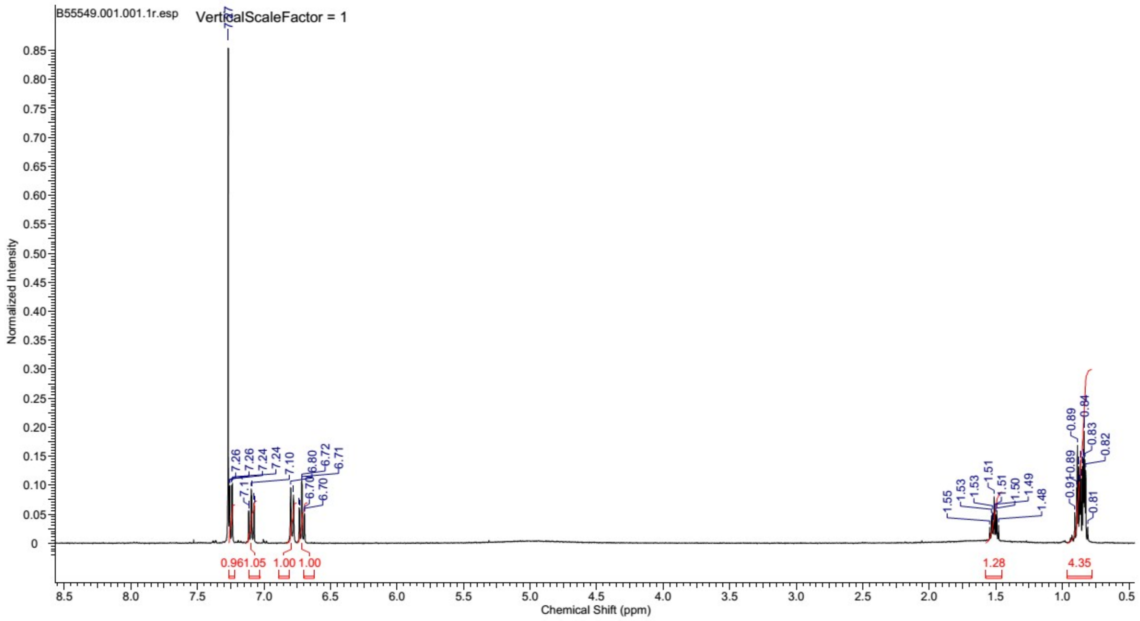

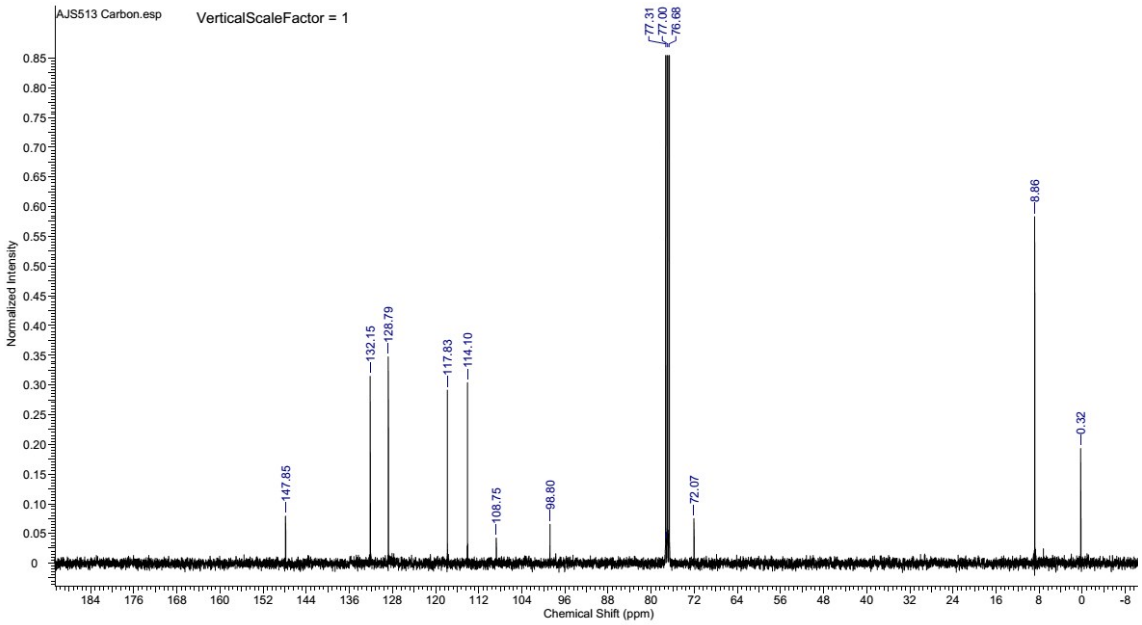

^{1}H and ^{13}C NMR Spectra of 2-Cyclopropyl-1*H*-indole

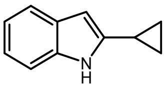

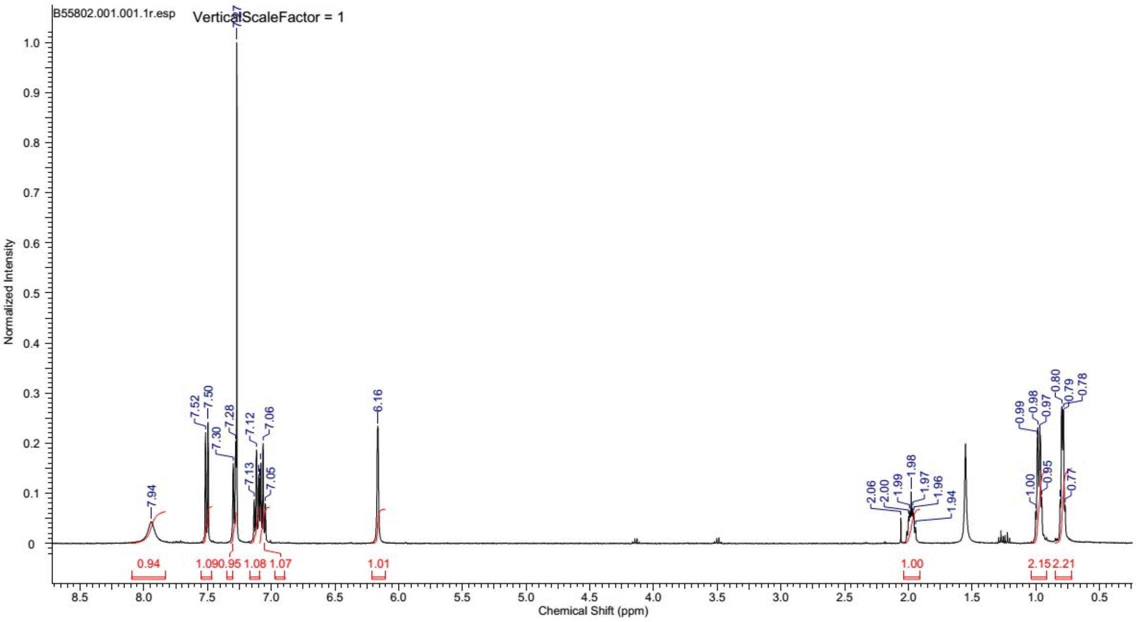

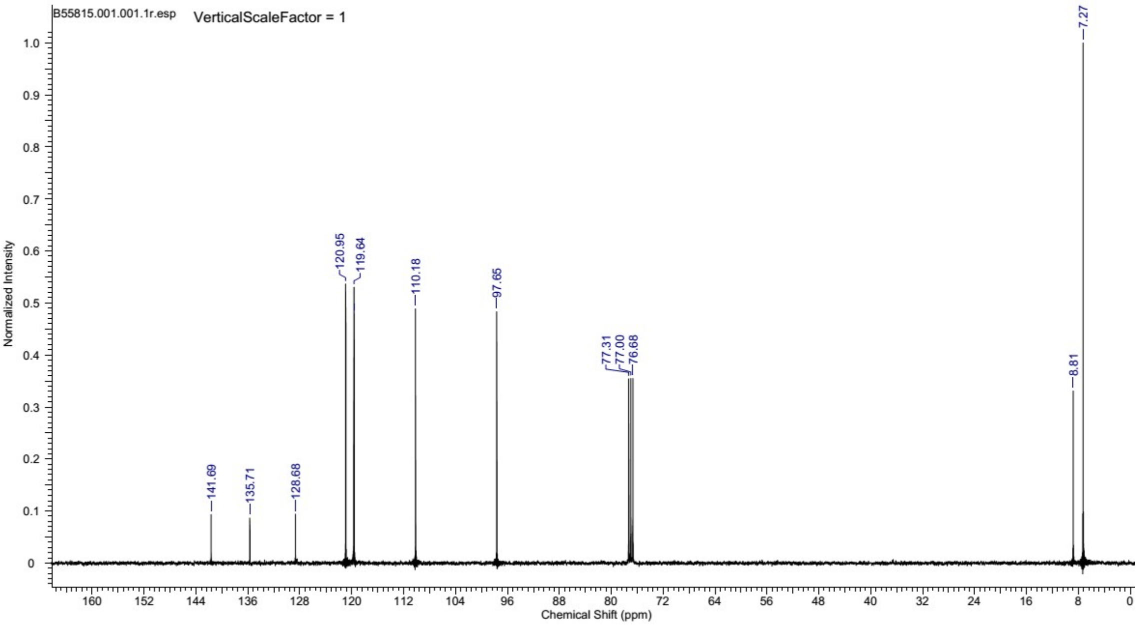

^{1}H and ^{13}C NMR Spectra of 2-Cyclopropyl-1-phenyl-1*H*-indole (**73**)

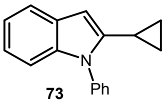

73

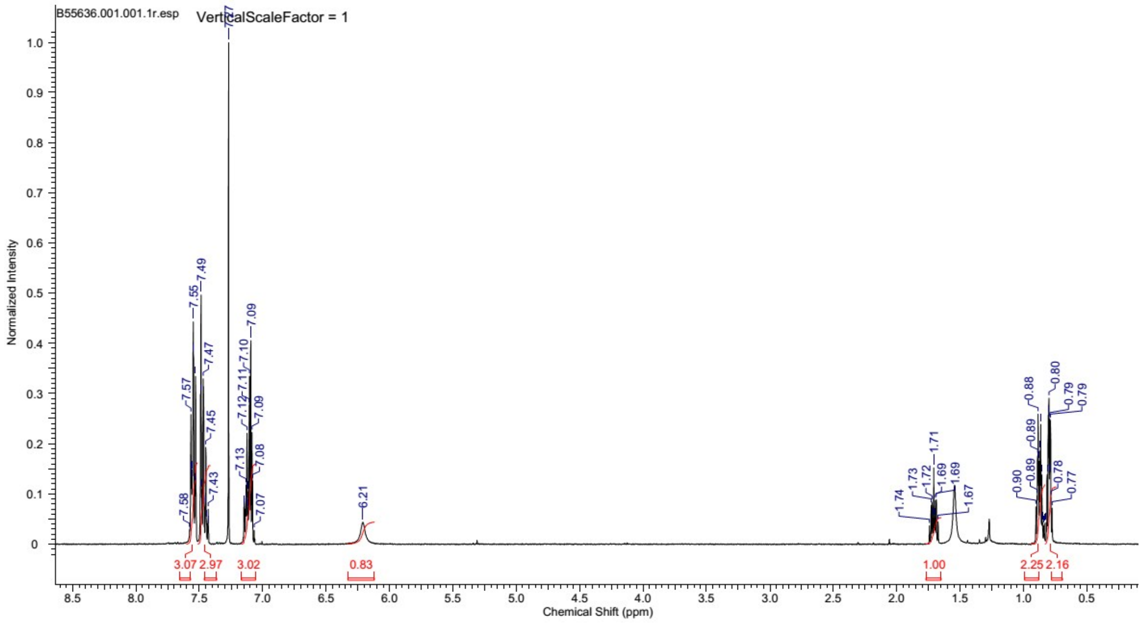

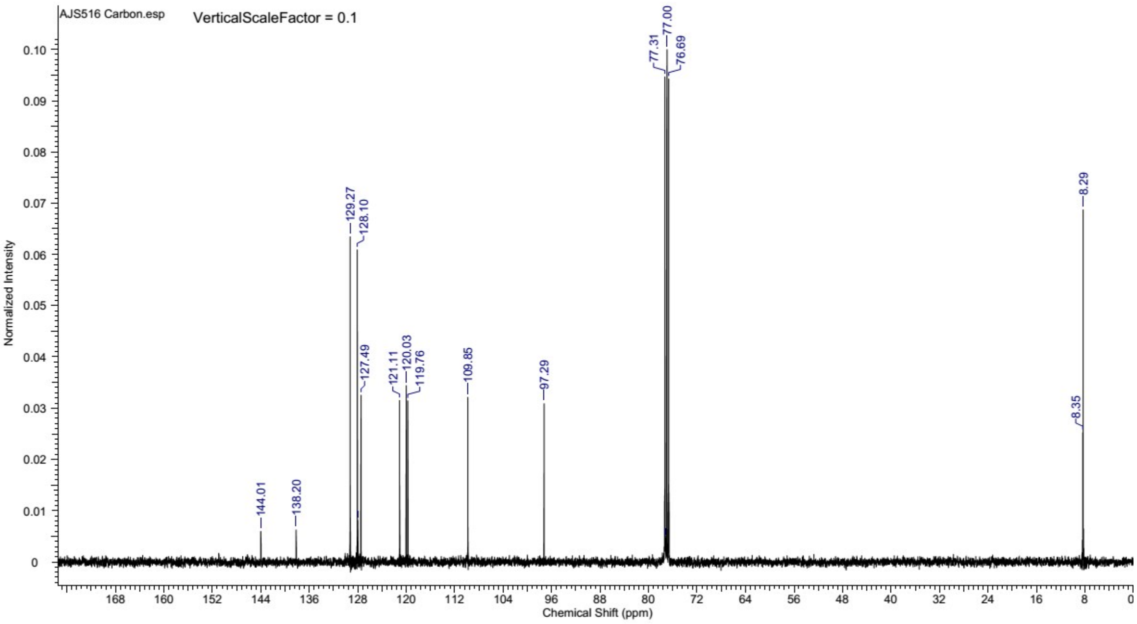

^{1}H and ^{13}C NMR Spectra of (2-(Phenylamino)phenyl)methanol

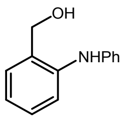

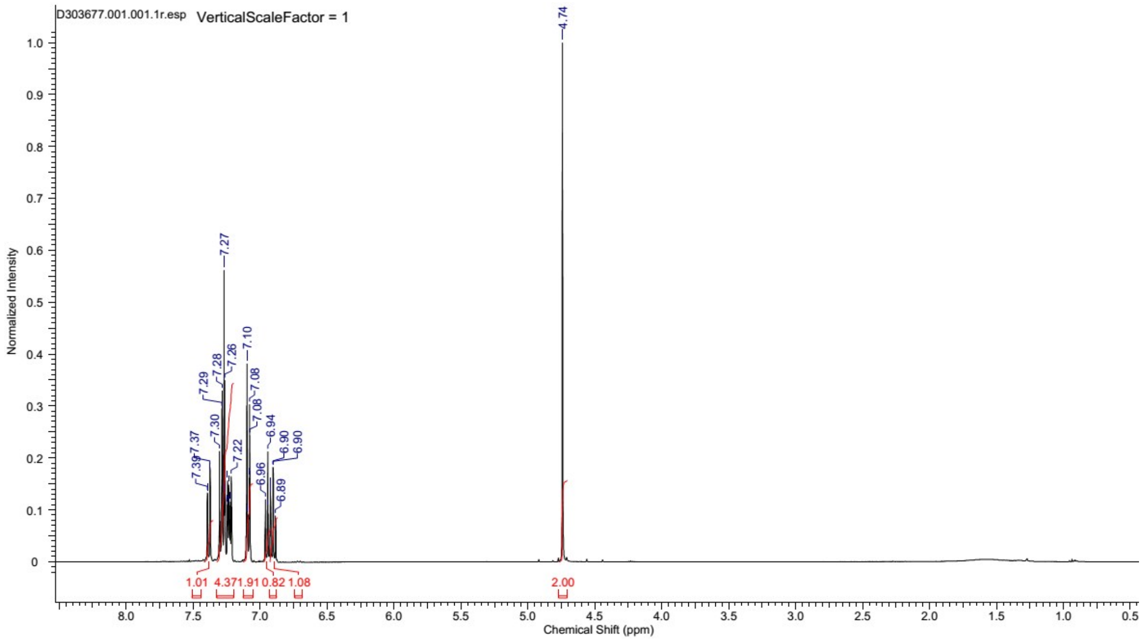

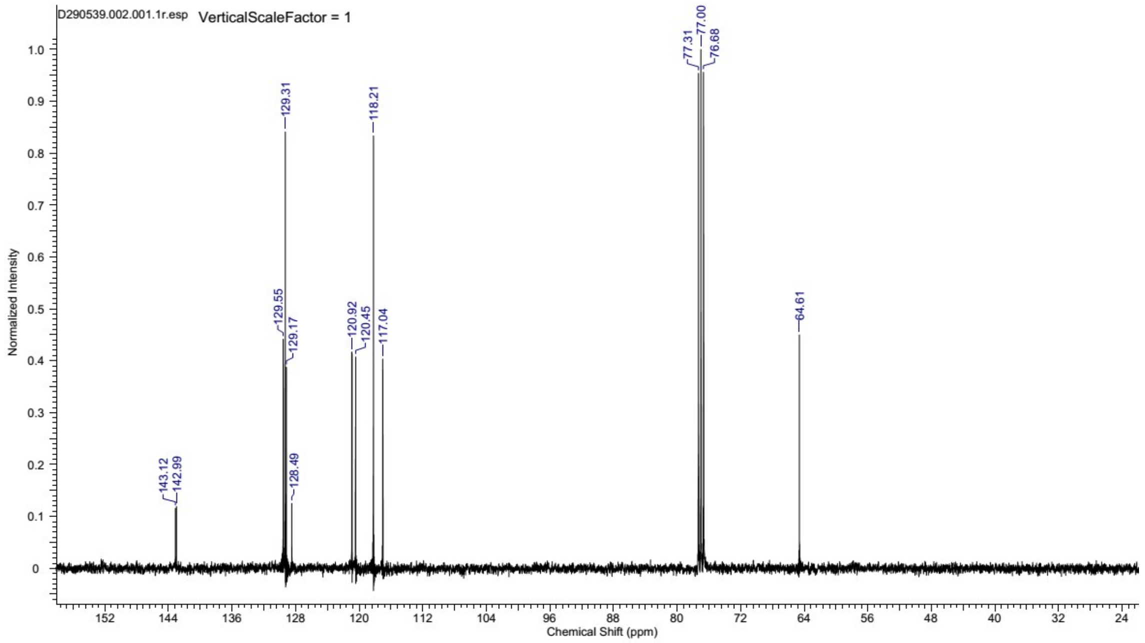

^{1}H and ^{13}C NMR Spectra of 2-(Phenylamino)benzaldehyde

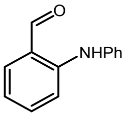

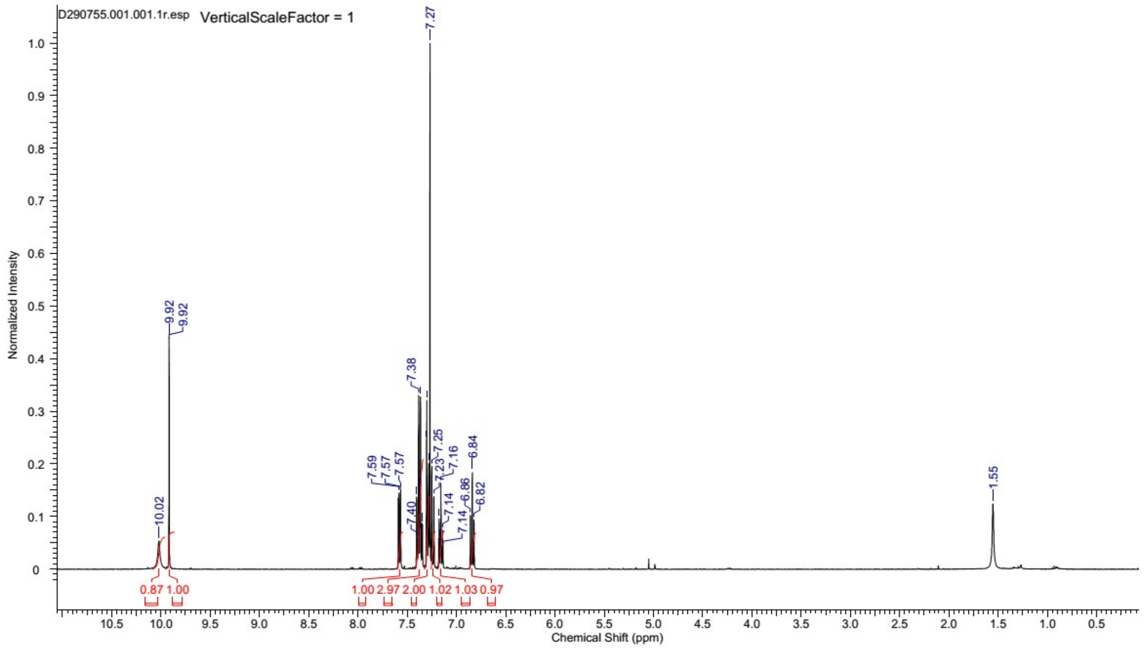

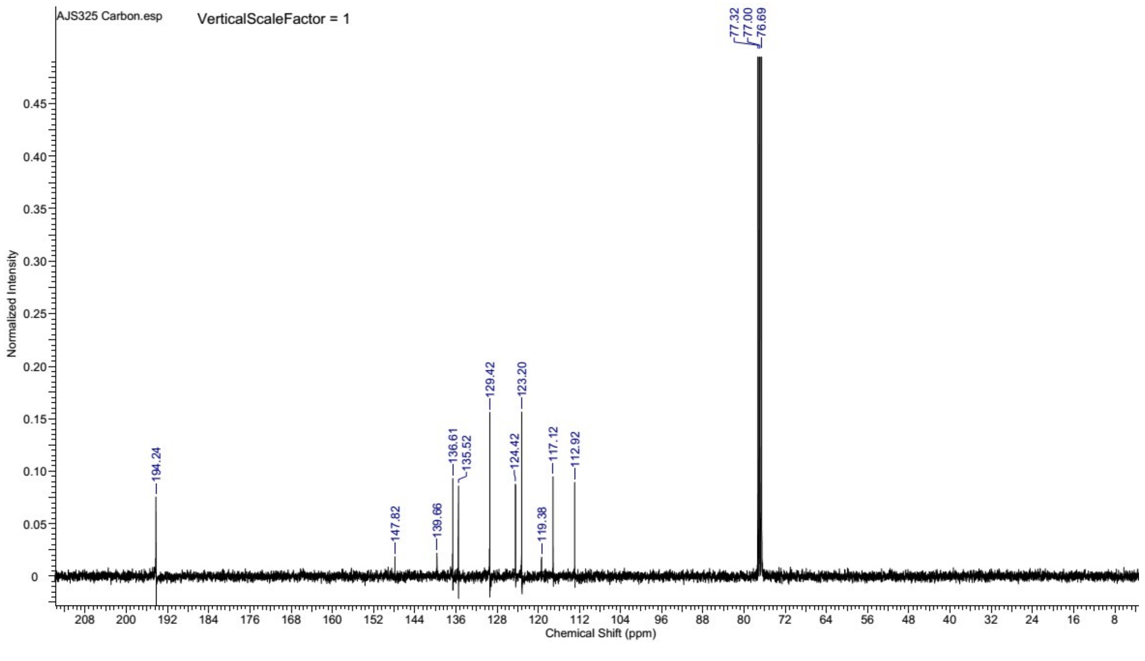

^{1}H and ^{13}C NMR Spectra of *N*-Phenyl-2-vinylaniline (**92**)

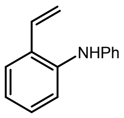

92

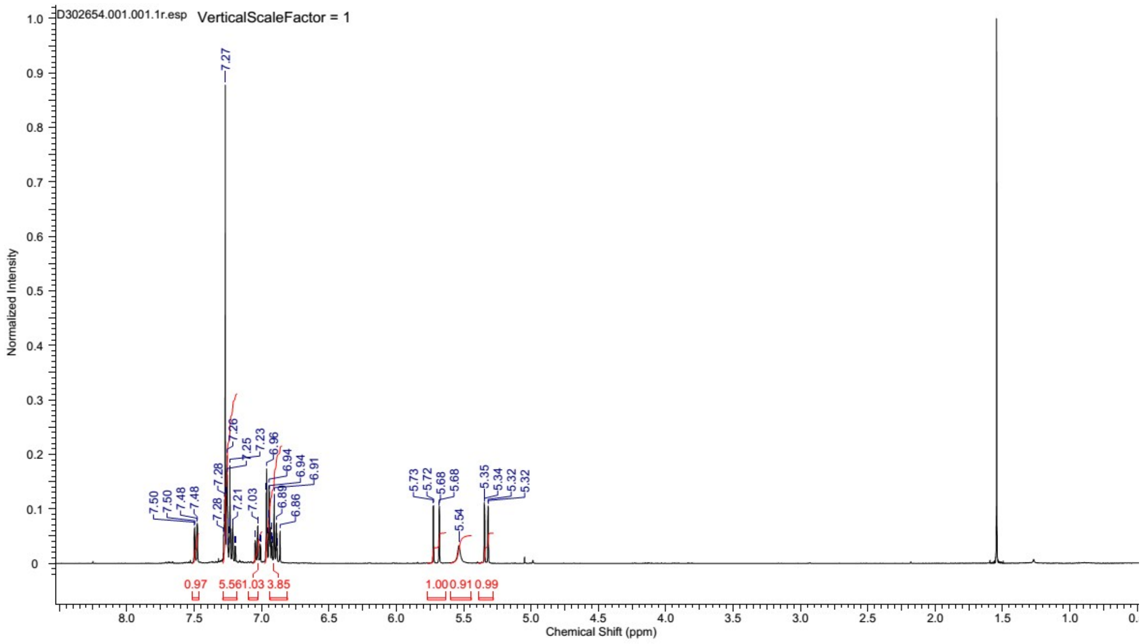

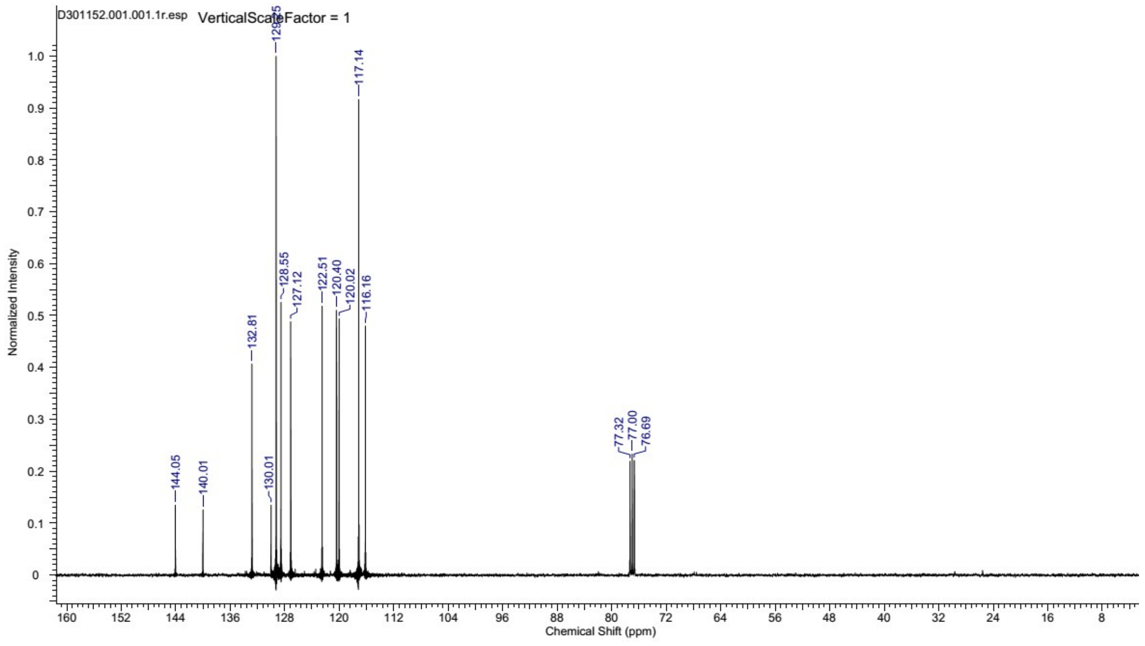

^{1}H and ^{13}C NMR Spectra of 9-Methyl-9,10-dihydroacridine (**30**)

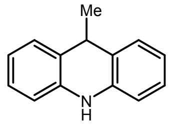

30

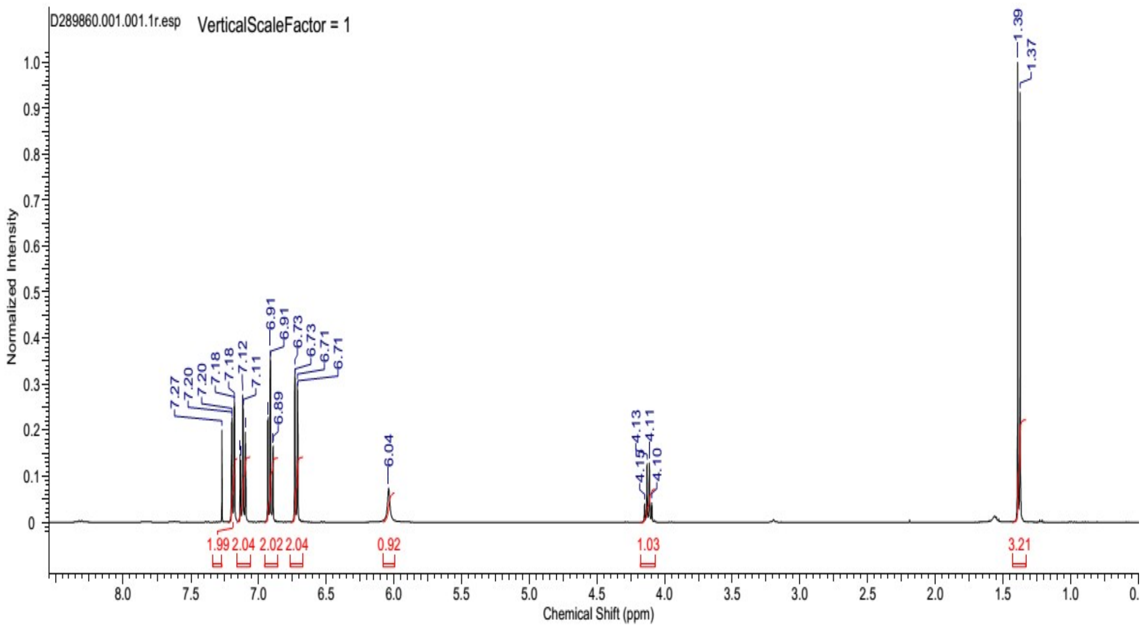

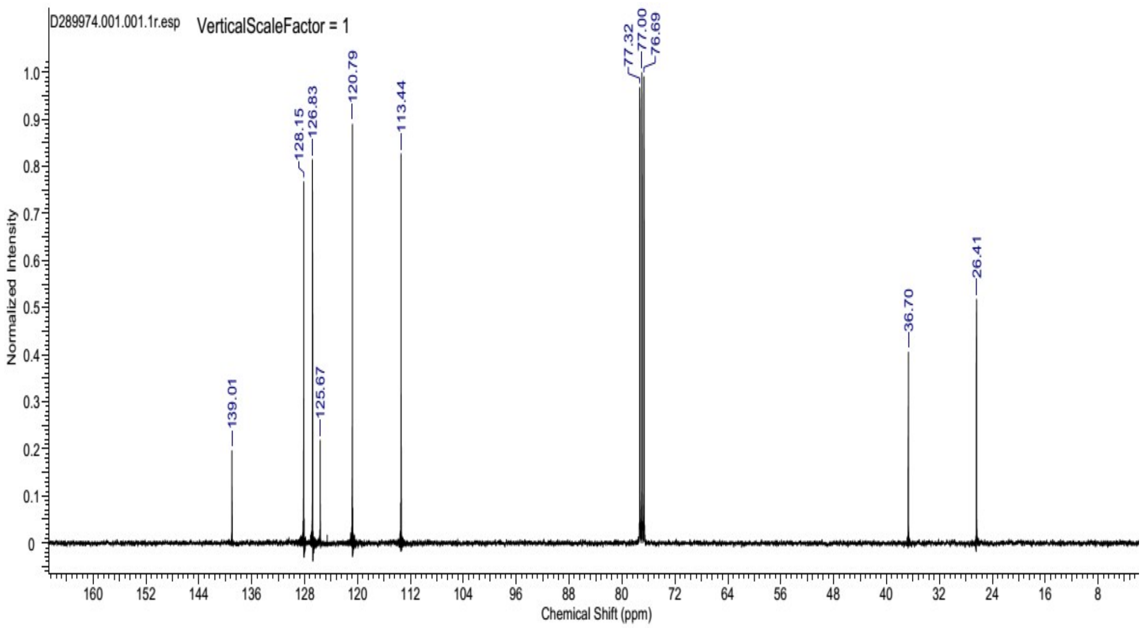

^{1}H and ^{13}C NMR Spectra of 9,9-Dimethyl-9,10-dihydroacridine (**31**)

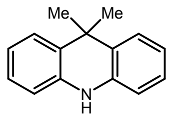

31

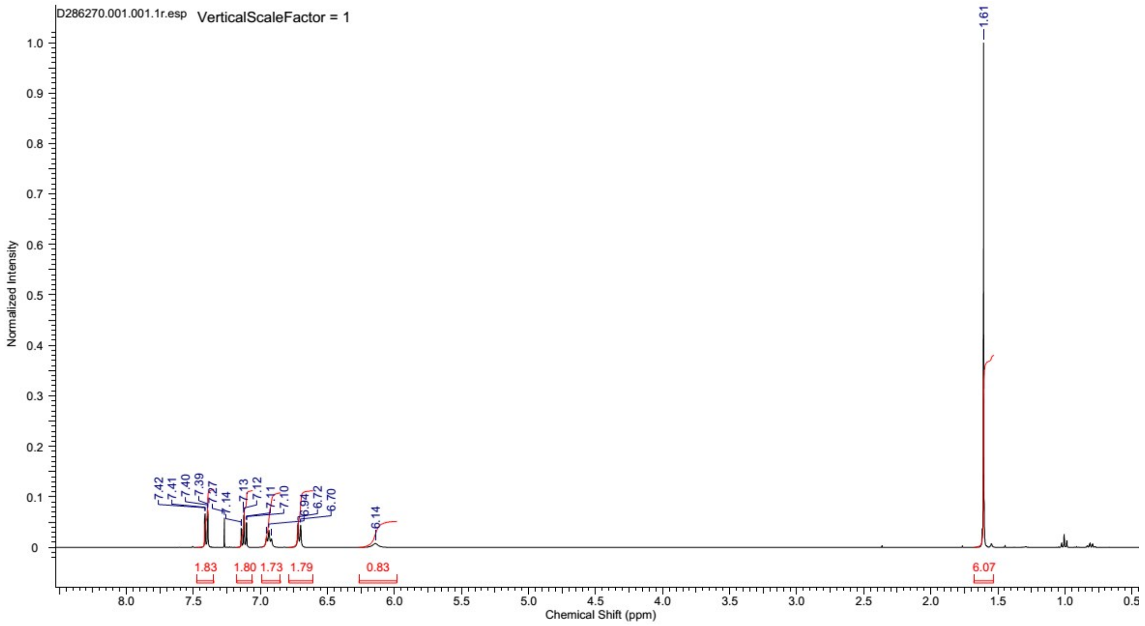

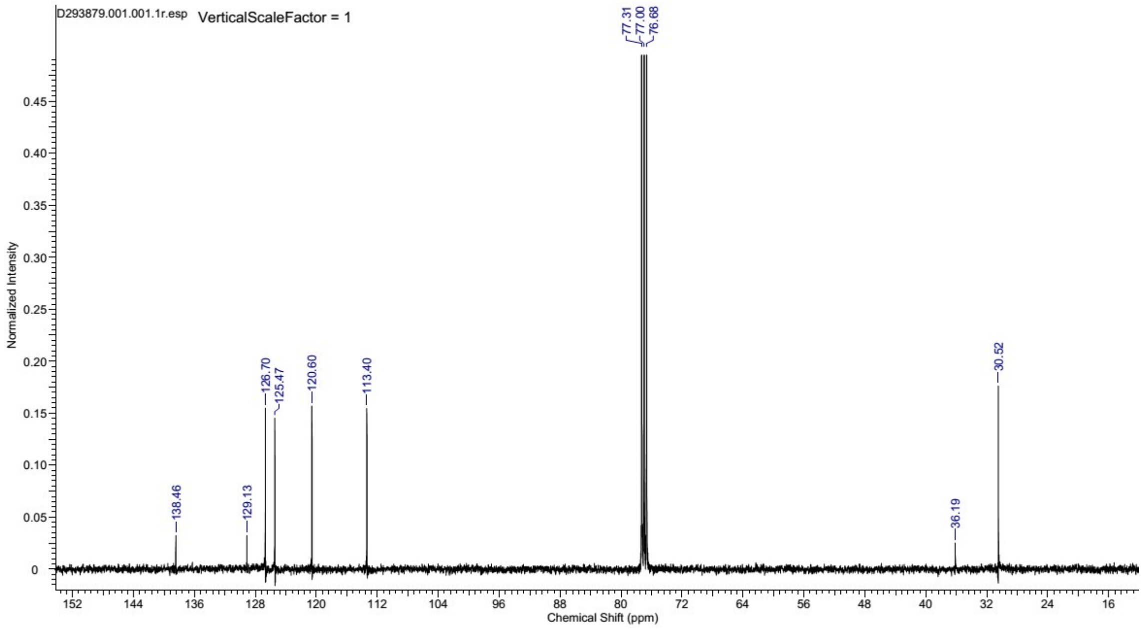

^{1}H and ^{13}C NMR Spectra of 9-Ethyl-9-methyl-9,10-dihydroacridine (**32**)

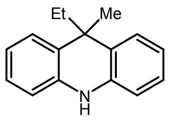

32

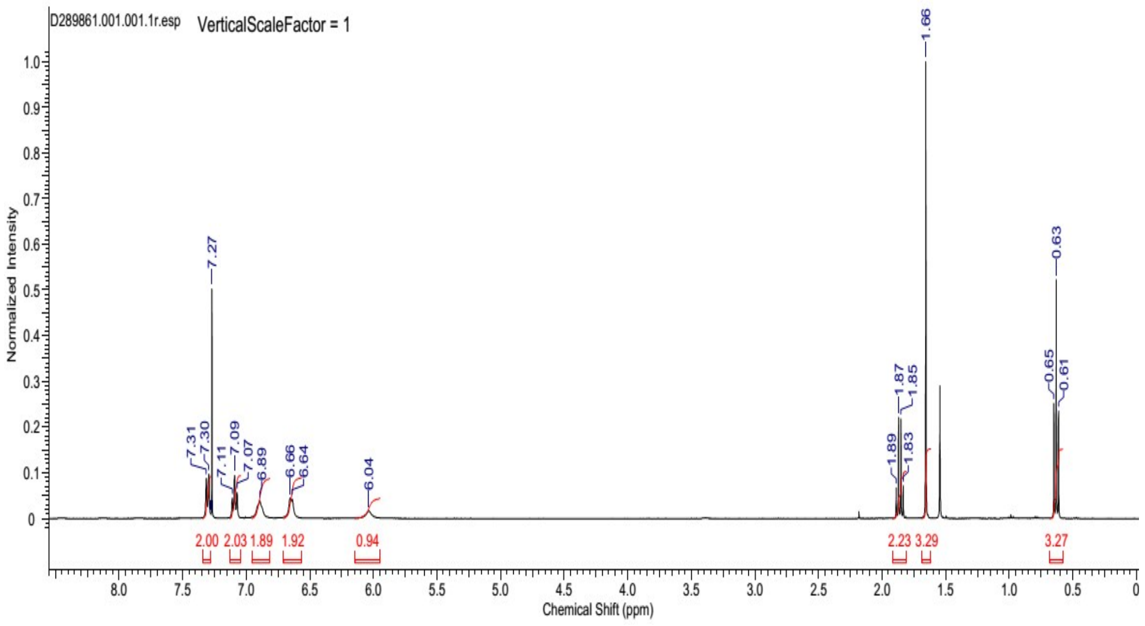

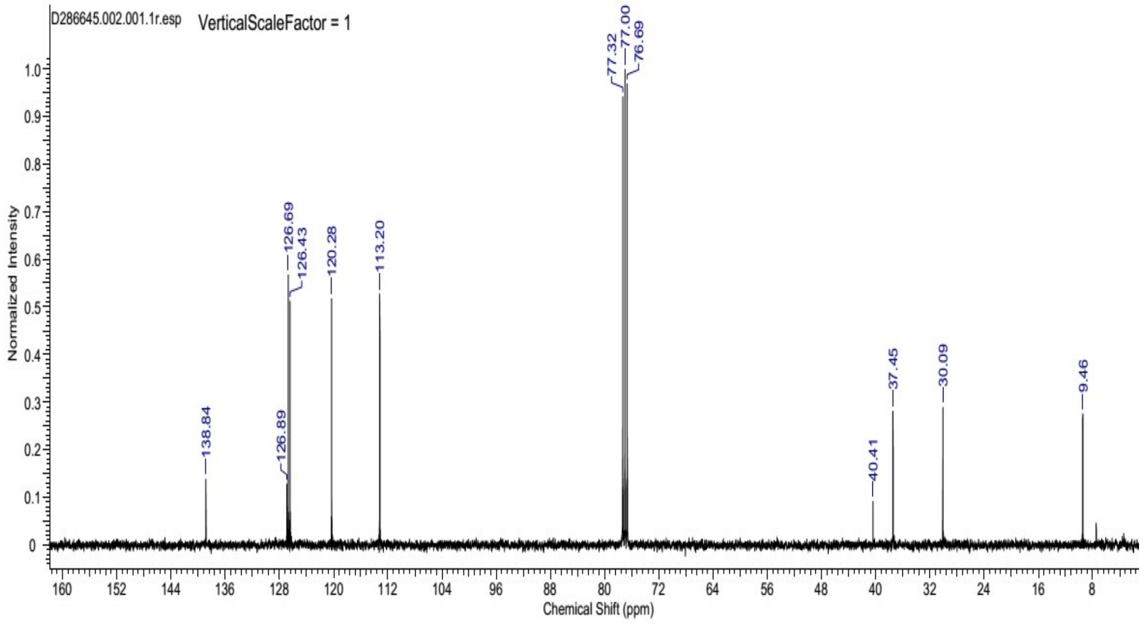

^{1}H and ^{13}C NMR Spectra of 9-Methyl-9-phenyl-9,10-dihydroacridine (**33**)

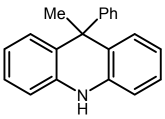

33

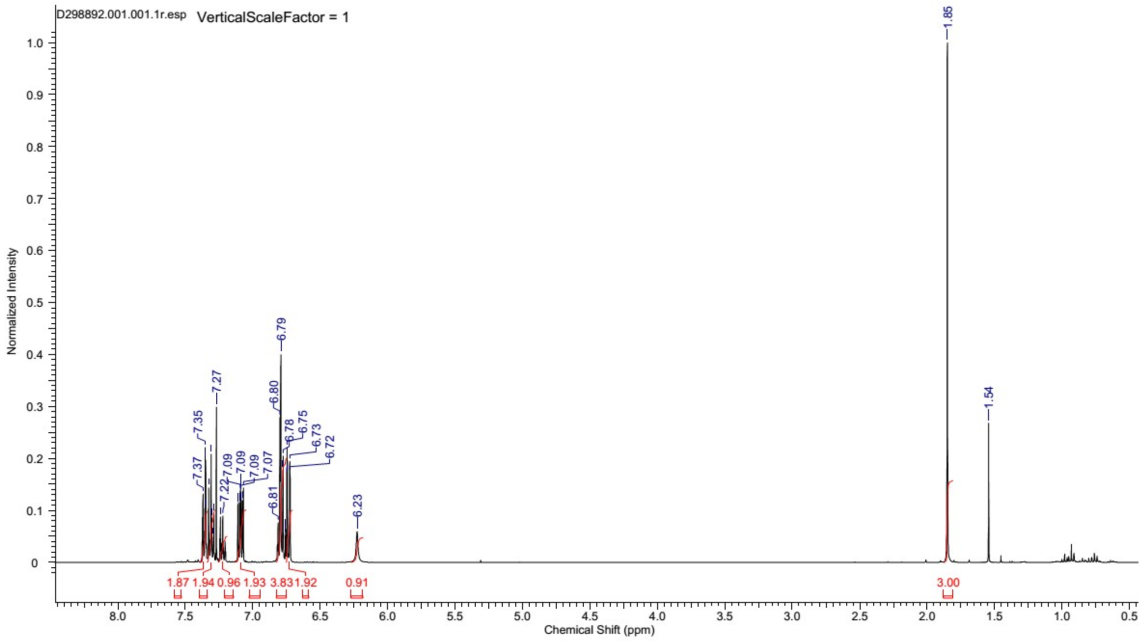

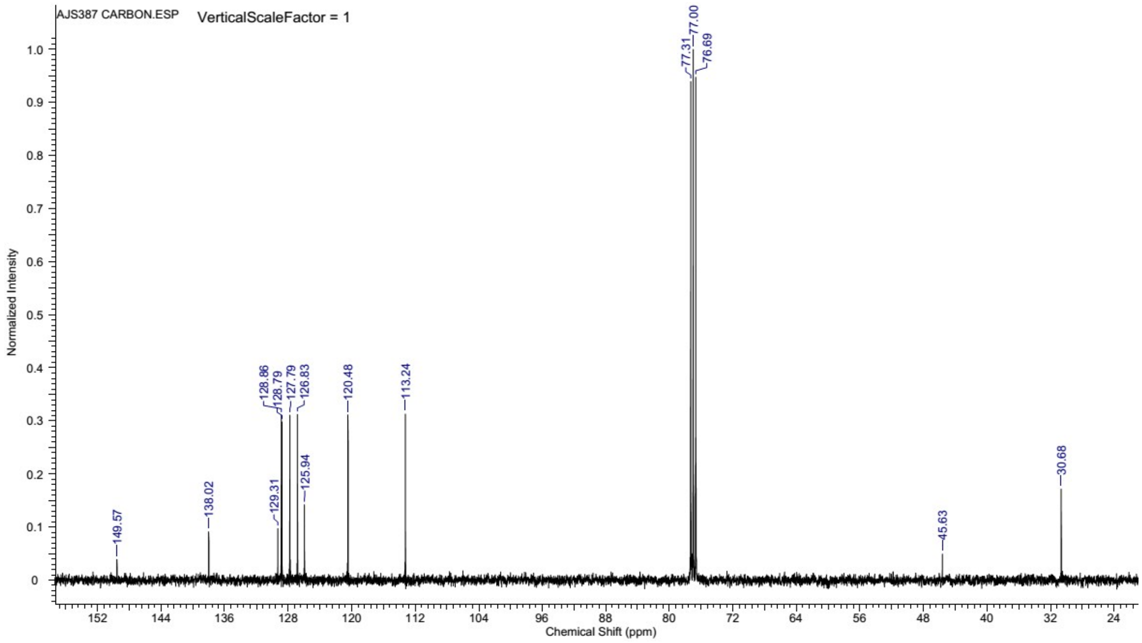

^{1}H and ^{13}C NMR Spectra of 9-Methyl-9-octyl-9,10-dihydroacridine (**34**)

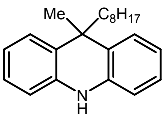

34

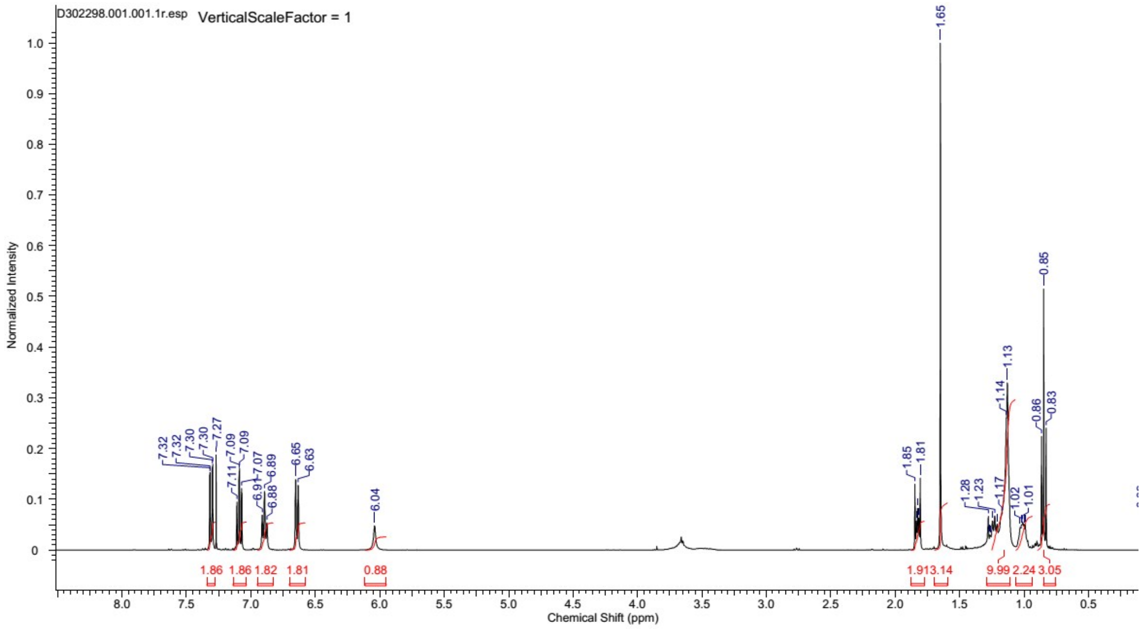

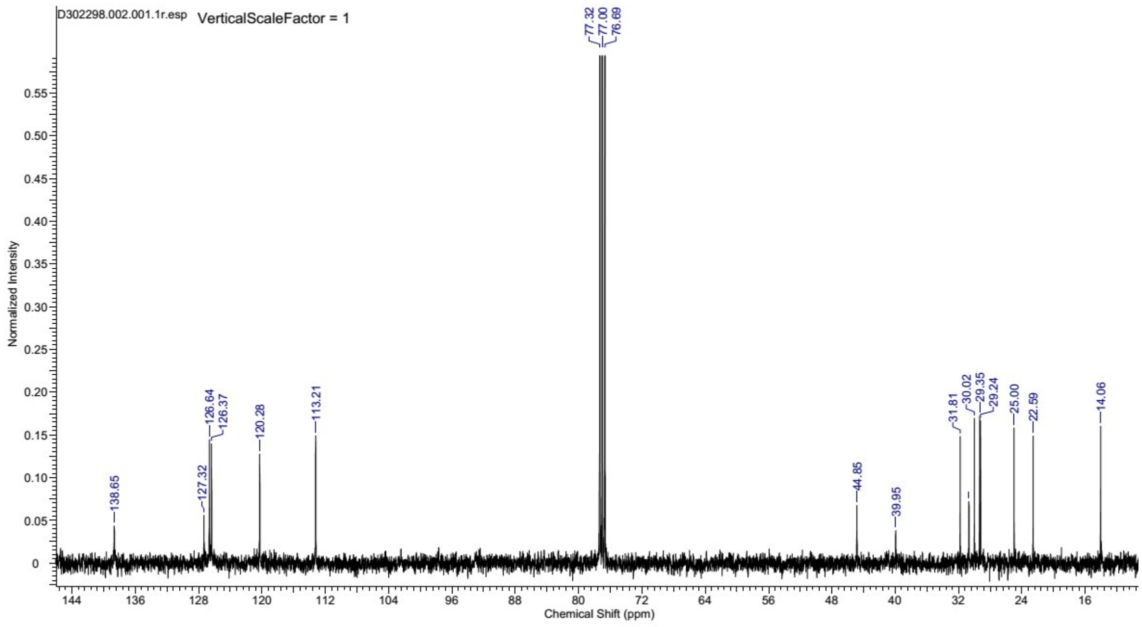

^{1}H and ^{13}C NMR Spectra of 9-Butyl-9-methyl-9,10-dihydroacridine (**35**)

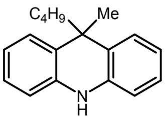

35

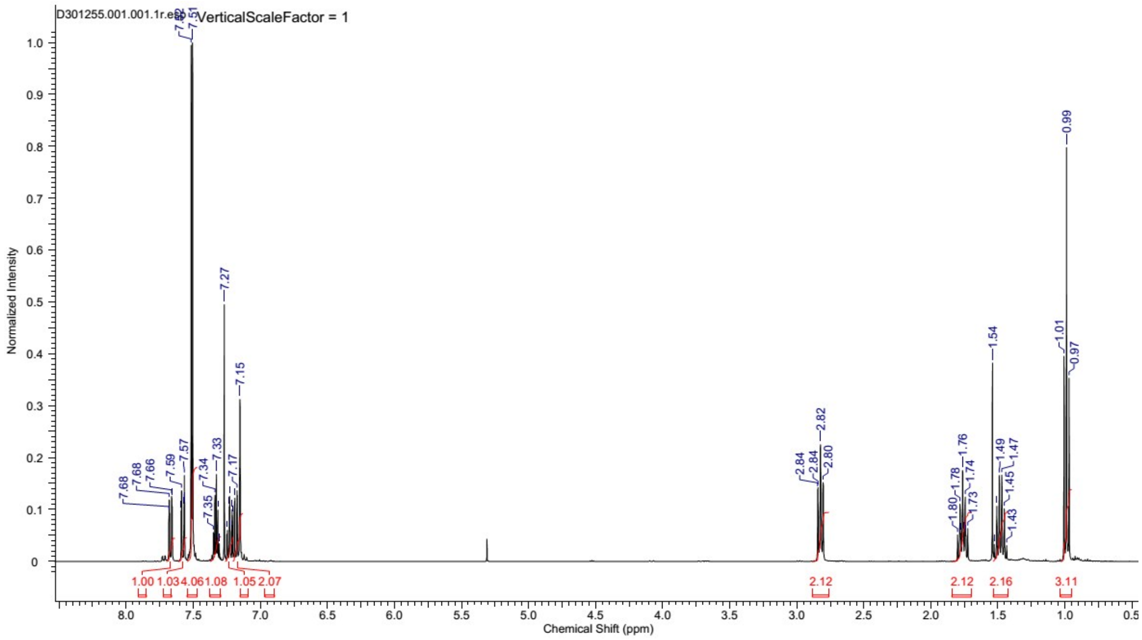

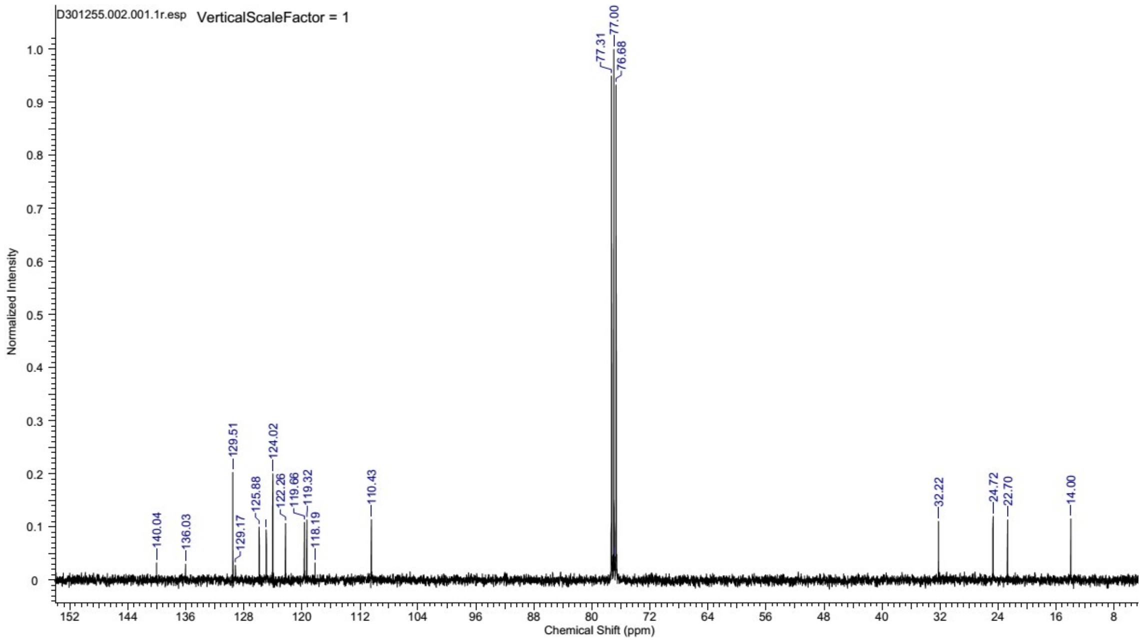

^{1}H and ^{13}C NMR Spectra of 2,9-Dimethyl-9,10-dihydroacridine (**36**)

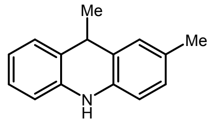

36

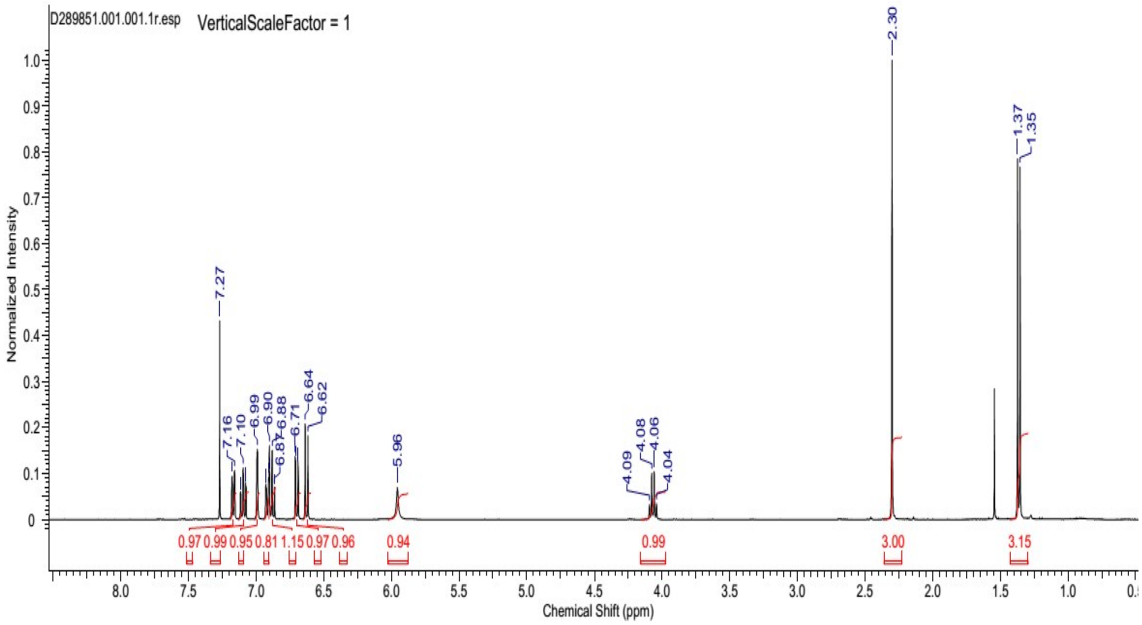

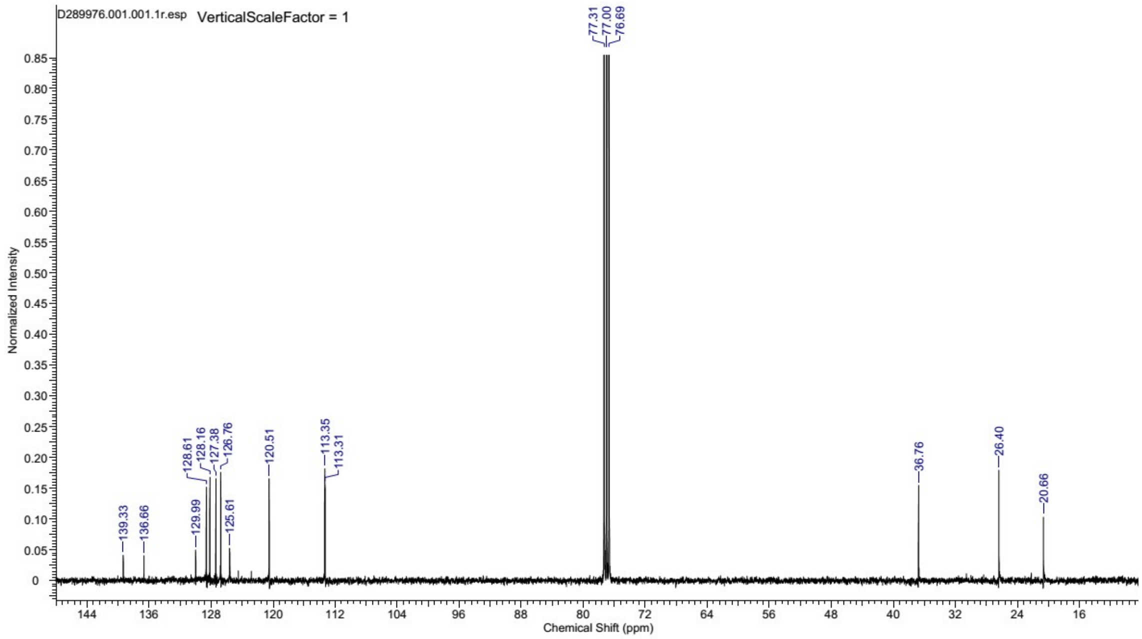

^{1}H and ^{13}C NMR Spectra of (1*R**,2*R**)-2-Methyl-1'-phenylspiro[cyclopentane-1,3'-indoline] (**52**)

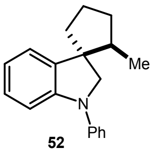

52

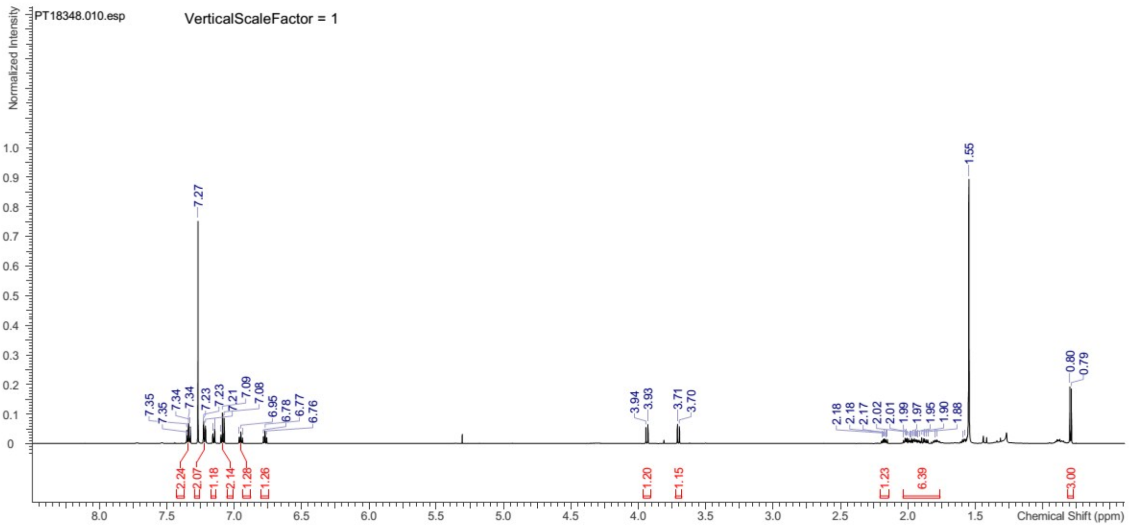

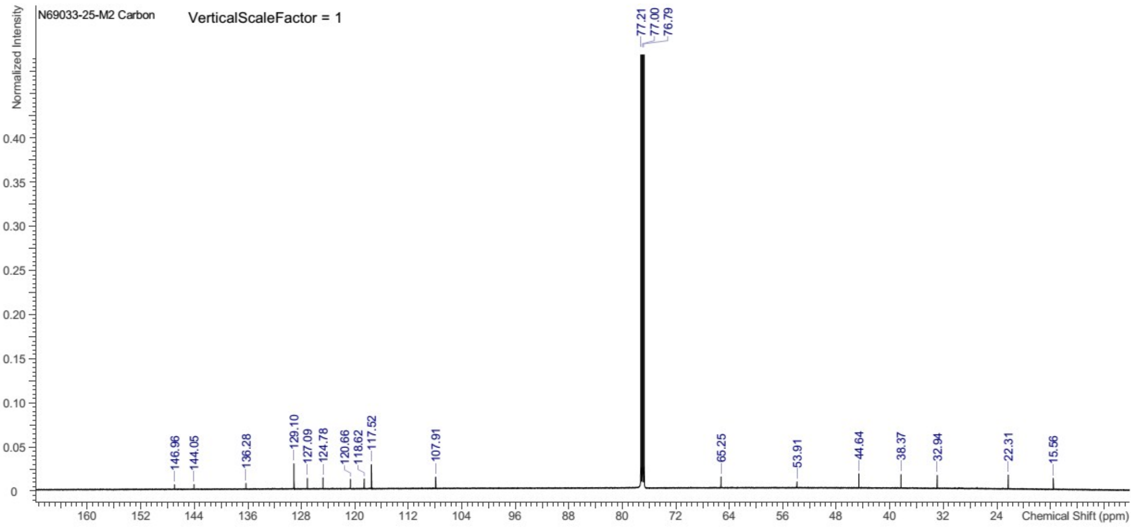

COSY NMR Spectra of (1*R**,2*R**)-2-Methyl-1'-phenylspiro[cyclopentane-1,3'-indoline] (52)

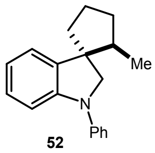

52

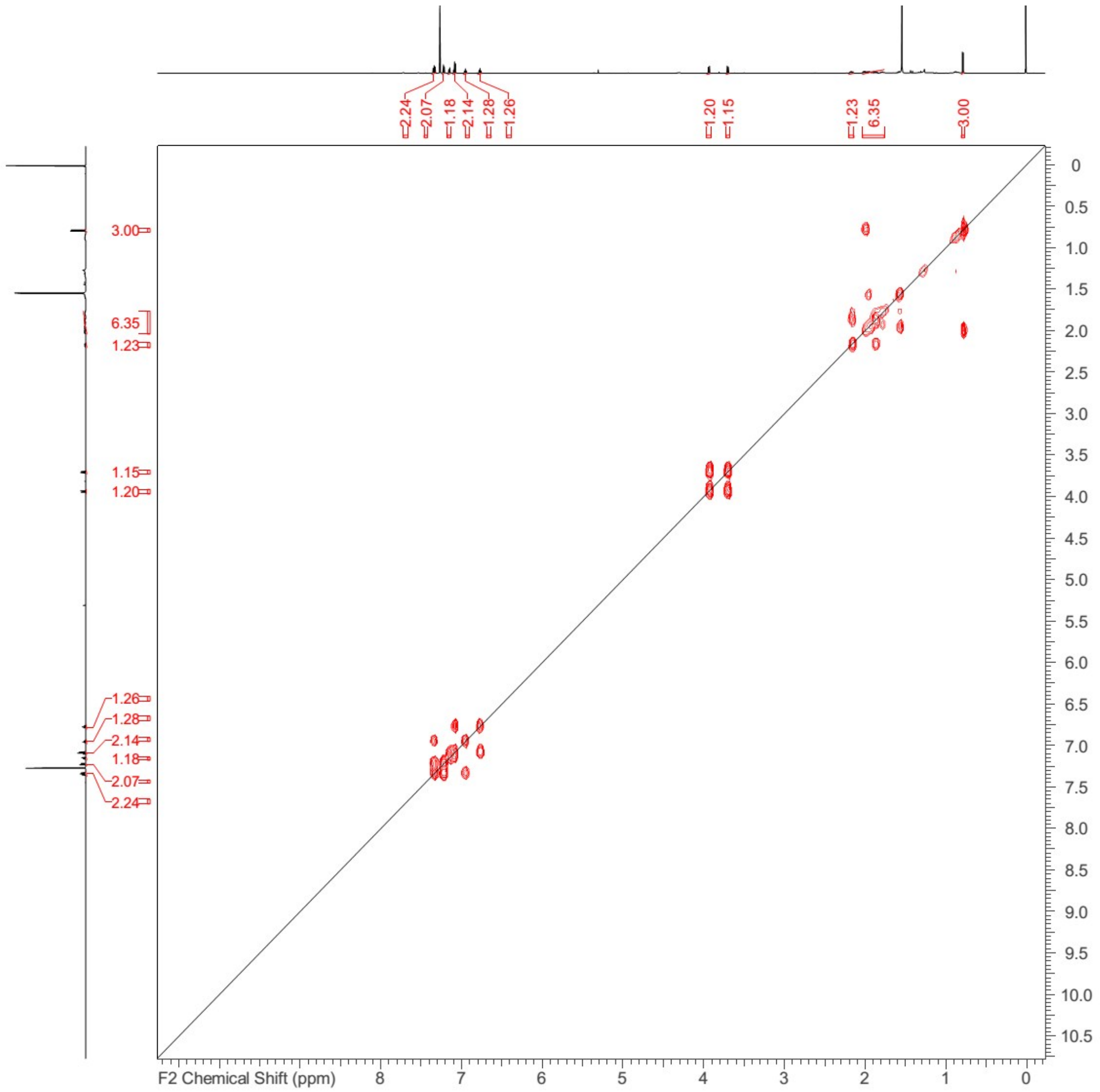

HSQC NMR Spectra of (1*R**,2*R**)-2-Methyl-1'-phenylspiro[cyclopentane-1,3'-indoline] (52)

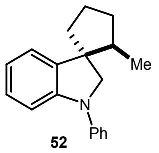

52

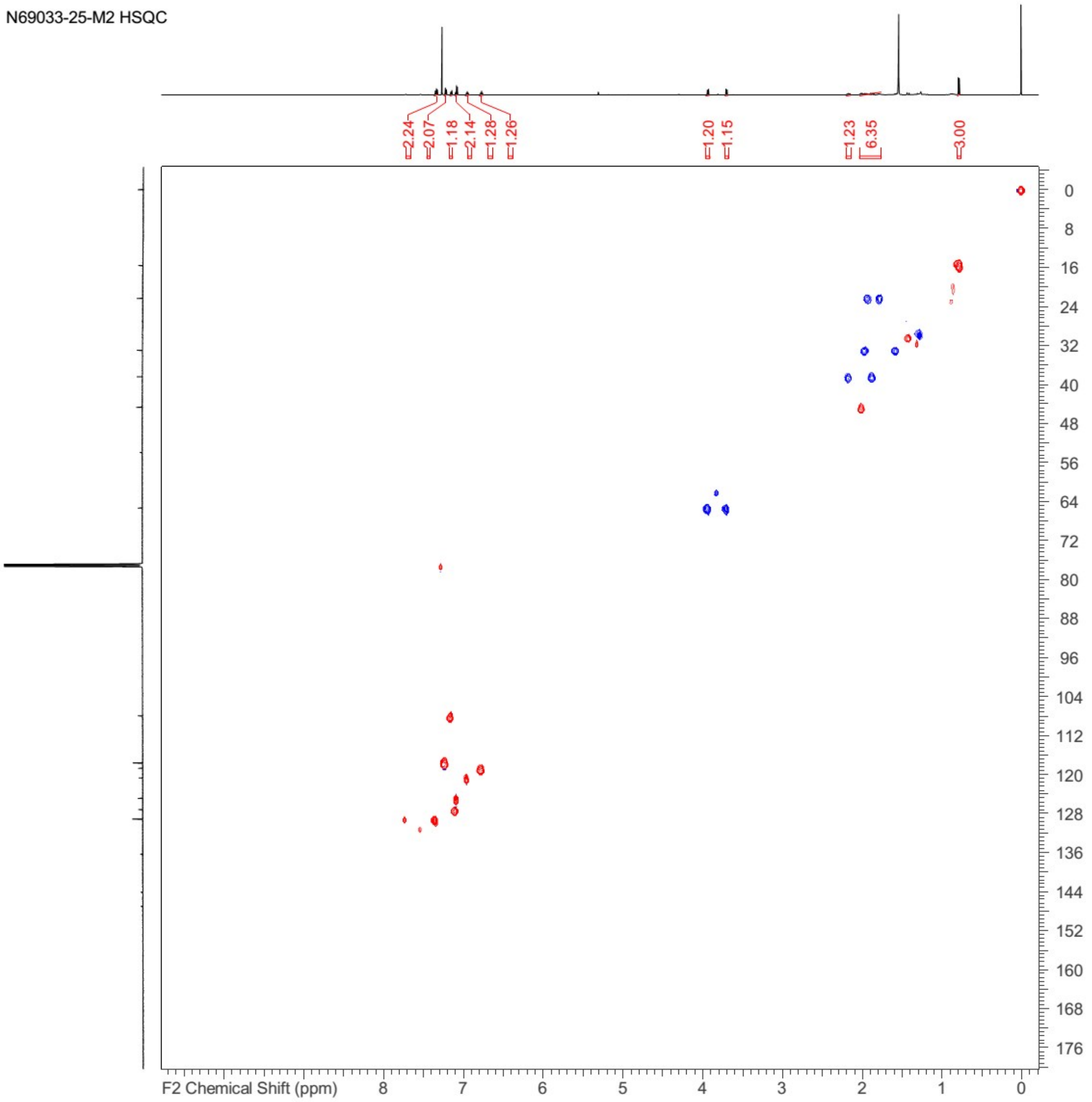

HMBC NMR Spectra of (1*R**,2*R**)-2-Methyl-1'-phenylspiro[cyclopentane-1,3'-indoline] (52)

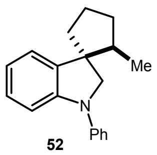

52

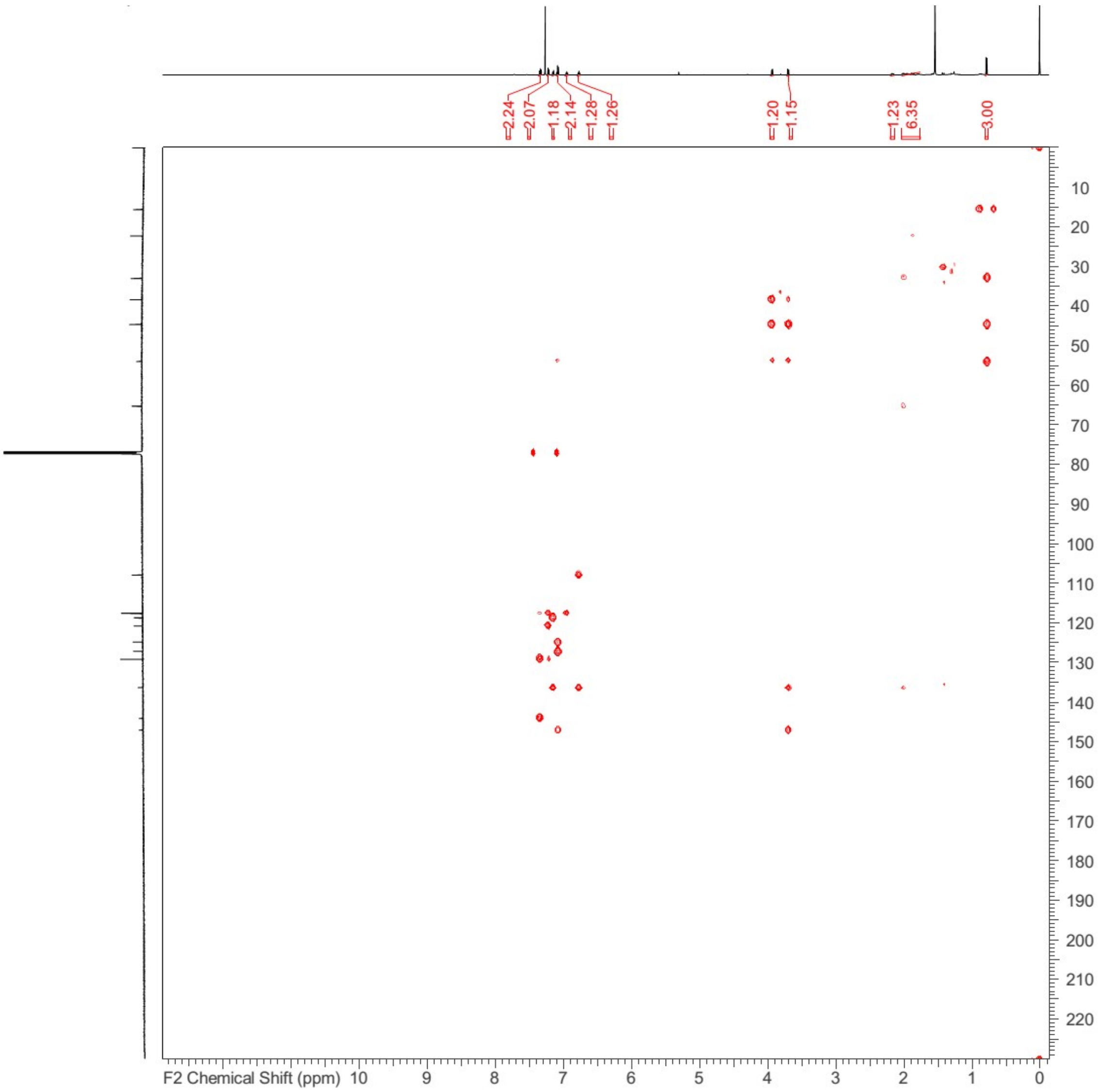

^{1}H and ^{13}C NMR Spectra of (1*R**,2*S**)-2-Methyl-1'-phenylspiro[cyclopentane-1,3'-indoline] (**53**)

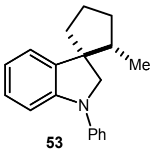

53

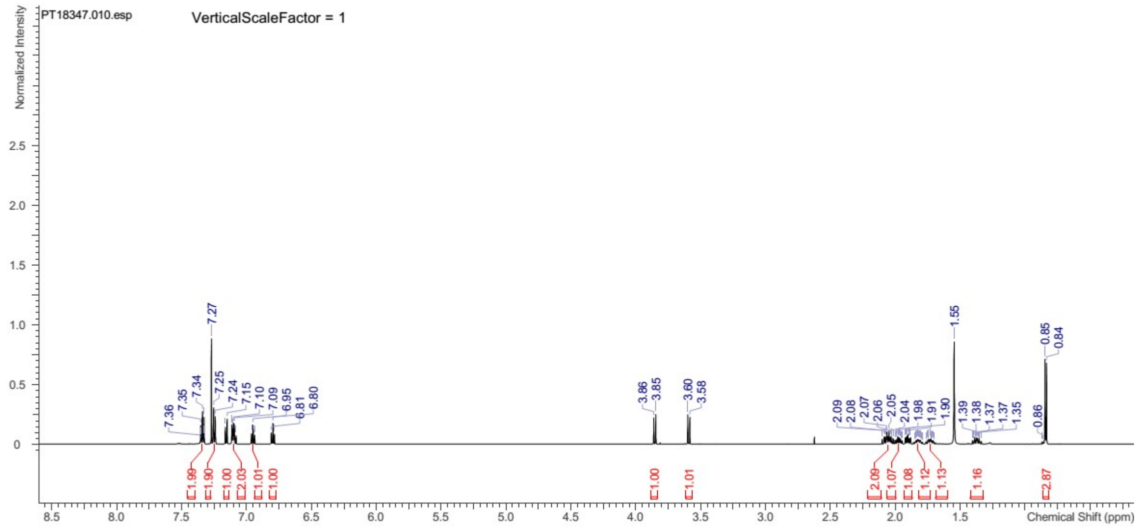

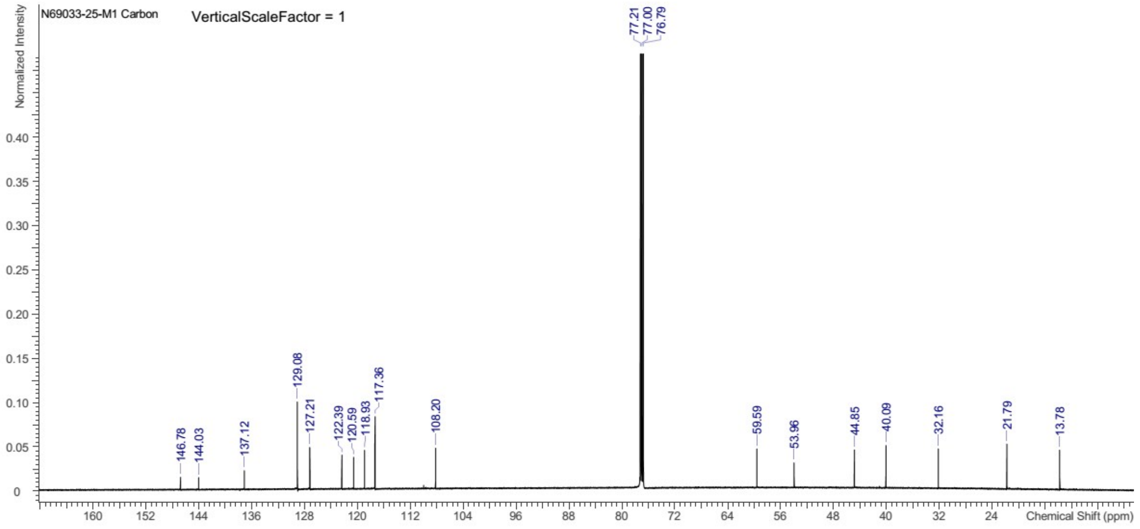

COSY NMR Spectrum of (1*R**,2*S**)-2-Methyl-1'-phenylspiro[cyclopentane-1,3'-indoline] (53)

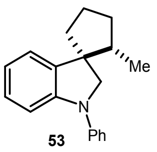

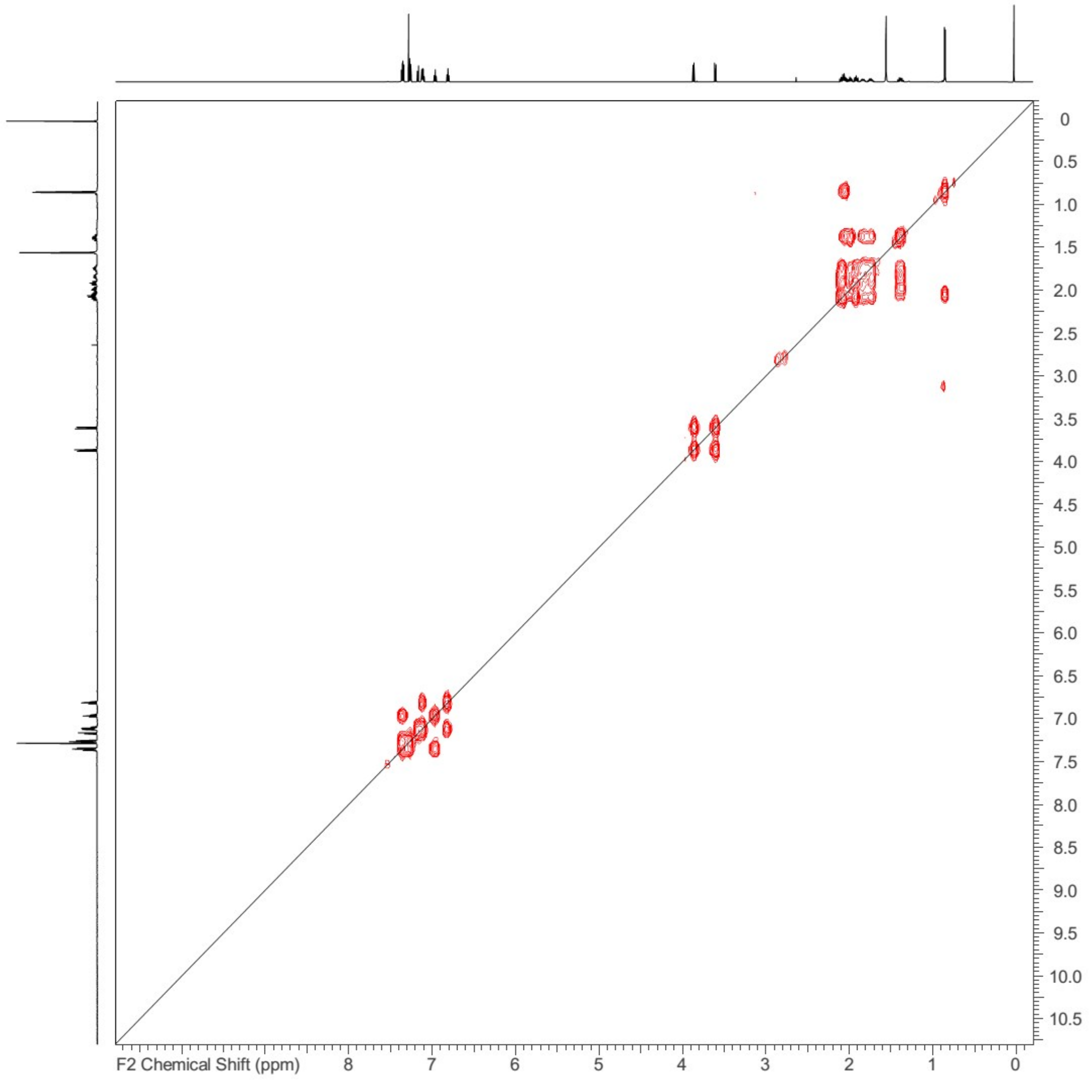

HSQC NMR Spectrum of (1*R**,2*S**)-2-Methyl-1'-phenylspiro[cyclopentane-1,3'-indoline] (53)

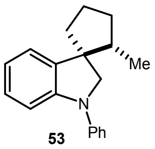

53

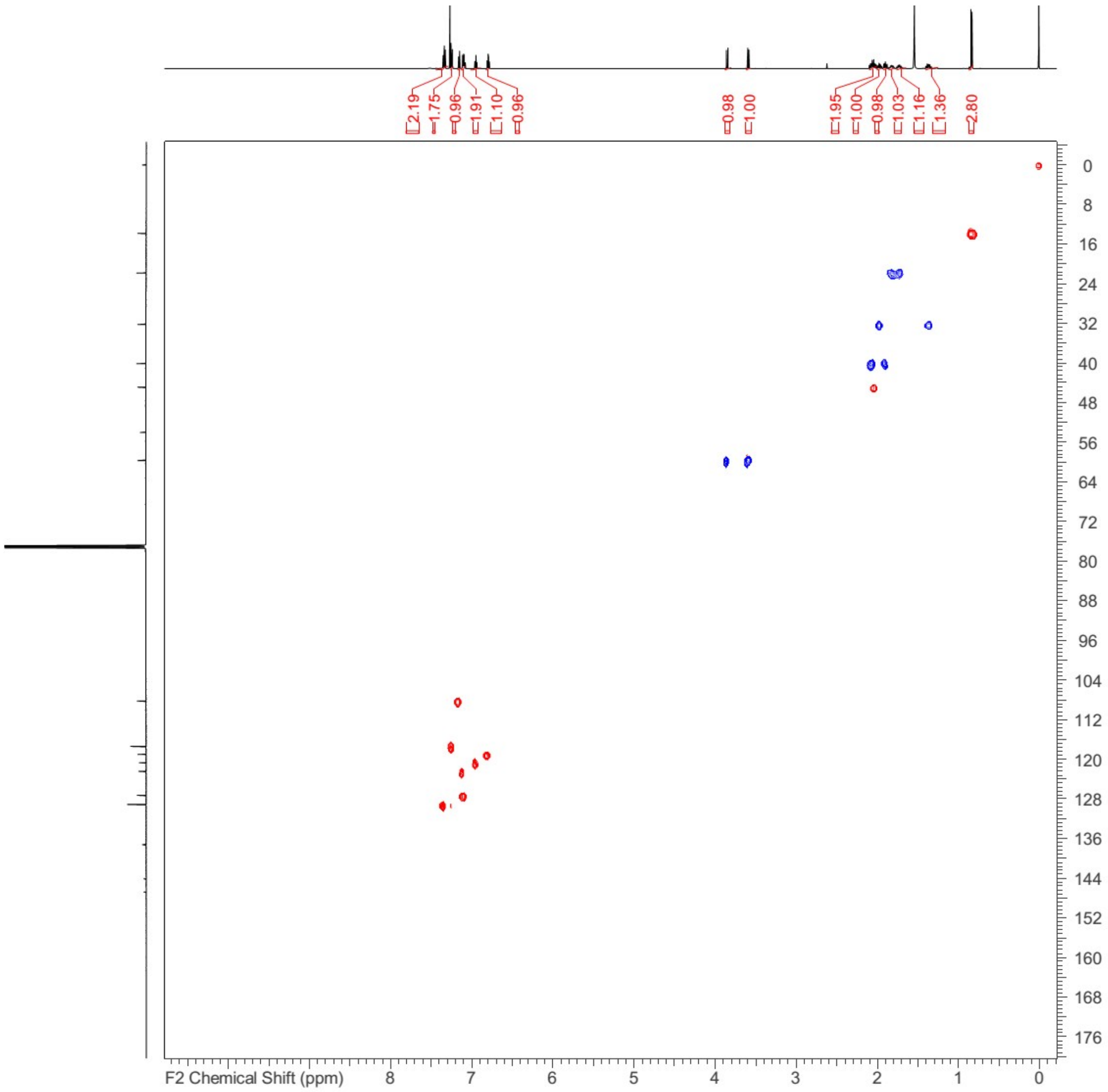

HMBC NMR Spectrum of (1*R**,2*S**)-2-Methyl-1'-phenylspiro[cyclopentane-1,3'-indoline] (53)

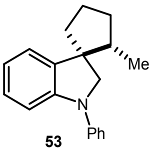

53

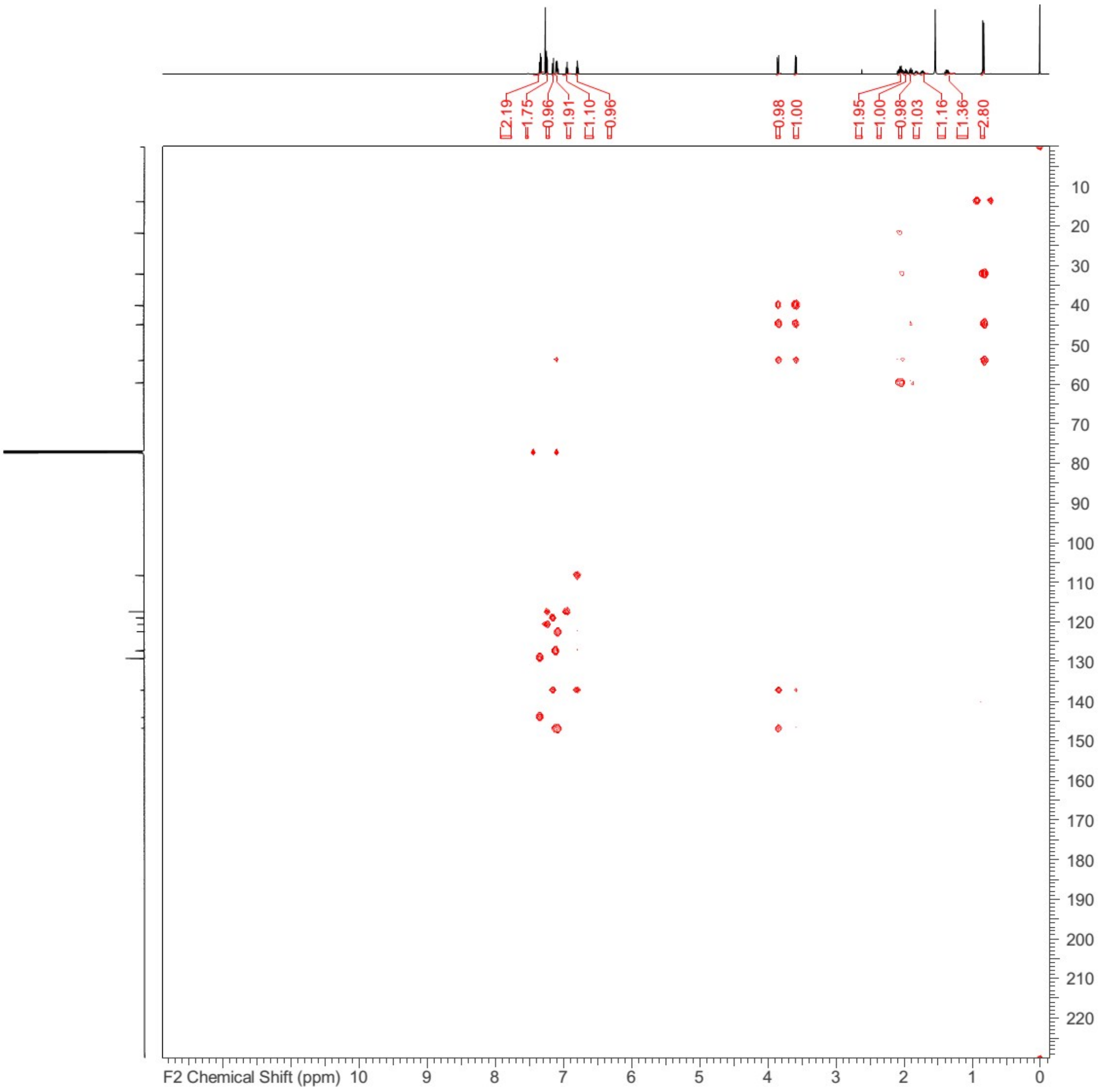

^{1}H and ^{13}C NMR Spectra of the Mixture of 9-Cyclopropyl-9-methyl-9,10-dihydroacridine (**62**) and 9-Methyl-9-propyl-9,10-dihydroacridine (**63**)

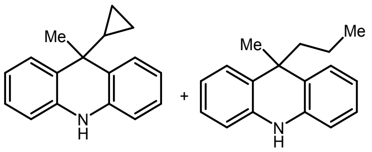

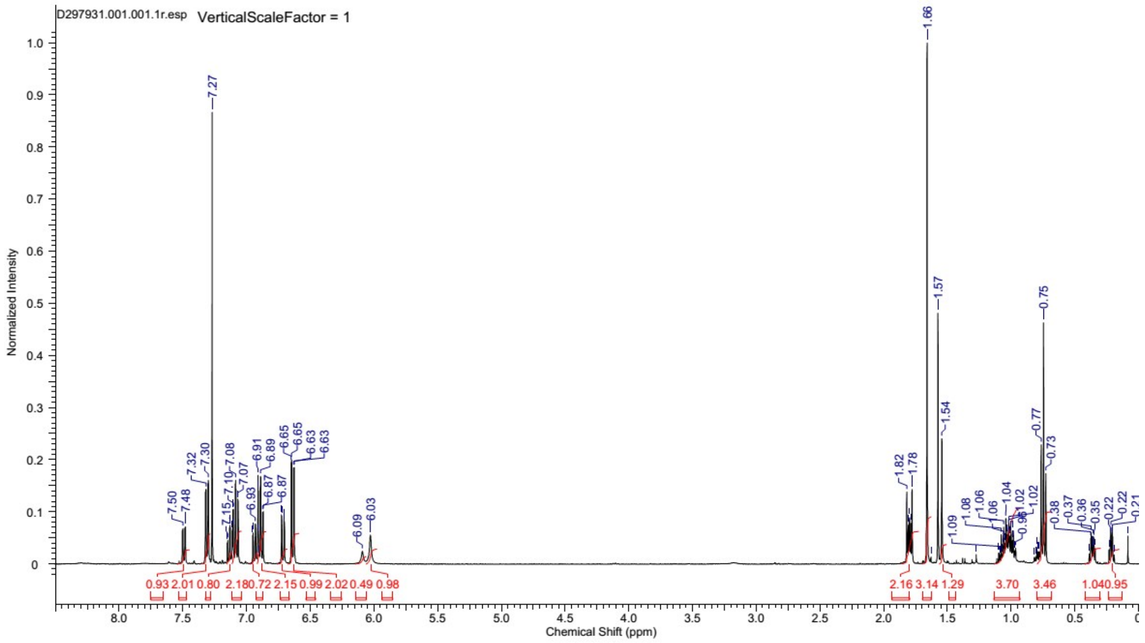

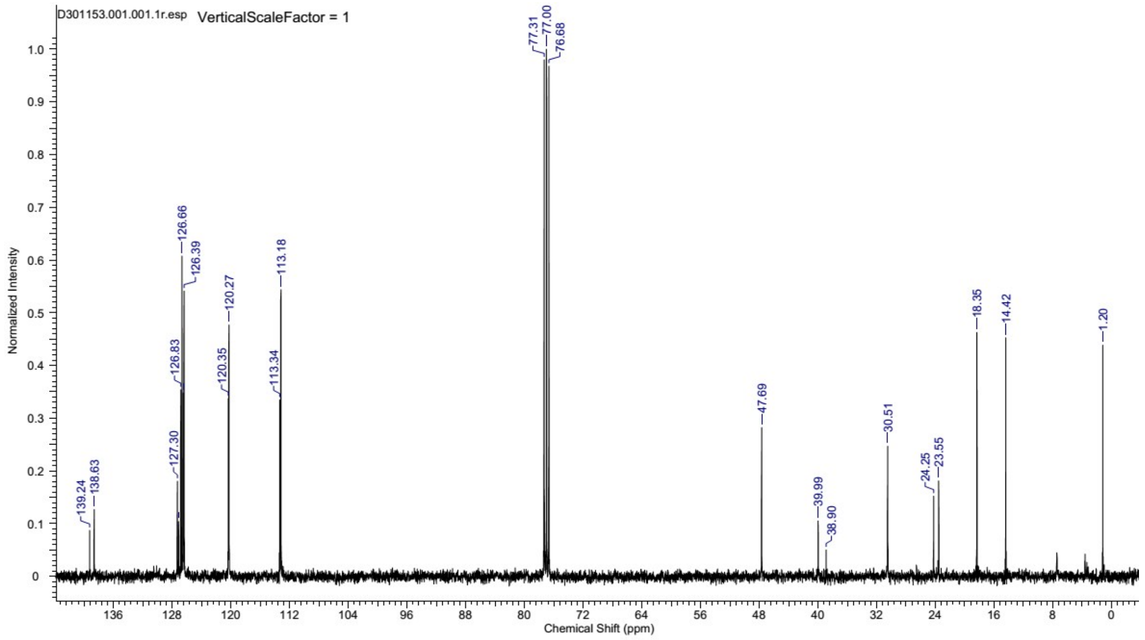

^{1}H and ^{13}C NMR Spectra of Acridin-9-yl(cyclopropyl)methanone (**76**)

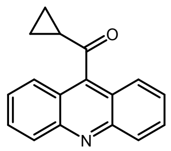

76

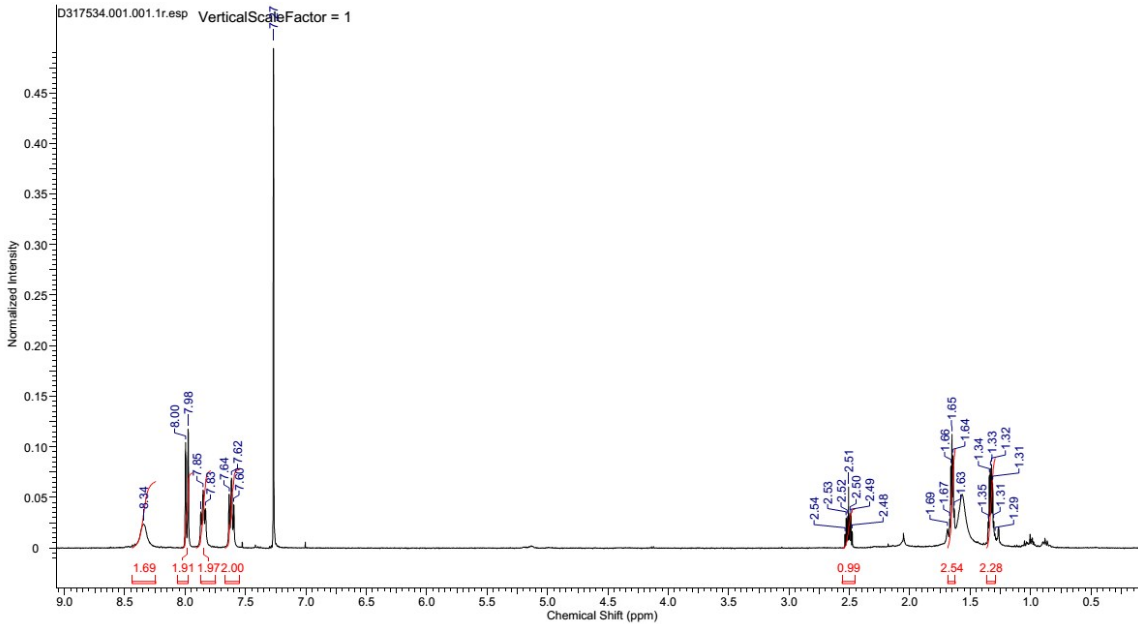

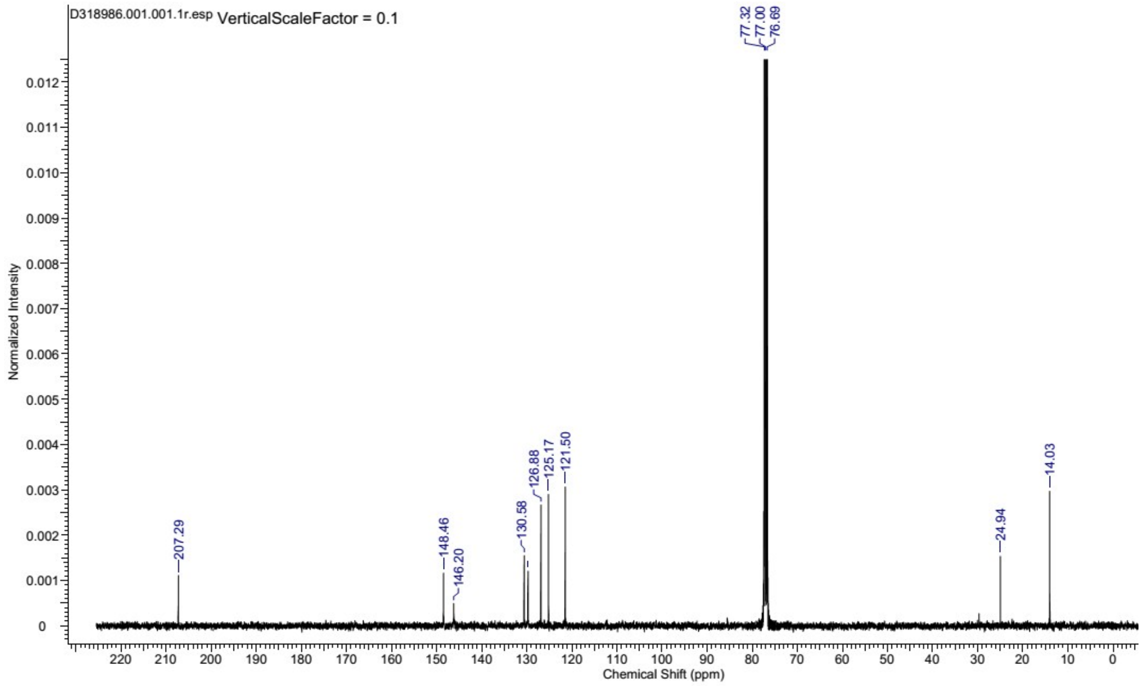

^{1}H and ^{13}C NMR Spectra of 9-Methylacridine (**96**)

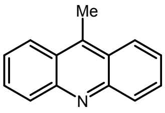

96

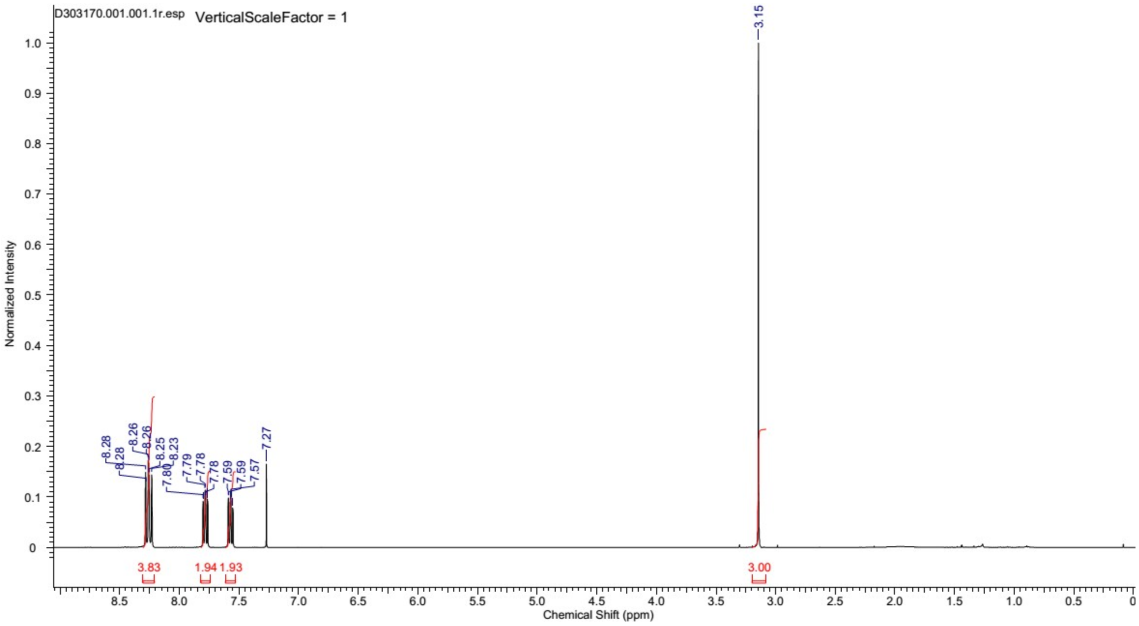

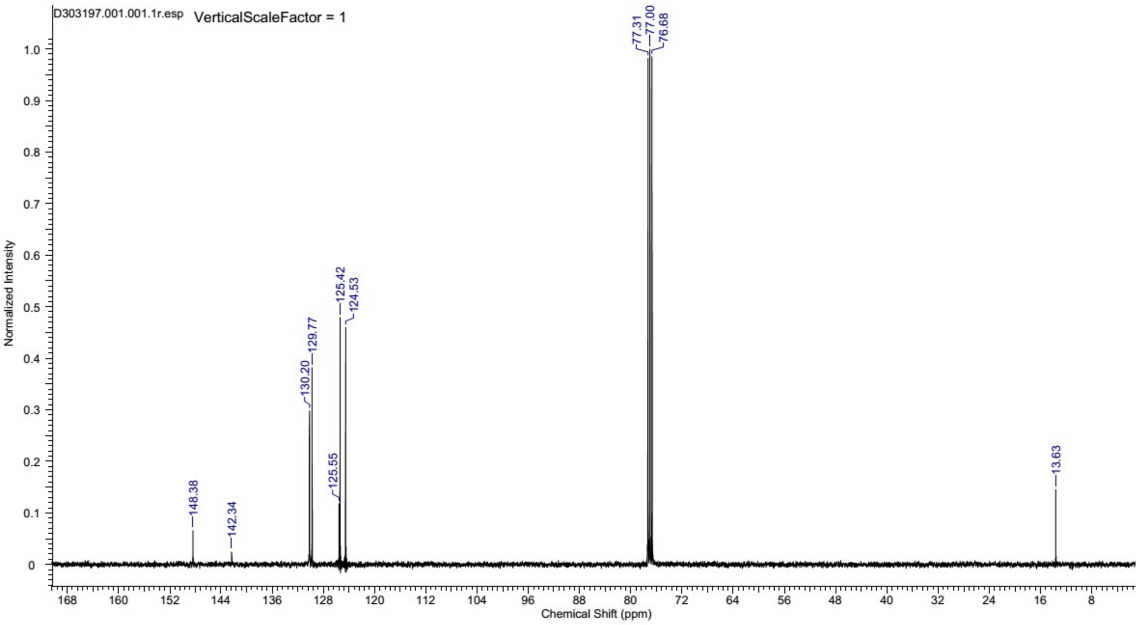

^{1}H and ^{13}C NMR Spectra of Indole (**100**)

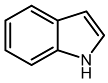

100

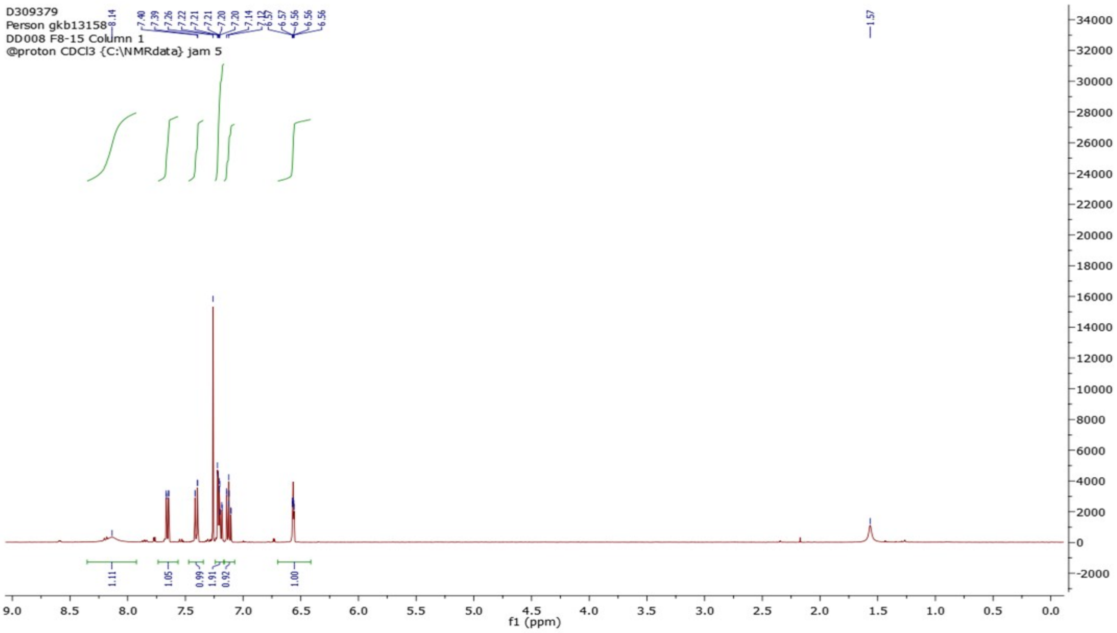

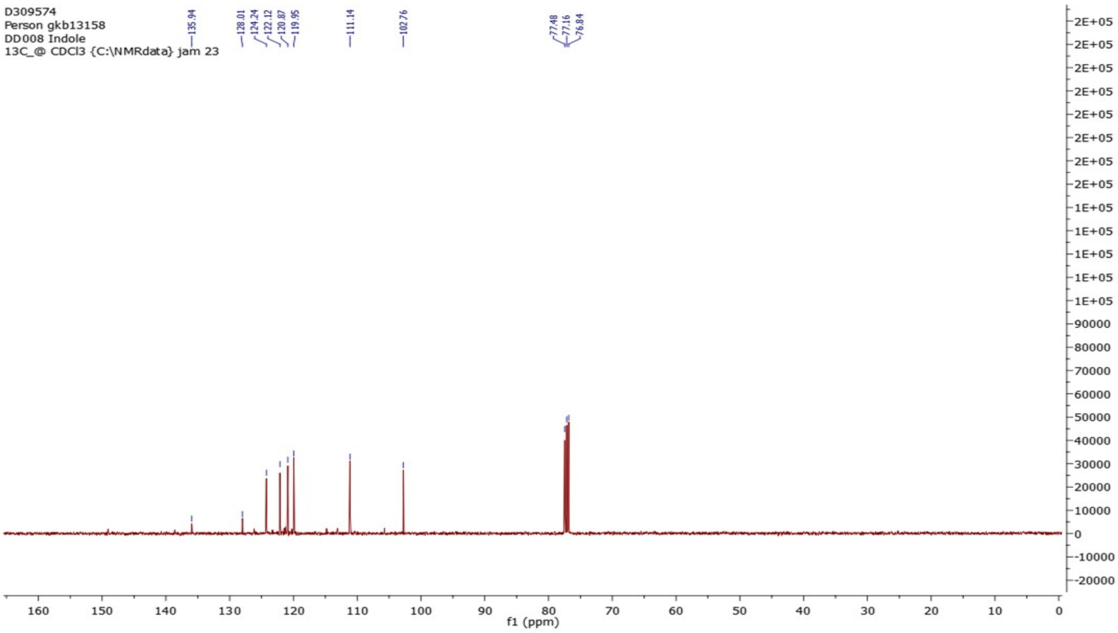

^{1}H and ^{13}C NMR Spectra of 9-Phenylacridine (**101**)

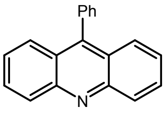

101

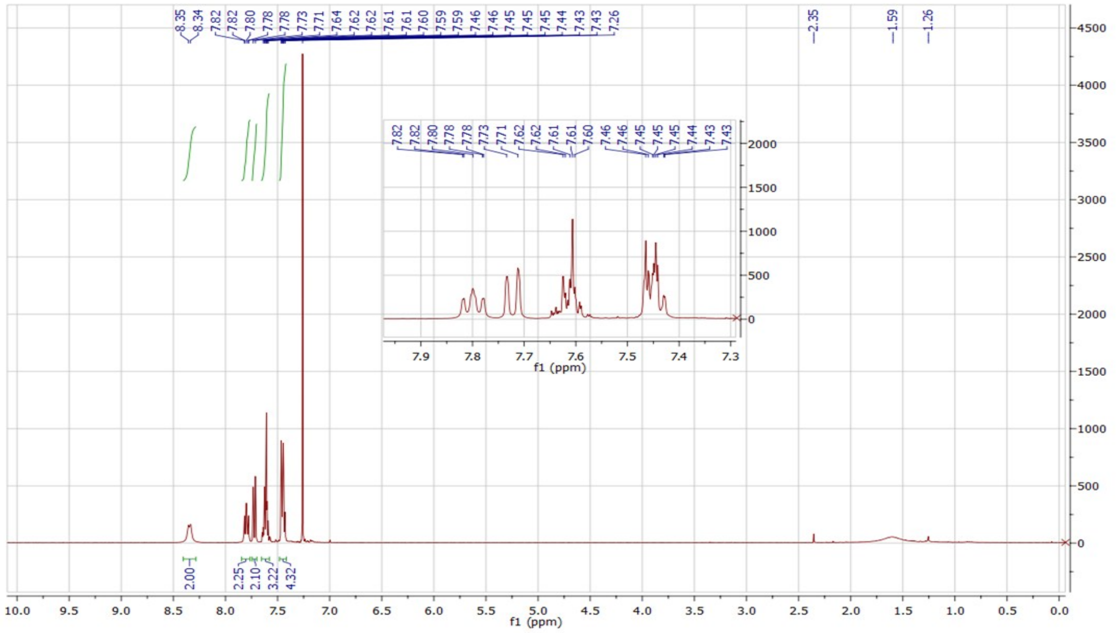

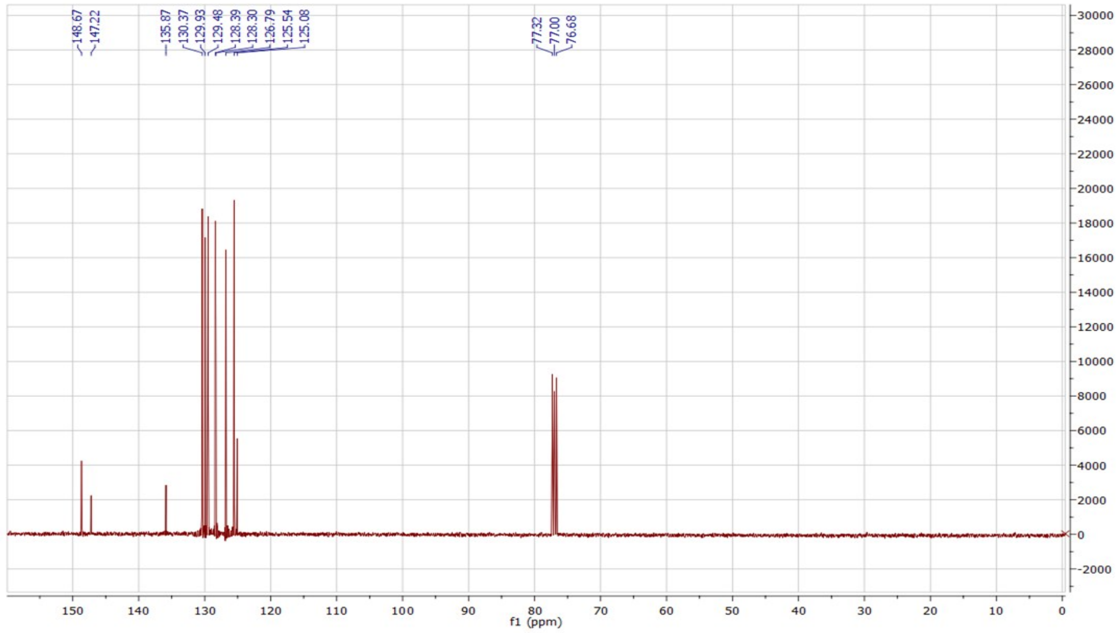

^{1}H, ^{2}D, and ^{13}C NMR Spectra of the Mixture of 9-Methyl-9,10-dihydroacridine (**30**), 9-(Methyl-*d*)-9,10-dihydroacridine (**30'**), and 9-Methyl-9,10-dihydroacridine-9-*d* (**30''**)

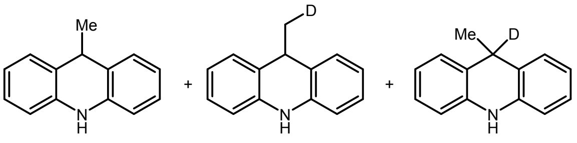

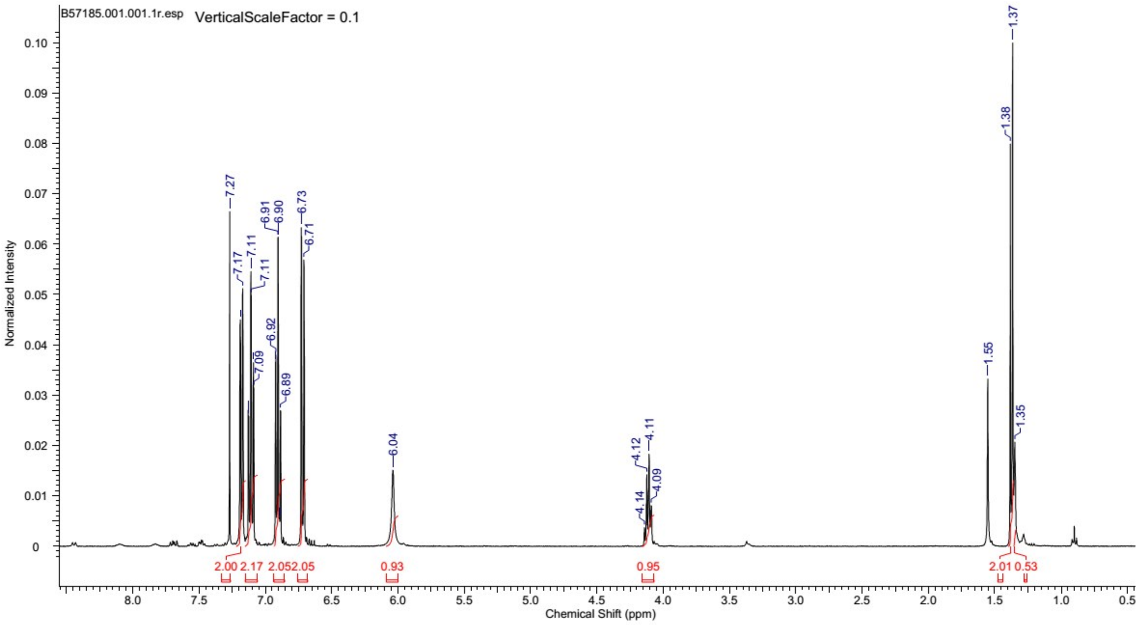

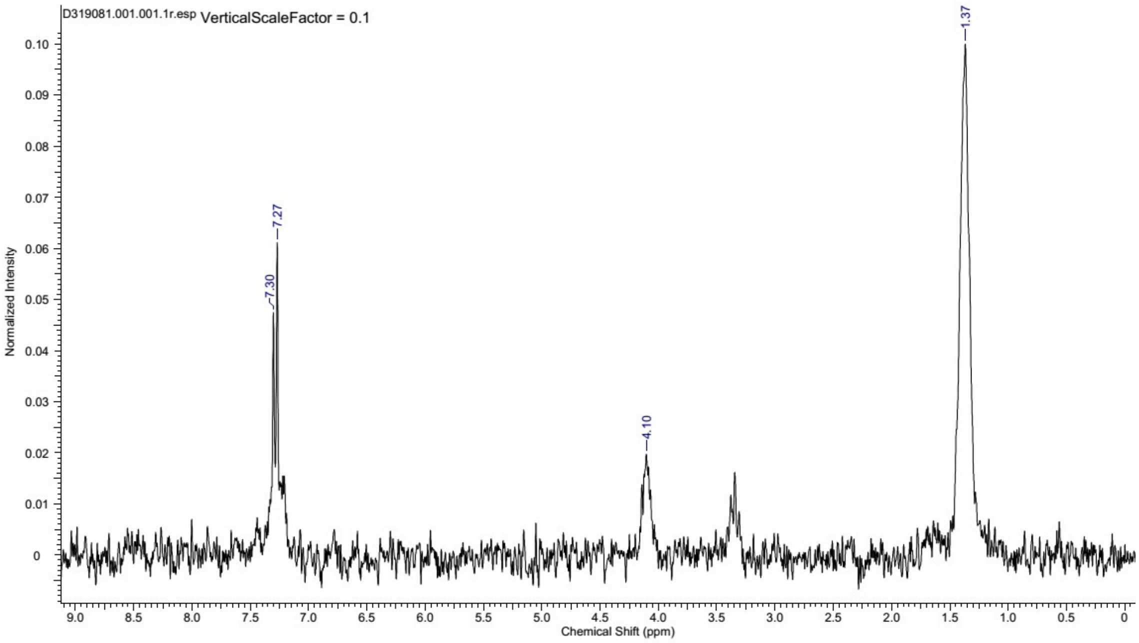

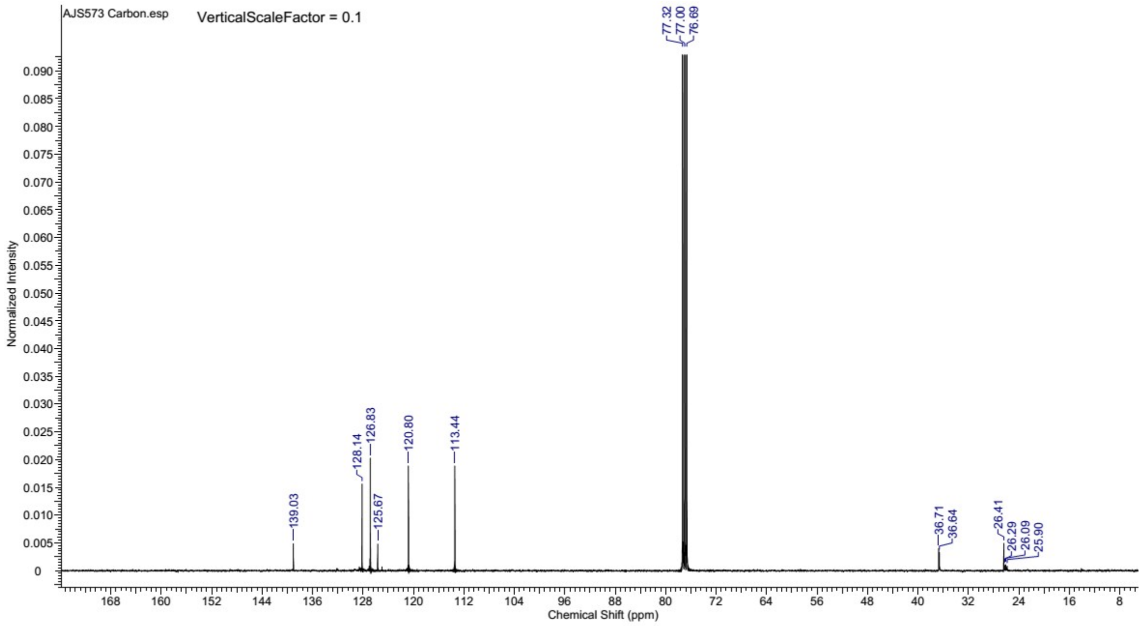

^{1}H, ^{2}D, and ^{13}C NMR Spectra of the Mixture of 9,9-Dimethyl-9,10-dihydroacridine (**31**) and 9-Methyl-9-(methyl-*d*)-9,10-dihydroacridine (**31'**)

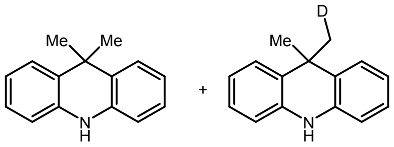

31 **31'**

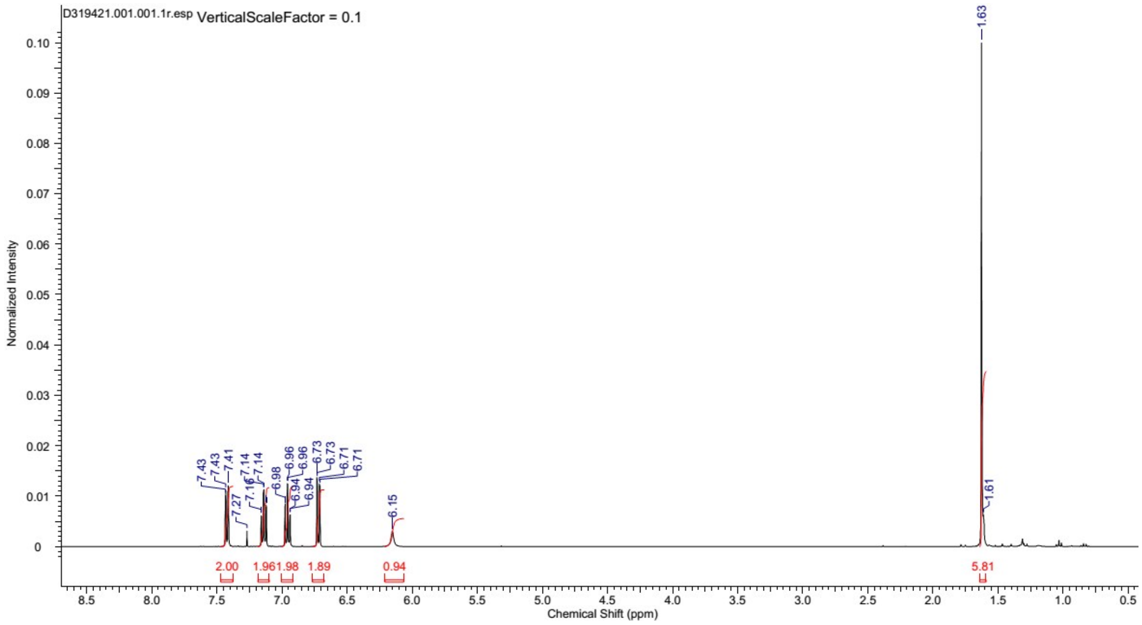

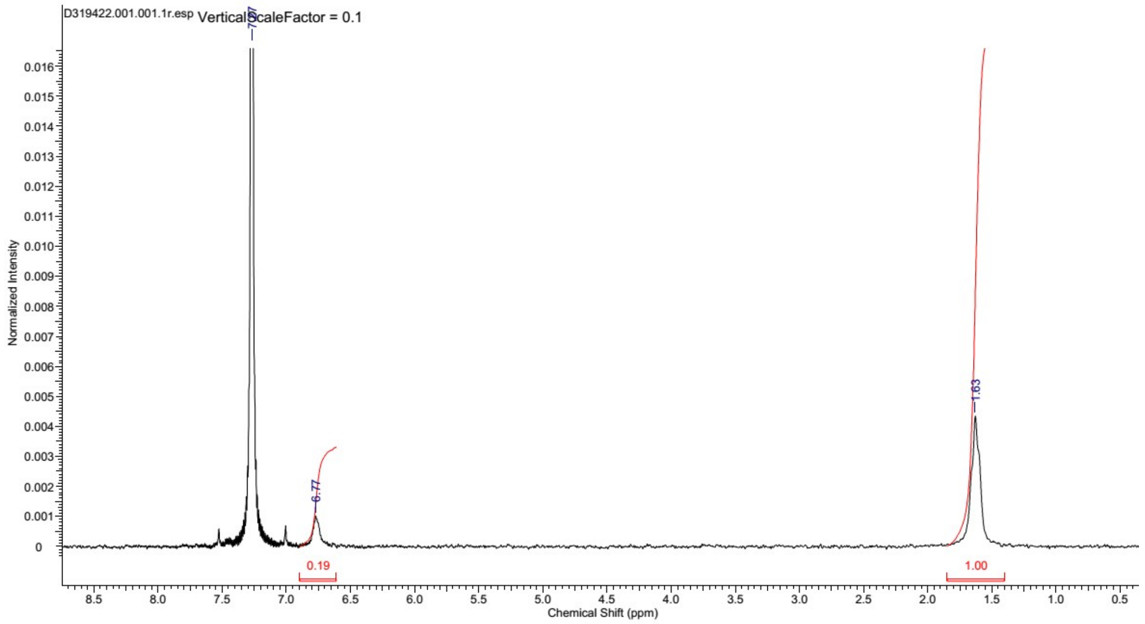
D319422.001.001.1r.esp VerticalScaleFactor = 0.1
7.27
6.77
1.63
0.19
1.00
Normalized Intensity
Chemical Shift (ppm)

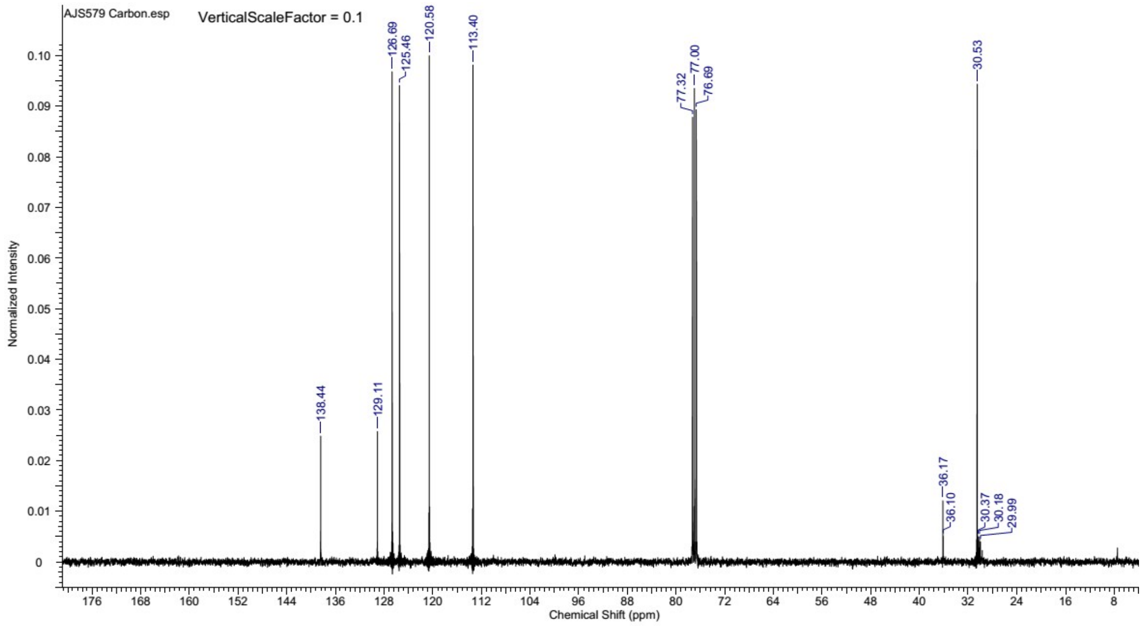
AJS579 Carbon.esp
VerticalScaleFactor = 0.1
138.44
129.11
126.69
125.46
120.58
113.40
77.32
77.00
76.69
36.17
36.10
30.53
30.37
30.18
29.99
Normalized Intensity
Chemical Shift (ppm)

Computational Results Radical anions and associated coordinates

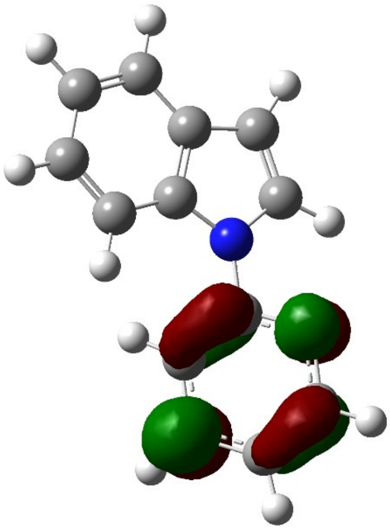

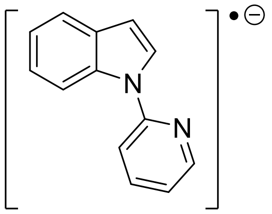

of N-(2-pyridyl)indole radical anion

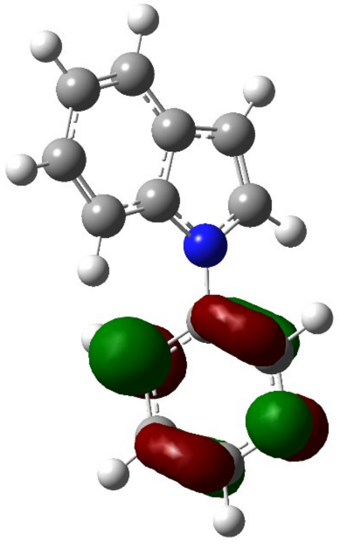

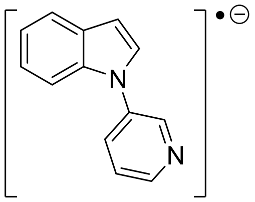

Figure S11. *SOMO of N-(3-pyridyl)indole radical anion*

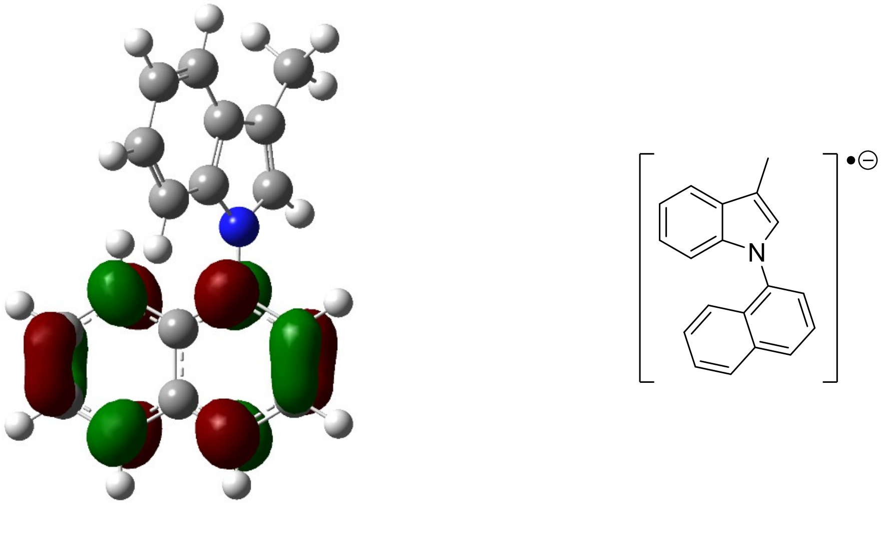

Figure S12. *SOMO of 3-methyl-N-(naphthalen-1-yl)indole radical anion*

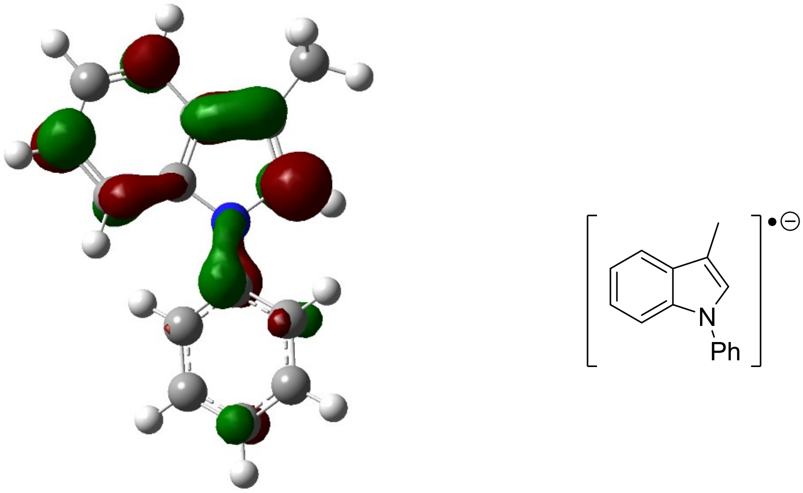

Figure S13. *SOMO of 3-methyl-N-phenylindole radical anion*

XYZ Coordinates

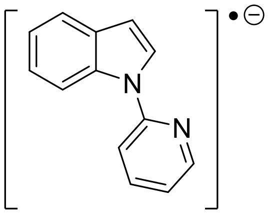

C	-2.20718600	0.82127700	-0.12799700
C	-1.11518800	-0.07887900	0.01687600
C	-1.32131500	-1.43976000	0.27690000
C	-2.62700400	-1.89411400	0.35465400
C	-3.72040100	-1.02181700	0.18141900
C	-3.51890800	0.32695200	-0.05050000
C	-1.64518300	2.12670600	-0.30226400
C	-0.28463000	1.97501500	-0.26040400
H	-0.48555900	-2.11058500	0.42628700
H	-2.81065000	-2.94283600	0.55844900
H	-4.72909100	-1.41444900	0.24178700
H	-4.36217900	0.99999100	-0.16434400
H	-2.17875200	3.05529900	-0.43147700
H	0.51024000	2.69912200	-0.33757800
N	0.04980600	0.65240100	-0.08965800
C	1.40398400	0.18366400	-0.03566300
C	1.74166100	-1.00021300	-0.64152600
C	3.09083400	-1.45771300	-0.58122000
H	0.98535000	-1.55660800	-1.18321400
C	4.00687900	-0.57904800	0.06958000
H	3.40116900	-2.38087100	-1.05027800
H	5.06178500	-0.82644700	0.13902300
N	2.25395900	1.03795000	0.60982700
C	3.56484000	0.60068500	0.61051000
H	4.27102600	1.26839900	1.09798100

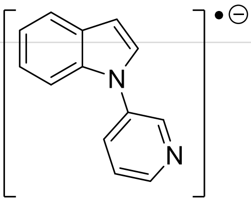

C	2.23302500	0.79170800	0.12114600
C	1.11116800	-0.06843300	-0.00580300
C	1.24777700	-1.43650400	-0.26902900
C	2.53165000	-1.94009500	-0.38135500
C	3.66161800	-1.10705500	-0.24166500
C	3.52282000	0.24721300	0.00358800
C	1.71662700	2.11002600	0.34156500
C	0.35051600	1.99607600	0.34516500
H	0.37132300	-2.06452600	-0.37374400
H	2.67282000	-2.99589600	-0.58234900
H	4.65204700	-1.53801600	-0.33447300
H	4.39629800	0.88338900	0.09915900
H	2.27984700	3.01757600	0.49172500
H	-0.41305800	2.74399900	0.49515900
N	-0.02784900	0.69222900	0.13393600
C	-1.37730400	0.21838900	0.06721100
C	-1.79319900	-0.84957400	0.91821000
C	-2.25157400	0.82108300	-0.80282700
C	-3.16659100	-1.21061000	0.76825100
H	-1.12940600	-1.29422500	1.64621000
H	-1.87926100	1.61759800	-1.44505000
C	-3.97253700	-0.54517300	-0.12042000
H	-3.59138400	-2.01283500	1.36400400
H	-5.01572800	-0.83802800	-0.21621500
N	-3.57607500	0.49615000	-0.93517200

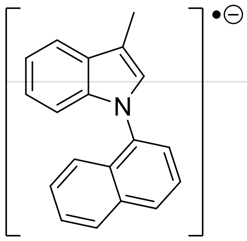

C	-2.73865700	-0.01197700	0.11249800
C	-1.44920800	0.21756500	-0.42956500
C	-1.17489900	1.30226800	-1.26833800
C	-2.22193300	2.15791700	-1.56544700
C	-3.51468500	1.94684000	-1.04148500
C	-3.78012200	0.87311800	-0.20794500
C	-2.65035100	-1.19608400	0.92185100
C	-1.34668400	-1.60913900	0.83693600
H	-0.17374600	1.45779600	-1.65387200
H	-2.04495800	3.01113500	-2.21028900
H	-4.30822800	2.64034100	-1.29564400
H	-4.77702300	0.71740400	0.19208100
H	-0.85924800	-2.45484300	1.30056600
N	-0.61063900	-0.77035700	0.02546400
C	0.77572200	-0.91910100	-0.29687200
C	1.71242500	0.01834000	0.21922300
C	1.16849600	-2.01651500	-1.08948200
C	1.35932500	1.10682900	1.05107800
C	3.10308200	-0.16969200	-0.12921800
C	2.50595300	-2.19253900	-1.40153000
H	0.40902000	-2.70089300	-1.45233300
C	2.33274800	2.00211300	1.52614900
H	0.31963500	1.24577600	1.32491400
C	4.04932500	0.75704100	0.36707200
C	3.46679000	-1.27923100	-0.93888300
H	2.81055300	-3.03449600	-2.01429800
C	3.66635600	1.83191700	1.18405000
H	2.03378400	2.82900200	2.16187900
H	5.09390000	0.61655300	0.10621700
H	4.51414300	-1.41578100	-1.18826000
H	4.41644600	2.52621000	1.54859900
C	-3.76219300	-1.82929500	1.69843600
H	-4.17553300	-1.13813000	2.43908600
H	-4.58460800	-2.13390900	1.04404800

H	-3.40860300	-2.71590600	2.22788900

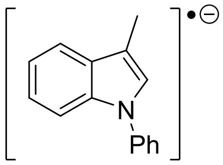

C	-2.09246900	0.28660900	-0.07966100
C	-0.88159700	-0.48167200	0.01613100
C	-0.90272300	-1.85214200	0.22241100
C	-2.15690000	-2.50943200	0.28204300
C	-3.34584500	-1.77829200	0.14304400
C	-3.34267800	-0.40256400	-0.03091400
C	-1.74530600	1.64583200	-0.12729800
C	-0.35351500	1.73923000	-0.04342700
H	0.00396500	-2.42210300	0.37053500
H	-2.19030700	-3.58072000	0.43895800
H	-4.29527000	-2.30475100	0.18325200
H	-4.27374100	0.14778500	-0.11850800
H	0.28680800	2.57542400	-0.27823100
N	0.18652500	0.43135100	-0.02499200
C	1.54278800	0.16080200	-0.01108200
C	2.07437000	-1.05240200	-0.49789100
C	2.45202300	1.14797100	0.44514200
C	3.44106500	-1.27859700	-0.49212200
H	1.41280400	-1.78982200	-0.93004900
C	3.81660500	0.89678200	0.45071200
H	2.07179600	2.08616500	0.82808300
C	4.33495800	-0.31464000	-0.00704600
H	3.81805200	-2.21540600	-0.88876600
H	4.48766300	1.66322600	0.82512600
H	5.40187200	-0.50014200	-0.00038600
C	-2.70066400	2.79523000	-0.20197100
H	-3.37196500	2.70549700	-1.06267700
H	-3.33324900	2.85609100	0.69193600
H	-2.16476700	3.74299800	-0.29275200

Probing the conversion of **43** to **44**

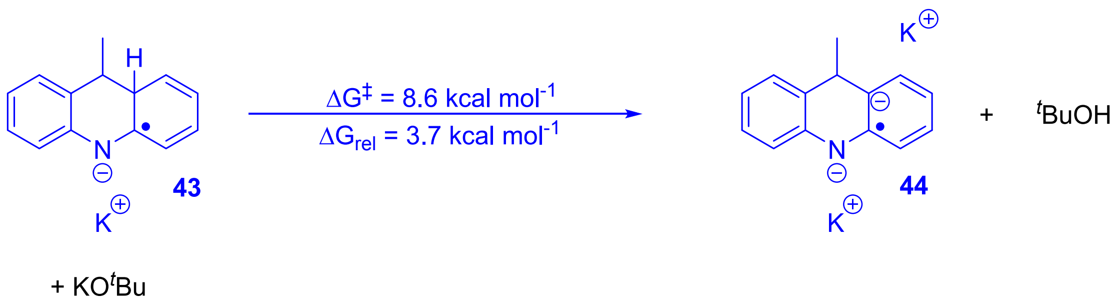

Coordinates

Reactant Complex for [the formation of **44** from] **43** and **KO^{t}Bu**

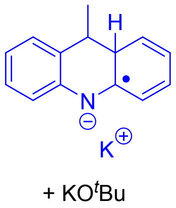

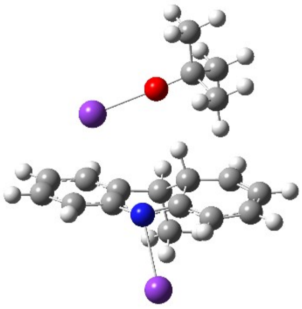

C	1.62458400	3.59030100	0.79854800
C	1.22719700	2.47385200	1.54152000
C	1.16256200	1.20470400	0.98053600
C	1.49897000	1.01238200	-0.39244600
C	1.92145500	2.14995700	-1.12347000
C	1.98256400	3.40849500	-0.54000800
C	0.79102200	-0.01658900	1.78285600
C	0.81007500	-1.22607700	-0.41637100
C	0.01752300	-0.97603600	0.85253000
C	-0.42097300	-2.24232100	1.53129200
H	-0.86490800	-2.14306500	2.51809900
C	-0.35131800	-3.45923100	0.93097000
C	0.21606100	-3.60773100	-0.36629300
C	0.79830000	-2.49954000	-0.99640300
H	1.66872700	4.57042200	1.25688000
H	0.96028600	2.59106700	2.58864800
H	2.18080000	2.01292000	-2.16816700
H	2.30566500	4.25677100	-1.13405400
H	-0.73604700	-4.33489400	1.44376000
H	0.21329400	-4.57424300	-0.85450600
H	1.21269500	-2.60632500	-1.99756400
H	0.12271700	0.27851600	2.59937300

H	-0.88399600	-0.40856500	0.53734800
C	-3.61178400	-0.16256300	-0.31995500
C	-4.95538400	0.31692100	-0.90809400
H	-5.32345100	1.16963700	-0.32921300
H	-4.80357200	0.64521600	-1.94092100
H	-5.72155600	-0.46653700	-0.90056500
C	-3.12847500	-1.37311800	-1.14734100
H	-2.94832300	-1.05632100	-2.17952800
H	-2.18913700	-1.75852000	-0.73920100
H	-3.85976700	-2.18960000	-1.15699200
C	-3.86152300	-0.63409600	1.12811700
H	-2.92439100	-0.99754400	1.56014800
H	-4.21394300	0.20982700	1.72926700
H	-4.60325100	-1.43871700	1.18628600
O	-2.68316400	0.85160200	-0.34650600
K	-1.16745000	2.62428700	-0.55968800
N	1.46518000	-0.20673200	-1.02055100
K	3.69228000	-1.59288100	-1.23250600
C	2.04082600	-0.66484000	2.38847300
H	2.56104400	0.03526900	3.04579200
H	1.77778200	-1.55929600	2.95785200
H	2.73651600	-0.96524700	1.59808100

Transition State for the formation of **44** from **43** and **KO*t*Bu**

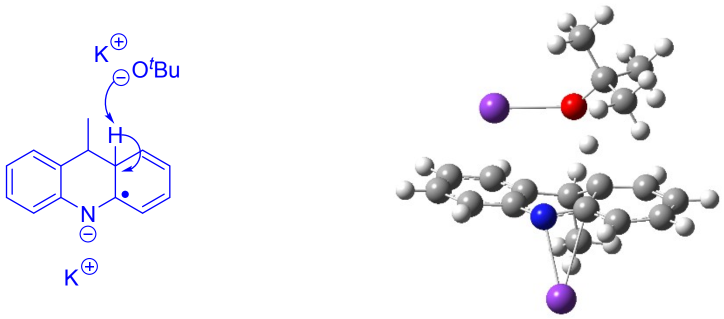

C	1.40798300	3.62239700	0.80418300
C	0.99900800	2.48980300	1.52344100
C	0.99371300	1.21977300	0.96619300
C	1.40309600	1.02557700	-0.39501600
C	1.85184400	2.18260300	-1.09474600
C	1.85411100	3.44158400	-0.50926400
C	0.62950500	-0.00750600	1.76361300

C	0.72886700	-1.22055100	-0.43230400
C	-0.02670200	-1.02657400	0.82277000
C	-0.48104100	-2.27238100	1.45683900
H	-0.90839000	-2.20256800	2.45530700
C	-0.45993500	-3.48410100	0.81205700
C	0.09777100	-3.59967300	-0.48197100
C	0.70592800	-2.46275000	-1.06636900
H	1.41020600	4.60222400	1.26520400
H	0.67605100	2.59841900	2.55633700
H	2.17718900	2.05444500	-2.12241600
H	2.19998200	4.29439100	-1.08497000
H	-0.87514000	-4.36204900	1.29803100
H	0.08685800	-4.54685700	-1.00759900
H	1.11638200	-2.52844800	-2.07374900
H	-0.09416800	0.27280100	2.53934700
H	-1.12613900	-0.32580200	0.43022600
C	-3.24091500	-0.24672500	-0.27067100
C	-4.22622500	0.73279200	-0.92132700
H	-4.46476200	1.54161100	-0.22362400
H	-3.78281600	1.16582900	-1.82457800
H	-5.15976600	0.23894600	-1.20822000
C	-2.88847500	-1.35491000	-1.27346400
H	-2.41080000	-0.91698100	-2.15543600
H	-2.18737600	-2.06370100	-0.82499200
H	-3.78184800	-1.89994800	-1.59490800
C	-3.89048300	-0.86954000	0.97264600
H	-3.18693100	-1.56193100	1.44202800
H	-4.13901700	-0.08465100	1.69274300
H	-4.80424100	-1.41724600	0.72159900
O	-2.08752800	0.46432000	0.09443300
K	-1.18272200	2.64724400	-0.65699300
N	1.39446700	-0.17421700	-1.03287400
K	3.58050800	-1.63848600	-1.05314100
C	1.88151300	-0.56691100	2.45933900
H	2.32194000	0.16550500	3.14165900
H	1.63670600	-1.47464100	3.01715800
H	2.63974400	-0.82826200	1.71236500

Product Complex for **44** and **HO*t*Bu**

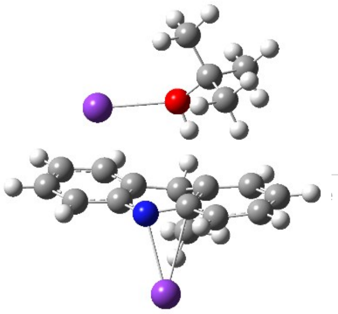

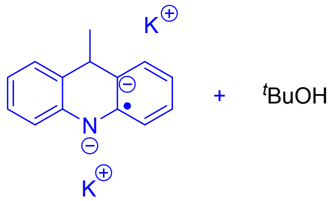

C	1.81526500	3.45814700	0.77545700
C	1.28213500	2.37603000	1.49745100
C	1.14304600	1.10939500	0.94821200
C	1.53079100	0.86173600	-0.41351100
C	2.13017900	1.96117700	-1.10731300
C	2.26528900	3.21483700	-0.52661100
C	0.68349300	-0.06492800	1.78033700
C	0.59735600	-1.29005600	-0.43162600
C	0.04893300	-1.10381100	0.88401900
C	-0.61975100	-2.23917600	1.48841100
H	-0.97170700	-2.14961600	2.51418000
C	-0.85523300	-3.40376300	0.78001400
C	-0.40714300	-3.54472800	-0.54563700
C	0.34746500	-2.47710700	-1.12717300
H	1.92366300	4.43350400	1.23306800
H	0.97522600	2.52177300	2.53091300
H	2.46031100	1.78967400	-2.12710700
H	2.72039000	4.01759400	-1.09899900
H	-1.39837100	-4.21739200	1.25343700
H	-0.60281800	-4.45038700	-1.10717600
H	0.64786600	-2.52416300	-2.17287100
H	-0.05566100	0.28984200	2.51495400
H	-1.41703100	0.04904600	0.29960000
C	-3.34077800	0.07619000	-0.22610000
C	-4.26352700	1.14016200	-0.80834100
H	-4.37628200	1.97070100	-0.10605200
H	-3.85550900	1.52323100	-1.74826400
H	-5.25265800	0.72321500	-1.01102300
C	-3.12225500	-1.06475800	-1.21661300
H	-2.71055800	-0.67621500	-2.15230500
H	-2.41645200	-1.79353500	-0.80700500
H	-4.06588300	-1.57265000	-1.43402000

C	-3.89450300	-0.45715100	1.09398400
H	-3.19930200	-1.18825300	1.51541600
H	-4.02336200	0.36131600	1.80688600
H	-4.86117000	-0.94406300	0.94076200
O	-2.09028300	0.73071800	0.01969600
K	-0.78742400	2.78740300	-0.74588500
N	1.36381700	-0.31168500	-1.06193400
K	3.30481500	-2.10318900	-1.06798600
C	1.87571900	-0.63286000	2.58377900
H	2.28540300	0.10571000	3.27973300
H	1.56324600	-1.51780400	3.14494100
H	2.67689200	-0.93155400	1.89801300
